# Development of Potent and Selective RIPK1 Degraders Targeting Its Nonenzymatic Function for Cancer Treatment

**DOI:** 10.1021/acs.jmedchem.5c01340

**Published:** 2025-07-16

**Authors:** Zhen Zhang, Chunrong Li, Nina J. Hawkins, Ramesh Mudududdla, Yiming Nie, Peng-Kai Liu, Penghsuan Huang, Natalia M. Del Rio, Hao Chang, Matthew E. Brown, Lingjun Li, Weiping Tang

**Affiliations:** † Lachman Institute for Pharmaceutical Development, School of Pharmacy, 5228 University of Wisconsin−Madison Madison, Wisconsin 53705, United States; ‡ Biophysics Graduate Program, University of Wisconsin−Madison, Madison, Wisconsin 53705, United States; § Department of Chemistry, University of Wisconsin−Madison, Madison, Wisconsin 53706, United States; ∥ Department of Surgery, School of Medicine and Public Health, University of Wisconsin−Madison, Madison, Wisconsin 53792, United States; ⊥ Department of Dermatology, School of Medicine and Public Health, University of Wisconsin−Madison, Madison, Wisconsin 53705, United States

## Abstract

Receptor-interacting protein kinase 1 (RIPK1) is a threonine/serine kinase that serves as a critical regulator of immune responses and cell death pathways, functioning through both its kinase activity and nonenzymatic scaffolding function. The scaffolding function of RIPK1 contributes to both intrinsic and extrinsic resistance to immune checkpoint blockades (ICBs), making it a compelling therapeutic target for cancer treatment. Recent studies have highlighted RIPK1’s potential as a key modulator for improving the efficacy of immune-stimulatory therapies, such as ICBs and X-ray radiotherapy (XRT). In this study, we have developed a highly potent and selective RIPK1 degrader. When combined with XRT, the degrader significantly suppressed tumor growth, achieving enhanced therapeutic efficacy without apparent adverse effects. In contrast, the RIPK1 inhibitor showed no notable therapeutic effect. These findings underscore the potential of targeting RIPK1 degradation, specifically its nonenzymatic function, as a novel strategy to augment the effects of radiotherapy.

## Graphical abstract

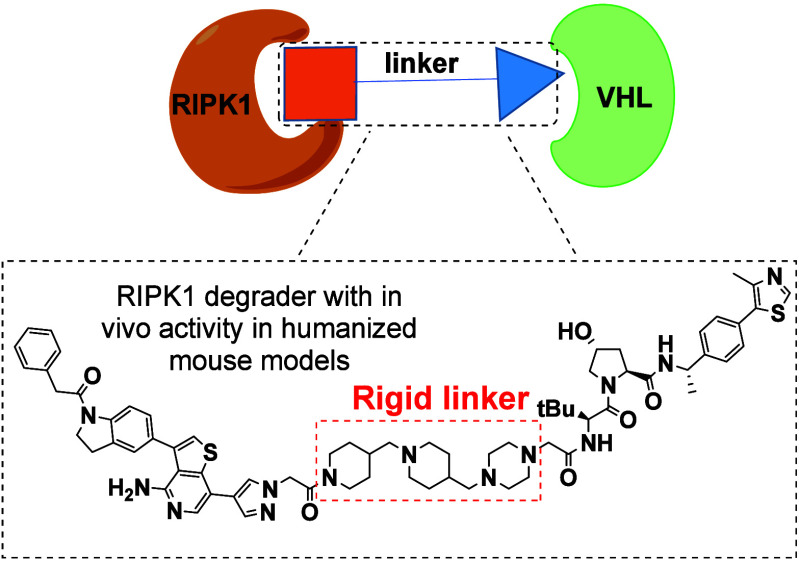

## Introduction

Receptor-interacting protein kinase 1 (RIPK1) is a serine/threonine kinase and a critical stress sentinel that plays a dual role in regulating cell death, innate immunity, and inflammation.^1,2^ It functions through both its kinase-dependent and kinase-independent (scaffolding) mechanisms, modulating pro-inflammatory responses downstream of multiple innate immune pathways, including those triggered by tumor necrosis factor-α (TNF-α), toll-like receptor (TLR) ligands, and interferons (IFNs).^3^ While RIPK1’s enzymatic kinase activity is essential for initiating cell death, its nonenzymatic scaffolding function is crucial for regulating pro-inflammatory and pro-survival signaling.^4–10^ These findings suggest that RIPK1’s disease-associate activities cannot be fully addressed by kinase inhibitors alone,^11^ which have primarily been developed for the treatment of autoimmune, inflammatory, and neurodegenerative diseases.^12^ However, targeting RIPK1’s nonenzymatic scaffolding function is challenging due to the absence of a well-defined binding pocket within the intermediate domain responsible for this activity. Moreover, previous studies have shown that knockout of RIPK1 led to a more significant inhibition of cell growth by UVB radiation, and up-regulation of inflammatory factors like IL-1α, further emphasizing RIPK1’s role in pro-survival signaling in epidermal cells.^13^ These insights highlight the need for alternative therapeutic strategies, such as developing targeted RIPK1 degraders, to disrupt its scaffolding function. Such approaches may offer a promising avenue for treating RIPK1-driven diseases and enhancing the efficacy of immune-stimulatory therapies, including immune checkpoint blockade (ICB) and X-ray radiotherapy (XRT).

Several RIPK1 inhibitors have progressed to Phase I clinical trials,^14,15^ including those for active ulcerative colitis^16^ and psoriasis,^17^ though none have yet received regulatory approved for clinical use. Proteolysis targeting chimera (PROTAC) represents a transformative therapeutic approach that leverages the body’s natural protein disposal system to degrade disease-causing proteins with small molecules.^18–20^ This technology offers an effective means for the elimination of the entire target protein including both enzymatic activity and nonenzymatic scaffolding functions. Additionally, PROTACs operate through a catalytic mechanism, allowing for repeated degradation of the target protein. This enhanced activity enables effective therapeutic outcomes at lower doses, improving selectivity toward diseased tissues and reducing off-target effects. Consequently, PROTACs offer the potential for targeting challenging proteins like RIPK1, where the nonenzymatic scaffolding functions contribute to disease pathology.

In 2024, three groups,^21–23^ along with ours,^24^ independently reported the development of RIPK1 PROTACs. Two of these degraders, R1-ICR-5 and LD4172, are based on type II RIPK1 inhibitors,^21,22^ which bind to the inactive kinase conformation. Both demonstrated in vivo efficacy in syngeneic mouse models when combined with anti-PD1 therapy. One was administered via intratumoral injection,^21^ while the other one was delivered intraperitoneally (IP).^22^ In contrast, the third RIPK1 degrader, MS2031, is based on a type III RIPK1 inhibitor,^23^ which primarily targets an allosteric site. These reports underscore the growing interest in developing effective PROTAC-based therapies for RIPK1-associated diseases. However, the in vivo efficacy of compounds R1-ICR-5 and LD4172 has only been demonstrated in murine cancers with syngeneic mouse models, while MS2031 has only shown cellular activity. In addition, the above three PROTACs all utilized long, flexible alkyl linkers, which often result in suboptimal physiochemical properties and pharmacological profiles, potentially limiting their translational potential. We have developed a highly potent and selective RIPK1 PROTAC featuring a rigid linker.^24^ For the first time, we demonstrate the in vivo antitumor efficacy of RIPK1 PROTAC in combination with XRT in a human cancer model in humanized mouse system.

## Results and Discussions

### Design and Synthesis of RIPK1 Degraders

We designed RIPK1 PROTACs based on GSK’074 (Figure 1A), a RIPK1/RIPK3 dual small-molecule inhibitor previously reported by us (*K*_d_ = 12 nM for RIPK1 and *K*_d_ = 130 nM for RIPK3).^3^ The plan is to use our Rapid-TAC platform^25^ to generate the first set of PROTACs by coupling a hydrazide-containing RIPK1 inhibitor with the aldehyde group in our preassembled partial PROTAC library to quickly identify the appropriate E3 ubiquitin ligase and the approximate linker length (Figure 1B). To guide our design, molecular docking studies of GSK’074 with the kinase domain of human RIPK1 in a DFG-out conformation were performed (Figure 1C). The hydrazide moiety was strategically placed on the pyrazole ring, one of the solvent-exposed regions (Figure 1B). Following our published procedures, 12 VHL ligand-based and 12 CRBN ligand-based PROTACs were quickly prepared in dimethyl sulfoxide (DMSO) under miniaturized conditions. These PROTACs were tested for their ability to degrade RIPK1. We found that VHL ligand-based PROTACs effectively induced degradation of RIPK1 and compound DY-2 (VHL ligand and n = 3, Figure 1D) is the most potent and selective degrader.

**Figure 1:**
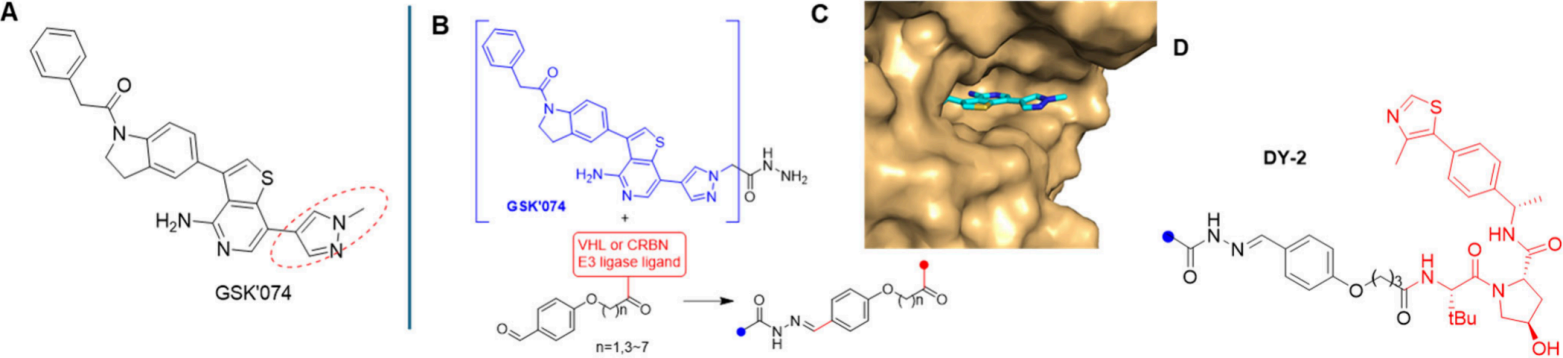
A) Structure of GSK’074 (The solvent-exposed pyrazole ring is circled). B) Quick identification of the linker length and E3 ubiquitin ligase for RIPK1 PROTAC using our Rapid-TAC platform, where the GSK’074 with a hydrazide motif is coupled with an aldehyde-containing partial PROTAC library. C) Molecular docking of GSK’074 and kinase domain of human RIPK1 in a DFG-out conformation (PDB: 3IDP). D) Structure of DY-2.

Since the acylhydrazone motif in DY-2 is hydrolytic labile, we next replaced the C=N double bond, by a C–C single bond to prepare more stable RIPK1 degraders. However, this change may influence the required optimal linker length. To cover a wider range of linkers, we prepared RIPK1 degraders with stable and flexible linkages that contain a benzene ring to mimic DY-2, such as 204-2, 204-4, 204-6; 214-6, 214-7, 214-8, 214-9; 217-10, 217-11, 217-12, 217-13; and 224-5, 224-6, 224-7, 224-8 (Figure 2). Degrader 204-6, 214-8, 217-13, 224-5 bear the same linker length as DY-2, while 204-4, 214-7, 217-12 have one less carbon and 204-2, 214-6, 217-11 have two less carbons. Compounds 214-9, 214-10, 224-6 have one or two more atoms. In addition to these RIPK1 degraders with flexible linkages that contain a benzene ring, we also prepared a series of degraders with completely flexible alkyl and PEG linkages 216-9, 216-10, 216-11, 216-12, 216-13, 216-14, 216-15, 216-16; 208-2, 208-4, 208-6, and a series of degraders with more rigid linkers. In compounds with rigid linker, compound 225-2 features two connected heterocycles within the linker. Compounds 225-6, 225-7, and 229-1 through 229-4 have similar linker lengths but differ in the positions of their heterocyclic rings and the connections. Compounds 225-3 through 225-5 bear one, two, three piperidine rings in the linker part. (Figure 2).

**Figure 2:**
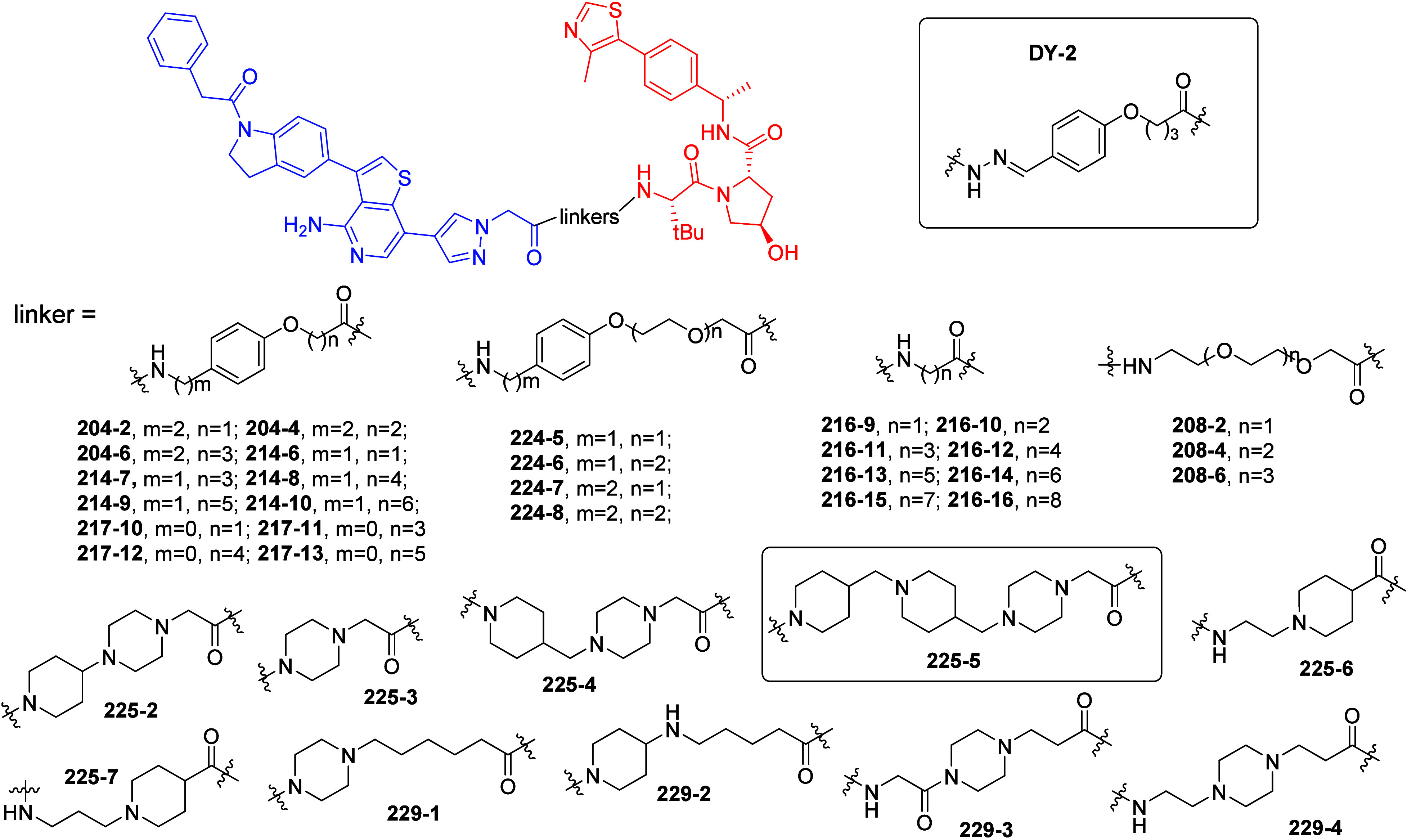
Structure of RIPK1 PROTACs with various flexible and rigid linkers.

### Screening of RIPK1 Degraders Using HEK-293T-RIPK1-HiBiT cells

We fused a HiBiT tag to the C-terminal of RIPK1 of HEK293T cells using the CRISPR-Cas9 system. These cells were used to measure compound induced RIPK1 degradation by monitoring luminescent signal using a luminescent plate-based reader and the luminescent signal were visualized Chemidoc MP imaging system. For screening, the HiBiT cells were seeded in 96-well plates and treated with different concentrations of compounds for 24 h (Figure 3A). Generally, compounds with alkyl linkers bearing a benzene ring or simple alkyl linkers demonstrated better activity compared to those with PEG linkers (Figure 3B). Among compounds with simple alkyl linkers or alkyl linkers bearing a benzene ring, 204-2, 214-8/9, 217-10/11/12, 216-15/16, and 224-5/6 exhibited stronger activity than others within the same series. Many of these compounds share similar linker lengths. For example, compound 216-16 has the same linker length as 204-2. Additionally, compounds in the 217 series has a relatively electron-rich benzene ring with an electron-donating oxygen and nitrogen groups at the para-position, which may introduce metabolic liability issues related to the formation of quinone. Given this, along with the preference for compounds with shorter linkers, degrader 204–2 was selected for comparison with those featuring rigid linkers.

**Figure 3:**
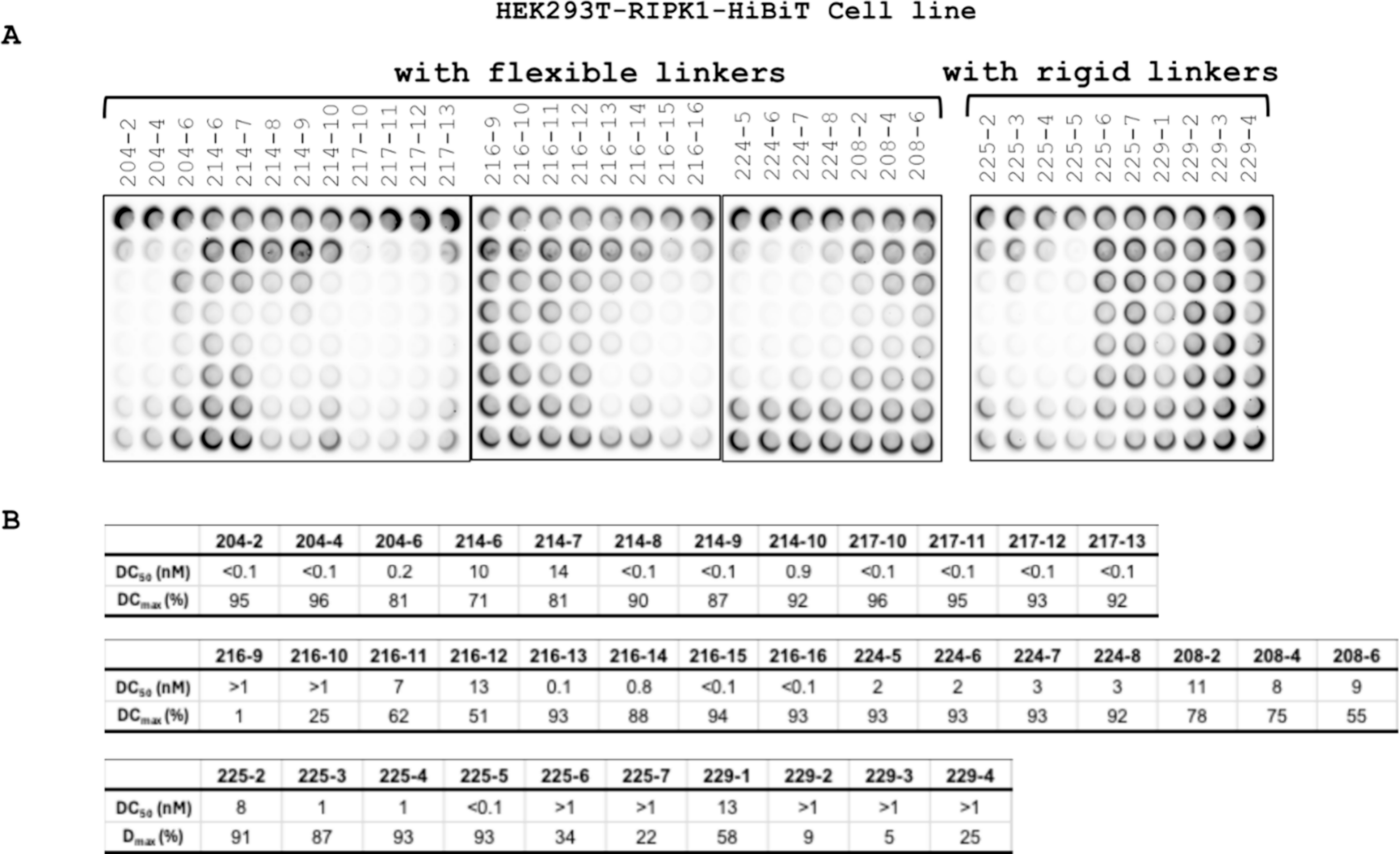
Primary screening RIPK1 degraders using HEK293T-RIPK1-HiBiT cell line. Cells were seeded in 96-well plates and treated with test compounds for 20 h. After treatment, Bioluminescence signal reflecting RIPK1 protein level was generated by adding Nano-Glo HiBiT detect reagent. A) Bioluminescence signal was visualized using Bio-Rad ChemiDoc MP Imaging system. The grayscale intensity is positively correlated with protein abundance. B) Relative luminescence Units (RLUs) in each well was measured using CLARIOstar plate reader. DC_50_ values were calculated by nonlinear regression analysis in GraphPad Prism software. DC_50_ = dose that reduces RIPK1 protein by 50%. *D*_max_ = maximum reduction of RIPK1 protein achieved by the compound.

For the compounds with rigid linkers, we found that compounds 225-3/4/5 bearing one, two, or three piperidine rings in the linker exhibited the strongest activity. Interestingly, a marked difference was observed for compounds 229-1 and 229-2, which share the same linker length. Substituting the N-CH_2_ motif in 229-1 with a CH-NH group in 229-2 completely abolished the degradation activity of the latter. Similarly, compounds 229-3 and 229-4 also have the same linker length, but replacing a methylene group in 229-4 with a carbonyl in 229-3 again once again resulted in complete loss of degradation activity. These sharp changes in RIPK1 degradation across this series of compounds are striking, highlighting that the orientation of the rigid linker is critical for effectively positioning RIPK1 in proximity to the E3 ligase for ubiquitination.

### 204-2 and 225-5 Effectively Degrade RIPK1 in a Diverse Range of Cancer Cell Lines

Based on the primary screening results, we further evaluated the degradation activity of the most potent degraders with distinct linker types, compound 204-2 featuring a flexible linker and compound 225-5 featuring a rigid linker, across a panel of human and murine cancer cell lines. Both compounds exhibited potent degradation activity in most human cancer cell lines but showed limited activity in murine cancer cells (Figure 4). Notably, compound 225-5 elicited a relatively stronger response than 204-2 in B16F10 murine cells. We selected compound 225-5 for further investigation in both B16F10 and human melanoma A375 cells.

**Figure 4:**
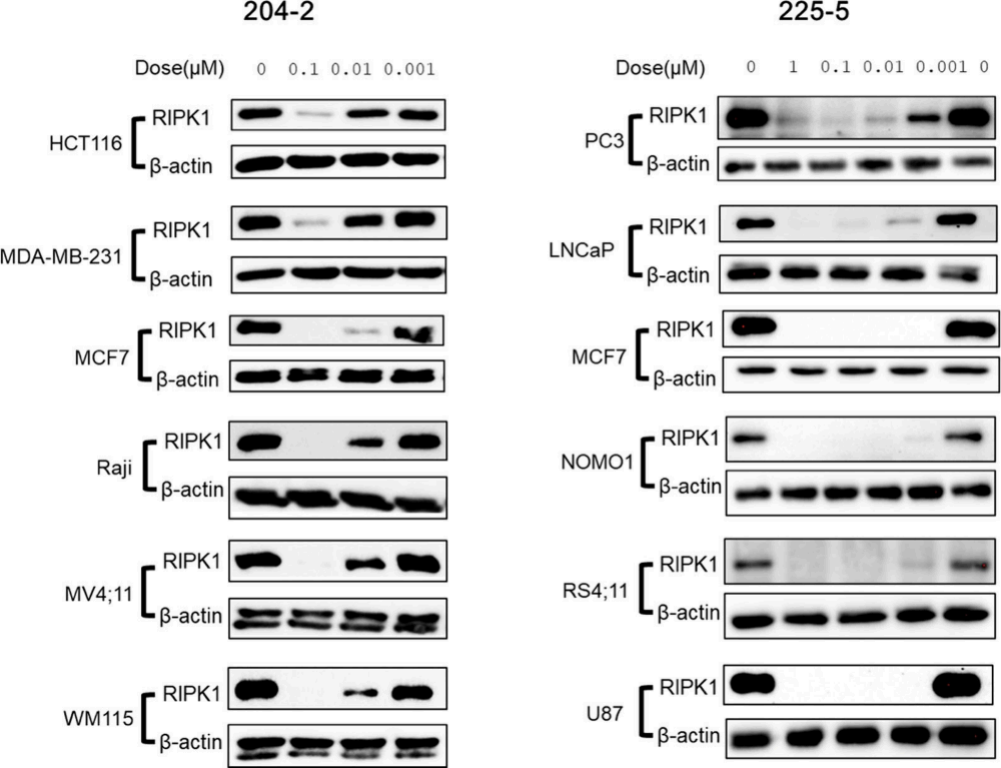
204–2 and 225–5 degraded RIPK1 in various cancer cell lines. Cancer cells were treated with 204–2 or 225–5 for 20 h. RIPK1 protein levels were evaluated by Western blot.

Cells were treated with 1 μM of compound 225-5 and RIPK1 protein levels were monitored over time using Western blot analysis. Significant RIPK1 degradation was observed as early as 1 h post-treatment. The degradation was sustained over the course of 20 h (Figure 5A). Following 24 h of treatment, compound 225-5 induced RIPK1 degradation in a dose-dependent manner in both cell lines, with a DC_50_ of 41 nM and *D*_max_ of 97% in A375 cells and a DC_50_ of 91 nM and *D*_max_ of 92% in B16F10 cells (Figure 5A,B).

**Figure 5:**
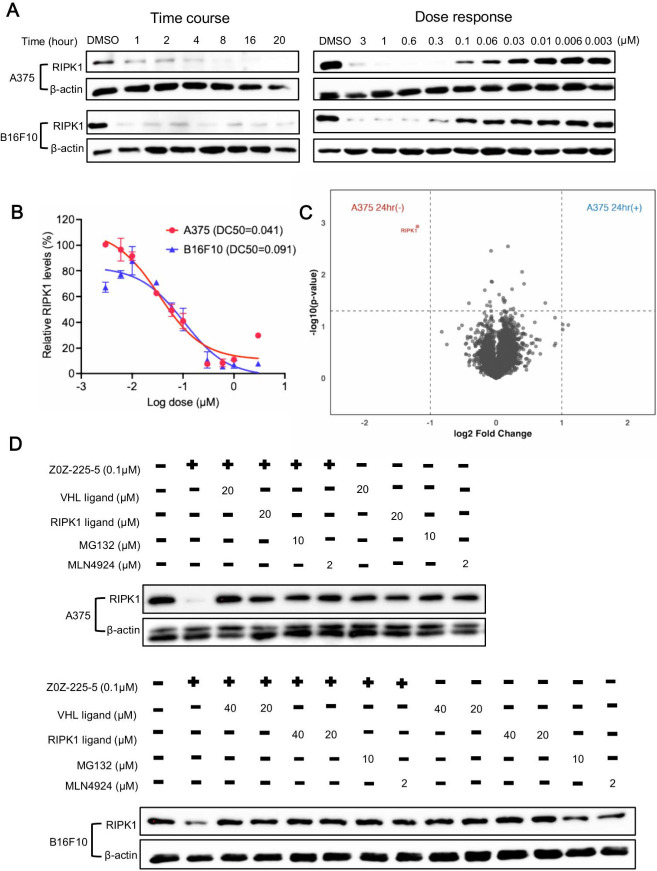
Activity, selectivity and mechanism of degrader 225-5 in human A375 and murine B16F10 melanoma cells. A) Time course (0.1 μM and 1.0 μM of 225-5 in A375 and B16F10, respectively); (A,B) dose response (20 h treatment); C) proteomic profiling in A375 cells after 24 h treatment of 255-5 (100 nM). Volcano plots showed protein expression level changes for 225-5 versus DMSO group. Log2 protein fold changes are plotted against the negative log10 *p*-values. Proteins exhibiting significant alternations (*p*-value <0.05, Student’s *t* test) are represented by points above the nonaxial horizontal line. Significantly down-regulated proteins are depicted in red. (protein |fold change| > 2); D) A375 and B16F10 cells were pretreated with DMSO, 10 μM of MG132, 20 μM of VH-032, 2 μM of MLN4924, and 20 μM of GSK’074 for 1 h followed by incubation with 0.1 μM of 225–5 for 2h. Cell lysates were collected, and RIPK1 levels were detected by Western blot.

To systematically assess proteome-wide changes induced by 225-5, we performed a global quantitative proteomic analysis in A375 cells using mass spectrometry. Cells were treated with either DMSO or 0.1 μM 225-5 for 24 h, with the dose and time selected based on prior time-course and dose-titration experiments (Figure 5C). A total of 5717 proteins were quantified. Notably, RIPK1 was selectively and significantly downregulated in the 225-5-treated group. No other proteins showed significant changes (≥2-fold, *p* ≤ 0.05) compared to the DMSO control.

We then validated the mechanisms of action of compound 225-5. As shown in the Figure 5D, pretreatment of the A375 and B16F10 cells with RIPK1 ligand GSK’074, VHL ligand VH-032, the proteasome inhibitor MG132, or neddylation inhibitor MLN4924 all abrogated RIPK1 degradation induced by 225-5. These results confirmed that 225-5 mediated RIPK1 degradation requires the engagement of RIPK1, VHL, the proteasome, and the Cullin-RING E3 ligase complex.

### Compound 225-5 Augments Radiation Response in Human and Murine Melanoma Models

To extend our in vitro findings to in vivo studies, we first conducted pharmacokinetic (PK) analysis by intraperitoneally (IP) administering 5 mg/kg of 204-2 or 225-5 in C57BL/6 mice. The concentration in plasma (pooled from 3 mice) of each compound was quantitatively analyzed at seven time points by LC/MS/MS. The following pharmacokinetic parameters were obtained: for 204-2, *C*_max_ = 1.2 μg/mL (1.1 μM), *T*_1/2_ = 3.9 h, and AUC = 4.1 μg/mL·h (3.7 μM·h) (); for 225-5, *C*_max_ = 3.3 μg/mL (2.6 μM), *T*_1/2_ = 5.4 h, and AUC = 4.3 μg/mL·h (3.4 μM·h) (). Although the two compounds exhibit comparable pharmacological profiles, compound 225-5 demonstrates greater potency in most cellular studies and was therefore selected for further investigation.

We then investigated the pharmacodynamics of 225–5 in vivo. We administered 225-5 via both IP and IV route, we observed almost completely reduction in RIPK1 levels in both A375 and B16F10 tumors (20 mg/kg, IV) or 50 mg/kg, IP) (Figure 6A/B).

**Figure 6:**
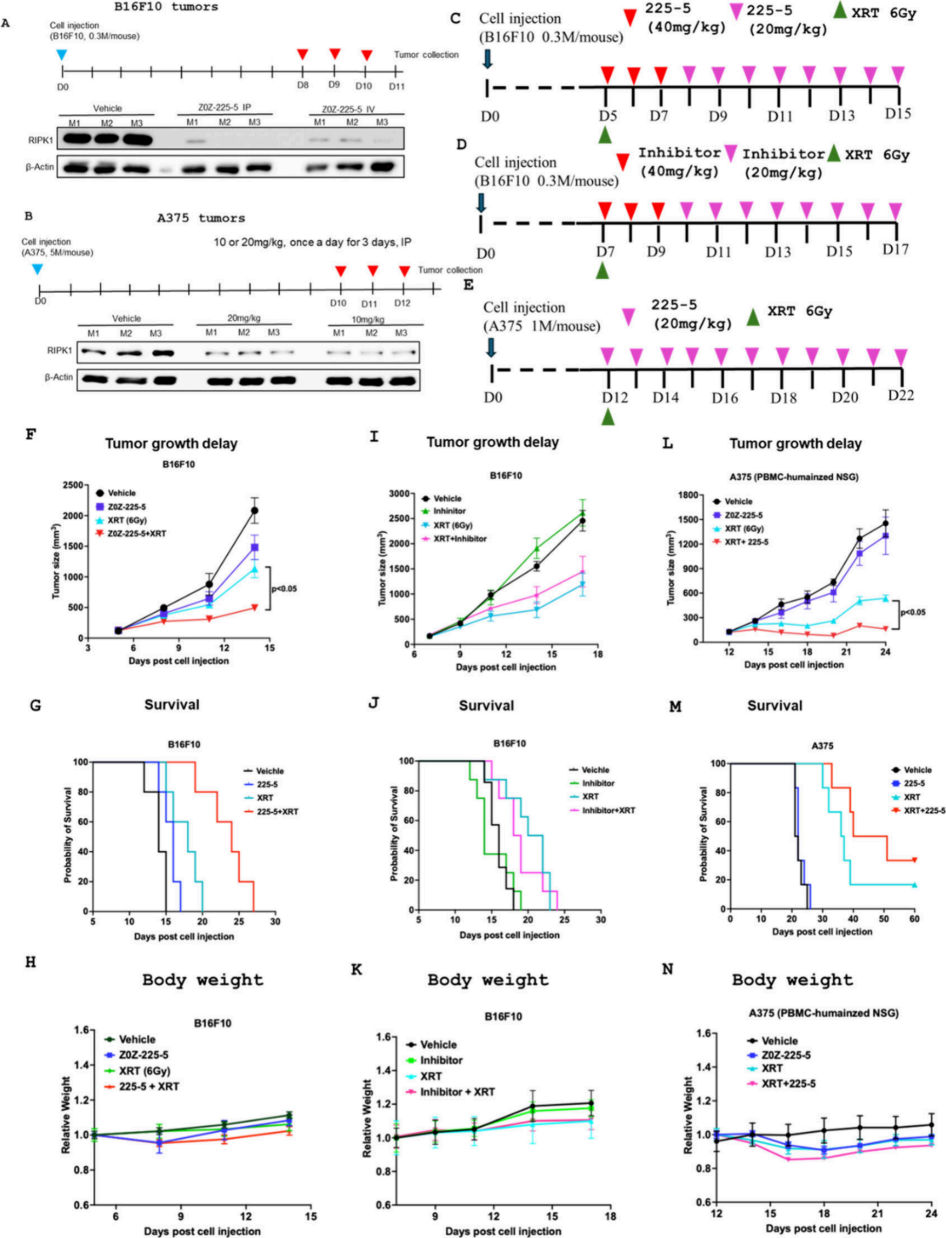
In vivo activity of 225-5 in syngeneic B16F10 and humanized A375 melanoma mouse models. (A,B) In vivo degradation of RIPK1 induced by 225-5 in B16F10 syngeneic mouse model (IP: 50 mg/kg twice daily for 3 days; IV 10 mg/kg twice daily for 3 days) and A375 xenograft mouse mode (IP: 10 or 20 mg/kg, once daily for 3 days), *N* = 3. C) Schematic illustration of treatments in B16F10 syngeneic mouse model (225-5:40 mg/kg once daily for 3 days followed by 20 mg/kg once daily for 8 days; XRT: 6Gy, single fraction) *N* = 5. D) Schematic illustration of treatments in B16F10 syngeneic mouse model (RIPK1 inhibitor: 40 mg/kg once daily for 3 days followed by 20 mg/kg once daily for 8 days; XRT: 6Gy, single fraction) *N* = 5. E) Schematic illustration of treatments in A375 humanized mouse model (225–5:20 mg/kg once daily for 11 days; XRT: 6Gy, single fraction), *N* = 5. F), (G,H) Tumor growth delay, Kaplan–Meier survival and body weight curve in B16F10 syngeneic mouse model (225-5:40 mg/kg once daily for 3 days followed by 20 mg/kg once daily for 8 days; XRT: 6Gy, single fraction) *N* = 5. I), (J,K) Tumor growth delay, Kaplan–Meier survival and body weight curve in B16F10 syngeneic mouse model (RIPK1 inhibitor: 40 mg/kg once daily for 3 days followed by 20 mg/kg once daily for 8 days; XRT: 6Gy, single fraction). (L,M,N) Tumor growth delay, Kaplan–Meier survival and body weight curve in A375 humanized mouse model (225-5:20 mg/kg once daily for 11 days; XRT: 6Gy, single fraction), *N* = 5.

Based on these in vivo degradation results, we evaluated the antitumor efficacy of compound 225-5 in the B16F10 syngeneic mouse model and the A375 humanized mouse model. Mice bearing B16F10 or A375 tumors were randomized into four treatment groups: vehicle control, 225-5, XRT (6 Gy), and 225-5 combined with XRT (n = 5 per group). In the B16F10 model, we employed a front-loaded dosing regimen (40 mg/kg once daily for 3 days, followed by 20 mg/kg once daily for 8 days) to minimize potential side effects (Figure 6C). In contrast, the A375 model was treated with a consistent 20 mg/kg daily dose throughout the study because of the high potency of compound 225-5 in human cancer cells as discussed before (Figure 6E). Body weights remained stable across all treatment groups (Figure 6H/K/N).

In the B16F10 model, the combination of 225-5 with radiation significantly delayed tumor growth and extended median survival from 19 to 24 days, compared to radiation alone (Figure 6F/G). Notably, the RIPK1 inhibitor GSK’074 failed to sensitize B16F10 tumors to radiation when following the schematic illustration of treatments as shown in Figure 6D (40 mg/kg once daily for 3 days followed by 20 mg/kg once daily for 8 days; XRT: 6Gy, single fraction), confirming that the observed synergy is independent of RIPK1 kinase activity (Figure 6I/J).

In the A375 humanized mouse model, human PBMCs were injected into NSG mice 1 day prior to A375 inoculation, allowing rapid engraftment of mature human T cells. The combination of 225-5 and radiation reduced tumor volume by 89% compared to control, 85.4% compared to 225-5 alone, and 70.2% compared to XRT alone (*p* < 0.05 for all comparisons). Median survival was extended from 36 to 51 days (Figure 6L/M).

Collectively, these results demonstrate that 225-5 significantly enhances tumor regression when combined with radiation therapy, suggesting its potential to sensitize tumors to XRT. This combination represents a promising therapeutic strategy for improving radiotherapy outcomes in cancer. The detailed mechanisms by which RIPK1 degradation enhances radiotherapy are under active investigation and will be reported in future studies.

## Synthesis of RIPK1 PROTACs

The synthesis of RIPK1-targeting PROTACs is summarized in Scheme 1 and Scheme 2. The RIPK1 binder (S3) and linkers were prepared according to published procedures. For compounds with flexible linkers, the commercially available VHL ligand (S1) was first coupled to the linker to form the amide intermediate S2. After Boc-deprotection, the resulting free amine was reacted with RIPK1 binder S3 to yield the final PROTAC.

**Scheme 1:**
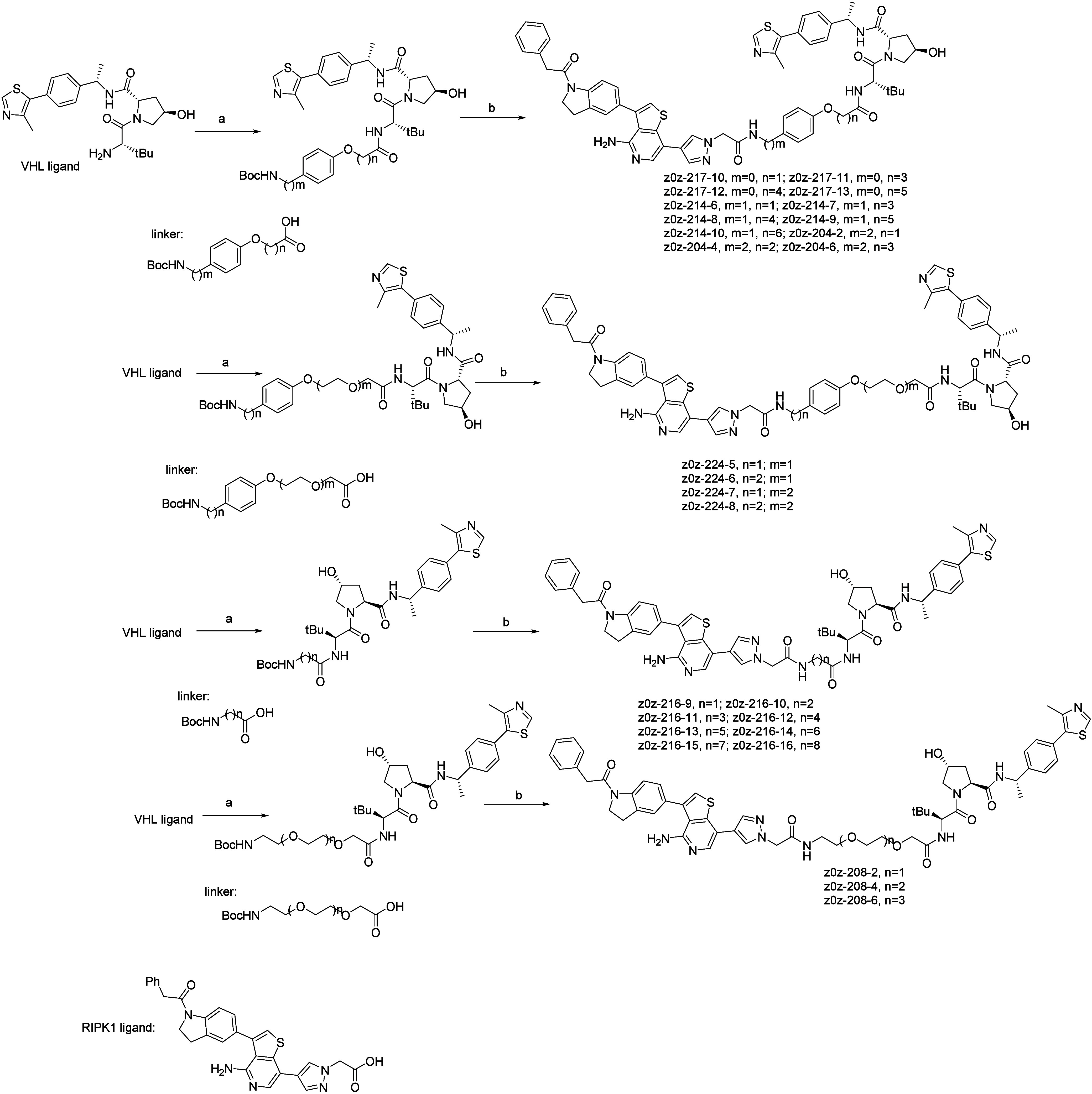
Synthesis of Compounds 204-2,4,6; 208-2,4,6; 214-6,7,8,9,10; 216-9,10,11,12,13,14,15,16; 217-10,11,12,13; 224-5,6,7,8^a^

a Reagent and conditions: (a) linker, HATU, DIPEA, DMF, rt; (b) TFA, DCM, rt; then RIPK1 ligand, HATU, DIPEA, DMF, rt.

**Scheme 2:**
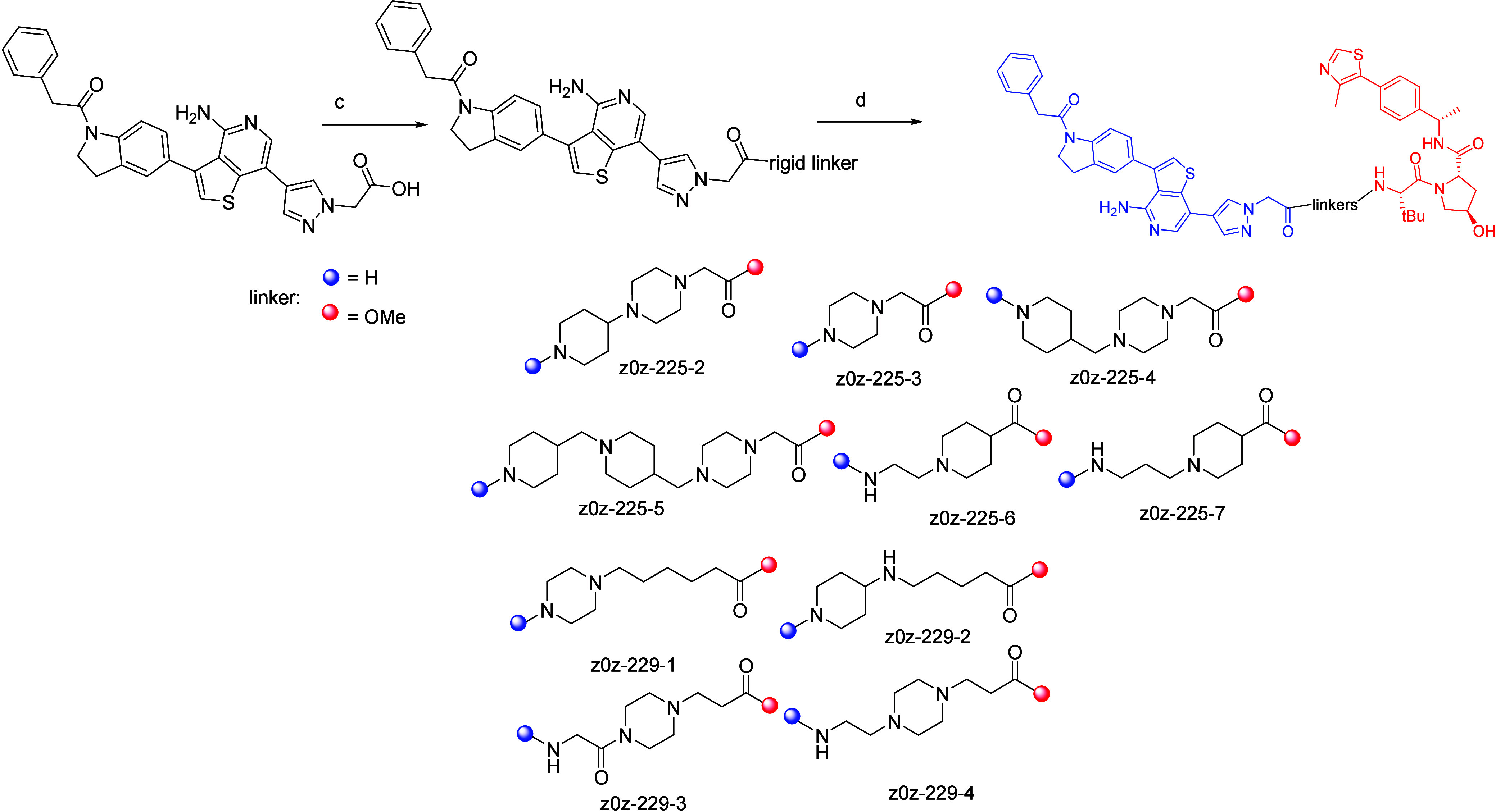
Synthesis of Compounds 225-2,3,4,5,6,7; 229-1,2,3,4^a^

a Reagent and conditions: (c) linker, HATU, DIPEA, DMF, rt; (d) LiOH, MeOH/THF/H_2_O (V/V/V = 3:3:1), rt, then VHL ligand, PyAOP, DIPEA, DMF, rt.

For compounds containing rigid linker, RIPK1 binder S3 was first coupled to the linker to form amide intermediate S4. Subsequent hydrolysis yielded the free acid, which was then coupled to VHL ligand S1 to generate the final PROTAC.

## Conclusions

In summary, we developed a series of potent and selective degraders targeting RIPK1 and rapidly identified suitable E3 ligases and linker lengths using our Rapid-TAC platform. Further optimization of linker rigidity through incorporation of piperidine and piperazine rings led to the development of compound 225-5, which exhibited superior pharmacokinetics compared to the flexible-linker analog 204-2. Compound 225-5 achieved a DC_50_ of ∼41 nM and *D*_max_ of 97% in human A375 melanoma cells.

Crucially, degrader 225-5 enhanced the therapeutic efficacy of radiotherapy in both syngeneic and humanized mouse models without inducing observable toxicity, as evidenced by stable body weight. In contrast, the corresponding RIPK1 inhibitor showed no meaningful therapeutic benefit, underscoring the unique advantage of targeted protein degradation over enzymatic inhibition.

To our knowledge, this study represents the first demonstration of in vivo antitumor efficacy of a RIPK1-targeting PROTAC in combination with XRT in a human cancer model. These findings highlight the potential of RIPK1 degradation, specifically targeting its nonenzymatic scaffolding function, as a novel and promising strategy to enhance the effectiveness of cancer radiotherapy.

## Experimental Section

### Chemistry

All reactions were conducted under a positive pressure of dry argon in glassware that had been oven-dried prior to use. Anhydrous solutions of reaction mixtures were transferred via an oven-dried syringe or cannula. All solvents were dried prior to use unless noted. Thin-layer chromatography (TLC) was performed using precoated silica gel plates. Flash column chromatography was performed with silica gel. ^1^H and ^13^C nuclear magnetic resonance (NMR) spectra were recorded on Bruker 400 MHz. 1H NMR spectra were reported in parts per million (ppm) referenced to 7.26 ppm of CDCl_3_ or referenced to the centerline of a septet at 2.50 ppm of DMSO-*d*_6_. Signal splitting patterns were described as singlet (s), doublet (d), triplet (t), quartet (q), quintet (quint), or multiplet (m), with coupling constants (*J*) in hertz. High-resolution mass spectra (HRMS) were acquired on an electrospray ionization (ESI) TOF mass spectrometer.

The HPLC spectrometry analysis of the final products was processed on an Schimadzu CMB-40 system using a Schimadzu Nexcol C18 column (5 cm × 3.0 mm, 5 μm) for chromatographic separation. Schimadzu SPD-40 LC/MS with multimode electrospray ionization plus atmospheric pressure chemical ionization was used for detection. Short-time method: The mobile phases were 0.1% formic acid in purified water (A) and 0.1% formic acid in MeCN (B). The gradient was increased from 5% to 100% at 10 min, then held at isocratic 100% B for 5 min, and then immediately stepped back down to 5% for 5 min re-equilibration. The flow rate was set at 1.0 mL/min. The column temperature was set at 30 °C. Long-time method: The mobile phases were 0.1% formic acid in purified water (A) and 0.1% formic acid in MeCN (B). The gradient was increased from 5% to 100% at 20 min, then held at isocratic 100% B for 5 min, and then immediately stepped back down to 5% for 5 min re-equilibration. The flow rate was set at 0.8 mL/min. The column temperature was set at 30 °C. The purities of all of the final compounds were determined to be over 95% by LC–MS.

### General Procedure a

(Condensation of Boc-protected amino acid with VHL ligand): To Boc-protected amino acid (1.0 equiv) and commercially available VHL ligand (1.05 equiv) in DMF, were added HATU (1.2 equiv) and DIPEA (3.0 equiv) successively. The solution was stirred at room temperature for 2h then added with EtOAc and water. The water phase was extracted with EtOAc (3×) and the combined organic phase was washed with water, brine, dried over sodium sulfate, filtered and condensed to afford a residue which was purified by flash column chromatography on silica to afford the desired compound.

### General Procedure b

(Boc deprotection and condensation with RIPK1 binder): To a stirring solution of Boc protected intermediate (1 equiv) in DCM was added TFA (5.0 equiv). After stirring at room temperature for 1 h, the mixture was condensed under reduced pressure to afford TFA salt quantitatively. The solid was dissolved in DMF, DIPEA (1.5 equiv) was added to neutralize the TFA, followed by the addition of a mixed solution of RIPK1 binder (1 equiv), HATU (1.2 equiv), and DIPEA (2 equiv) in DMF dropwise at 0 °C. The solution was stirred at room temperature for 2 h then added with EtOAc and water. The water phase was extracted with EtOAc (3×) and the combined organic phase was washed with water, brine, dried over sodium sulfate, filtered, and condensed to afford a residue which was purified by reverse-ISCO to yield final compound.

### General Procedure c

(condensation of ester protected amino acid with RIPK1 binder): To a stirring solution of ester protected amino acid (1.05 equiv) RIPK1 binder (1 equiv) in DMF, were added HATU (1.2 equiv) and DIPEA (3.0 equiv) successively. The solution was stirred at room temperature for 2 h then added with EtOAc and water. The water phase was extracted with EtOAc (3×) and the combined organic phase was washed with water, brine, dried over sodium sulfate, filtered and condensed to afford a residue which was purified by flash column chromatography on silica to afford the desired compound.

### General Procedure d

(Ester deprotection and condensation with VHL ligand): To a stirring solution of ester protected intermediate (1 equiv) in MeOH/THF/H_2_O (V/V/V = 3:3:1) was added LiOH (5.0 equiv). After stirring at room temperature for 2 h, the mixture was condensed under reduced pressure and add 2 N HCl to PH = 4 to afford free acid which can be filted and fully dried. The solid was dissolved in DMF, VHL ligand (1 equiv), PyAOP (1.2 equiv) and DIPEA (3 equiv) were added. The solution was stirred at room temperature for 2 h then added with EtOAc and water. The water phase was extracted with EtOAc (3×) and the combined organic phase was washed with water, brine, dried over sodium sulfate, filtered, and condensed to afford a residue which was purified by reverse-ISCO to yield final compound.

#### 1-(5-(4-Amino-7-(1-methyl-1*H*-pyrazol-4-yl)­thieno­[3,2-*c*]­pyridin-3-yl)­indolin-1-yl)-2-phenylethan-1-one (**z0z**-**243**-**1**)

Prepared according to the literature procedure^27^ ^1^H NMR (400 MHz, DMSO-*d*_6_) δ 8.20–8.12 (m, 3H), 8.03 (s, 1H), 7.88 (s, 1H), 7.49 (s, 1H), 7.38–7.23 (m, 8H), 5.41 (s, 2H), 4.26 (t, *J* = 8.5 Hz, 2H), 3.94 (s, 3H), 3.89 (s, 2H), 3.23 (d, *J* = 8.6 Hz, 2H).

#### 2-(4-(4-Amino-3-(1-(2-phenylacetyl)­indolin-5-yl)­thieno­[3,2-*c*]­pyridin-7-yl)-1*H*-pyrazol-1-yl)­acetic Acid (**z0z**-**203**-**3**)

Prepared according to the literature procedure^27^ ^1^H NMR (400 MHz, DMSO-*d*_6_) δ 13.11 (s, 1H), 8.18 (d, *J* = 14.2 Hz, 2H), 8.07 (s, 1H), 7.94 (s, 1H), 7.51 (s, 1H), 7.33 (dd, *J* = 11.3, 7.1 Hz, 5H), 7.27 (t, *J* = 7.3 Hz, 2H), 5.46 (s, 2H), 5.05 (s, 2H), 4.25 (t, *J* = 8.4 Hz, 2H), 3.89 (s, 2H), 3.26–3.20 (m, 3H).

#### (2*S*,4*R*)-1-((*S*)-2-(2-(4-(2-(2-(4-(4-Amino-3-(1-(2-phenylacetyl)­indolin-5-yl)­thieno­[3,2-c]­pyridin-7-yl)-1*H*-pyrazol-1-yl)­acetamido)­ethyl)­phenoxy)­acetamido)-3,3-dimethylbutanoyl)-4-hydroxy-*N*-((*S*)-1-(4-(4-methylthiazol-5-yl)­phenyl)­ethyl)­pyrrolidine-2-carboxamide (**z0z-204–2**)

Linker part was prepared according to the literature procedure^28^ ^1^H NMR (400 MHz, Chloroform-*d*) δ 8.66 (s, 1H), 8.36 (d, *J* = 8.2 Hz, 1H), 7.94 (d, *J* = 9.5 Hz, 2H), 7.81 (s, 1H), 7.60 (d, *J* = 7.8 Hz, 1H), 7.42–7.26 (m, 12H), 7.15 (s, 1H), 6.96 (d, *J* = 8.1 Hz, 2H), 6.68 (d, *J* = 8.1 Hz, 2H), 6.32 (d, *J* = 6.2 Hz, 1H), 5.08 (t, *J* = 7.2 Hz, 1H), 4.98 (s, 2H), 4.85 (s, 2H), 4.75 (t, *J* = 7.9 Hz, 1H), 4.67 (d, *J* = 9.0 Hz, 1H), 4.50 (s, 1H), 4.30 (d, *J* = 14.7 Hz, 1H), 4.22–4.10 (m, 3H), 4.01 (d, *J* = 11.1 Hz, 1H), 3.84 (s, 2H), 3.66 (dd, *J* = 11.5, 3.9 Hz, 1H), 3.48 (p, *J* = 7.0 Hz, 2H), 3.23 (t, *J* = 8.6 Hz, 2H), 2.70 (t, *J* = 6.8 Hz, 2H), 2.50 (s, 3H), 2.40 (dd, *J* = 10.4, 5.6 Hz, 1H), 2.14–2.01 (m, 1H), 1.47 (d, *J* = 6.9 Hz, 3H), 1.03 (s, 9H). ^13^C NMR (101 MHz, chloroform-*d*) δ 171.1, 170.0, 169.5, 168.2, 167.0, 155.7, 153.3, 150.3, 148.4, 147.5, 143.4, 143.3, 139.2, 138.9, 138.0, 133.9, 131.8, 131.6, 131.2, 130.7, 129.9, 129.5, 129.1, 128.8, 128.3, 127.1, 126.4, 125.7, 122.6, 119.6, 119.5, 117.0, 114.8, 113.7, 70.0, 67.0, 58.7, 57.0, 56.9, 55.3, 48.8, 48.4, 43.5, 40.5, 36.2, 35.7, 34.3, 28.0, 26.4, 22.2, 16.1. HRMS-ESI (*m*/*z*): [M + H^+^]^+^ calculated for C_61_H_64_N_10_O_7_S_2_, 1113.4474; found, 1113.4449. HPLC retention time: 10.4 min, purity >95%.

#### (2*S*,4*R*)-1-((*S*)-2-(3-(4-(2-(2-(4-(4-Amino-3-(1-(2-phenylacetyl)­indolin-5-yl)­thieno­[3,2-*c*]­pyridin-7-yl)-1*H*-pyrazol-1-yl)­acetamido)­ethyl)­phenoxy)­propanamido)-3,3-dimethylbutanoyl)-4-hydroxy-*N*-((*S*)-1-(4-(4-methylthiazol-5-yl)­phenyl)­ethyl)­pyrrolidine-2-carboxamide (**z0z-204-4**)

Linker part was prepared according to the literature procedure^28^ ^1^H NMR (400 MHz, chloroform-*d*) δ 8.66 (s, 1H), 8.35 (d, *J* = 8.2 Hz, 1H), 7.99 (s, 1H), 7.86 (s, 1H), 7.74 (s, 1H), 7.43–7.24 (m, 15H), 7.14 (s, 1H), 6.91 (d, *J* = 8.2 Hz, 2H), 6.66 (d, *J* = 8.1 Hz, 2H), 6.11 (s, 1H), 5.17 (s, 1H), 5.06 (t, *J* = 7.2 Hz, 1H), 4.82 (s, 2H), 4.73 (t, *J* = 8.0 Hz, 1H), 4.66 (d, *J* = 8.7 Hz, 1H), 4.48 (s, 1H), 4.21–4.02 (m, 5H), 4.00–3.92 (m, 1H), 3.82 (s, 2H), 3.64–3.58 (m, 1H), 3.53–3.44 (m, 2H), 3.22 (s, 2H), 2.65 (dt, *J* = 29.8, 7.1 Hz, 4H), 2.50 (s, 7H), 2.07 (t, *J* = 10.9 Hz, 1H), 1.43 (d, *J* = 6.9 Hz, 3H), 1.06 (s, 10H). ^13^C NMR (101 MHz, chloroform-*d*) δ 172.0, 171.3, 169.9, 169.5, 167.0, 156.9, 153.4, 150.3, 148.5, 147.9, 143.5, 143.2, 139.1, 133.9, 132.0, 131.6, 131.2, 130.8, 130.7, 129.8, 129.5, 129.1, 128.8, 128.8, 128.3, 127.2, 126.5, 125.7, 122.8, 119.5, 117.1, 114.7, 70.0, 64.1, 58.6, 57.9, 57.0, 55.3, 48.8, 48.4, 43.5, 40.4, 36.5, 35.8, 35.3, 34.0, 28.0, 26.6, 22.2, 16.1. HRMS-ESI (*m*/*z*): [M + H^+^]^+^ calculated for C_62_H_66_N_10_O_7_S_2_, 1127.4630; found, 1127.4585. HPLC retention time: 10.3 min, purity >95%.

#### (2*S*,4*R*)-1-((*S*)-2-(4-(4-(2-(2-(4-(4-Amino-3-(1-(2-phenylacetyl)­indolin-5-yl)­thieno­[3,2-*c*]­pyridin-7-yl)-1*H*-pyrazol-1-yl)­acetamido)­ethyl)­phenoxy)­butanamido)-3,3-dimethylbutanoyl)-4-hydroxy-*N*-((*S*)-1-(4-(4-methylthiazol-5-yl)­phenyl)­ethyl)­pyrrolidine-2-carboxamide (**z0z-204-6**)

Linker part was prepared according to the literature procedure^28^ ^1^H NMR (400 MHz, chloroform-*d*) δ 8.65 (s, 1H), 8.34 (d, *J* = 8.2 Hz, 1H), 7.99 (s, 1H), 7.86 (s, 1H), 7.80 (s, 1H), 7.48–7.23 (m, 16H), 7.14 (s, 1H), 6.87 (d, *J* = 8.1 Hz, 2H), 6.59 (d, *J* = 8.2 Hz, 2H), 6.17 (t, *J* = 5.7 Hz, 1H), 5.24 (s, 2H), 5.06 (t, *J* = 7.2 Hz, 1H), 4.83 (s, 2H), 4.73–4.63 (m, 2H), 4.47 (s, 1H), 4.11 (q, *J* = 9.7, 9.1 Hz, 3H), 3.81 (s, 2H), 3.75–3.70 (m, 2H), 3.67–3.58 (m, 2H), 3.51 (dt, *J* = 12.4, 6.4 Hz, 1H), 3.41 (dd, *J* = 13.1, 6.7 Hz, 1H), 3.21 (t, *J* = 8.2 Hz, 2H), 2.66 (q, *J* = 6.0 Hz, 2H), 2.49 (s, 4H), 2.36 (td, *J* = 16.1, 15.3, 7.5 Hz, 3H), 2.07 (dd, *J* = 13.4, 8.3 Hz, 1H), 1.97 (q, *J* = 7.7 Hz, 2H), 1.40 (dd, *J* = 13.7, 6.9 Hz, 6H), 1.04 (s, 11H). ^13^C NMR (101 MHz, chloroform-*d*) δ 173.3, 172.1, 170.0, 169.5, 167.0, 157.4, 153.4, 150.3, 148.4, 147.6, 143.4, 143.2, 138.9, 138.8, 138.0, 133.9, 131.9, 131.5, 131.2, 130.8, 130.1, 129.6, 129.5, 129.1, 128.8, 128.7, 128.2, 127.1, 126.4, 125.7, 122.6, 119.5, 119.4, 117.0, 114.6, 113.7, 69.8, 66.9, 58.7, 57.8, 56.9, 55.3, 55.1, 48.7, 48.4, 43.4, 40.3, 35.9, 35.2, 34.1, 32.5, 28.0, 26.6, 25.1, 22.1, 16.1. HRMS-ESI (*m*/*z*): [M + H^+^]^+^ calculated for C_63_H_68_N_10_O_7_S_2_, 1141.4787; found, 1141.4747. HPLC retention time: 10.4 min, purity >95%.

#### (2*S*,4*R*)-1-((*S*)-14-(4-(4-Amino-3-(1-(2-phenylacetyl)­indolin-5-yl)­thieno­[3,2-*c*]­pyridin-7-yl)-1*H*-pyrazol-1-yl)-2-(*tert*-butyl)-4,13-dioxo-6,9-dioxa-3,12-diazatetradecanoyl)-4-hydroxy-*N*-((*S*)-1-(4-(4-methylthiazol-5-yl)­phenyl)­ethyl)­pyrrolidine-2-carboxamide (**z0z-208-2**)

^1^H NMR (400 MHz, chloroform-*d*) δ 8.62 (s, 1H), 8.33 (d, *J* = 8.2 Hz, 1H), 8.17 (d, *J* = 7.6 Hz, 2H), 8.04 (s, 1H), 7.91 (s, 1H), 7.62 (d, *J* = 8.9 Hz, 2H), 7.40–7.21 (m, 14H), 7.06 (s, 1H), 5.16 (t, *J* = 10.9 Hz, 2H), 4.81 (dd, *J* = 32.7, 15.3 Hz, 5H), 4.54 (s, 1H), 4.16 (t, *J* = 8.4 Hz, 2H), 3.92 (dd, *J* = 35.7, 16.3 Hz, 6H), 3.81–3.49 (m, 12H), 3.31–3.17 (m, 4H), 2.45 (s, 4H), 2.26 (t, *J* = 10.4 Hz, 2H), 1.39 (dd, *J* = 14.0, 6.8 Hz, 7H), 1.08 (s, 11H). ^13^C NMR (101 MHz, chloroform-*d*) δ 170.7, 170.7, 170.0, 169.5, 167.0, 152.8, 150.2, 148.3, 147.7, 143.4, 143.0, 137.8, 137.7, 133.9, 131.9, 131.6, 131.1, 130.6, 129.3, 129.1, 128.9, 128.8, 128.7, 127.1, 126.7, 125.7, 122.8, 119.4, 119.0, 117.0, 114.2, 71.3, 70.1, 69.9, 69.8, 59.5, 57.3, 56.8, 55.2, 54.8, 48.5, 48.4, 43.5, 43.2, 39.2, 37.3, 36.8, 28.0, 26.6, 21.1, 16.1, 12.5. HRMS-ESI (*m*/*z*): [M + H^+^]^+^ calculated for C_57_H_64_N_10_O_8_S_2_, 1081.4423; found, 1081.4407. HPLC retention time: 9.8 min, purity >95%.

#### (2*S*,4*R*)-1-((*S*)-17-(4-(4-Amino-3-(1-(2-phenylacetyl)­indolin-5-yl)­thieno­[3,2-*c*]­pyridin-7-yl)-1*H*-pyrazol-1-yl)-2-(*tert*-butyl)-4,16-dioxo-6,9,12-trioxa-3,15-diazaheptadecanoyl)-4-hydroxy-*N*-((*S*)-1-(4-(4-methylthiazol-5-yl)­phenyl)­ethyl)­pyrrolidine-2-carboxamide (**z0z-208-4**)

^1^H NMR (400 MHz, chloroform-*d*) δ 8.63 (s, 1H), 8.31 (d, *J* = 8.1 Hz, 1H), 7.95 (s, 2H), 7.87 (s, 1H), 7.65 (d, *J* = 7.7 Hz, 1H), 7.48 (s, 1H), 7.27 (d, *J* = 50.0 Hz, 12H), 7.07 (s, 1H), 5.06 (t, *J* = 7.3 Hz, 1H), 4.97 (d, *J* = 16.4 Hz, 1H), 4.91–4.76 (m, 3H), 4.72 (t, *J* = 8.1 Hz, 1H), 4.60 (d, *J* = 8.7 Hz, 1H), 4.49 (s, 1H), 4.14 (t, *J* = 8.4 Hz, 2H), 4.07–3.93 (m, 3H), 3.83 (s, 2H), 3.71–3.35 (m, 14H), 3.21 (d, *J* = 8.7 Hz, 2H), 2.47 (s, 3H), 2.30 (s, 1H), 2.12 (d, *J* = 13.5 Hz, 1H), 1.44 (d, *J* = 6.9 Hz, 3H), 1.04 (s, 9H). ^13^C NMR (101 MHz, chloroform-*d*) δ 171.2, 170.6, 170.3, 169.5, 167.8, 153.0, 150.3, 148.4, 147.6, 143.4, 138.2, 137.8, 134.0, 132.0, 131.6, 131.2, 130.7, 129.4, 129.1, 128.9, 128.8, 128.7, 127.1, 126.4, 125.7, 122.7, 119.4, 119.2, 117.0, 114.0, 70.4, 70.3, 70.2, 70.0, 69.9, 58.9, 57.3, 57.1, 54.8, 48.8, 48.4, 43.5, 39.6, 36.5, 35.4, 28.0, 26.5, 22.0, 16.1. HRMS-ESI (*m*/*z*): [M + ^+^]^+^ calculated for C_59_H_68_N_10_O_9_S_2_, 1125.4685; found, 1125.4673. HPLC retention time: 9.7 min, purity >95%.

#### (2*S*,4*R*)-1-((*S*)-20-(4-(4-Amino-3-(1-(2-phenylacetyl)­indolin-5-yl)­thieno­[3,2-*c*]­pyridin-7-yl)-1*H*-pyrazol-1-yl)-2-(*tert*-butyl)-4,19-dioxo-6,9,12,15-tetraoxa-3,18-diazaicosanoyl)-4-hydroxy-*N*-((*S*)-1-(4-(4-methylthiazol-5-yl)­phenyl)­ethyl)­pyrrolidine-2-carboxamide (**z0z-208-6**)

^1^H NMR (400 MHz, chloroform-*d*) δ 8.65 (s, 1H), 8.34 (d, *J* = 8.2 Hz, 1H), 8.00 (s, 1H), 7.94 (d, *J* = 3.1 Hz, 2H), 7.61 (d, *J* = 7.7 Hz, 1H), 7.39–7.16 (m, 14H), 7.11 (s, 1H), 5.08 (p, *J* = 7.3 Hz, 1H), 4.96–4.90 (m, 2H), 4.85 (s, 2H), 4.73 (t, *J* = 7.9 Hz, 1H), 4.62 (d, *J* = 8.9 Hz, 1H), 4.49 (s, 1H), 4.15 (t, *J* = 8.5 Hz, 2H), 4.07–3.94 (m, 4H), 3.84 (s, 2H), 3.73–3.41 (m, 20H), 3.22 (t, *J* = 8.6 Hz, 2H), 2.50 (s, 3H), 2.38 (q, *J* = 6.6, 5.2 Hz, 1H), 2.16–2.05 (m, 1H), 1.46 (d, *J* = 6.9 Hz, 3H), 1.05 (s, 10H). ^13^C NMR (101 MHz, chloroform-*d*) δ 171.1, 170.1, 170.1, 169.4, 167.2, 153.0, 150.2, 148.3, 147.6, 143.3, 143.3, 139.3, 138.4, 137.8, 133.9, 131.8, 131.6, 131.3, 130.6, 129.4, 129.0, 128.7, 128.6, 128.6, 127.1, 126.4, 125.6, 122.5, 119.3, 119.3, 116.9, 114.0, 70.8, 70.3, 70.3, 70.3, 70.2, 70.2, 69.8, 69.6, 58.7, 56.9, 56.9, 55.0, 48.7, 48.3, 43.4, 39.4, 36.1, 35.5, 27.9, 26.5, 22.1, 16.0. HRMS-ESI (*m*/*z*): [M + Na^+^]^+^ calculated for C_61_H_72_N_10_O_10_S_2_Na, 1191.4767; found, 1191.4766. HPLC retention time: 9.6 min, purity >95%.

#### (2*S*,4*R*)-1-((*S*)-2-(2-(4-((2-(4-(4-Amino-3-(1-(2-phenylacetyl)­indolin-5-yl)­thieno­[3,2-*c*]­pyridin-7-yl)-1*H*-pyrazol-1-yl)­acetamido)­methyl)­phenoxy)­acetamido)-3,3-dimethylbutanoyl)-4-hydroxy-*N*-((*S*)-1-(4-(4-methylthiazol-5-yl)­phenyl)­ethyl)­pyrrolidine-2-carboxamide (**z0z-214-6**)

Linker part was prepared according to the literature procedure^28^ ^1^H NMR (400 MHz, chloroform-*d*) δ 8.64 (s, 1H), 8.32 (d, *J* = 8.2 Hz, 1H), 7.90 (d, *J* = 5.7 Hz, 3H), 7.66 (d, *J* = 7.6 Hz, 1H), 7.37–7.17 (m, 15H), 7.11 (d, *J* = 7.9 Hz, 3H), 6.73 (d, *J* = 8.2 Hz, 2H), 5.06 (t, *J* = 7.2 Hz, 1H), 4.94 (d, *J* = 9.9 Hz, 4H), 4.73–4.60 (m, 2H), 4.48 (s, 1H), 4.34 (d, *J* = 6.6 Hz, 3H), 4.24 (d, *J* = 15.0 Hz, 1H), 4.13 (t, *J* = 8.4 Hz, 3H), 3.96 (d, *J* = 11.2 Hz, 1H), 3.83 (s, 2H), 3.69–3.57 (m, 2H), 3.20 (t, *J* = 8.4 Hz, 3H), 3.06 (q, *J* = 7.4 Hz, 1H), 2.47 (s, 3H), 2.30 (s, 1H), 2.10 (d, *J* = 11.2 Hz, 1H), 1.45 (t, *J* = 7.9 Hz, 8H), 1.00 (s, 10H). ^13^C NMR (101 MHz, chloroform-*d*) δ 170.7, 170.1, 169.5, 168.3, 167.0, 156.3, 153.0, 150.3, 148.3, 147.7, 143.4, 143.3, 138.8, 138.7, 137.8, 133.9, 131.9, 131.6, 131.4, 131.1, 130.6, 129.4, 129.0, 129.0, 128.8, 128.7, 127.1, 126.3, 125.6, 122.7, 119.4, 119.3, 117.0, 114.8, 113.9, 69.9, 67.0, 58.8, 56.9, 55.1, 53.6, 48.8, 48.4, 43.4, 42.7, 41.9, 36.4, 35.7, 27.9, 26.4, 22.2, 16.0, 12.0. HRMS-ESI (*m*/*z*): [M + H^+^]^+^ calculated for C_60_H_62_N_10_O_7_S_2_, 1099.4317; found, 1099.4317. HPLC retention time: 9.2 min, purity >95%.

#### (2*S*,4*R*)-1-((*S*)-2-(4-(4-((2-(4-(4-Amino-3-(1-(2-phenylacetyl)­indolin-5-yl)­thieno­[3,2-*c*]­pyridin-7-yl)-1*H*-pyrazol-1-yl)­acetamido)­methyl)­phenoxy)­butanamido)-3,3-dimethylbutanoyl)-4-hydroxy-*N*-((*S*)-1-(4-(4-methylthiazol-5-yl)­phenyl)­ethyl)­pyrrolidine-2-carboxamide (**z0z-214-7**)

Linker part was prepared according to the literature procedure^28^ ^1^H NMR (400 MHz, chloroform-*d*) δ 8.62 (d, *J* = 3.8 Hz, 1H), 8.29 (t, *J* = 5.8 Hz, 1H), 7.87 (d, *J* = 20.4 Hz, 3H), 7.65 (d, *J* = 40.5 Hz, 2H), 7.38–7.20 (m, 10H), 7.17 (s, 2H), 7.05 (d, *J* = 6.4 Hz, 4H), 6.72–6.61 (m, 2H), 5.20–5.01 (m, 3H), 4.89 (s, 2H), 4.65 (s, 2H), 4.46 (s, 1H), 4.29 (s, 2H), 4.03 (d, *J* = 38.1 Hz, 3H), 3.78 (s, 5H), 3.63 (s, 2H), 3.14 (s, 2H), 2.45 (d, *J* = 4.4 Hz, 3H), 2.39–2.18 (m, 3H), 2.01 (d, *J* = 57.0 Hz, 3H), 1.48–1.32 (m, 5H), 1.02 (s, 10H). ^13^C NMR (101 MHz, chloroform-*d*) δ 172.7, 171.2, 170.1, 169.3, 166.8, 157.8, 152.8, 150.2, 148.0, 147.9, 147.4, 143.2, 143.0, 138.3, 138.0, 137.5, 133.7, 131.7, 131.3, 130.9, 130.3, 129.6, 129.1, 128.8, 128.6, 128.5, 128.3, 126.8, 126.2, 125.4, 122.5, 119.0, 118.8, 116.6, 114.3, 113.7, 69.5, 66.6, 58.7, 57.4, 56.7, 54.7, 53.5, 48.4, 48.1, 43.1, 42.6, 36.3, 35.2, 32.2, 27.6, 26.3, 24.8, 21.9, 15.8. HRMS-ESI (*m*/*z*): [M + H^+^]^+^ calculated for C_62_H_66_N_10_O_7_S_2_, 1127.4630; found, 1127.4626. HPLC retention time: 9.2 min, purity >95%.

#### (2*S*,4*R*)-1-((*S*)-2-(5-(4-((2-(4-(4-Amino-3-(1-(2-phenylacetyl)­indolin-5-yl)­thieno­[3,2-*c*]­pyridin-7-yl)-1*H*-pyrazol-1-yl)­acetamido)­methyl)­phenoxy)­pentanamido)-3,3-dimethylbutanoyl)-4-hydroxy-*N*-((*S*)-1-(4-(4-methylthiazol-5-yl)­phenyl)­ethyl)­pyrrolidine-2-carboxamide (**z0z-214-8**)

Linker part was prepared according to the literature procedure^28^ ^1^H NMR (400 MHz, chloroform-*d*) δ 8.63 (d, *J* = 4.2 Hz, 1H), 8.29 (d, *J* = 8.5 Hz, 1H), 7.94–7.81 (m, 3H), 7.59 (d, *J* = 51.5 Hz, 2H), 7.38–7.13 (m, 12H), 7.07 (d, *J* = 9.4 Hz, 4H), 6.95 (s, 1H), 6.71 (d, *J* = 8.2 Hz, 3H), 5.15–4.98 (m, 2H), 4.89 (s, 2H), 4.66 (q, *J* = 8.7, 8.2 Hz, 2H), 4.46 (s, 1H), 4.31 (s, 2H), 4.04 (d, *J* = 36.4 Hz, 4H), 3.71 (d, *J* = 62.4 Hz, 6H), 3.15 (s, 2H), 2.46 (d, *J* = 4.9 Hz, 3H), 2.35–1.98 (m, 5H), 1.66 (s, 5H), 1.43 (s, 3H), 1.31–1.19 (m, 2H), 1.01 (d, *J* = 21.9 Hz, 12H). ^13^C NMR (101 MHz, chloroform-*d*) δ 173.3, 171.7, 170.4, 169.6, 167.1, 158.2, 153.1, 150.5, 148.2, 147.7, 143.4, 138.8, 138.4, 137.8, 134.0, 132.0, 131.6, 131.2, 130.6, 129.7, 129.4, 129.1, 128.9, 128.8, 127.1, 126.5, 125.7, 122.8, 119.3, 119.2, 116.9, 114.5, 114.0, 69.8, 67.4, 59.0, 57.6, 57.0, 55.0, 54.6, 48.7, 48.4, 43.4, 42.9, 36.4, 35.7, 35.5, 28.6, 27.9,^26^ 8, 26.6, 22.3, 22.1, 17.9, 16.1. HRMS-ESI (*m*/*z*): [M + H^+^]^+^ calculated for C_63_H_68_N_10_O_7_S_2_, 1141.4787; found, 1141.4785. HPLC retention time: 6.2 min, purity >95%.

#### (2*S*,4*R*)-1-((*S*)-2-(6-(4-((2-(4-(4-Amino-3-(1-(2-phenylacetyl)­indolin-5-yl)­thieno­[3,2-*c*]­pyridin-7-yl)-1*H*-pyrazol-1-yl)­acetamido)­methyl)­phenoxy)­hexanamido)-3,3-dimethylbutanoyl)-4-hydroxy-*N*-((*S*)-1-(4-(4-methylthiazol-5-yl)­phenyl)­ethyl)­pyrrolidine-2-carboxamide (**z0z-214-9**)

Linker part was prepared according to the literature procedure^28^ ^1^H NMR (400 MHz, chloroform-*d*) δ 8.64 (s, 1H), 8.32 (d, *J* = 8.2 Hz, 1H), 7.92 (d, *J* = 18.2 Hz, 3H), 7.54 (d, *J* = 8.0 Hz, 1H), 7.39–7.20 (m, 13H), 7.09 (d, *J* = 7.9 Hz, 4H), 6.74 (d, *J* = 8.2 Hz, 2H), 6.63 (d, *J* = 8.5 Hz, 1H), 5.07 (t, *J* = 7.2 Hz, 1H), 4.99–4.86 (m, 4H), 4.70–4.60 (m, 2H), 4.47 (s, 1H), 4.37–4.29 (m, 2H), 4.12 (t, *J* = 8.4 Hz, 2H), 4.00 (d, *J* = 11.2 Hz, 1H), 3.82 (s, 5H), 3.65–3.57 (m, 1H), 3.19 (t, *J* = 8.8 Hz, 2H), 2.48 (s, 3H), 2.35 (d, *J* = 11.4 Hz, 1H), 2.20–2.02 (m, 3H), 1.72–1.53 (m, 5H), 1.45 (d, *J* = 6.9 Hz, 3H), 1.42–1.34 (m, 2H), 1.03 (s, 10H), 0.97 (d, *J* = 5.6 Hz, 2H). ^13^C NMR (101 MHz, chloroform-*d*) δ 173.2, 171.8, 170.0, 170.0, 169.4, 166.8, 158.3, 153.1, 150.3, 148.3, 147.6, 143.2, 143.2, 139.2, 138.6, 137.7, 133.8, 131.8, 131.5, 131.2, 130.7, 129.5, 129.4, 129.0, 128.8, 128.7, 128.6, 128.5, 127.0, 126.3, 125.6, 122.5, 119.3, 119.3, 116.9, 114.5, 113.8, 69.7, 67.5, 58.7, 57.3, 56.7, 55.1, 48.6, 48.3, 43.4, 42.8, 36.0, 35.9, 35.3, 28.7, 27.9, 26.6, 26.5, 25.6, 25.2, 22.1, 16.0. HRMS-ESI (*m*/*z*): [M + H^+^]^+^ calculated for C_64_H_70_N_10_O_7_S_2_, 1155.4943; found, 1155.4940. HPLC retention time: 6.1 min, purity >95%.

#### (2*S*,4*R*)-1-((*S*)-2-(7-(4-((2-(4-(4-Amino-3-(1-(2-phenylacetyl)­indolin-5-yl)­thieno­[3,2-*c*]­pyridin-7-yl)-1*H*-pyrazol-1-yl)­acetamido)­methyl)­phenoxy)­heptanamido)-3,3-dimethylbutanoyl)-4-hydroxy-*N*-((*S*)-1-(4-(4-methylthiazol-5-yl)­phenyl)­ethyl)­pyrrolidine-2-carboxamide (**z0z-214-10**)

Linker part was prepared according to the literature procedure^28^ ^1^H NMR (400 MHz, chloroform-*d*) δ 8.64 (s, 1H), 8.30 (d, *J* = 8.1 Hz, 1H), 7.93 (d, *J* = 5.7 Hz, 2H), 7.89 (s, 1H), 7.63 (d, *J* = 7.8 Hz, 1H), 7.44–7.18 (m, 14H), 7.14–7.04 (m, 4H), 6.76 (t, *J* = 7.0 Hz, 3H), 5.07 (dd, *J* = 15.5, 8.1 Hz, 3H), 4.91 (s, 2H), 4.68 (q, *J* = 9.6, 8.4 Hz, 2H), 4.49 (s, 1H), 4.34 (s, 2H), 4.10 (t, *J* = 8.1 Hz, 2H), 3.99 (d, *J* = 11.1 Hz, 1H), 3.81 (d, *J* = 7.8 Hz, 5H), 3.65 (d, *J* = 8.9 Hz, 1H), 3.16 (t, *J* = 8.5 Hz, 2H), 2.47 (s, 3H), 2.34 (t, *J* = 9.1 Hz, 1H), 2.11 (dt, *J* = 17.0, 8.6 Hz, 3H), 1.66 (p, *J* = 7.4, 6.7 Hz, 3H), 1.56 (dt, *J* = 14.7, 7.2 Hz, 2H), 1.45 (d, *J* = 6.8 Hz, 3H), 1.31 (dt, *J* = 32.5, 6.9 Hz, 6H), 1.05 (s, 11H). ^13^C NMR (101 MHz, chloroform-*d*) δ 173.1, 171.5, 170.0, 169.2, 166.7, 158.1, 152.9, 150.2, 148.0, 147.3, 143.1, 143.0, 138.8, 138.2, 137.5, 133.7, 131.7, 131.3, 131.0, 130.4, 129.3, 129.1, 128.8, 128.6, 128.5, 128.4, 126.8, 126.2, 125.4, 122.4, 119.0, 119.0, 116.6, 114.3, 113.6, 69.4, 67.4, 58.6, 57.1, 56.6, 54.8, 48.4, 48.1, 43.1, 42.7, 35.9, 35.2, 28.7, 28.6, 27.6, 26.5, 26.3, 25.4, 25.3, 21.9, 15.8. HRMS-ESI (*m*/*z*): [M + H^+^]^+^ calculated for C_65_H_72_N_10_O_7_S_2_, 1169.5100; found, 1169.5109. HPLC retention time: 6.3 min, purity >95%.

#### (2*S*,4*R*)-1-((*S*)-2-(2-(2-(4-(4-Amino-3-(1-(2-phenylacetyl)­indolin-5-yl)­thieno­[3,2-*c*]­pyridin-7-yl)-1*H*-pyrazol-1-yl)­acetamido)­acetamido)-3,3-dimethylbutanoyl)-4-hydroxy-*N*-((*S*)-1-(4-(4-methylthiazol-5-yl)­phenyl)­ethyl)­pyrrolidine-2-carboxamide (**z0z-216-9**)

^1^H NMR (400 MHz, chloroform-*d*) δ 8.59 (s, 1H), 8.30 (d, *J* = 8.2 Hz, 1H), 8.12 (s, 1H), 7.98–7.84 (m, 4H), 7.39–7.21 (m, 10H), 7.16 (d, *J* = 8.8 Hz, 2H), 6.99 (s, 1H), 5.17–4.92 (m, 3H), 4.86 (s, 2H), 4.77 (t, *J* = 8.5 Hz, 1H), 4.69 (d, *J* = 9.2 Hz, 1H), 4.44 (s, 1H), 4.21 (s, 1H), 4.12 (t, *J* = 8.5 Hz, 2H), 4.01 (d, *J* = 11.3 Hz, 1H), 3.83 (s, 3H), 3.65 (dd, *J* = 13.0, 7.6 Hz, 1H), 3.17 (t, *J* = 8.5 Hz, 2H), 2.42 (s, 3H), 2.19 (s, 2H), 1.43 (dd, *J* = 15.6, 6.8 Hz, 4H), 1.03 (s, 10H). ^13^C NMR (101 MHz, chloroform-*d*) δ 171.1, 170.6, 169.5, 169.1, 167.8, 152.9, 150.2, 148.3, 147.5, 143.4, 143.4, 138.3, 137.8, 133.9, 131.9, 131.6, 131.1, 130.5, 129.3, 129.1, 129.0, 128.8, 128.7, 127.1, 126.5, 125.6, 122.7, 119.3, 119.2, 117.0, 113.9, 70.3, 59.2, 57.9, 54.7, 53.7, 48.5, 48.4, 43.5, 42.5, 42.0, 37.5, 36.1, 28.0, 26.6, 22.2, 16.1. HRMS-ESI (*m*/*z*): [M + H^+^]^+^ calculated for C_53_H_56_N_10_O_6_S_2_, 993.3898; found, 993.3880. HPLC retention time: 9.5 min, purity >95%.

#### (2*S*,4*R*)-1-((*S*)-2-(3-(2-(4-(4-Amino-3-(1-(2-phenylacetyl)­indolin-5-yl)­thieno­[3,2-*c*]­pyridin-7-yl)-1*H*-pyrazol-1-yl)­acetamido)­propanamido)-3,3-dimethylbutanoyl)-4-hydroxy-*N*-((*S*)-1-(4-(4-methylthiazol-5-yl)­phenyl)­ethyl)­pyrrolidine-2-carboxamide (**z0z-216-10**)

^1^H NMR (400 MHz, chloroform-*d*) δ 8.64 (s, 1H), 8.34 (d, *J* = 8.2 Hz, 1H), 7.96 (d, *J* = 12.0 Hz, 2H), 7.86 (s, 1H), 7.50 (d, *J* = 7.9 Hz, 1H), 7.40–7.20 (m, 12H), 7.11 (d, *J* = 14.1 Hz, 2H), 6.81 (d, *J* = 8.6 Hz, 1H), 5.10 (t, *J* = 7.3 Hz, 1H), 4.90 (s, 2H), 4.77 (s, 2H), 4.69 (t, *J* = 8.2 Hz, 1H), 4.62 (d, *J* = 9.1 Hz, 1H), 4.51 (s, 1H), 4.16 (t, *J* = 8.4 Hz, 2H), 3.98 (d, *J* = 11.2 Hz, 1H), 3.85 (s, 2H), 3.63 (d, *J* = 11.8 Hz, 2H), 3.36 (s, 1H), 3.22 (t, *J* = 8.6 Hz, 2H), 2.45 (d, *J* = 29.2 Hz, 7H), 2.11 (t, *J* = 10.9 Hz, 1H), 1.46 (d, *J* = 7.0 Hz, 3H), 1.02 (s, 9H). ^13^C NMR (101 MHz, chloroform-*d*) δ 171.8, 171.6, 170.0, 169.5, 167.4, 153.1, 150.3, 148.5, 148.0, 143.5, 143.2, 139.4, 138.7, 137.9, 133.9, 131.9, 131.6, 131.2, 130.8, 129.5, 129.1, 128.9, 128.8, 127.2, 126.5, 125.7, 122.8, 119.7, 119.5, 117.1, 114.1, 69.9, 58.9, 57.6, 57.3, 55.2, 48.8, 48.4, 43.6, 36.2, 36.0, 35.5, 35.3, 28.0, 26.6, 22.2, 16.1. HRMS-ESI (*m*/*z*): [M+H^+^]^+^ calculated for C_54_H_58_N_10_O_6_S_2_, 1007.4055; found, 1007.4033. HPLC retention time: 9.5 min, purity >95%.

#### (2*S*,4*R*)-1-((*S*)-2-(4-(2-(4-(4-Amino-3-(1-(2-phenylacetyl)­indolin-5-yl)­thieno­[3,2-*c*]­pyridin-7-yl)-1*H*-pyrazol-1-yl)­acetamido)­butanamido)-3,3-dimethylbutanoyl)-4-hydroxy-*N*-((*S*)-1-(4-(4-methylthiazol-5-yl)­phenyl)­ethyl)­pyrrolidine-2-carboxamide (**z0z-216-11**)

^1^H NMR (400 MHz, chloroform-*d*) δ 8.64 (s, 1H), 8.33 (d, *J* = 8.2 Hz, 1H), 7.99–7.88 (m, 3H), 7.66 (d, *J* = 7.8 Hz, 1H), 7.40–7.17 (m, 16H), 7.09 (s, 1H), 5.08 (t, *J* = 7.2 Hz, 1H), 4.98–4.85 (m, 4H), 4.74 (t, *J* = 8.2 Hz, 1H), 4.60 (d, *J* = 8.8 Hz, 1H), 4.47 (s, 1H), 4.14 (t, *J* = 8.4 Hz, 2H), 4.03 (d, *J* = 11.4 Hz, 1H), 3.84 (s, 2H), 3.70–3.57 (m, 3H), 3.23 (dt, *J* = 18.0, 7.1 Hz, 5H), 3.08 (q, *J* = 7.4 Hz, 2H), 2.48 (s, 4H), 2.36 (s, 1H), 2.19 (dq, *J* = 23.0, 14.3, 10.8 Hz, 4H), 1.82–1.72 (m, 2H), 1.56–1.37 (m, 11H), 1.03 (s, 11H). ^13^C NMR (101 MHz, chloroform-*d*) δ 173.1, 171.8, 170.1, 169.5, 167.5, 152.8, 150.3, 148.4, 147.9, 143.4, 143.3, 138.5, 137.9, 133.9, 131.9, 131.6, 131.1, 130.7, 129.4, 129.1, 128.9, 128.8, 128.7, 127.1, 126.4, 125.6, 122.9, 119.4, 119.2, 117.0, 114.1, 69.9, 58.8, 57.9, 57.2, 55.1, 53.7, 48.7, 48.4, 43.5, 42.0, 38.8, 36.4, 35.4, 33.1, 28.0, 26.6, 25.0, 22.1, 16.1, 12.1. HRMS-ESI (*m*/*z*): [M+H^+^]^+^ calculated for C_55_H_60_N_10_O_6_S_2_, 1021.4211; found, 1021.4192. HPLC retention time: 9.6 min, purity >95%.

#### (2*S*,4*R*)-1-((*S*)-2-(5-(2-(4-(4-Amino-3-(1-(2-phenylacetyl)­indolin-5-yl)­thieno­[3,2-*c*]­pyridin-7-yl)-1*H*-pyrazol-1-yl)­acetamido)­pentanamido)-3,3-dimethylbutanoyl)-4-hydroxy-*N*-((*S*)-1-(4-(4-methylthiazol-5-yl)­phenyl)­ethyl)­pyrrolidine-2-carboxamide (**z0z-216-12**)

^1^H NMR (400 MHz, chloroform-*d*) δ 8.66 (d, *J* = 4.4 Hz, 1H), 8.35 (d, *J* = 8.2 Hz, 1H), 8.03 (s, 1H), 7.97 (s, 2H), 7.55 (d, *J* = 7.8 Hz, 1H), 7.40–7.23 (m, 12H), 7.12 (s, 1H), 6.67 (s, 1H), 6.62–6.55 (m, 1H), 5.09 (t, *J* = 7.2 Hz, 1H), 4.91 (s, 2H), 4.83 (s, 2H), 4.72 (t, *J* = 8.0 Hz, 1H), 4.61 (d, *J* = 9.0 Hz, 1H), 4.50 (s, 1H), 4.15 (t, *J* = 8.5 Hz, 2H), 4.05 (d, *J* = 11.3 Hz, 1H), 3.85 (s, 2H), 3.62 (dd, *J* = 11.4, 3.6 Hz, 1H), 3.30–3.14 (m, 4H), 2.54–2.39 (m, 5H), 2.19 (t, *J* = 7.1 Hz, 2H), 2.10 (dd, *J* = 13.4, 8.6 Hz, 1H), 1.60–1.52 (m, 2H), 1.46 (d, *J* = 6.9 Hz, 5H), 1.03 (s, 10H). ^13^C NMR (101 MHz, chloroform-*d*) δ 173.3, 172.0, 170.0, 169.5, 167.3, 153.1, 150.3, 148.5, 143.5, 143.3, 139.2, 138.9, 137.9, 133.9, 131.9, 131.6, 131.2, 130.8, 129.5, 129.1, 128.9, 128.8, 128.8, 127.2, 126.5, 125.7, 122.8, 119.5, 117.1, 69.8, 58.7, 57.6, 57.2, 55.3, 48.8, 48.4, 43.6, 38.6, 35.3, 28.4, 28.0, 26.6, 22.2, 16.1. HRMS-ESI (*m*/*z*): [M + H^+^]^+^ calculated for C_56_H_62_N_10_O_6_S_2_, 1035.4368; found, 1035.4351. HPLC retention time: 9.7 min, purity >95%.

#### (2*S*,4*R*)-1-((*S*)-2-(6-(2-(4-(4-Amino-3-(1-(2-phenylacetyl)­indolin-5-yl)­thieno­[3,2-*c*]­pyridin-7-yl)-1*H*-pyrazol-1-yl)­acetamido)­hexanamido)-3,3-dimethylbutanoyl)-4-hydroxy-*N*-((*S*)-1-(4-(4-methylthiazol-5-yl)­phenyl)­ethyl)­pyrrolidine-2-carboxamide (**z0z-216-13**)

^1^H NMR (400 MHz, chloroform-*d*) δ 8.68 (s, 1H), 8.36 (d, *J* = 8.2 Hz, 1H), 8.03–7.95 (m, 3H), 7.49 (d, *J* = 7.9 Hz, 1H), 7.43–7.25 (m, 15H), 7.15 (s, 1H), 6.65 (s, 1H), 6.47 (d, *J* = 8.9 Hz, 1H), 5.14–5.06 (m, 1H), 4.91 (s, 4H), 4.75 (t, *J* = 8.0 Hz, 1H), 4.64 (d, *J* = 8.8 Hz, 1H), 4.52 (s, 1H), 4.18 (t, *J* = 8.5 Hz, 2H), 4.11 (d, *J* = 11.2 Hz, 1H), 3.87 (s, 2H), 3.71 (p, *J* = 6.7 Hz, 1H), 3.62 (dd, *J* = 11.4, 3.6 Hz, 1H), 3.31–3.21 (m, 5H), 3.18 (t, *J* = 7.4 Hz, 2H), 2.54–2.42 (m, 6H), 2.19 (dp, *J* = 23.6, 8.9, 8.1 Hz, 5H), 1.60 (dt, *J* = 12.6, 7.1 Hz, 3H), 1.46 (dd, *J* = 16.8, 6.8 Hz, 16H), 1.29 (d, *J* = 9.1 Hz, 3H), 1.06 (s, 12H). ^13^C NMR (101 MHz, chloroform-*d*) δ 173.6, 172.0, 170.0, 169.6, 167.2, 153.0, 150.3, 148.5, 147.9, 143.5, 143.2, 138.8, 138.0, 134.0, 132.0, 131.6, 131.2, 130.9, 129.6, 129.1, 128.9, 128.8, 127.2, 126.5, 125.7, 122.8, 119.5, 117.1, 114.0, 69.9, 58.7, 57.5, 57.1, 55.5, 55.3, 48.8, 48.5, 43.6, 43.5, 39.3, 36.0, 35.9, 35.2, 28.6, 28.0, 26.6, 26.1, 24.9, 22.2, 16.1, 12.6. HRMS-ESI (*m*/*z*): [M + H^+^]^+^ calculated for C_57_H_64_N_10_O_6_S_2_, 1049.4524; found, 1049.4496. HPLC retention time: 9.7 min, purity >95%.

#### (2*S*,4*R*)-1-((*S*)-2-(7-(2-(4-(4-Amino-3-(1-(2-phenylacetyl)­indolin-5-yl)­thieno­[3,2-*c*]­pyridin-7-yl)-1*H*-pyrazol-1-yl)­acetamido)­heptanamido)-3,3-dimethylbutanoyl)-4-hydroxy-*N*-((*S*)-1-(4-(4-methylthiazol-5-yl)­phenyl)­ethyl)­pyrrolidine-2-carboxamide (**z0z-216-14**)

^1^H NMR (400 MHz, chloroform-*d*) δ 8.66 (d, *J* = 3.2 Hz, 1H), 8.34 (d, *J* = 8.2 Hz, 1H), 8.00 (d, *J* = 9.2 Hz, 2H), 7.94 (s, 1H), 7.59 (d, *J* = 7.7 Hz, 1H), 7.40–7.23 (m, 13H), 7.12 (s, 1H), 6.67 (dd, *J* = 19.9, 7.3 Hz, 2H), 5.09 (t, *J* = 7.2 Hz, 1H), 4.92 (q, *J* = 8.3, 7.2 Hz, 4H), 4.71 (t, *J* = 8.0 Hz, 1H), 4.64 (d, *J* = 9.0 Hz, 1H), 4.50 (s, 1H), 4.15 (t, *J* = 8.5 Hz, 2H), 4.06 (d, *J* = 11.3 Hz, 1H), 3.84 (s, 2H), 3.64 (dq, *J* = 11.3, 5.2, 3.9 Hz, 2H), 3.23 (q, *J* = 7.8, 7.0 Hz, 5H), 2.51 (d, *J* = 3.9 Hz, 4H), 2.38 (ddd, *J* = 13.1, 8.3, 4.8 Hz, 1H), 2.14 (ddt, *J* = 21.3, 13.4, 7.2 Hz, 4H), 1.62–1.38 (m, 14H), 1.24 (d, *J* = 13.0 Hz, 7H), 1.04 (s, 12H). ^13^C NMR (101 MHz, chloroform-*d*) δ 173.5, 171.8, 170.1, 169.4, 167.1, 153.1, 150.3, 148.4, 147.7, 143.4, 143.3, 139.1, 138.8, 137.9, 133.9, 131.8, 131.6, 131.2, 130.7, 129.4, 129.0, 128.8, 128.7, 127.1, 126.4, 125.7, 122.6, 119.5, 119.4, 117.0, 113.9, 69.8, 58.7, 57.4, 57.0, 55.2, 53.7, 48.7, 48.4, 43.5, 42.0, 39.2, 36.1, 36.0, 35.3, 28.7, 28.2, 28.0, 26.5, 26.0, 25.3, 22.2, 16.1. HRMS-ESI (*m*/*z*): [M + H^+^]^+^ calculated for C_58_H_66_N_10_O_6_S_2_, 1063.4681; found, 1063.4660. HPLC retention time: 10.1 min, purity >95%.

#### (2*S*,4*R*)-1-((*S*)-2-(8-(2-(4-(4-Amino-3-(1-(2-phenylacetyl)­indolin-5-yl)­thieno­[3,2-*c*]­pyridin-7-yl)-1*H*-pyrazol-1-yl)­acetamido)­octanamido)-3,3-dimethylbutanoyl)-4-hydroxy-*N*-((*S*)-1-(4-(4-methylthiazol-5-yl)­phenyl)­ethyl)­pyrrolidine-2-carboxamide (**z0z-216-15**)

^1^H NMR (400 MHz, chloroform-*d*) δ 8.66 (d, *J* = 2.6 Hz, 1H), 8.31 (d, *J* = 8.5 Hz, 1H), 7.99–7.95 (m, 3H), 7.50 (d, *J* = 7.9 Hz, 1H), 7.39–7.21 (m, 16H), 7.14 (s, 1H), 6.70 (t, *J* = 6.2 Hz, 2H), 5.05 (q, *J* = 6.7 Hz, 3H), 4.89 (s, 2H), 4.69 (t, *J* = 8.1 Hz, 2H), 4.61 (d, *J* = 8.9 Hz, 2H), 4.49 (s, 2H), 4.16 (t, *J* = 8.4 Hz, 3H), 4.05 (d, *J* = 11.3 Hz, 2H), 3.84 (s, 3H), 3.64 (dq, *J* = 13.6, 6.5 Hz, 7H), 3.22 (q, *J* = 6.6 Hz, 5H), 3.13 (q, *J* = 7.4 Hz, 5H), 2.50 (d, *J* = 3.6 Hz, 5H), 2.38–2.30 (m, 2H), 2.14 (dq, *J* = 21.3, 7.0 Hz, 4H), 1.53–1.33 (m, 41H), 1.23 (d, *J* = 19.6 Hz, 13H), 1.03 (s, 14H). ^13^C NMR (101 MHz, chloroform-*d*) δ 173.9, 171.8, 170.2, 169.6, 167.2, 152.9, 150.4, 148.3, 147.9, 143.4, 143.3, 138.7, 138.2, 137.9, 133.9, 132.2, 131.6, 131.0, 130.7, 129.4, 129.1, 128.8, 128.6, 127.1, 126.4, 125.7, 123.0, 119.4, 119.2, 117.0, 114.0, 69.8, 58.8, 57.5, 56.9, 55.2, 55.1, 48.8, 48.4, 46.4, 46.4, 43.4, 43.2, 39.4, 36.2, 35.3, 28.7, 28.5, 28.5, 27.9, 26.5, 26.5, 26.4, 26.3, 26.1, 25.3, 22.2, 16.0, 12.6. HRMS-ESI (*m*/*z*): [M + H^+^]^+^ calculated for C_59_H_68_N_10_O_6_S_2_, 1077.4837; found, 1077.4828. HPLC retention time: 6.0 min, purity >95%.

#### (2*S*,4*R*)-1-((*S*)-2-(9-(2-(4-(4-Amino-3-(1-(2-phenylacetyl)­indolin-5-yl)­thieno­[3,2-*c*]­pyridin-7-yl)-1*H*-pyrazol-1-yl)­acetamido)­nonanamido)-3,3-dimethylbutanoyl)-4-hydroxy-*N*-((*S*)-1-(4-(4-methylthiazol-5-yl)­phenyl)­ethyl)­pyrrolidine-2-carboxamide (**z0z-216-16**)

^1^H NMR (400 MHz, chloroform-*d*) δ 8.66 (s, 1H), 8.32 (d, *J* = 8.2 Hz, 1H), 7.98 (s, 2H), 7.92 (s, 1H), 7.53 (d, *J* = 7.9 Hz, 1H), 7.39–7.21 (m, 13H), 7.11 (s, 1H), 6.69 (d, *J* = 8.5 Hz, 1H), 6.55 (s, 1H), 5.11–5.04 (m, 1H), 4.97 (s, 2H), 4.88 (s, 2H), 4.68 (t, *J* = 8.0 Hz, 1H), 4.55 (d, *J* = 8.5 Hz, 1H), 4.48 (s, 1H), 4.11 (dt, *J* = 21.0, 9.9 Hz, 4H), 3.83 (s, 2H), 3.66–3.56 (m, 2H), 3.22 (d, *J* = 7.8 Hz, 5H), 2.50 (s, 4H), 2.42–2.31 (m, 1H), 2.07 (q, *J* = 7.7, 6.7 Hz, 3H), 1.56–1.32 (m, 11H), 1.16 (s, 9H), 1.04 (s, 11H). ^13^C NMR (101 MHz, chloroform-*d*) δ 174.0, 171.9, 170.1, 169.5, 167.2, 153.2, 150.3, 148.4, 147.7, 143.4, 143.3, 138.9, 137.9, 133.9, 132.0, 131.6, 131.2, 130.8, 129.5, 129.1, 128.8, 128.7, 128.6, 127.1, 126.5, 125.7, 122.7, 119.5, 119.4, 117.0, 113.9, 69.9, 58.8, 57.8, 56.8, 55.2, 48.8, 48.4, 43.5, 39.4, 36.1, 35.0, 28.9, 28.8, 28.7, 28.6, 28.0, 26.6, 26.3, 25.4, 22.1, 16.1. HRMS-ESI (*m*/*z*): [M + H^+^]^+^ calculated for C_60_H_70_N_10_O_6_S_2_, 1091.4994; found, 1091.4978. HPLC retention time: 10.7 min, purity >95%.

#### (2*S*,4*R*)-1-((*S*)-2-(2-(4-(2-(4-(4-Amino-3-(1-(2-phenylacetyl)­indolin-5-yl)­thieno­[3,2-*c*]­pyridin-7-yl)-1*H*-pyrazol-1-yl)­acetamido)­phenoxy)­acetamido)-3,3-dimethylbutanoyl)-4-hydroxy-*N*-((S)-1-(4-(4-methylthiazol-5-yl)­phenyl)­ethyl)­pyrrolidine-2-carboxamide (**z0z-217-10**)

Linker part was prepared according to the literature procedure^28^ ^1^H NMR (400 MHz, chloroform-*d*) δ 8.96 (s, 1H), 8.65 (s, 1H), 8.34 (d, *J* = 8.2 Hz, 1H), 7.97 (d, *J* = 20.6 Hz, 3H), 7.73–7.59 (m, 3H), 7.50 (d, *J* = 7.7 Hz, 1H), 7.46–7.20 (m, 18H), 7.11 (s, 1H), 6.78 (d, *J* = 8.5 Hz, 2H), 5.08–4.98 (m, 3H), 4.83 (s, 1H), 4.70 (t, *J* = 8.0 Hz, 1H), 4.62 (d, *J* = 9.1 Hz, 1H), 4.49 (s, 1H), 4.41 (t, *J* = 15.7 Hz, 2H), 4.15 (t, *J* = 8.4 Hz, 2H), 4.02 (d, *J* = 11.3 Hz, 1H), 3.85 (s, 2H), 3.61 (d, *J* = 10.7 Hz, 1H), 3.56–3.42 (m, 1H), 3.22 (t, *J* = 8.5 Hz, 2H), 2.50 (s, 5H), 2.04 (d, *J* = 45.1 Hz, 6H), 1.46 (d, *J* = 7.0 Hz, 3H), 1.01 (s, 10H). ^13^C NMR (101 MHz, chloroform-*d*) δ 171.0, 169.9, 169.5, 164.9, 153.9, 150.3, 148.4, 147.8, 143.3, 137.9, 134.0, 133.8, 133.7, 131.9, 131.8, 131.7, 131.3, 130.8, 130.6, 130.5, 129.5, 129.1, 128.9, 128.8, 127.2, 126.4, 125.7, 122.7, 122.0, 119.5, 117.1, 115.0, 114.0, 70.1, 67.4, 58.6, 57.1, 56.9, 55.6, 48.9, 48.4, 43.6, 36.0, 35.6, 28.0, 26.5, 22.3, 16.1. HRMS-ESI (*m*/*z*): [M + H^+^]^+^ calculated for C_59_H_60_N_10_O_7_S_2_, 1085.4161; found, 1085.4119. HPLC retention time: 5.9 min, purity >95%.

#### (2*S*,4*R*)-1-((*S*)-2-(4-(4-(2-(4-(4-Amino-3-(1-(2-phenylacetyl)­indolin-5-yl)­thieno­[3,2-*c*]­pyridin-7-yl)-1*H*-pyrazol-1-yl)­acetamido)­phenoxy)­butanamido)-3,3-dimethylbutanoyl)-4-hydroxy-*N*-((S)-1-(4-(4-methylthiazol-5-yl)­phenyl)­ethyl)­pyrrolidine-2-carboxamide (**z0z-217-11**)

Linker part was prepared according to the literature procedure^28^ ^1^H NMR (400 MHz, chloroform-*d*) δ 8.88 (s, 1H), 8.65 (d, *J* = 3.8 Hz, 1H), 8.33 (d, *J* = 8.1 Hz, 1H), 7.98–7.87 (m, 3H), 7.52 (d, *J* = 7.6 Hz, 1H), 7.38–7.20 (m, 16H), 7.10 (s, 1H), 6.71 (t, *J* = 8.8 Hz, 4H), 6.58 (d, *J* = 8.1 Hz, 1H), 5.09–5.02 (m, 1H), 4.96 (d, *J* = 17.4 Hz, 3H), 4.68–4.57 (m, 3H), 4.46 (s, 1H), 4.13 (t, *J* = 8.4 Hz, 2H), 4.03 (d, *J* = 11.5 Hz, 1H), 3.92 (d, *J* = 8.2 Hz, 1H), 3.83 (d, *J* = 6.3 Hz, 4H), 3.70–3.57 (m, 2H), 3.20 (t, *J* = 8.6 Hz, 2H), 3.09 (d, *J* = 7.4 Hz, 1H), 2.49 (d, *J* = 4.8 Hz, 6H), 2.34 (dp, *J* = 14.8, 7.8 Hz, 5H), 2.09 (d, *J* = 10.2 Hz, 2H), 1.99 (d, *J* = 7.7 Hz, 3H), 1.45 (dd, *J* = 10.7, 6.5 Hz, 8H), 1.01 (s, 12H). ^13^C NMR (101 MHz, cChloroform-*d*) δ 173.1, 171.7, 170.1, 169.5, 164.9, 155.9, 153.0, 150.3, 148.4, 147.9, 143.4, 143.4, 138.8, 137.9, 133.9, 131.9, 131.6, 131.2, 130.7, 130.3, 129.5, 129.1, 129.0, 128.8, 128.7, 127.2, 126.4, 125.7, 122.8, 122.1, 119.4, 119.3, 117.0, 116.5, 115.7, 114.7, 114.0, 69.9, 67.0, 58.7, 57.7, 56.9, 55.5, 54.0, 48.8, 48.4, 43.5, 42.2, 36.1, 35.3, 32.6, 28.0, 26.5, 25.0, 22.2, 16.1, 12.1. HRMS-ESI (*m*/*z*): [M + H^+^]^+^ calculated for C_61_H_64_N_10_O_7_S_2_, 1113.4474; found, 1113.4467. HPLC retention time: 9.2 min, purity >95%.

#### (2*S*,4*R*)-1-((*S*)-2-(5-(4-(2-(4-(4-Amino-3-(1-(2-phenylacetyl)­indolin-5-yl)­thieno­[3,2-*c*]­pyridin-7-yl)-1*H*-pyrazol-1-yl)­acetamido)­phenoxy)­pentanamido)-3,3-dimethylbutanoyl)-4-hydroxy-*N*-((*S*)-1-(4-(4-methylthiazol-5-yl)­phenyl)­ethyl)­pyrrolidine-2-carboxamide (**z0z-217-12**)

Linker part was prepared according to the literature procedure^2^ ^1^H NMR (400 MHz, chloroform-*d*) δ 9.19 (s, 1H), 8.63 (s, 1H), 8.30 (d, *J* = 8.1 Hz, 1H), 7.94 (d, *J* = 6.8 Hz, 3H), 7.63 (d, *J* = 7.6 Hz, 1H), 7.38–7.14 (m, 19H), 7.00 (d, *J* = 31.8 Hz, 2H), 6.65 (d, *J* = 8.2 Hz, 3H), 5.10–4.87 (m, 6H), 4.74–4.59 (m, 3H), 4.47 (s, 1H), 4.07 (s, 4H), 3.84–3.56 (m, 9H), 3.14 (s, 3H), 2.46 (s, 4H), 2.20 (d, *J* = 63.0 Hz, 6H), 1.62 (s, 6H), 1.41 (d, *J* = 6.9 Hz, 4H), 1.03 (s, 16H). ^13^C NMR (101 MHz, chloroform-*d*) δ 173.4, 171.7, 170.2, 169.5, 164.9, 155.8, 153.0, 150.4, 148.2, 147.6, 143.3, 138.9, 137.7, 133.9, 131.9, 131.5, 131.2, 130.5, 130.4, 129.3, 129.0, 128.7, 127.0, 126.3, 125.6, 122.7, 121.8, 119.3, 119.1, 116.9, 114.5, 69.7, 67.5, 58.9, 57.6, 48.7, 48.3, 43.3, 36.4, 35.6, 35.3, 28.5, 27.8, 26.5, 22.2, 22.0, 16.0. HRMS-ESI (*m*/*z*): [M + H^+^]^+^ calculated for C_62_H_66_N_10_O_7_S_2_, 1127.4630; found, 1127.4603. HPLC retention time: 9.4 min, purity >95%.

#### (2*S*,4*R*)-1-((*S*)-2-(6-(4-(2-(4-(4-Amino-3-(1-(2-phenylacetyl)­indolin-5-yl)­thieno­[3,2-*c*]­pyridin-7-yl)-1*H*-pyrazol-1-yl)­acetamido)­phenoxy)­hexanamido)-3,3-dimethylbutanoyl)-4-hydroxy-*N*-((*S*)-1-(4-(4-methylthiazol-5-yl)­phenyl)­ethyl)­pyrrolidine-2-carboxamide (**z0z-217-13**)

Linker part was prepared according to the literature procedure^28^ ^1^H NMR (400 MHz, chloroform-*d*) δ 8.83 (s, 1H), 8.56 (s, 1H), 8.24 (d, *J* = 8.2 Hz, 1H), 7.88 (d, *J* = 8.4 Hz, 3H), 7.44 (d, *J* = 7.9 Hz, 1H), 7.31–7.10 (m, 16H), 7.00 (s, 1H), 6.62 (dd, *J* = 13.7, 6.6 Hz, 3H), 5.02–4.81 (m, 6H), 4.63 (t, *J* = 7.7 Hz, 1H), 4.54 (d, *J* = 8.9 Hz, 1H), 4.40 (s, 1H), 4.07–3.93 (m, 3H), 3.71 (d, *J* = 20.5 Hz, 5H), 3.57–3.48 (m, 2H), 3.09 (d, *J* = 9.0 Hz, 2H), 2.40 (d, *J* = 3.4 Hz, 3H), 2.28 (s, 1H), 2.19 (d, *J* = 10.0 Hz, 1H), 2.05 (dt, *J* = 22.6, 11.0 Hz, 3H), 1.63–1.42 (m, 7H), 1.38–1.23 (m, 7H), 0.95 (s, 12H). ^13^C NMR (101 MHz, chloroform-*d*) δ 173.5, 171.9, 170.0, 169.5, 164.8, 156.0, 153.1, 150.3, 148.3, 147.7, 143.3, 143.2, 138.9, 138.7, 137.8, 133.9, 131.9, 131.6, 131.2, 130.7, 130.2, 129.4, 129.1, 128.9, 128.8, 128.7, 127.1, 126.4, 125.6, 122.7, 121.9, 119.4, 119.3, 116.9, 114.6, 113.9, 69.8, 67.7, 58.8, 57.5, 56.9, 55.5, 48.7, 48.4, 43.4, 36.1, 35.3, 28.8, 27.9, 26.7, 26.5, 25.6, 25.3, 22.1, 16.0. HRMS-ESI (*m*/*z*): [M + H^+^]^+^ calculated for C_63_H_68_N_10_O_7_S_2_, 1141.4787; found, 1141.4752. HPLC retention time: 6.2 min, purity >95%.

#### (2*S*,4*R*)-1-((*S*)-2-(2-(2-(4-((2-(4-(4-Amino-3-(1-(2-phenylacetyl)­indolin-5-yl)­thieno­[3,2-*c*]­pyridin-7-yl)-1*H*-pyrazol-1-yl)­acetamido)­methyl)­phenoxy)­ethoxy)­acetamido)-3,3-dimethylbutanoyl)-4-hydroxy-*N*-((*S*)-1-(4-(4-methylthiazol-5-yl)­phenyl)­ethyl)­pyrrolidine-2-carboxamide (**z0z-224-5**)

Linker part was prepared according to the literature procedure^28^ ^1^H NMR (400 MHz, chloroform-*d*) δ 8.66 (d, *J* = 3.1 Hz, 1H), 8.34 (d, *J* = 6.0 Hz, 1H), 7.94–7.85 (m, 2H), 7.45 (d, *J* = 7.3 Hz, 1H), 7.40–7.23 (m, 14H), 7.18–7.11 (m, 2H), 6.86 (dd, *J* = 8.8, 3.0 Hz, 1H), 6.81–6.70 (m, 1H), 5.06 (d, *J* = 7.8 Hz, 2H), 4.93 (d, *J* = 3.0 Hz, 2H), 4.73–4.64 (m, 1H), 4.55–4.45 (m, 2H), 4.38 (d, *J* = 4.8 Hz, 1H), 4.21–4.13 (m, 2H), 4.12–3.95 (m, 5H), 3.89–3.78 (m, 4H), 3.60 (d, *J* = 10.6 Hz, 1H), 3.24 (s, 2H), 2.51 (d, *J* = 3.1 Hz, 4H), 2.23 (s, 5H), 2.05 (d, *J* = 11.9 Hz, 2H), 1.47 (dd, *J* = 7.1, 3.2 Hz, 3H), 1.05 (d, *J* = 3.1 Hz, 9H). ^13^C NMR (126 MHz, chloroform-*d*) δ 171.5, 170.2, 169.7, 166.8, 158.0, 150.4, 148.5, 143.2, 139.1, 133.9, 131.6, 130.9, 130.3, 129.8, 129.6, 129.2, 129.1, 129.0, 128.9, 128.8, 127.2, 126.5, 125.6, 117.3, 114.9, 114.8, 70.6, 70.5, 70.4, 70.2, 70.1, 67.4, 67.3, 58.5, 57.1, 56.6, 55.4, 48.9, 48.5, 43.6, 43.0, 35.6, 35.3, 29.8, 29.7, 28.0, 26.6, 22.3, 22.3, 16.1. HRMS-ESI (*m*/*z*): [M + H^+^]^+^ calculated for C_62_H_66_N_10_O_8_S_2_, 1143.4579; found, 1143.4559. HPLC retention time: 6.0 min, purity >95%.

#### (2*S*,4*R*)-1-((*S*)-2-(2-(2-(4-(2-(2-(4-(4-Amino-3-(1-(2-phenylacetyl)­indolin-5-yl)­thieno­[3,2-*c*]­pyridin-7-yl)-1*H*-pyrazol-1-yl)­acetamido)­ethyl)­phenoxy)­ethoxy)­acetamido)-3,3-dimethylbutanoyl)-4-hydroxy-*N*-((*S*)-1-(4-(4-methylthiazol-5-yl)­phenyl)­ethyl)­pyrrolidine-2-carboxamide (**z0z-224-6**)

Linker part was prepared according to the literature procedure^28^ ^1^H NMR (400 MHz, acetone-*d*_6_) δ 8.82 (s, 1H), 8.30 (d, *J* = 8.2 Hz, 1H), 8.23 (s, 1H), 7.92 (s, 2H), 7.80–7.76 (m, 1H), 7.74 (s, 1H), 7.52 (d, *J* = 9.3 Hz, 1H), 7.46–7.23 (m, 13H), 7.04 (d, *J* = 8.4 Hz, 2H), 6.83 (d, *J* = 8.1 Hz, 2H), 6.73 (s, 1H), 5.08–5.02 (m, 1H), 4.95 (d, *J* = 15.3 Hz, 2H), 4.75–4.70 (m, 1H), 4.63 (t, *J* = 8.1 Hz, 1H), 4.50–4.46 (m, 1H), 4.28 (t, *J* = 8.5 Hz, 2H), 4.11–4.03 (m, 4H), 3.93–3.81 (m, 5H), 3.75 (dd, *J* = 10.9, 4.1 Hz, 1H), 3.45 (q, *J* = 6.6 Hz, 2H), 3.26 (t, *J* = 8.5 Hz, 2H), 2.70 (t, *J* = 7.0 Hz, 2H), 2.42 (s, 3H), 2.16–2.10 (m, 2H), 1.45 (d, *J* = 7.0 Hz, 3H), 1.03 (d, *J* = 6.1 Hz, 9H). ^13^C NMR (101 MHz, acetone-*d*_6_) δ 210.0, 171.6, 171.5, 171.1, 170.4, 170.3, 170.2, 167.7, 167.6, 158.2, 151.9, 151.4, 150.4, 149.0, 145.1, 144.9, 139.9, 138.6, 135.8, 134.3, 132.1, 132.0, 131.8, 131.3, 130.6, 130.5, 130.3, 130.0, 129.9, 129.5, 129.2, 129.1, 128.4, 128.1, 127.5, 127.4, 127.0, 126.5, 120.5, 117.6, 116.7, 116.0, 115.4, 115.3, 71.1, 70.8, 70.5, 69.3, 67.9, 60.3, 57.6, 57.5, 57.4, 55.6, 49.4, 49.3, 49.2, 43.4, 41.5, 38.3, 37.1, 36.6, 35.0, 26.9, 26.8, 22.6, 22.6, 16.3. HRMS-ESI (*m*/*z*): [M + H^+^]^+^ calculated for C_63_H_68_N_10_O_8_S_2_, 1157.4736; found, 1157.4721. HPLC retention time: 6.0 min, purity >95%.

#### (2*S*,4*R*)-1-((*S*)-2-(2-(2-(2-(4-((2-(4-(4-Amino-3-(1-(2-phenylacetyl)­indolin-5-yl)­thieno­[3,2-*c*]­pyridin-7-yl)-1*H*-pyrazol-1-yl)­acetamido)­methyl)­phenoxy)­ethoxy)­ethoxy)­acetamido)-3,3-dimethylbutanoyl)-4-hydroxy-*N*-((*S*)-1-(4-(4-methylthiazol-5-yl)­phenyl)­ethyl)­pyrrolidine-2-carboxamide (**z0z-224-7**)

Linker part was prepared according to the literature procedure^28^ ^1^H NMR (400 MHz, chloroform-*d*) δ 8.65 (s, 1H), 8.34 (d, *J* = 8.2 Hz, 1H), 7.95–7.90 (m, 3H), 7.50 (d, *J* = 7.8 Hz, 1H), 7.39–7.23 (m, 15H), 7.16–7.09 (m, 3H), 7.01 (t, *J* = 5.7 Hz, 1H), 6.85–6.80 (m, 2H), 5.05 (dd, *J* = 14.5, 7.3 Hz, 3H), 4.93 (s, 2H), 4.71 (t, *J* = 7.8 Hz, 1H), 4.57 (d, *J* = 8.8 Hz, 1H), 4.53–4.46 (m, 1H), 4.36 (d, *J* = 5.6 Hz, 2H), 4.21–4.08 (m, 4H), 4.01 (d, *J* = 11.1 Hz, 2H), 3.96–3.80 (m, 7H), 3.72–3.61 (m, 7H), 3.23 (t, *J* = 8.5 Hz, 2H), 2.50 (s, 5H), 2.05 (dd, *J* = 13.6, 8.4 Hz, 1H), 1.45 (d, *J* = 6.9 Hz, 6H), 1.03 (s, 10H). ^13^C NMR (101 MHz, chloroform-*d*) δ 171.3, 170.3, 169.8, 169.5, 166.8, 158.1, 152.8, 150.3, 148.4, 148.1, 143.5, 143.2, 138.8, 138.0, 133.9, 132.0, 131.6, 131.0, 130.8, 129.9, 129.5, 129.1, 129.0, 128.8, 128.7, 127.1, 126.4, 125.7, 123.0, 119.5, 119.2, 117.1, 114.9, 114.7, 114.0, 71.2, 70.7, 70.4, 69.9, 69.9, 67.3, 58.5, 57.0, 56.7, 55.2, 54.1, 48.8, 48.4, 43.5, 43.0, 42.3, 35.6, 35.3, 28.0, 26.5, 22.3, 16.1, 12.1. HRMS-ESI (*m*/*z*): [M + H^+^]^+^ calculated for C_64_H_70_N_10_O_9_S_2_, 1187.4841; found, 1187.4816. HPLC retention time: 6.0 min, purity >95%.

#### (2*S*,4*R*)-1-((*S*)-2-(2-(2-(2-(4-(2-(2-(4-(4-Amino-3-(1-(2-phenylacetyl)­indolin-5-yl)­thieno­[3,2-*c*]­pyridin-7-yl)-1*H*-pyrazol-1-yl)­acetamido)­ethyl)­phenoxy)­ethoxy)­ethoxy)­acetamido)-3,3-dimethylbutanoyl)-4-hydroxy-*N*-((*S*)-1-(4-(4-methylthiazol-5-yl)­phenyl)­ethyl)­pyrrolidine-2-carboxamide (**z0z-224-8**)

Linker part was prepared according to the literature procedure^28^ ^1^H NMR (400 MHz, chloroform-*d*) δ 8.64 (s, 1H), 8.33 (d, *J* = 8.2 Hz, 1H), 7.93 (d, *J* = 15.8 Hz, 2H), 7.85 (s, 1H), 7.56 (d, *J* = 7.4 Hz, 1H), 7.31 (dt, *J* = 33.1, 7.7 Hz, 15H), 7.16 (s, 1H), 6.96 (d, *J* = 8.3 Hz, 2H), 6.74 (t, *J* = 6.6 Hz, 2H), 6.56 (d, *J* = 6.0 Hz, 1H), 5.08–4.96 (m, 3H), 4.85 (s, 2H), 4.72 (t, *J* = 7.9 Hz, 1H), 4.57 (d, *J* = 8.7 Hz, 1H), 4.48 (s, 1H), 4.16 (t, *J* = 8.4 Hz, 2H), 4.02–3.89 (m, 6H), 3.86–3.73 (m, 5H), 3.65 (dq, *J* = 13.8, 7.0 Hz, 9H), 3.45 (q, *J* = 6.6 Hz, 3H), 3.22 (d, *J* = 8.7 Hz, 2H), 3.12 (d, *J* = 7.4 Hz, 2H), 2.68 (t, *J* = 7.1 Hz, 2H), 2.49 (s, 3H), 2.41 (dd, *J* = 14.2, 6.5 Hz, 1H), 2.06 (t, *J* = 10.6 Hz, 1H), 1.45 (d, *J* = 6.8 Hz, 3H), 1.36 (d, *J* = 6.5 Hz, 11H), 1.03 (s, 11H). ^13^C NMR (101 MHz, chloroform-*d*) δ 171.2, 170.2, 170.0, 169.5, 167.0, 157.3, 152.8, 150.2, 148.3, 147.8, 143.4, 143.3, 138.7, 137.9, 133.9, 132.0, 131.5, 131.0, 130.6, 130.6, 129.7, 129.6, 129.4, 129.1, 128.7, 128.6, 128.5, 127.0, 126.3, 125.6, 122.9, 119.3, 119.2, 116.9, 114.6, 114.6, 114.5, 113.9, 71.0, 70.5, 70.3, 69.8, 69.7, 67.2, 58.6, 57.0, 56.6, 55.1, 55.1, 48.8, 48.4, 43.4, 43.2, 40.8, 35.9, 35.3, 34.3, 27.9, 26.4, 22.2, 16.0, 12.5. HRMS-ESI (*m*/*z*): [M + H^+^]^+^ calculated for C_65_H_72_N_10_O_9_S_2_, 1201.4998; found, 1201.4968. HPLC retention time: 7.8 min, purity >95%.

#### (2*S*,4*R*)-1-((*S*)-2-(2-(4-(1-(2-(4-(4-Amino-3-(1-(2-phenylacetyl)­indolin-5-yl)­thieno­[3,2-*c*]­pyridin-7-yl)-1*H*-pyrazol-1-yl)­acetyl)­piperidin-4-yl)­piperazin-1-yl)­acetamido)-3,3-dimethylbutanoyl)-4-hydroxy-*N*-((*S*)-1-(4-(4-methylthiazol-5-yl)­phenyl)­ethyl)­pyrrolidine-2-carboxamide (**z0z-225-2**)

^1^H NMR (500 MHz, DMSO-*d*_6_) δ 8.99 (s, 1H), 8.43 (d, *J* = 7.7 Hz, 1H), 8.25 (s, 1H), 8.21 (d, *J* = 8.3 Hz, 1H), 8.10 (s, 1H), 7.98 (s, 1H), 7.84 (s, 1H), 7.44 (d, *J* = 7.8 Hz, 2H), 7.42–7.26 (m, 12H), 6.73 (s, 1H), 5.31 (q, *J* = 16.8 Hz, 2H), 4.92 (p, *J* = 5.8, 5.0 Hz, 2H), 4.55–4.38 (m, 4H), 4.27 (dd, *J* = 17.5, 9.0 Hz, 4H), 4.11 (d, *J* = 12.1 Hz, 1H), 3.91 (s, 2H), 3.67–3.27 (m, 20H), 3.24 (t, *J* = 8.5 Hz, 4H), 3.15–3.01 (m, 7H), 2.84–2.73 (m, 2H), 2.67–2.60 (m, 1H), 2.17 (d, *J* = 12.1 Hz, 2H), 2.11–2.02 (m, 3H), 1.77 (tdd, *J* = 13.1, 8.7, 5.3 Hz, 3H), 1.57–1.47 (m, 2H), 1.41–1.33 (m, 8H), 1.29–1.22 (m, 6H), 0.96 (d, *J* = 3.4 Hz, 13H). ^13^C NMR (126 MHz, DMSO-*d*_6_) δ 170.5, 169.5, 165.1, 151.5, 147.8, 144.7, 143.7, 138.4, 136.9, 135.0, 133.1, 131.1, 129.8, 129.7, 129.5, 128.8, 128.3, 126.6, 126.4, 125.7, 119.3, 116.1, 114.3, 73.1, 68.8, 58.6, 56.5, 54.9, 53.5, 53.0, 51.4, 48.0, 47.7, 42.1, 41.7, 37.8, 35.6, 28.2, 28.0, 27.5, 27.1, 26.5, 26.4, 22.5, 18.0, 16.7, 16.0, 12.3. HRMS-ESI (*m*/*z*): [M+H^+^] ^+^ calculated for C_62_H_72_N_12_O_6_S_2_, 1145.5212; found, 1145.5222. HPLC retention time: 4.9 min, purity >95%.

#### (2*S*,4*R*)-1-((*S*)-2-(2-(4-(2-(4-(4-Amino-3-(1-(2-phenylacetyl)­indolin-5-yl)­thieno­[3,2-*c*]­pyridin-7-yl)-1*H*-pyrazol-1-yl)­acetyl)­piperazin-1-yl)­acetamido)-3,3-dimethylbutanoyl)-4-hydroxy-*N*-((*S*)-1-(4-(4-methylthiazol-5-yl)­phenyl)­ethyl)­pyrrolidine-2-carboxamide (**z0z-225-3**)

Linker part was prepared according to the literature procedure^29^ ^1^H NMR (500 MHz, DMSO-*d*_6_) δ 10.62 (s, 1H), 9.03 (s, 2H), 8.82–8.60 (m, 4H), 8.44 (d, *J* = 7.6 Hz, 1H), 8.26 (s, 1H), 8.20 (d, *J* = 8.3 Hz, 2H), 8.12 (d, *J* = 10.4 Hz, 1H), 8.01 (s, 1H), 7.91 (s, 1H), 7.35 (dtd, *J* = 34.9, 23.0, 20.7, 7.2 Hz, 14H), 7.14 (d, *J* = 17.2 Hz, 1H), 5.43–5.31 (m, 2H), 4.92 (t, *J* = 7.1 Hz, 2H), 4.60–4.36 (m, 7H), 4.32–4.06 (m, 8H), 3.90 (s, 3H), 3.58 (dd, *J* = 35.0, 24.3 Hz, 6H), 3.23 (t, *J* = 8.0 Hz, 3H), 3.08–2.95 (m, 8H), 2.68 (s, 2H), 2.44 (d, *J* = 14.3 Hz, 4H), 2.10–2.01 (m, 1H), 1.81 (d, *J* = 6.9 Hz, 2H), 1.73 (d, *J* = 6.1 Hz, 7H), 1.38 (d, *J* = 6.8 Hz, 3H), 1.29–1.21 (m, 4H), 0.97 (d, *J* = 12.5 Hz, 10H). ^13^C NMR (126 MHz, DMSO-*d*_6_) δ 170.4, 169.5, 168.5, 165.6, 165.5, 163.7, 151.7, 150.7, 149.0, 147.5, 144.7, 143.7, 138.6, 137.2, 134.9, 133.2, 130.0, 129.6, 129.5, 128.8, 128.3, 128.2, 127.7, 126.6, 126.4, 125.6, 125.3, 119.4, 116.1, 115.0, 114.3, 68.7, 68.1, 58.6, 57.1, 56.4, 56.1, 53.3, 52.9, 51.4, 51.3, 48.0, 47.7, 45.9, 44.5, 42.1, 38.2, 37.8, 34.1, 27.4, 26.3, 25.9, 25.8, 23.7, 22.4, 18.0, 16.7, 15.9, 12.2. HRMS-ESI (*m*/*z*): [M + H^+^]^+^ calculated for C_57_H_63_N_11_O_6_S_2_, 1062.4477; found, 1062.4480. HPLC retention time: 5.2 min, purity >95%.

#### (2*S*,4*R*)-1-((*S*)-2-(2-(4-((1-(2-(4-(4-Amino-3-(1-(2-phenylacetyl)­indolin-5-yl)­thieno­[3,2-*c*]­pyridin-7-yl)-1*H*-pyrazol-1-yl)­acetyl)­piperidin-4-yl)­methyl)­piperazin-1-yl)­acetamido)-3,3-dimethylbutanoyl)-4-hydroxy-*N*-((*S*)-1-(4-(4-methylthiazol-5-yl)­phenyl)­ethyl)­pyrrolidine-2-carboxamide (**z0z-225-4**)

Linker part was prepared according to the literature procedure^29^ ^1^H NMR (400 MHz, DMSO-*d*_6_) δ 8.98 (s, 1H), 8.15 (dd, *J* = 34.0, 12.7 Hz, 3H), 7.93 (s, 1H), 7.37 (ddt, *J* = 32.1, 23.4, 17.9 Hz, 11H), 5.75 (s, 5H), 5.56 (s, 1H), 5.31–5.10 (m, 2H), 4.92 (d, *J* = 6.9 Hz, 1H), 4.56–4.40 (m, 2H), 4.40–4.18 (m, 3H), 4.08–3.84 (m, 4H), 3.61 (d, *J* = 9.2 Hz, 2H), 3.32–3.06 (m, 4H), 2.94–2.58 (m, 6H), 2.46 (s, 3H), 2.03 (d, *J* = 33.3 Hz, 6H), 1.83–1.64 (m, 3H), 1.39 (d, *J* = 7.0 Hz, 2H), 1.18 (t, *J* = 7.3 Hz, 4H), 0.97 (s, 12H). ^13^C NMR (101 MHz, DMSO-*d*_6_) δ 207.0, 168.8, 168.7, 168.7, 167.8, 167.4, 167.4, 163.4, 149.8, 146.1, 144.8, 143.0, 141.5, 135.9, 134.7, 133.4, 130.9, 129.5, 128.9, 128.1, 127.9, 127.3, 127.2, 127.1, 126.6, 126.5, 124.9, 124.7, 124.6, 124.1, 121.6, 116.4, 116.0, 114.1, 58.1, 53.2, 46.3, 46.0, 40.5, 34.0, 25.8, 24.7, 20.8, 20.7, 19.1, 14.4, 14.3, 12.4. HRMS-ESI (*m*/*z*): [M + H^+^]^+^ calculated for C_63_H_74_N_12_O_6_S_2_, 1159.5368; found, 1159.5360. HPLC retention time: 5.0 min, purity >95%.

#### (2*S*,4*R*)-1-((*S*)-2-(2-(4-((1-((1-(2-(4-(4-Amino-3-(1-(2-phenylacetyl)­indolin-5-yl)­thieno­[3,2-*c*]­pyridin-7-yl)-1*H*-pyrazol-1-yl)­acetyl)­piperidin-4-yl)­methyl)­piperidin-4-yl)­methyl)­piperazin-1-yl)­acetamido)-3,3-dimethylbutanoyl)-4-hydroxy-*N*-((*S*)-1-(4-(4-methylthiazol-5-yl)­phenyl)­ethyl)­pyrrolidine-2-carboxamide (**z0z-225-5**)

Linker part was prepared according to the literature procedure.^29^ ^1^H NMR (400 MHz, DMSO-*d*_6_) δ 8.98 (s, 1H), 8.44 (d, *J* = 7.7 Hz, 1H), 8.18 (d, *J* = 15.2 Hz, 4H), 8.09 (d, *J* = 13.8 Hz, 2H), 7.93 (d, *J* = 15.8 Hz, 2H), 7.72 (d, *J* = 9.5 Hz, 1H), 7.50–7.22 (m, 13H), 5.43 (s, 3H), 5.21 (d, *J* = 8.7 Hz, 3H), 4.95–4.83 (m, 1H), 4.52–4.40 (m, 2H), 4.32–4.18 (m, 4H), 3.88 (s, 3H), 3.58 (s, 2H), 3.23 (t, *J* = 8.6 Hz, 2H), 3.05 (d, *J* = 18.2 Hz, 4H), 2.91 (d, *J* = 16.7 Hz, 4H), 2.73 (s, 2H), 2.64 (s, 1H), 2.43 (d, *J* = 22.4 Hz, 10H), 2.11 (d, *J* = 43.6 Hz, 7H), 1.91–1.49 (m, 9H), 1.38 (d, *J* = 7.0 Hz, 3H), 1.21 (s, 4H), 0.93 (s, 12H). ^13^C NMR (101 MHz, DMSO-*d*_6_) δ 170.5, 169.4, 169.1, 168.5, 165.0, 163.7, 162.3, 153.2, 151.5, 147.8, 146.1, 144.8, 143.1, 139.4, 137.4, 136.3, 135.0, 132.5, 131.1, 130.8, 129.7, 129.6, 128.9, 128.8, 128.6, 128.3, 128.1, 126.6, 126.3, 125.8, 122.9, 118.6, 117.9, 115.7, 113.5, 68.8, 63.5, 62.8, 60.7, 58.5, 56.6, 55.7, 53.3, 53.0, 48.0, 47.8, 44.2, 42.1, 41.5, 37.8, 35.8, 32.0, 31.8, 30.8, 30.6, 29.9, 29.2, 27.5, 26.3, 22.5, 16.0. HRMS-ESI (*m*/*z*): [M + H^+^]^+^ calculated for C_69_H_85_N_13_O_6_S_2_, 1256.6260; found, 1256.6233. HPLC retention time: 4.9 min, purity >95%.

#### 1-(2-(2-(4-(4-Amino-3-(1-(2-phenylacetyl)­indolin-5-yl)­thieno­[3,2-*c*]­pyridin-7-yl)-1*H*-pyrazol-1-yl)­acetamido)­ethyl)-*N*-((*S*)-1-((2S,4*R*)-4-hydroxy-2-(((*S*)-1-(4-(4-methylthiazol-5-yl)­phenyl)­ethyl)­carbamoyl)­pyrrolidin-1-yl)-3,3-dimethyl-1-oxobutan-2-yl)­piperidine-4-carboxamide (**z0z-225-6**)

^1^H NMR (400 MHz, chloroform-*d*) δ 10.35 (s, 1H), 8.66 (s, 1H), 8.34 (d, *J* = 8.0 Hz, 2H), 8.13–7.83 (m, 3H), 7.74 (s, 1H), 7.44–7.11 (m, 10H), 6.88 (s, 2H), 5.15–5.03 (m, 1H), 4.93 (s, 1H), 4.68 (s, 1H), 4.50 (s, 1H), 4.21–3.95 (m, 2H), 3.85 (s, 1H), 3.65 (s, 1H), 3.45 (s, 1H), 3.20 (d, *J* = 10.2 Hz, 2H), 3.06 (s, 1H), 2.74 (s, 1H), 2.49 (s, 7H), 1.81 (d, *J* = 37.1 Hz, 3H), 1.45 (d, *J* = 6.8 Hz, 3H), 1.03 (s, 10H). ^13^C NMR (101 MHz, chloroform-*d*) δ 174.2, 171.7, 170.2, 169.6, 167.4, 151.9, 150.3, 149.5, 148.3, 143.9, 143.4, 138.8, 138.6, 133.9, 132.3, 131.6, 130.7, 129.7, 129.5, 129.1, 128.8, 128.8, 127.2, 126.4, 125.5, 124.5, 119.8, 117.7, 117.3, 114.0, 69.7, 58.9, 57.4, 57.3, 55.3, 55.1, 51.8, 51.5, 48.7, 48.4, 43.5, 36.5, 35.7, 35.1, 28.0, 27.7, 26.9, 26.6, 22.2, 16.1. HRMS-ESI (*m*/*z*): [M + H^+^]^+^ calculated for C_59_H_67_N_11_O_6_S_2_, 1090.4790; found, 1090.4750. HPLC retention time: 5.0 min, purity >95%.

#### 1-(3-(2-(4-(4-Amino-3-(1-(2-phenylacetyl)­indolin-5-yl)­thieno­[3,2-*c*]­pyridin-7-yl)-1*H*-pyrazol-1-yl)­acetamido)­propyl)-*N*-((*S*)-1-((2*S*,4*R*)-4-hydroxy-2-(((*S*)-1-(4-(4-methylthiazol-5-yl)­phenyl)­ethyl)­carbamoyl)­pyrrolidin-1-yl)-3,3-dimethyl-1-oxobutan-2-yl)­piperidine-4-carboxamide (**z0z-225-7**)

^1^H NMR (400 MHz, Acetone-*d*_6_) δ 8.84 (s, 1H), 8.36–8.26 (m, 2H), 8.00 (s, 2H), 7.80 (s, 1H), 7.37 (tq, *J* = 25.0, 7.5, 7.0 Hz, 14H), 6.82 (s, 1H), 5.17–4.96 (m, 4H), 4.67–4.56 (m, 2H), 4.53–4.46 (m, 1H), 4.31 (t, *J* = 8.6 Hz, 3H), 4.18 (dd, *J* = 7.4, 3.1 Hz, 1H), 3.99–3.67 (m, 9H), 3.50 (s, 3H), 3.35–3.22 (m, 4H), 3.12 (s, 2H), 2.44 (s, 3H), 2.31–2.20 (m, 1H), 2.17–2.10 (m, 3H), 1.44 (d, *J* = 6.9 Hz, 4H), 1.28 (s, 2H), 1.23 (tt, *J* = 7.2, 3.5 Hz, 1H), 1.15 (dd, *J* = 4.0, 2.1 Hz, 1H), 0.99 (d, *J* = 8.4 Hz, 11H). ^13^C NMR (101 MHz, scetone-*d*_6_) δ 209.2, 170.8, 169.6, 150.5, 148.2, 144.4, 144.3, 139.2, 138.1, 135.0, 133.5, 131.3, 130.5, 129.5, 129.1, 128.6, 128.4, 128.3, 127.6, 126.7, 126.6, 125.7, 116.8, 115.7, 115.3, 69.7, 68.4, 59.5, 56.7, 54.4, 54.0, 53.8, 53.6, 48.5, 48.4, 48.3, 42.6, 37.3, 35.5, 35.4, 26.1, 24.3, 21.7, 21.7, 15.5. HRMS-ESI (*m*/*z*): [M + H^+^]^+^ calculated for C_60_H_69_N_11_O_6_S_2_, 1104.4891; found, 1104.4915. HPLC retention time: 5.0 min, purity >95%.

#### (2*S*,4*R*)-1-((*S*)-2-(6-(4-(2-(4-(4-Amino-3-(1-(2-phenylacetyl)­indolin-5-yl)­thieno­[3,2-*c*]­pyridin-7-yl)-1*H*-pyrazol-1-yl)­acetyl)­piperazin-1-yl)­hexanamido)-3,3-dimethylbutanoyl)-4-hydroxy-*N*-((*S*)-1-(4-(4-methylthiazol-5-yl)­phenyl)­ethyl)­pyrrolidine-2-carboxamide (**z0z-229-1**)

^1^H NMR (400 MHz, Methanol-*d*_4_) δ 8.82 (d, *J* = 9.5 Hz, 1H), 8.20 (d, *J* = 24.6 Hz, 4H), 8.09 (d, *J* = 14.6 Hz, 1H), 7.87 (s, 1H), 7.77 (s, 1H), 7.44 (d, *J* = 5.8 Hz, 1H), 7.41–7.34 (m, 4H), 7.31 (d, *J* = 6.8 Hz, 4H), 7.24 (td, *J* = 6.1, 2.4 Hz, 1H), 7.19 (d, *J* = 8.1 Hz, 1H), 7.10 (d, *J* = 8.4 Hz, 1H), 5.31 (s, 2H), 4.98 (q, *J* = 6.8 Hz, 2H), 4.68–4.49 (m, 2H), 4.43 (s, 1H), 4.17 (t, *J* = 8.5 Hz, 2H), 3.87 (d, *J* = 10.4 Hz, 6H), 3.77–3.67 (m, 1H), 3.35 (s, 2H), 3.14 (d, *J* = 31.5 Hz, 6H), 2.97 (d, *J* = 10.7 Hz, 2H), 2.43 (d, *J* = 2.4 Hz, 3H), 2.36–2.15 (m, 3H), 1.94 (td, *J* = 9.0, 4.6 Hz, 1H), 1.81–1.59 (m, 4H), 1.51–1.43 (m, 3H), 1.38 (d, *J* = 7.5 Hz, 2H), 1.02 (d, *J* = 6.7 Hz, 9H). ^13^C NMR (101 MHz, methanol-*d*_4_) δ 174.2, 171.8, 170.9, 170.7, 166.2, 165.0, 151.5, 150.5, 149.9, 147.6, 144.2, 143.5, 138.5, 137.3, 134.4, 133.3, 131.9, 130.0, 129.1, 129.0, 128.4, 126.7, 126.2, 126.0, 125.8, 125.5, 119.2, 116.7, 114.4, 69.6, 59.2, 57.7, 56.8, 56.7, 51.5, 51.2, 48.7, 48.5, 42.5, 39.6, 37.5, 36.2, 35.1, 34.7, 27.4, 25.9, 25.7, 24.8, 23.6, 21.1, 14.5. HRMS-ESI (*m*/*z*): [M + H^+^]^+^ calculated for C_61_H_71_N_11_O_6_S_2_, 1118.5103; found, 1118.5076. HPLC retention time: 5.1 min, purity >95%.

#### (2*S*,4*R*)-1-((*S*)-2-(5-((1-(2-(4-(4-Amino-3-(1-(2-phenylacetyl)­indolin-5-yl)­thieno­[3,2-*c*]­pyridin-7-yl)-1*H*-pyrazol-1-yl)­acetyl)­piperidin-4-yl)­amino)­pentanamido)-3,3-dimethylbutanoyl)-4-hydroxy-*N*-((*S*)-1-(4-(4-methylthiazol-5-yl)­phenyl)­ethyl)­pyrrolidine-2-carboxamide (**z0z-229-2**)

^1^H NMR (500 MHz, chloroform-*d*) δ 8.67 (d, *J* = 2.8 Hz, 1H), 8.53 (s, 1H), 8.36 (d, *J* = 8.2 Hz, 1H), 7.99–7.86 (m, 3H), 7.82 (s, 1H), 7.43–7.29 (m, 12H), 7.27–7.21 (m, 2H), 7.17 (s, 1H), 6.09 (s, 2H), 5.35–5.02 (m, 3H), 4.78–4.57 (m, 3H), 4.48 (s, 1H), 4.17 (t, *J* = 8.7 Hz, 2H), 4.04 (d, *J* = 11.0 Hz, 2H), 3.88 (s, 2H), 3.66 (d, *J* = 10.5 Hz, 1H), 3.28–3.09 (m, 4H), 2.89 (s, 2H), 2.69 (s, 1H), 2.52 (d, *J* = 4.2 Hz, 3H), 2.35–2.07 (m, 6H), 1.65 (d, *J* = 32.6 Hz, 6H), 1.48 (d, *J* = 6.9 Hz, 3H), 1.07 (s, 10H). ^13^C NMR (126 MHz, chloroform-*d*) δ 173.1, 171.8, 171.6, 170.3, 169.5, 169.1, 165.2, 165.2, 152.3, 150.3, 148.8, 148.7, 148.4, 148.4, 143.7, 143.6, 143.5, 138.3, 137.6, 137.6, 133.9, 132.1, 131.6, 130.7, 130.6, 130.4, 129.4, 129.1, 128.8, 128.8, 127.2, 126.4, 125.6, 123.8, 119.6, 118.3, 118.2, 117.2, 114.2, 69.8, 59.0, 57.9, 57.9, 57.5, 54.6, 53.4, 48.7, 48.4, 44.2, 44.1, 43.6, 43.5, 40.8, 37.1, 37.0, 35.5, 35.5, 34.7, 28.0, 26.6, 22.4, 22.3, 16.1. HRMS-ESI (*m*/*z*): [M + H^+^]^+^ calculated for C_61_H_71_N_11_O_6_S_2_, 1118.5103; found, 1118.5069. HPLC retention time: 4.9 min, purity >95%.

#### (2*S*,4*R*)-1-((*S*)-2-(3-(4-((2-(4-(4-Amino-3-(1-(2-phenylacetyl)­indolin-5-yl)­thieno­[3,2-*c*]­pyridin-7-yl)-1*H*-pyrazol-1-yl)­acetyl)­glycyl)­piperazin-1-yl)­propanamido)-3,3-dimethylbutanoyl)-4-hydroxy-*N*-((*S*)-1-(4-(4-methylthiazol-5-yl)­phenyl)­ethyl)­pyrrolidine-2-carboxamide (**z0z-229-3**)

^1^H NMR (400 MHz, chloroform-*d*) δ 8.60 (s, 1H), 8.55 (d, *J* = 8.1 Hz, 1H), 8.29 (d, *J* = 8.2 Hz, 1H), 7.97 (s, 1H), 7.92 (s, 1H), 7.84 (s, 1H), 7.43 (d, *J* = 7.7 Hz, 1H), 7.37–7.25 (m, 9H), 7.25–7.21 (m, 2H), 7.09 (s, 1H), 5.18–4.94 (m, 4H), 4.94–4.80 (m, 2H), 4.66 (t, *J* = 7.9 Hz, 1H), 4.41 (d, *J* = 8.3 Hz, 2H), 4.16–4.03 (m, 4H), 3.96 (dd, *J* = 17.0, 4.0 Hz, 1H), 3.79 (s, 3H), 3.50 (dd, *J* = 11.4, 3.6 Hz, 2H), 3.42 (d, *J* = 0.7 Hz, 6H), 3.17 (t, *J* = 8.5 Hz, 3H), 2.63 (t, *J* = 5.9 Hz, 3H), 2.50–2.25 (m, 11H), 2.01 (dd, *J* = 13.6, 8.2 Hz, 1H), 1.40 (d, *J* = 6.9 Hz, 3H), 0.99 (s, 9H). ^13^C NMR (101 MHz, chloroform-*d*) δ 172.8, 172.1, 169.7, 166.9, 165.9, 152.9, 150.3, 148.5, 143.5, 143.2, 139.2, 133.9, 131.9, 131.6, 130.9, 129.5, 129.1, 128.9, 128.5, 127.2, 126.4, 125.7, 122.9, 119.6, 119.4, 117.1, 114.0, 77.2, 70.1, 58.0, 56.6, 55.1, 53.6, 52.1, 52.0, 50.9, 48.8, 48.4, 44.4, 43.6, 42.0, 41.4, 35.5, 34.6, 31.8, 28.0, 26.7, 22.2, 16.1. HRMS-ESI (*m*/*z*): [M+H^+^]^+^ calculated for C_60_H_68_N_12_O_7_S_2_, 1133.4848; found, 1133.4820. HPLC retention time: 5.0 min, purity >95%.

#### (2*S*,4*R*)-1-((*S*)-2-(3-(4-(2-(2-(4-(4-Amino-3-(1-(2-phenylacetyl)­indolin-5-yl)­thieno­[3,2-*c*]­pyridin-7-yl)-1*H*-pyrazol-1-yl)­acetamido)­ethyl)­piperazin-1-yl)­propanamido)-3,3-dimethylbutanoyl)-4-hydroxy-*N*-((*S*)-1-(4-(4-methylthiazol-5-yl)­phenyl)­ethyl)­pyrrolidine-2-carboxamide (**z0z-229-4**)

^1^H NMR (400 MHz, methanol-*d*_4_/chloroform-*d*(V/V = 3:1)) δ 8.77 (d, *J* = 6.5 Hz, 1H), 8.21 (q, *J* = 7.8, 6.3 Hz, 2H), 7.97 (d, *J* = 4.3 Hz, 1H), 7.71 (d, *J* = 14.2 Hz, 1H), 7.61 (s, 1H), 7.38 (s, 4H), 7.31 (d, *J* = 4.6 Hz, 5H), 7.27–7.16 (m, 2H), 5.00 (d, *J* = 10.2 Hz, 3H), 4.63–4.54 (m, 2H), 4.46 (d, *J* = 9.7 Hz, 1H), 4.22 (t, *J* = 8.5 Hz, 2H), 3.90 (d, *J* = 10.6 Hz, 3H), 3.72 (dd, *J* = 11.1, 3.8 Hz, 1H), 3.62–3.47 (m, 2H), 3.37 (s, 1H), 3.25 (s, 3H), 3.19–2.82 (m, 9H), 2.62 (d, *J* = 7.8 Hz, 2H), 2.45 (s, 3H), 2.24 (dd, *J* = 13.3, 7.9 Hz, 1H), 1.99 (td, *J* = 9.1, 8.6, 4.8 Hz, 1H), 1.59 (d, *J* = 7.0 Hz, 1H), 1.48 (d, *J* = 7.0 Hz, 3H), 1.03 (d, *J* = 7.9 Hz, 9H). ^13^C NMR (101 MHz, methanol-*d*_4_/chloroform-*d*(V/V = 3:1)) δ 171.6, 171.0, 170.9, 168.7, 163.4, 151.8, 151.3, 149.0, 147.6, 144.0, 143.9, 138.9, 138.1, 134.1, 133.8, 132.0, 130.5, 130.1, 130.1, 129.3, 129.2, 129.2, 128.8, 128.5, 128.1, 127.5, 126.8, 126.3, 125.8, 125.6, 125.0, 119.6, 117.1, 115.7, 115.6, 115.1, 69.6, 67.7, 59.2, 58.0, 56.7, 55.8, 54.0, 52.7, 42.6, 37.4, 35.9, 35.2, 27.5, 26.1, 25.9, 25.8, 21.7, 21.4, 15.0, 14.9. HRMS-ESI (*m*/*z*): [M+H^+^] ^+^ calculated for C_60_H_70_N_12_O_6_S_2_, 1119.5061; found, 1119.5020. HPLC retention time: 5.0 min, purity >95%.

##### Cell Culture

B16F10, A375 and 293T-RIPK1-HiBiT cells were cultured in DMEM medium (Corning), supplemented with 10% fetal bovine serum (FBS) and 1% penicillin–streptomycin. All cells were incubated at 37 °C in a 5% CO_2_ incubator.

##### Western Blot Assay

Western blotting was performed as previously described.^25c^ Cells were lysed in RIPA buffer, and protein concentrations were determined using the BCA assay. Equal amounts of protein were separated by SDS-PAGE, transferred to PVDF membranes, and probed with antibodies against Brd4 and β-Actin. Bound antibodies were visualized using the ECL assay (Bio-Rad), and images were captured using the Chemidoc MP imaging system (Bio-Rad). Antibodies were purchased from Cell Signaling Technology, including Anti-RIPK1 (CS#3493), Anti-β-Actin (CS#3700), and HRP-conjugated antirabbit IgG (CS#7074).

##### Generation of HEK293T-RIPK1-HiBiT Cell Line

HiBiT is a 1.3 kDa peptide (11 amino acids) capable of producing bright and quantitative luminescence through high affinity complementation with an 18 kDa subunit derived from NanoLuc (LgBiT). HiBiT tag was fused to the C-terminal of RIPK1 of HEK293T cells using the CRISPR-Cas9 system.^26^

Guide RNA oligos for RIPK1 (foward: CACCGA­GCCCAT­CCAGGG­TTAGTTC. reverse: AAACGA­ACTAAC­CCTGGA­TGGGCTC) were cloned into Cas9 expression plasmid (pSpCas9–2A-GFP purchased from Addgene), and cotransfected with donor DNA oligo using FuGENE HD Transfection Reagent (Promega). GFP positive cells were FACS sorted and single clones were screened for HiBiT signal by Nano Glo HiBiT Lytic Detection System (Promega).

### Animal Model and Treatments

All animal work were approved by the University of Wisconsin-Madison Institutional Animal Care and Use Committee (IACUC) and conducted in accordance with the NIH Guide for the care and use of laboratory animals (animal protocol number M006790-A2). C57BL/6 and NSG mice were purchased from the Jackson Laboratory and breed in BRMS Animal Facilities in UW-Madison. Cells were inoculated into the flanks of mice aged 6 to 10 weeks. Once tumors reached to about 100–200 mm^3^ in size. Mice were randomized into treatment groups and were injected intraperitoneally with vehicle, degrader, X-ray radiation (XRAD320) or degrader combing with radiation. Mice were monitored daily and weighted three times a week. Tumor volume was estimated by measuring the length and width of a tumor using calipers and then inputting the values in the equation *V* = 0.5 × *L* × *W*2. For detecting RIPK1 degradation induced by 225-5, tumor-bearing mice were treated with 225-5 for 3 days. Tumor tissues were collected at day 4, and RIPK1 expression level in tumors was analyzed by Western blot assay. Tumor growth delay was graphed using GraphPad Prism.

### PBMC-Humanized Mice

To establish humanized mouse models, 5–10 week-old NSG mice were injected with 1 × 10^7^ human peripheral blood mononuclear cells (PBMCs) intravenously 1–7 days after cancer cells inoculation. The engraftment levels of human CD45+ cells and human immune cell populations in the peripheral blood, and spleen tissue was determined using flow cytometry.

### Sample Preparation for Global Proteomics Analysis

#### Chemicals and Materials

Optima UPLC-grade acetonitrile (ACN), Optima UPLC grade water, Optima LC/MS grade formic acid (FA), calcium chloride dihydrate (CaCl_2_•2H_2_O), Urea, Tris base, and hydrochloric acid (HCl) were obtained from Fisher Scientific (Hampton, NH). Iodoacetamide (IAA), trifluoroacetic acid (TFA), and dithiothreitol (DTT) were purchased from Sigma-Aldrich (St. Louis, MO). EDTA-free Protease Inhibitor Cocktail and Phosphatase Inhibitor Cocktail tablets were acquired from Roche (Basel, Switzerland). Pierce BCA protein assay kit and quantitative colorimetric peptide assay were obtained from Thermo Fisher Scientific (Waltham, MA). Mass spectrometry grade Trypsin/Lys-C were purchased from Promega (Madison, WI). Sep-Pak C18 cartridges and Bridged Ethylene Hybrid (BEH) C18 particles were purchased from Waters Corporation (Milford, MA).

#### Protein Extraction and in-Solution Digestion

Two types of cell samples (A375 and MDA-MB-231) were processed with the 12-plex DiLeu scheme, respectively. In each 12-plex DiLeu set, four groups of cell samples (6± h and 24± h) with three biological replicates for each were processed with the following procedure: Each cell sample was first dissolved in 300 μL extraction solution (8 M urea, 50 mM Tris buffer with a pH value of 8 containing 5 mM CaCl_2_, 20 mM NaCl, EDTA-free protease inhibitor, and phosphatase inhibitor) and sonicated on ice using a probe sonicator (Thermo Fisher Scientific, AMPL 50%, and PULSE: 15 s with a 5 s pause for 3 min). After the sonication, each sample was centrifuged at 14,000*g* for 10 min at 4 °C, and the supernatant was transferred to a new tube for further protein digestion. The supernatant of each sample was measured by protein bicinchoninic acid (BCA) assay to acquire the protein concentration. A 300 μg protein of each sample was aliquoted, reduced with 5 mM dithiothreitol (DTT) at 37 °C for 30 min, alkylated with 15 mM iodoacetamide (IAA) in the dark for 45 min, and quenched with 5 mM DTT for 10 min. Samples were diluted with 50 mM Tris buffer to a urea concentration <1M. In solution protein digestion was performed with LysC/trypsin (Promega) in a 50:1 ratio (protein: enzyme; w/w) at 37 °C overnight. The digestion solutions were quenched with 10% trifluoroacetic acid (TFA) to reach a final concentration of 1% TFA. Peptides were desalted with Sep-Pak C18 cartridges, dried in vacuo.

#### 12-plex DiLeu Isobaric Tag Labeling

A 100 μg peptides of each sample was aliquoted and reconstituted in 20 μL 0.5 M TEAB. One mg of each 12-plex DiLeu tags was activated by adding and shaking in activation solution for 40 min as reported previously.^30,31^ After activation, peptide aliquots were combined and shaken with each channel of DiLeu tag respectively for another 2 h. Then 5% hydroxylamine were added into each tube to make the final concentration of hydroxylamine 0.25% to quench the reaction. Equal amount of labeled peptides were combined, dried in vacuo, and cleaned with SCX SpinTip (TT200SEA tip, PolyLC containing 12 mg Polysulfoethyl A beads, 20-m, 300-A) by following the manufacturer’s protocol.

#### High-pH Fractionation

The samples were reconstituted in water with 10 mM ammonium formate at pH = 10 for HpH RPLC fractionation. The HpH RPLC fractionation was performed with the following procedure: HpH fractionation was performed on a Waters Alliance e2695 HPLC using a C18 column (Phenomenex, 150 mm × 2.1 mm, 5 mm, 100 Å) at a 0.2 mL/min flow rate. Mobile phase A consisted of 10 mM ammonium formate in water, and mobile phase B consisted of 10 mM ammonium formate in 90% ACN, which both mobile phases were adjusted to pH 10. Separation was performed with the following gradient: 1% B (0–5 min), 1–40% B (5–50 min), 40–60% B (50–54 min), 60–70% B (54–58 min), and 70–100% B (58–59 min) operating at 0.2 mL/min. Fractions were collected every 2 min from the seventh minute (7–65 min) and total of 29 fractions were collected. Nonadjacent fractions were concatenated into 6 tubes for global proteomic analysis.

#### LC–MS/MS Data Acquisition

Before the mass spectrometry analysis, the peptide concentrations were measured by nanoDrop. One μg peptides of each sample were separated on a self-fabricated microcapillary column packed with C18 beads (Waters Bridged Ethylene Hybrid, 1.7 μm, 130Å, 75 μm × 15 cm). All the LC-MS experiments were performed on Thermo Scientific Q Exactive HF mass spectrometer interfaced with a Dionex Ultimate 3000 UPLC system. Mobile phase A was composed of optima grade water with 0.1% FA, while mobile phase B was composed of 80% ACN with 0.1% FA. LC separation was achieved via a 155 min gradient at a flow rate of 300 nL/min: 0–18.33 min, 4%B; 18.33–120.0 min, 4–40%B; 120.0–120.5 min, 40–75%B; 120.5–130.0 min, 75–75%B; 130.0–130.5 min, 75–97.0%B; 130.5–140.0 min, 97.0%B; 140.0–140.5 min, 97.0–4.0%B; 140.5–155.0 min, 4.0%B. Survey scans of peptide precursors from 300 to 1500 *m*/*z* were performed at a resolving power of 60k (at *m*/*z* 200) with an AGC target of 1E6 and maximum injection time of 100 ms. The top 10 precursors were then selected for higher energy collisional dissociation fragmentation with a normalized collision energy of 30, an isolation width of 1.0 Da, a resolving power of 60k, an AGC target of 1E5, a maximum injection time of 200 ms, and a lower mass limit of 110 *m*/*z*. Precursors were subject to dynamic exclusion for 30 s with a 10 ppm tolerance. Each sample was acquired in technical duplicates.

#### Data Analysis

Protein identification and quantification of mass spectrometry (MS) data were conducted using Proteome Discoverer (version 2.5, Thermo Scientific). All raw files were searched against the Uniprot *Homo sapiens* reviewed database (July 22, 2024) using Sequest HT with trypsin/Lys-C selected as the enzyme and two missed cleavages allowed. DiLeu labeling on peptide N termini and lysine residues (+145.12801), and carbamidomethylation of cysteine residues (+57.02146 Da) were chosen as fixed modifications. Variable modifications included oxidation of methionine residues (+15.99492 Da). Peptide spectral matches (PSMs) were validated on the basis of q-values to 1% FDR using prelocator. Quantification of reporter ions in MS2 spectra were performed in PD using an integration tolerance of 20 ppm for the most confident centroid. Reporter ion intensities for PSMs were exported and analyzed in R studio.

## Supplementary Material





## Notes

RIPK1 is expressed in a variety of cancer cell types; screening of initial set of RIPK1 degraders in NOMO-1 cells; PK study of 204-2 and 225-5; NMR spectra, HPLC analyses for compounds reported in this study, and HRMS data of 204-2, 216-16, 225-5 (PDF)
Molecular formula strings and biological activity of RIPK1 PROTAC (CSV)

## Abbreviations Used

RIPK1: receptor-interacting serine/threonine-protein kinase 1
PK: pharmacokinetics
TLC: thin layer chromatography
