# Supplementary material for: Development of Potent and Selective RIPK1 Degraders Targeting Its Nonenzymatic Function for Cancer Treatment
Source: J Med Chem. 2025 Jul 16;68(14):15120–36. doi: 10.1021/acs.jmedchem.5c01340 (PMC12283027; doi:10.1021/acs.jmedchem.5c01340)
Supplement: Supplementary file 1 [file jm5c01340_si_001.pdf]

Supporting Information

Development of Potent and Selective RIPK1 Degraders Targeting Its Non-Enzymatic Function for Cancer Treatment

Zhen Zhang,[1#] Chunrong Li,[1#] Nina J. Hawkins,[1#] Ramesh Mudududdla[1], Yiming Nie[1], Peng-Kai Liu[2], Penghsuan Huang[3], Natalia M. Del Rio[4], Hao Chang[5], Matthew E. Brown[4], Lingjun Li[1,2,3], Weiping Tang[1,3]*

[1] Lachman Institute for Pharmaceutical Development, School of Pharmacy, University of Wisconsin−Madison, Madison, Wisconsin 53705, United States.

[2] Biophysics Graduate Program, University of Wisconsin–Madison, Madison, Wisconsin 53705, United States.

[3] Department of Chemistry, University of Wisconsin–Madison, Madison, Wisconsin 53706, United States.

[4] Department of Surgery, School of Medicine and Public Health, University of Wisconsin−Madison, Madison, Wisconsin 53792, United States.

[5] Department of Dermatology, School of Medicine and Public Health, University of Wisconsin−Madison, Madison, Wisconsin 53705, United States.

[#] Z.Z., C.L. and N.J.H. contributed equally to this work.

* Correspondence: Email: weiping.tang@wisc.edu; orcid.org/0000-0002-0039-3196.

Table of Contents

SUPPORTING INFORMATION....1

FIGURE S1. RIPK1 IS EXPRESSED IN A VARIETY OF CANCER CELL TYPES. 3

FIGURE S2. SCREENING OF DEGRADERS IN NOMO-1 CELLS. 3

FIGURE S3. PK STUDY OF 204-2 AND 225-5. 3

NMR SPECTRUM 4

HPLC DATA 42

HRMS SPECTRUM OF 204-2, 216-16, 225-5....79

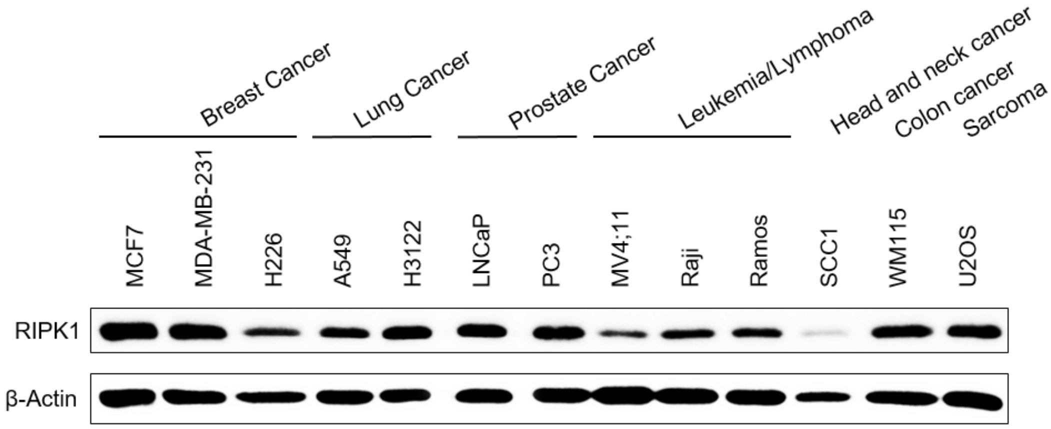

Figure S1. RIPK1 is expressed in a variety of cancer cell types.

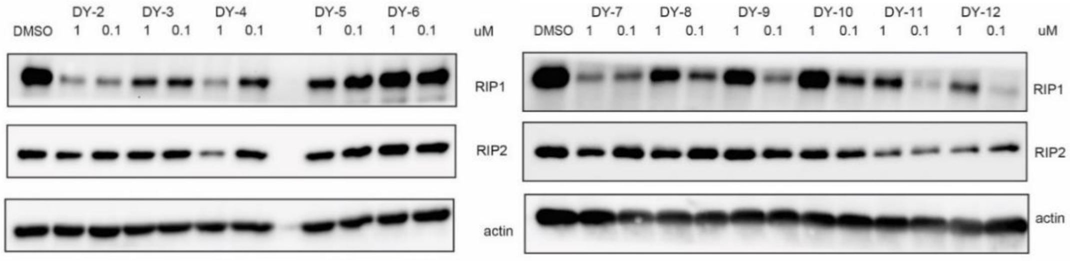

Figure S2. Screening of initial set of RIPK1 degraders in NOMO-1 cells.

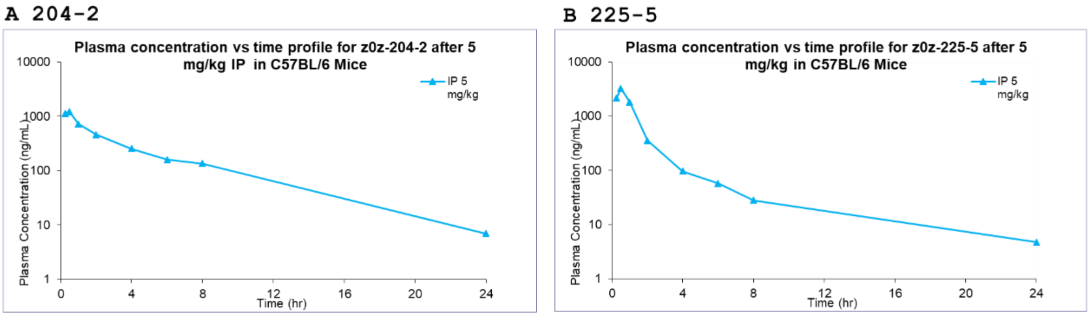

IP Dose	5	mg/kg	
PK parameters		Unit	IP
$T_{1/2}$		h	3.88
T_{max}		h	0.5
C_{max}		ng/mL	1210
AUC_{last}		h*ng/mL	4074
AUC_{Inf}		h*ng/mL	4113
$AUC__{\%Extrap}$_obs		%	0.940
MRT_{Inf}_obs		h	4.46
AUC_{last}/D		h*mg/mL	815
F		%	NA

IP Dose	5	mg/kg	
PK parameters		Unit	IP
$T_{1/2}$		h	5.41
T_{max}		h	0.5
C_{max}		ng/mL	3270
AUC_{last}		h*ng/mL	4279
AUC_{Inf}		h*ng/mL	4316
$AUC__{\%Extrap}$_obs		%	0.859
MRT_{Inf}_obs		h	2.03
AUC_{last}/D		h*mg/mL	856
F		%	NA

Figure S3. PK study of 204-2 and 225-5.

NMR Spectrum

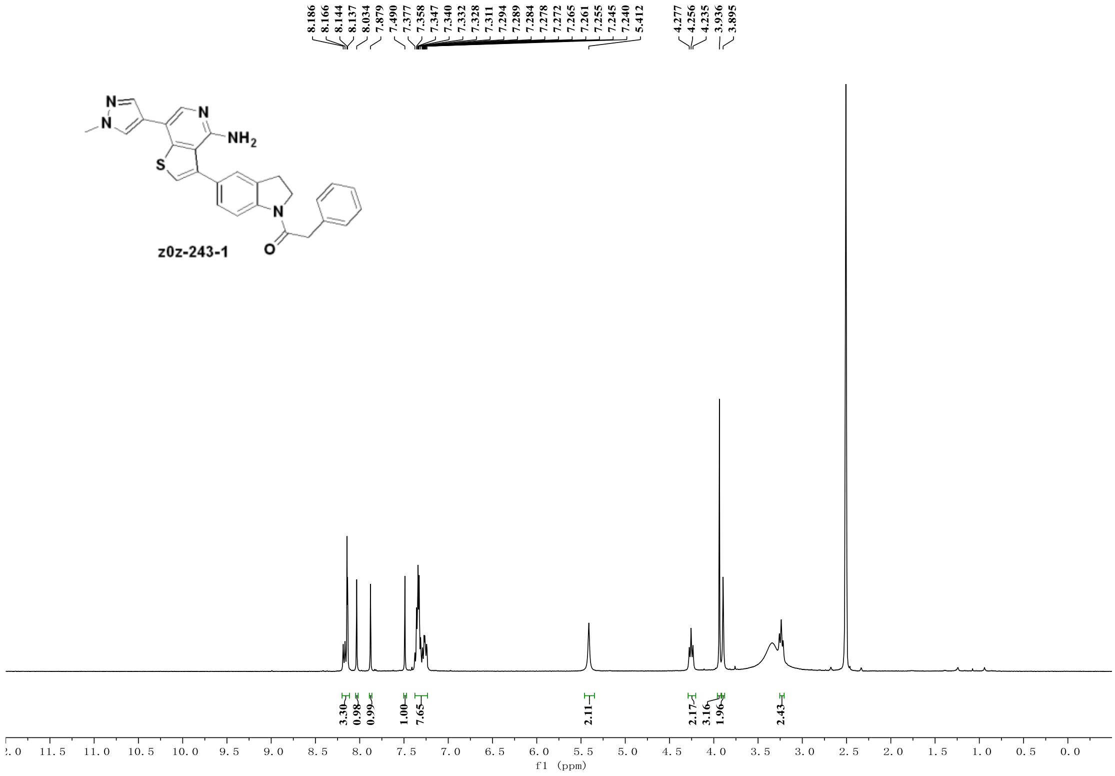

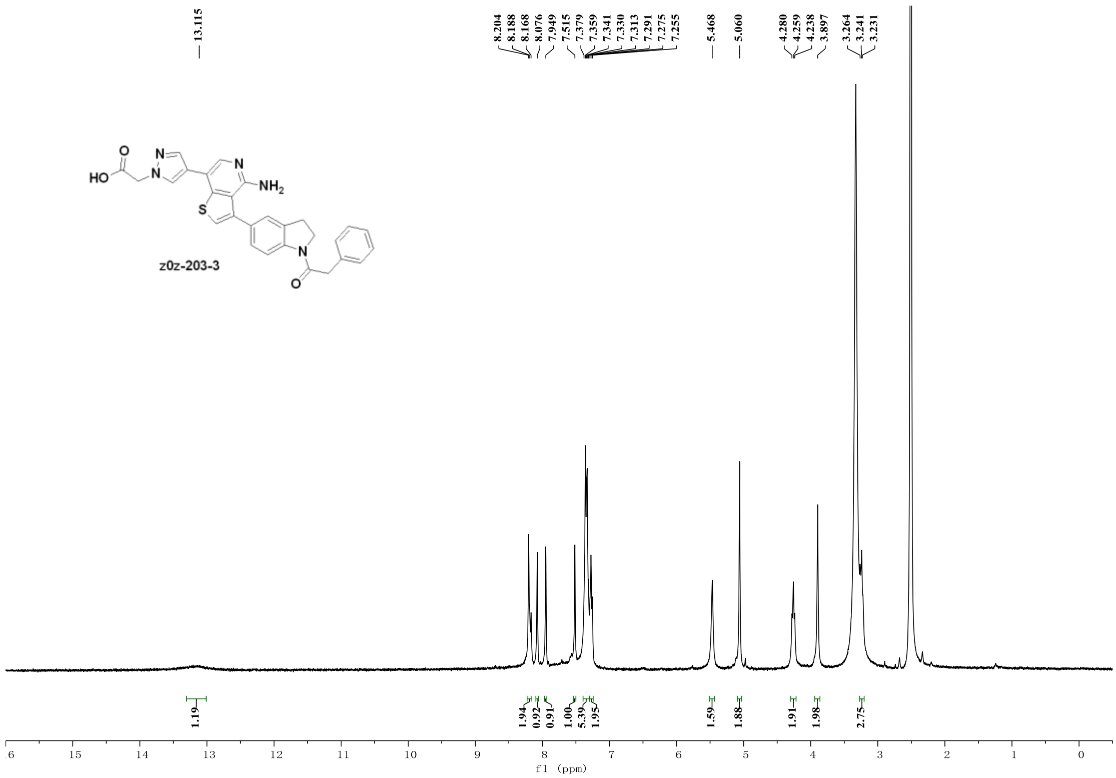

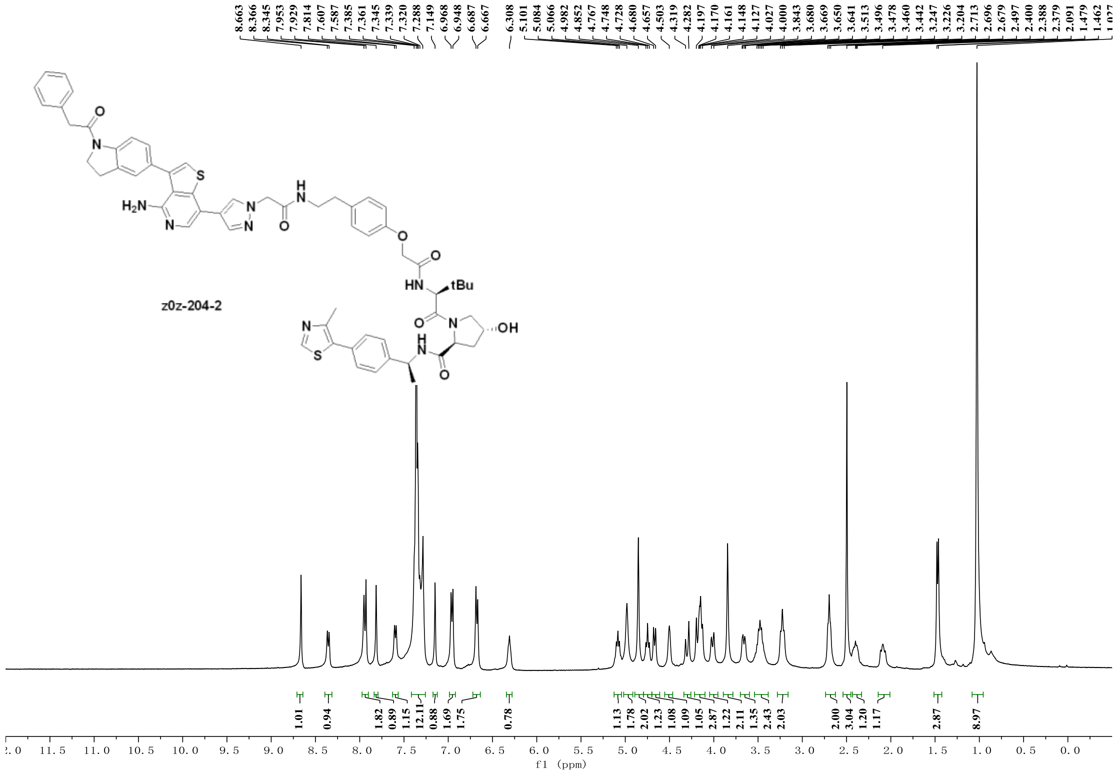
8.663 8.366 8.345 7.953 7.929 7.814 7.607 7.587 7.385 7.361 7.345 7.339 7.320 7.288 7.149 6.968 6.948 6.687 6.667 6.308 5.101 5.084 5.066 4.982 4.852 4.767 4.748 4.728 4.680 4.657 4.503 4.319 4.282 4.197 4.170 4.161 4.148 4.127 4.027 4.000 3.843 3.680 3.669 3.650 3.641 3.513 3.496 3.478 3.460 3.442 3.247 3.226 3.204 2.713 2.696 2.679 2.497 2.400 2.388 2.379 2.091 1.479 1.462
z0z-204-2
H2N
tBu
OH
1.01 0.94 1.82 0.89 1.15 12.11 0.88 1.69 1.75 0.78 1.13 1.78 2.02 1.23 1.08 1.09 1.05 2.87 1.22 2.11 1.35 2.43 2.03 2.00 3.04 1.20 1.17 2.87 8.97
f1 (ppm)

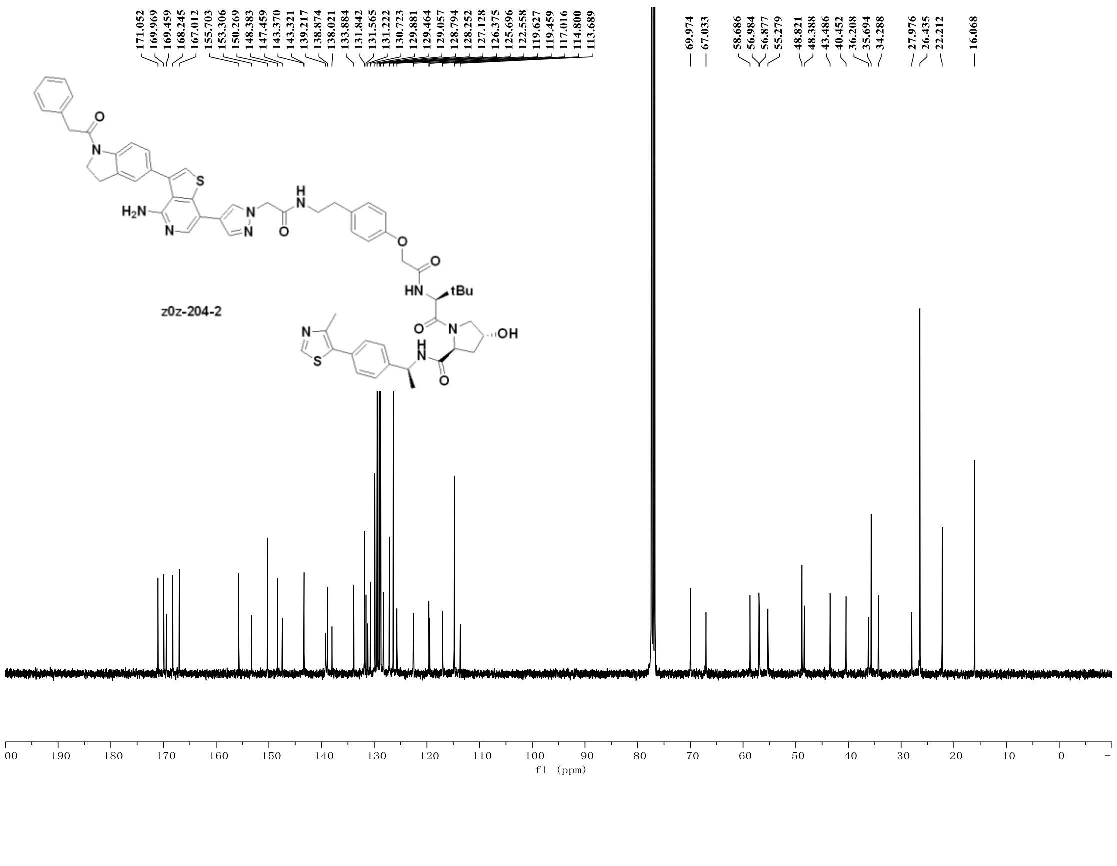
171.052 169.969 169.459 168.245 167.012 155.703 153.306 150.269 148.383 147.459 143.370 143.321 139.217 138.874 138.021 133.884 131.842 131.565 131.222 130.723 129.881 129.464 129.057 128.794 128.252 127.128 126.375 125.696 122.558 119.627 119.459 117.016 114.800 113.689
69.974 67.033 58.686 56.984 56.877 55.279 48.821 48.388 43.486 40.452 36.208 35.694 34.288 27.976 26.435 22.212 16.068
z0z-204-2
H2N
tBu
OH
f1 (ppm)

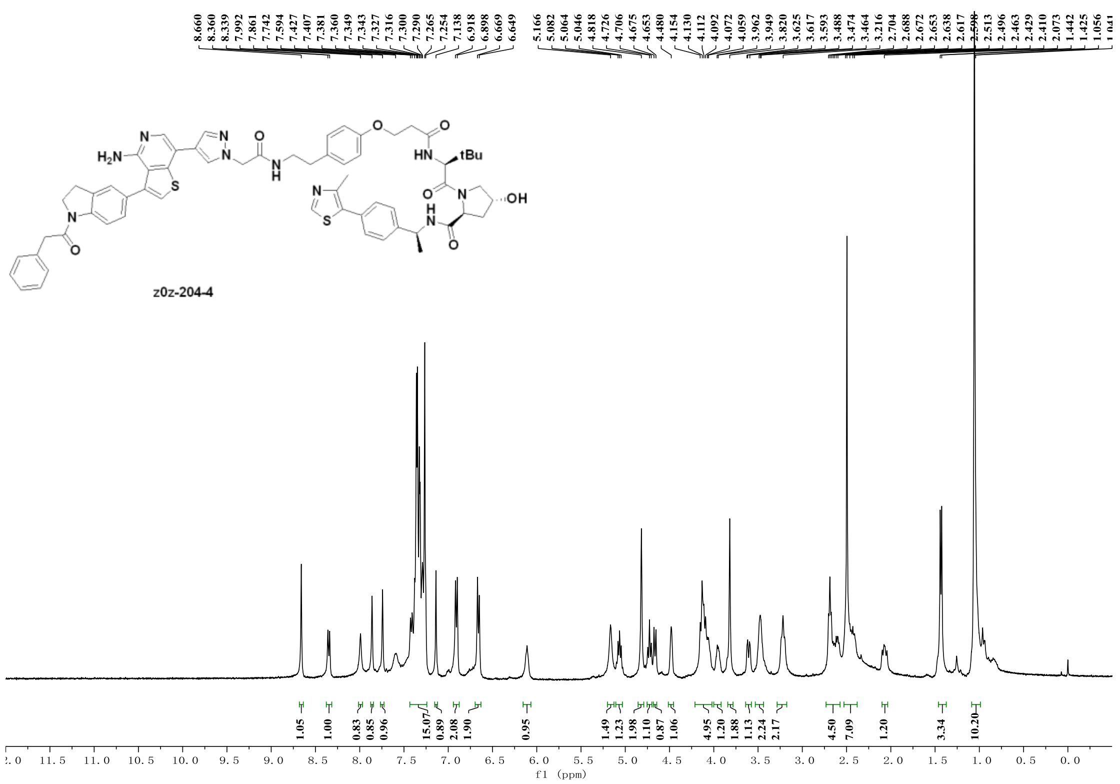
z0z-204-4
f1 (ppm)

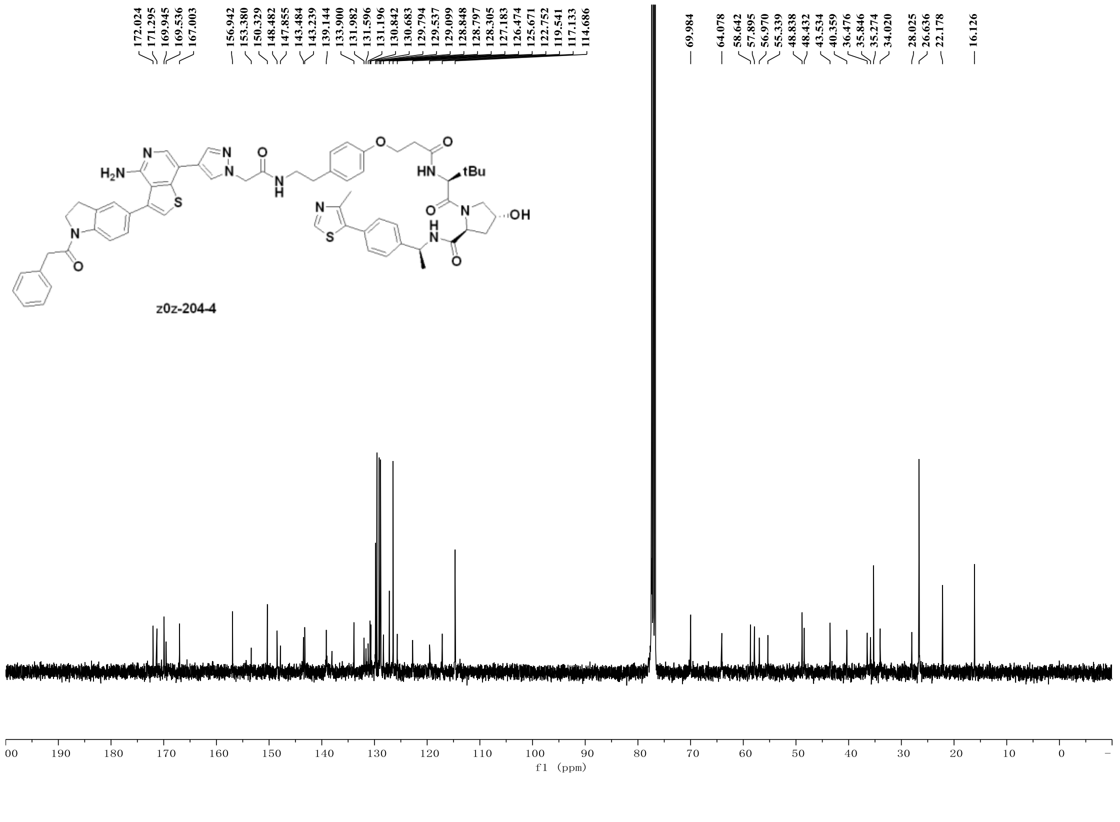
z0z-204-4
f1 (ppm)

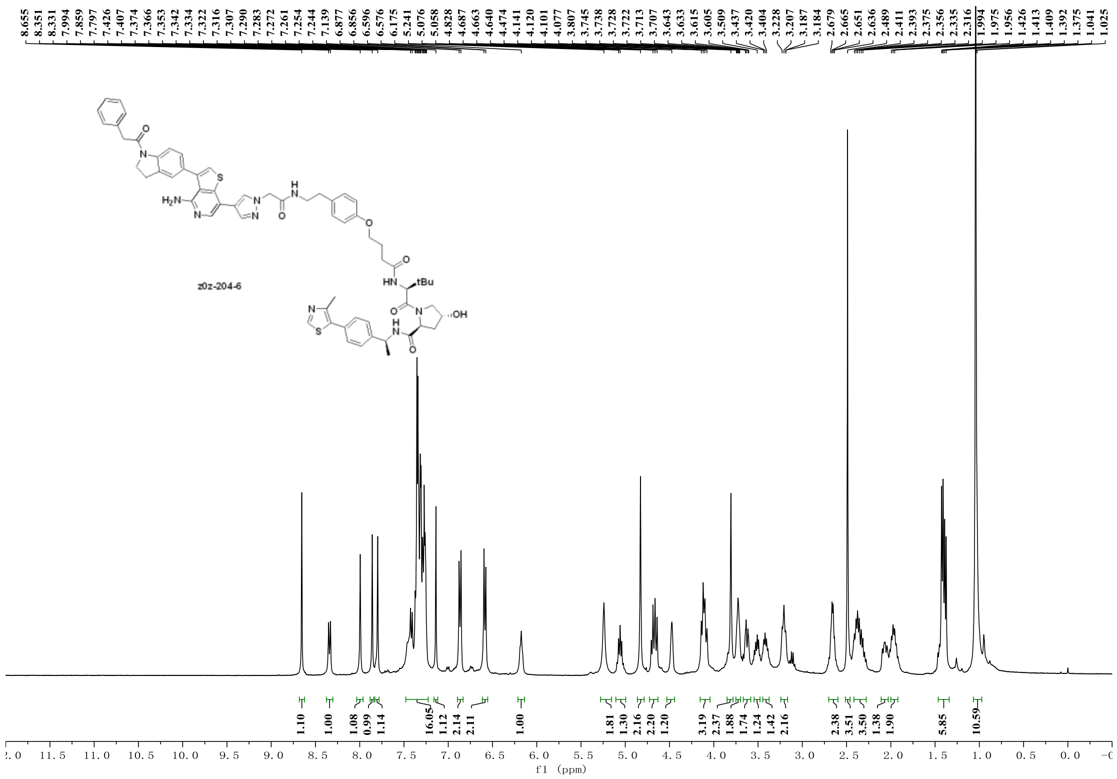
8.655
8.351
8.331
7.994
7.859
7.797
7.426
7.407
7.374
7.366
7.353
7.342
7.334
7.322
7.316
7.307
7.290
7.283
7.272
7.261
7.254
7.244
7.139
6.877
6.856
6.596
6.576
6.175
5.241
5.076
5.058
4.828
4.687
4.663
4.640
4.474
4.141
4.120
4.101
4.077
3.807
3.745
3.738
3.728
3.722
3.713
3.707
3.643
3.633
3.615
3.605
3.509
3.437
3.420
3.404
3.228
3.207
3.187
3.184
2.679
2.665
2.651
2.636
2.489
2.411
2.393
2.375
2.356
2.335
2.316
1.994
1.975
1.956
1.426
1.413
1.409
1.392
1.375
1.041
1.025
H2N
N
S
N
O
H
N
O
O
HN
tBu
N
OH
N
S
H
N
O
z0z-204-6
1.10
1.00
1.08
0.99
1.14
16.05
1.12
2.14
2.11
1.00
1.81
1.30
2.16
2.20
1.20
3.19
2.37
1.88
1.74
1.24
1.42
2.16
2.38
3.51
3.50
1.38
1.90
5.85
10.59
f1 (ppm)

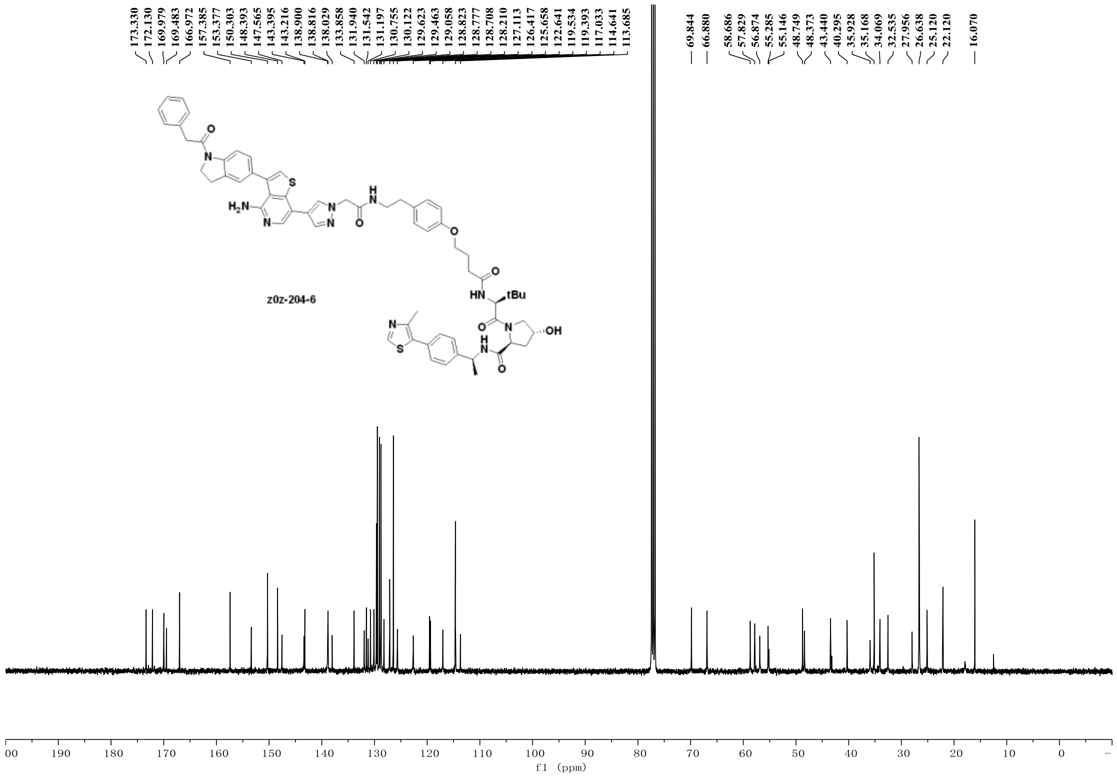
173.330
172.130
169.979
169.483
166.972
157.385
153.377
150.303
148.393
147.565
143.395
143.216
138.900
138.816
138.029
133.858
131.940
131.542
131.197
130.755
130.122
129.623
129.463
129.058
128.823
128.777
128.708
128.210
127.113
126.417
125.658
122.641
119.534
119.393
117.033
114.641
113.685
69.844
66.880
58.686
57.829
56.874
55.285
55.146
48.749
48.373
43.440
40.295
35.928
35.168
34.069
32.535
27.956
26.638
25.120
22.120
16.070
z0z-204-6
f1 (ppm)

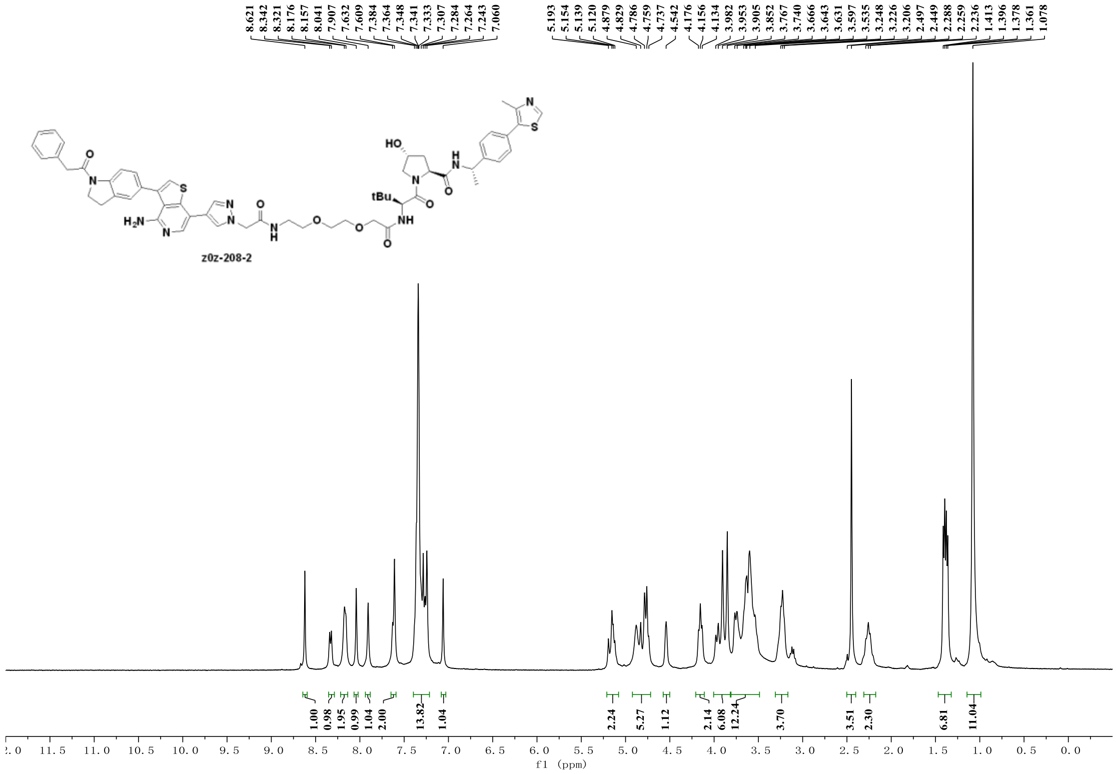
z0z-208-2
f1 (ppm)

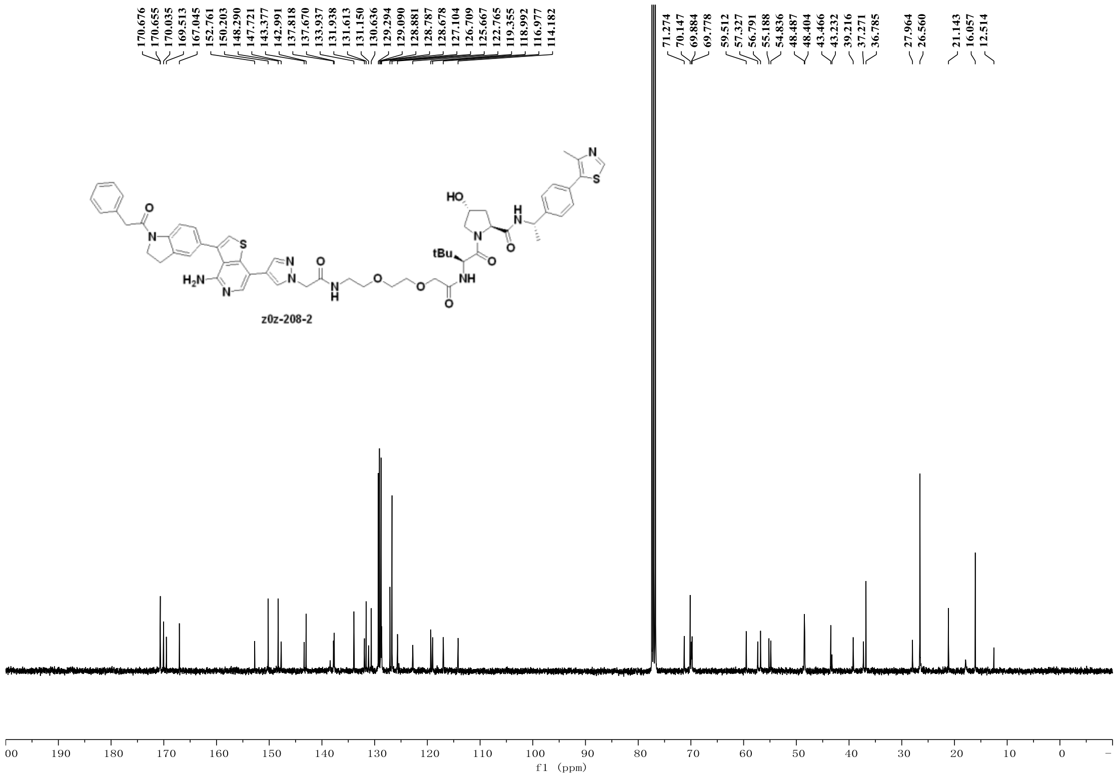
z0z-208-2
f1 (ppm)

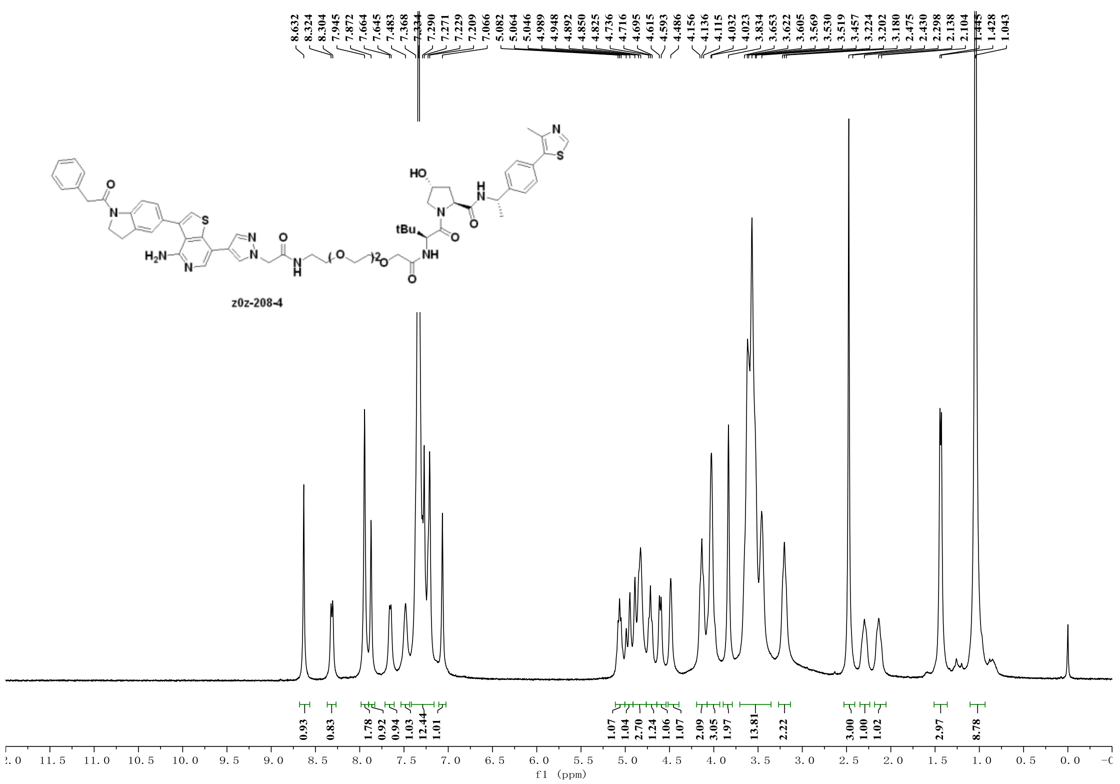
8.632 8.324 8.304 7.945 7.872 7.664 7.645 7.483 7.368 7.334 7.290 7.271 7.229 7.209 7.066 5.082 5.064 5.046 4.989 4.948 4.892 4.850 4.825 4.736 4.716 4.695 4.615 4.593 4.486 4.156 4.136 4.115 4.032 4.023 3.834 3.653 3.622 3.605 3.569 3.530 3.519 3.457 3.224 3.202 3.180 2.475 2.430 2.298 2.138 2.104 1.445 1.428 1.043
HO
tBu
H2N
z0z-208-4
0.93 0.83 1.78 0.92 0.94 1.03 12.44 1.01 1.07 1.04 2.70 1.24 1.06 1.07 2.09 3.05 1.97 13.81 2.22 3.00 1.00 1.02 2.97 8.78
f1 (ppm)

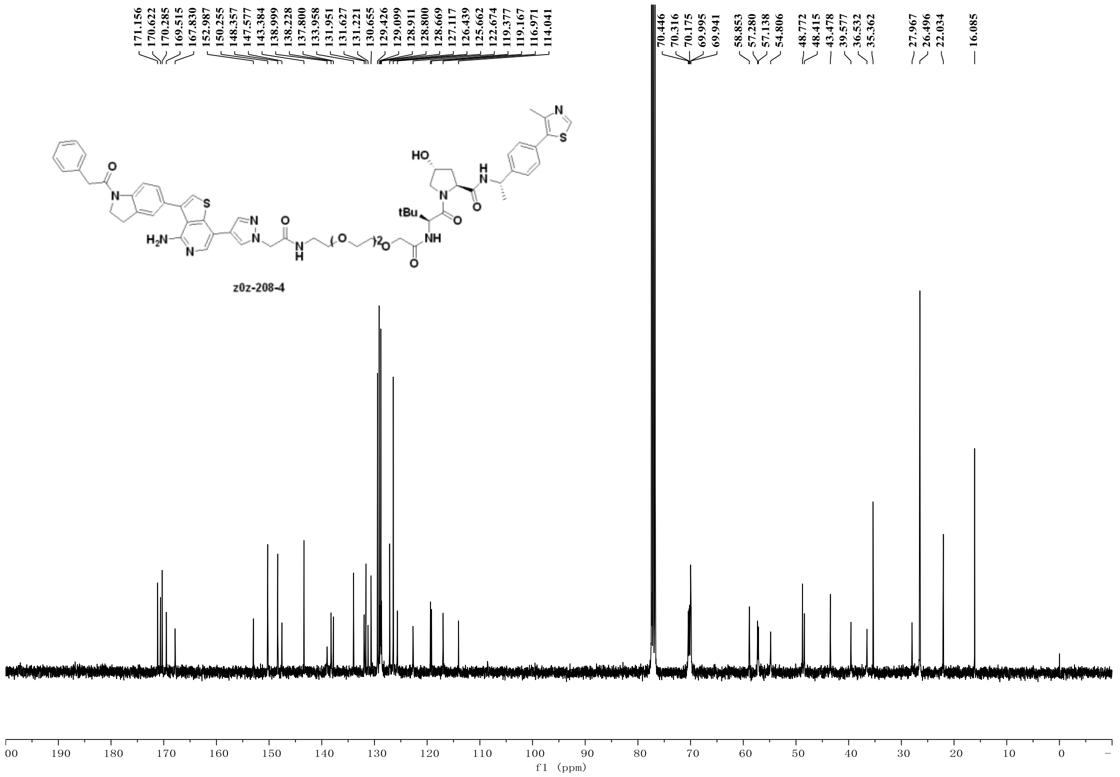
171.156 170.622 170.285 169.515 167.830 152.987 150.255 148.357 147.577 143.384 138.999 138.228 137.800 133.958 131.951 131.627 131.221 130.655 129.426 129.099 128.911 128.800 128.669 127.117 126.439 125.662 122.674 119.377 119.167 116.971 114.041
70.446 70.316 70.175 69.995 69.941 58.853 57.280 57.138 54.806 48.772 48.415 43.478 39.577 36.532 35.362 27.967 26.496 22.034 16.085
HO
tBu
H2N
z0z-208-4
f1 (ppm)

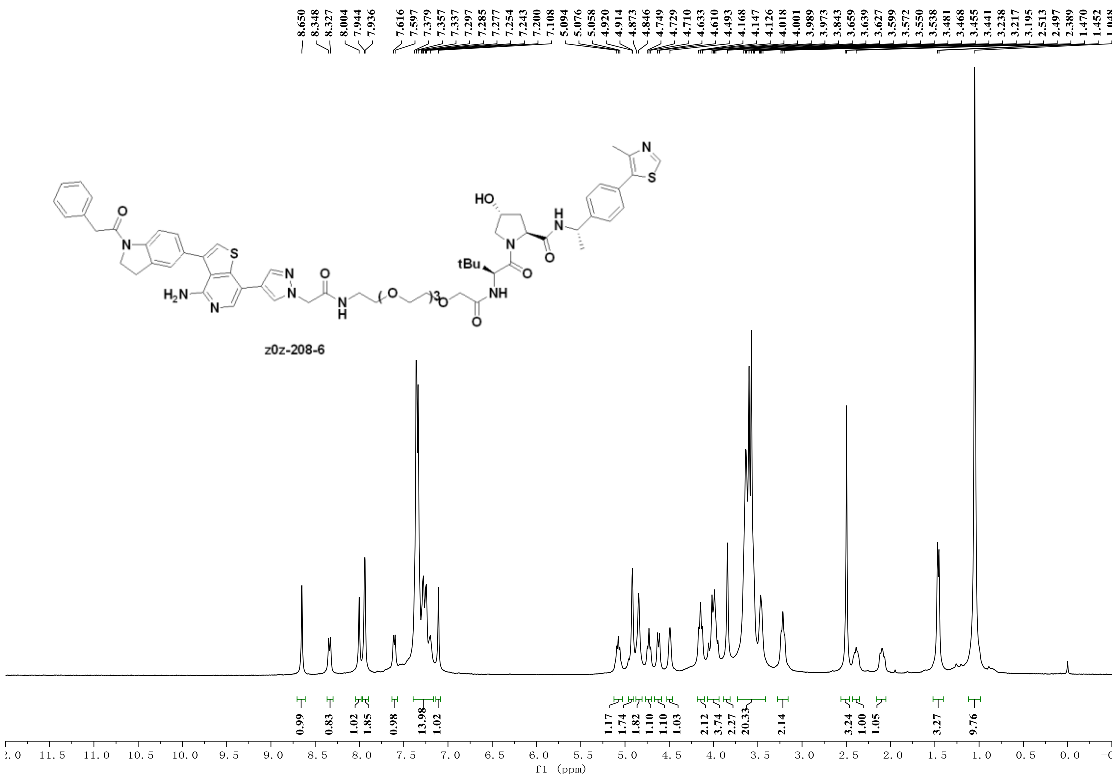
8.650 8.348 8.327 8.004 7.944 7.936 7.616 7.597 7.379 7.357 7.337 7.297 7.285 7.277 7.254 7.243 7.200 7.108 5.094 5.076 5.058 4.920 4.914 4.873 4.846 4.749 4.729 4.710 4.633 4.610 4.493 4.168 4.147 4.126 4.018 4.001 3.989 3.973 3.843 3.659 3.639 3.627 3.599 3.572 3.550 3.538 3.481 3.468 3.455 3.441 3.238 3.217 3.195 2.513 2.497 2.389 1.470 1.452
HO
N
S
NH
N
O
tBu
NH
O
O
S
N
H2N
N
N
N
O
N
H
O
3
O
O
z0z-208-6
0.99 0.83 1.02 1.85 0.98 13.98 1.02 1.17 1.74 1.82 1.10 1.10 1.03 2.12 3.74 2.27 20.33 2.14 3.24 1.00 1.05 3.27 9.76
11.5 11.0 10.5 10.0 9.5 9.0 8.5 8.0 7.5 7.0 6.5 6.0 5.5 5.0 4.5 4.0 3.5 3.0 2.5 2.0 1.5 1.0 0.5 0.0
f1 (ppm)

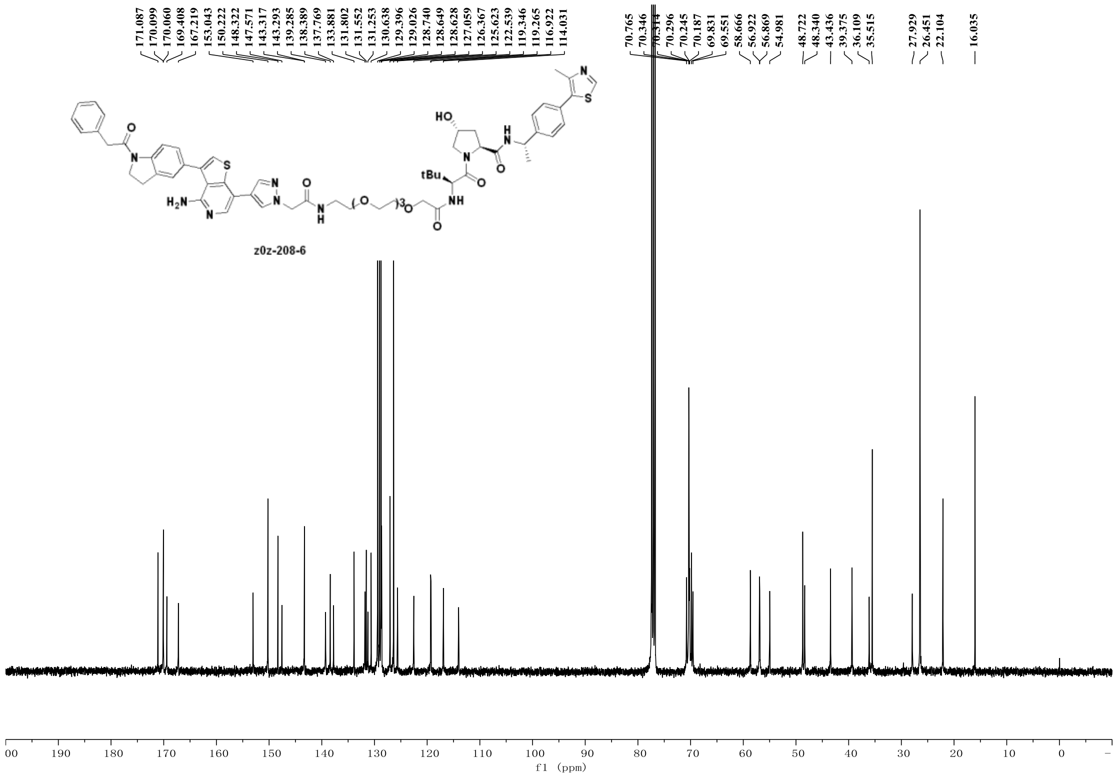
171.087 170.099 170.060 169.408 167.219 153.043 150.222 148.322 147.571 143.317 143.293 139.285 138.389 137.769 133.881 131.802 131.552 131.253 130.638 129.396 129.026 128.740 128.649 128.628 127.059 126.367 125.623 122.539 119.346 119.265 116.922 114.031
70.765 70.346 70.296 70.245 70.187 69.831 69.551 58.666 56.922 56.869 54.981 48.722 48.340 43.436 39.375 36.109 35.515 27.929 26.451 22.104 16.035
z0z-208-6
190 180 170 160 150 140 130 120 110 100 90 80 70 60 50 40 30 20 10 0
f1 (ppm)

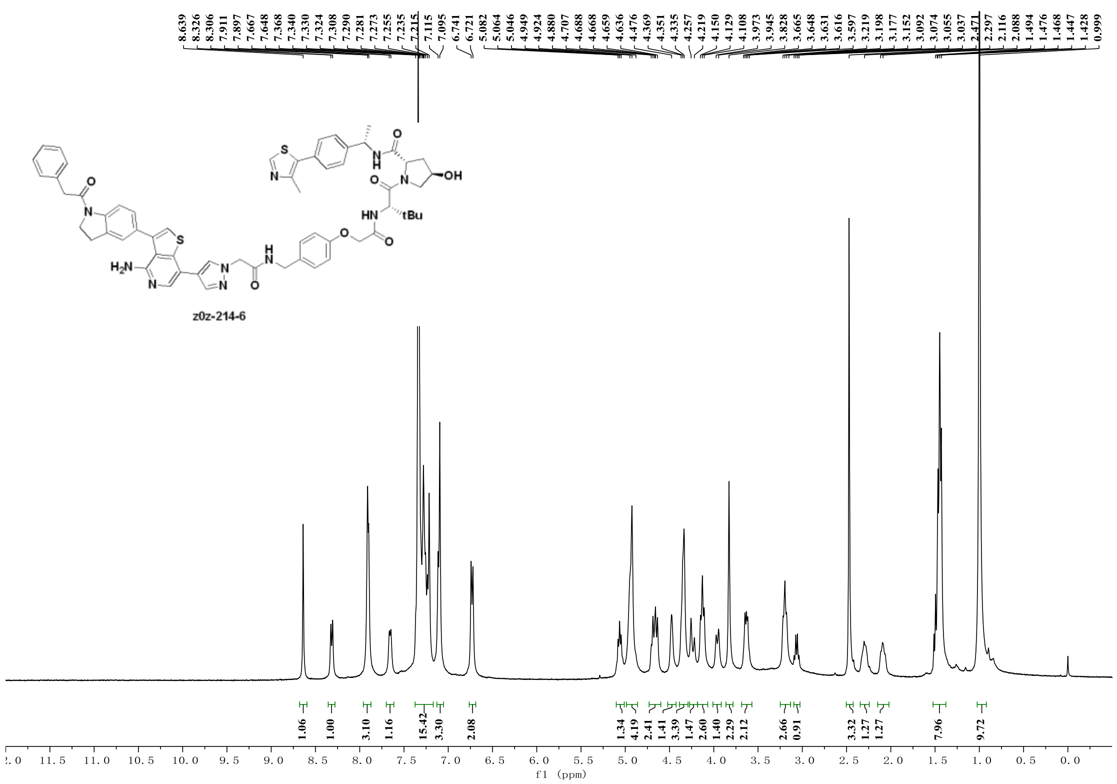
z0z-214-6
f1 (ppm)

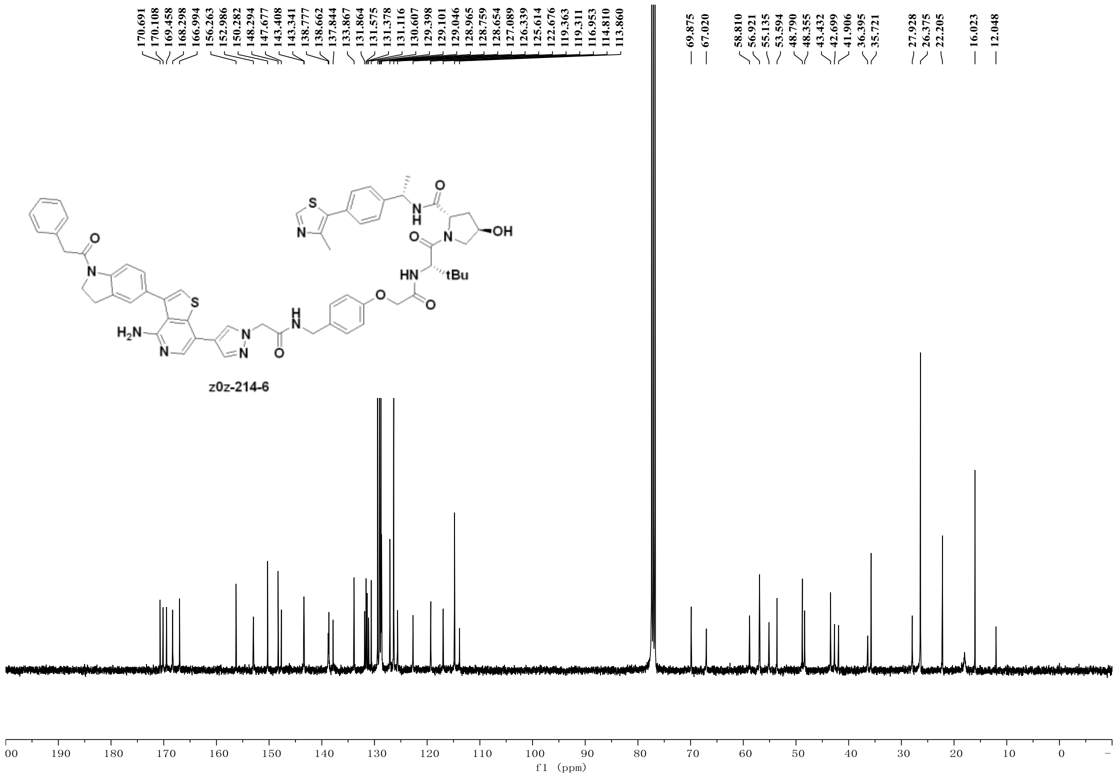
z0z-214-6
f1 (ppm)

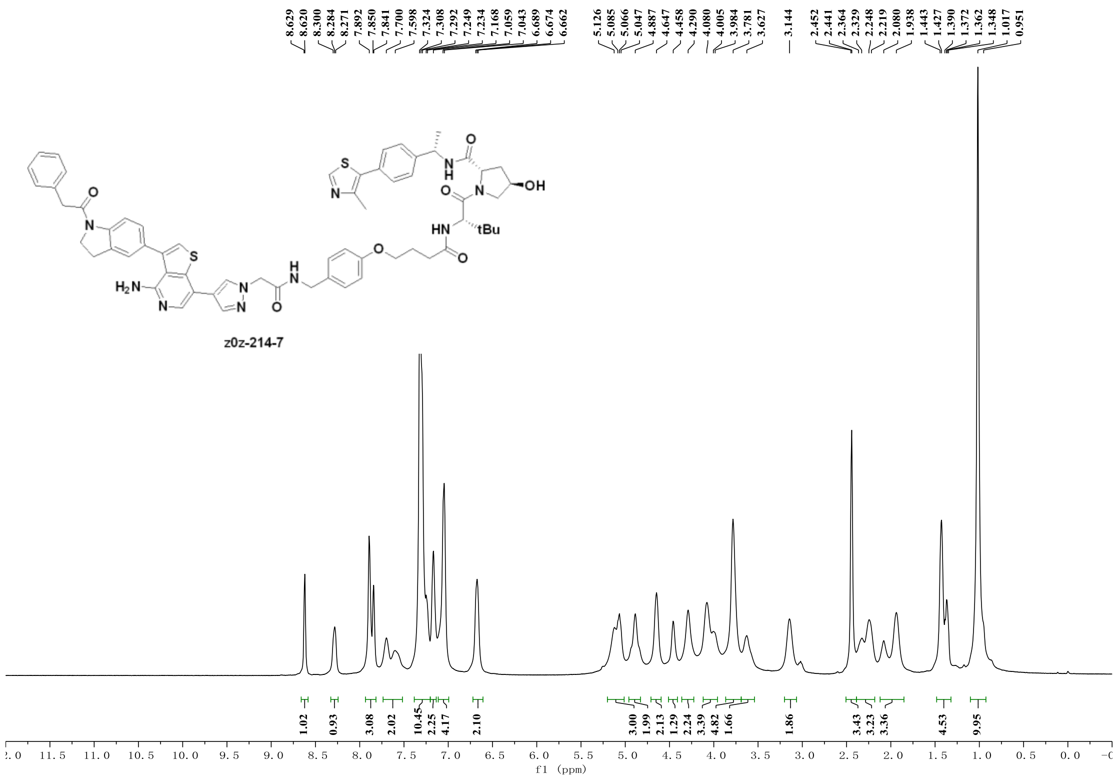
z0z-214-7
f1 (ppm)

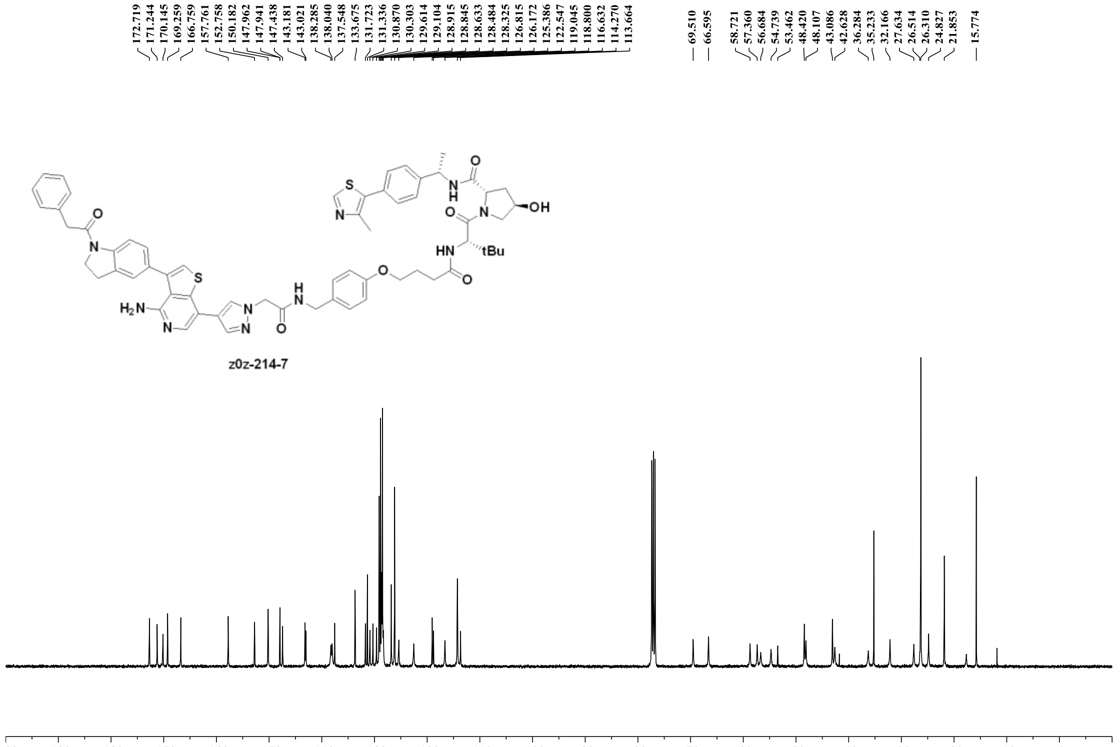
z0z-214-7
f1 (ppm)

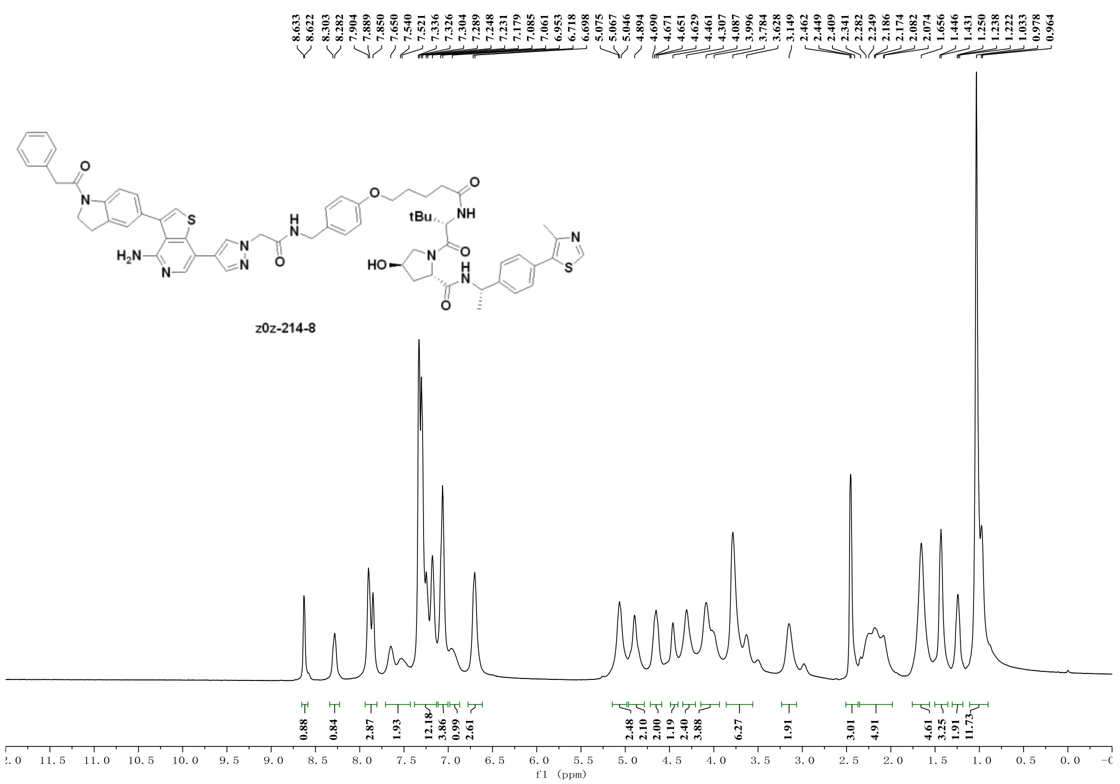
8.633 8.622 8.303 8.282 7.904 7.889 7.850 7.650 7.540 7.521 7.336 7.326 7.304 7.289 7.248 7.231 7.179 7.085 7.061 6.953 6.718 6.698 5.075 5.067 5.046 4.894 4.690 4.671 4.651 4.629 4.461 4.307 4.087 3.996 3.784 3.628 3.149 2.462 2.449 2.409 2.341 2.282 2.249 2.186 2.174 2.082 2.074 1.656 1.446 1.431 1.250 1.238 1.222 1.033 0.978 0.964
tBu
NH
O
H_2N
HO
N
S
z0z-214-8
0.88 0.84 2.87 1.93 12.18 3.86 0.99 2.61 2.48 2.10 2.00 1.19 2.40 3.88 6.27 1.91 3.01 4.91 4.61 3.25 1.91 11.73
f1 (ppm)

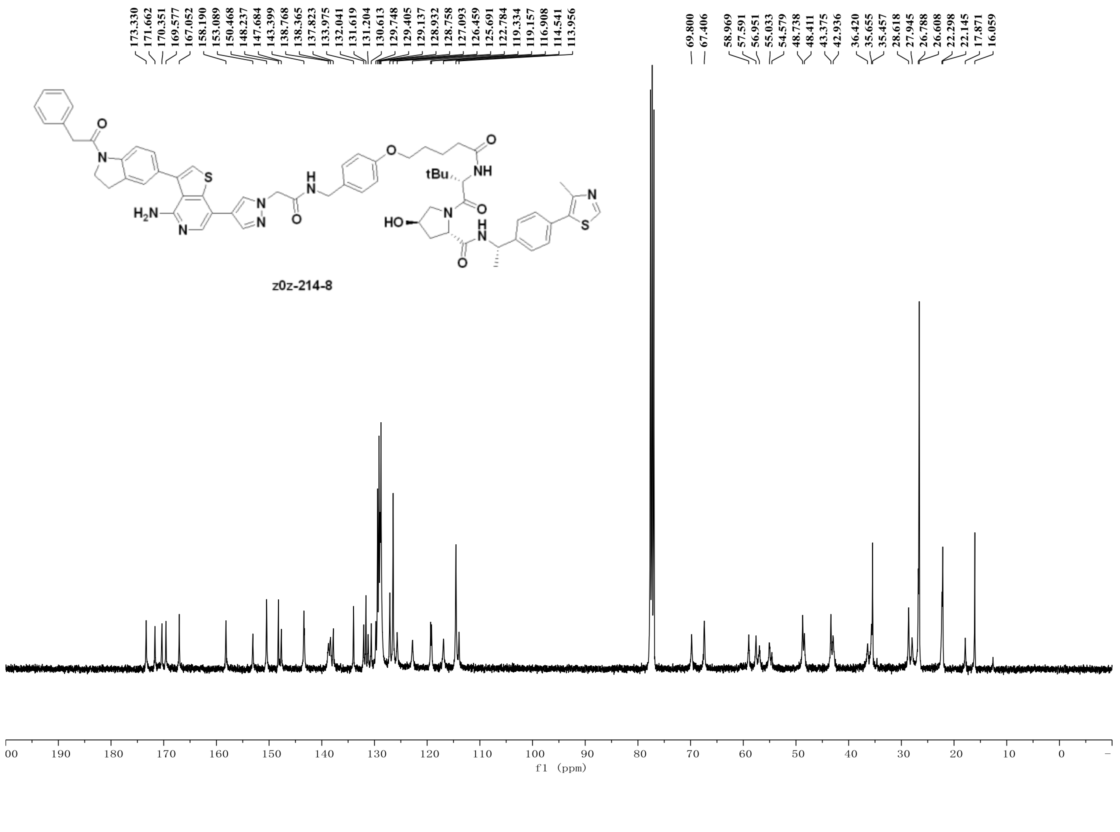
173.330 171.662 170.351 169.577 167.052 158.190 153.089 150.468 148.237 147.684 143.399 138.768 138.365 137.823 133.975 132.041 131.619 131.204 130.613 129.748 129.405 129.137 128.932 128.758 127.093 126.459 125.691 122.784 119.334 119.157 116.908 114.541 113.956
69.800 67.406 58.969 57.591 56.951 55.033 54.579 48.738 48.411 43.375 42.936 36.420 35.655 35.457 28.618 27.945 26.788 26.608 22.298 22.145 17.871 16.059
tBu
NH
O
H_2N
HO
N
S
z0z-214-8
f1 (ppm)

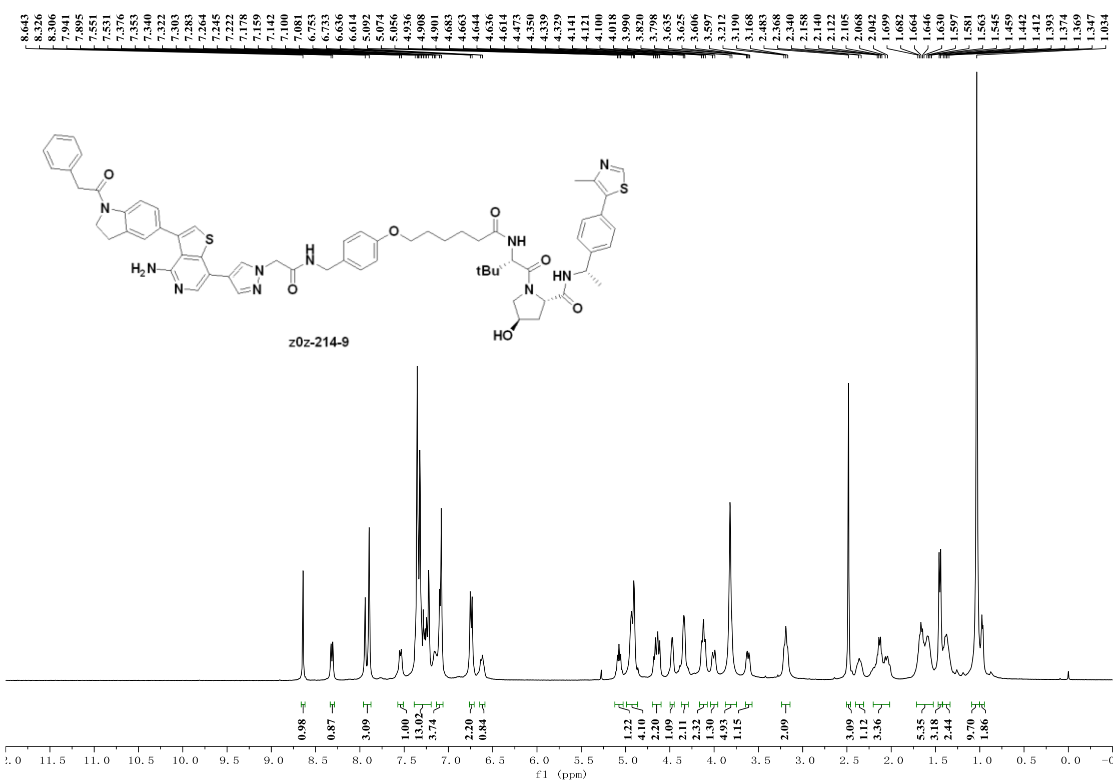
8.643 8.326 8.306 7.941 7.895 7.551 7.531 7.376 7.353 7.340 7.322 7.303 7.283 7.264 7.245 7.222 7.178 7.159 7.142 7.100 7.081 6.753 6.733 6.636 6.614 5.092 5.074 5.056 4.936 4.908 4.901 4.683 4.663 4.644 4.636 4.614 4.473 4.350 4.339 4.329 4.141 4.121 4.100 4.018 3.990 3.820 3.798 3.635 3.625 3.606 3.597 3.212 3.190 3.168 2.483 2.368 2.340 2.158 2.140 2.122 2.105 2.068 2.042 1.699 1.682 1.664 1.646 1.630 1.597 1.581 1.563 1.545 1.459 1.442 1.412 1.393 1.374 1.369 1.347 1.034
H₂N
tBu
HN
NH
HO
z0z-214-9
0.98 0.87 3.09 1.00 13.02 3.74 2.20 0.84 1.22 4.10 2.20 1.09 2.11 2.32 1.30 4.93 1.15 2.09 3.09 1.12 3.36 5.35 3.18 2.44 9.70 1.86
12.0 11.5 11.0 10.5 10.0 9.5 9.0 8.5 8.0 7.5 7.0 6.5 6.0 5.5 5.0 4.5 4.0 3.5 3.0 2.5 2.0 1.5 1.0 0.5 0.0
f1 (ppm)

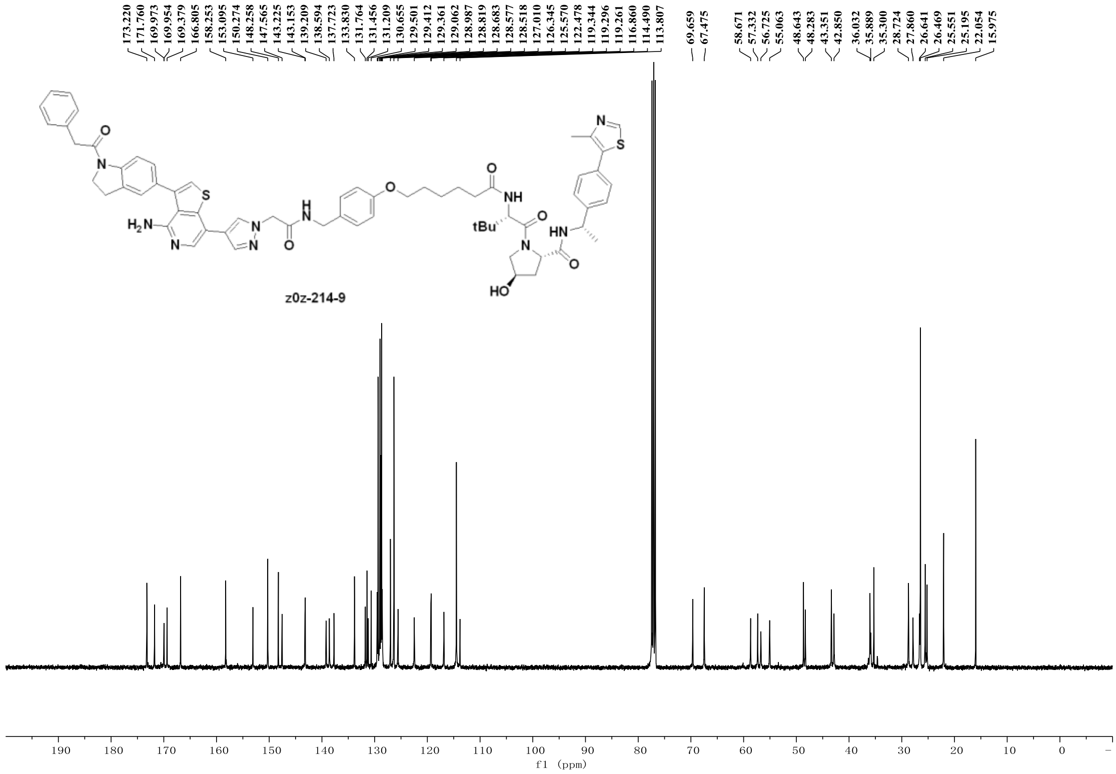
173.220 171.760 169.973 169.954 169.379 166.805 158.253 153.095 150.274 148.258 147.565 143.225 143.153 139.209 138.594 137.723 133.830 131.764 131.456 131.209 130.655 129.501 129.412 129.361 129.062 128.987 128.819 128.683 128.577 128.518 127.010 126.345 125.570 122.478 119.344 119.296 119.261 116.860 114.490 113.807 69.659 67.475 58.671 57.332 56.725 55.063 48.643 48.283 43.351 42.850 36.032 35.889 35.300 28.724 27.860 26.641 26.469 25.551 25.195 22.054 15.975
H₂N
tBu
HN
NH
HO
z0z-214-9
190 180 170 160 150 140 130 120 110 100 90 80 70 60 50 40 30 20 10 0
f1 (ppm)

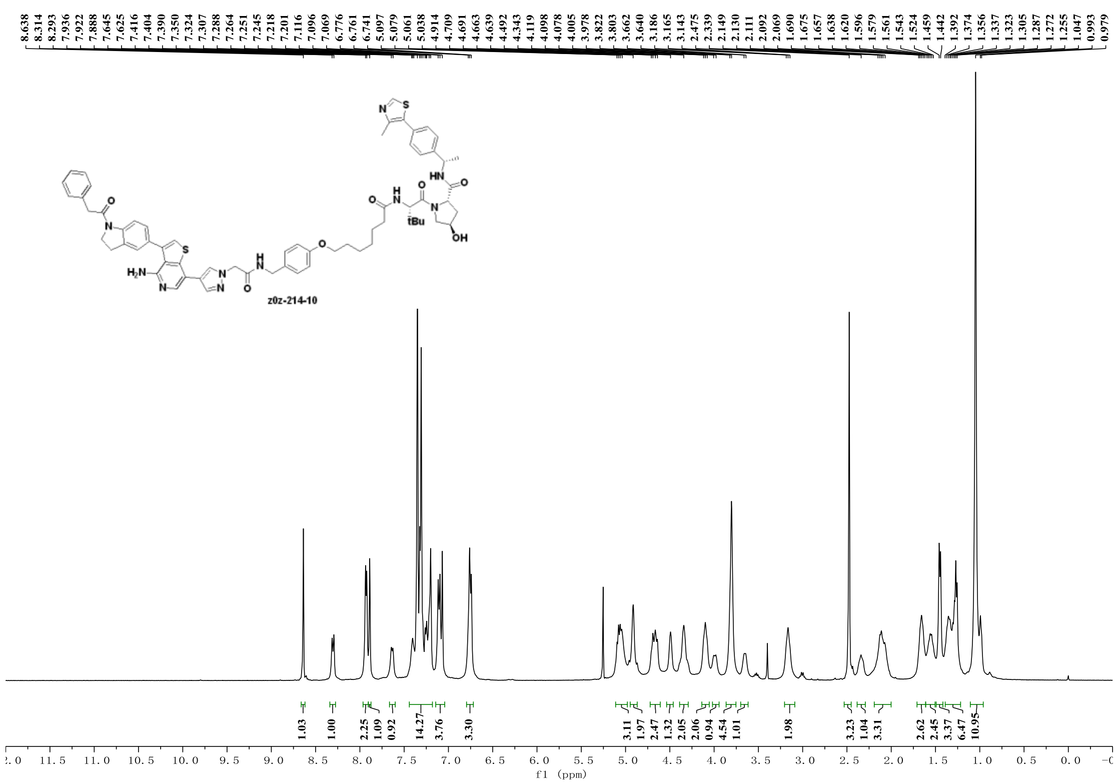
8.638 8.314 8.293 7.936 7.922 7.888 7.645 7.625 7.416 7.404 7.390 7.350 7.324 7.307 7.288 7.264 7.251 7.245 7.218 7.201 7.116 7.096 7.069 6.776 6.761 6.741 5.097 5.079 5.061 5.038 4.914 4.709 4.691 4.663 4.639 4.492 4.343 4.119 4.098 4.078 4.005 3.978 3.822 3.803 3.662 3.640 3.186 3.165 3.143 2.475 2.339 2.149 2.130 2.111 2.092 2.069 1.690 1.675 1.657 1.638 1.620 1.596 1.579 1.561 1.543 1.524 1.459 1.442 1.392 1.374 1.356 1.337 1.323 1.305 1.287 1.272 1.255 1.047 0.993 0.979
H2N
tBu
OH
z0z-214-10
1.03 1.00 2.25 1.09 0.92 14.27 3.76 3.30 3.11 1.97 2.47 1.32 2.05 2.06 0.94 4.54 1.01 1.98 3.23 1.04 3.31 2.62 2.45 3.37 6.47 10.95
f1 (ppm)

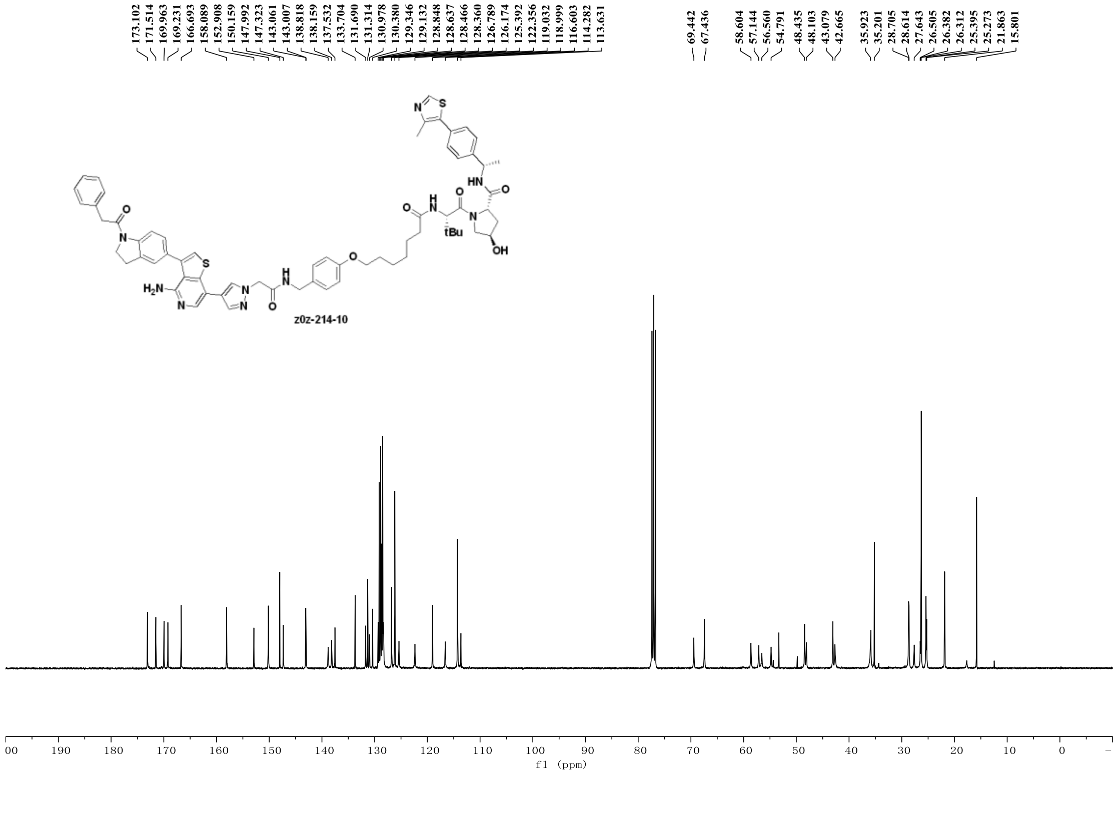
173.102 171.514 169.963 169.231 166.693 158.089 152.908 150.159 147.992 147.323 143.061 143.007 138.818 138.159 137.532 133.704 131.690 131.314 130.978 130.380 129.346 129.132 128.848 128.637 128.466 128.360 126.789 126.174 125.392 122.356 119.032 118.999 116.603 114.282 113.631
69.442 67.436
58.604 57.144 56.560 54.791 48.435 48.103 43.079 42.665 35.923 35.201 28.705 28.614 27.643 26.505 26.382 26.312 25.395 25.273 21.863 15.801
H2N
tBu
OH
z0z-214-10
f1 (ppm)

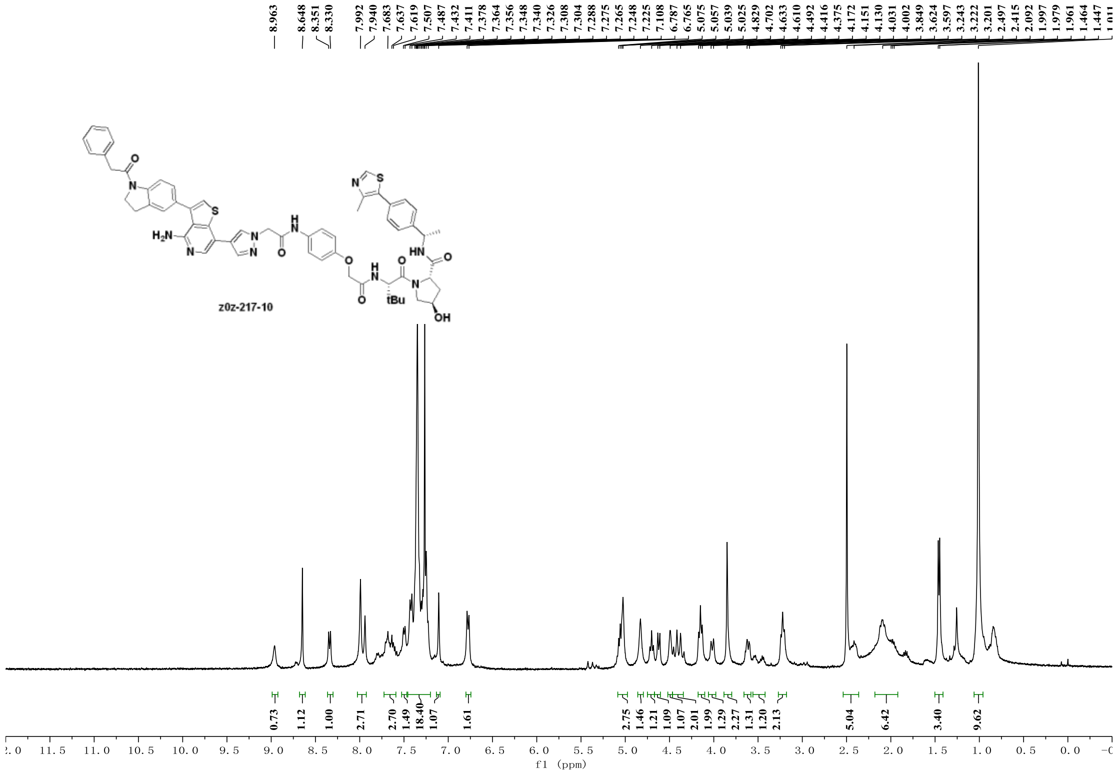
8.963
8.648
8.351
8.330
7.992
7.940
7.683
7.637
7.619
7.507
7.487
7.432
7.411
7.378
7.364
7.356
7.348
7.340
7.326
7.308
7.304
7.288
7.275
7.265
7.248
7.225
7.108
6.787
6.765
5.075
5.057
5.039
5.025
4.829
4.702
4.633
4.610
4.492
4.416
4.375
4.172
4.151
4.130
4.031
4.002
3.849
3.624
3.597
3.243
3.222
3.201
2.497
2.415
2.092
1.997
1.979
1.961
1.464
1.447
z0z-217-10
0.73
1.12
1.00
2.71
2.70
1.49
18.40
1.07
1.61
2.75
1.46
1.21
1.09
1.07
2.01
1.99
1.29
2.27
1.31
1.20
2.13
5.04
6.42
3.40
9.62
f1 (ppm)

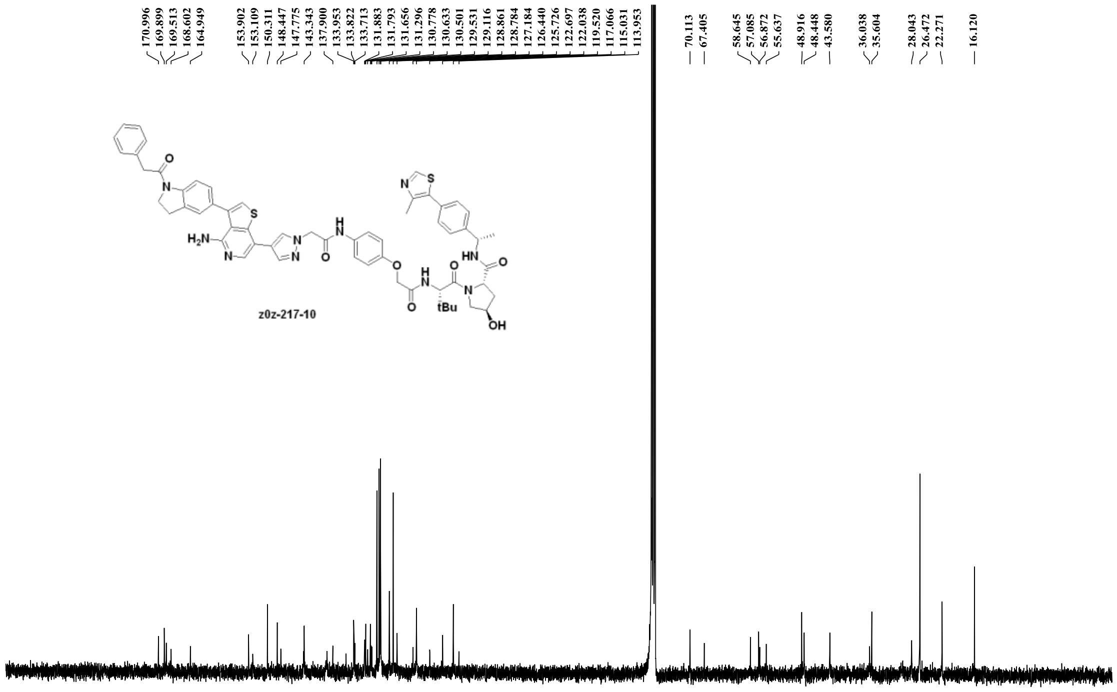
170.996
169.899
169.513
168.602
164.949
153.902
153.109
150.311
148.447
147.775
143.343
137.900
133.953
133.822
133.713
131.883
131.793
131.656
131.296
130.778
130.633
130.501
129.531
129.116
128.861
128.784
127.184
126.440
125.726
122.697
122.038
119.520
117.066
115.031
113.953
70.113
67.405
58.645
57.085
56.872
55.637
48.916
48.448
43.580
36.038
35.604
28.043
26.472
22.271
16.120
z0z-217-10
f1 (ppm)

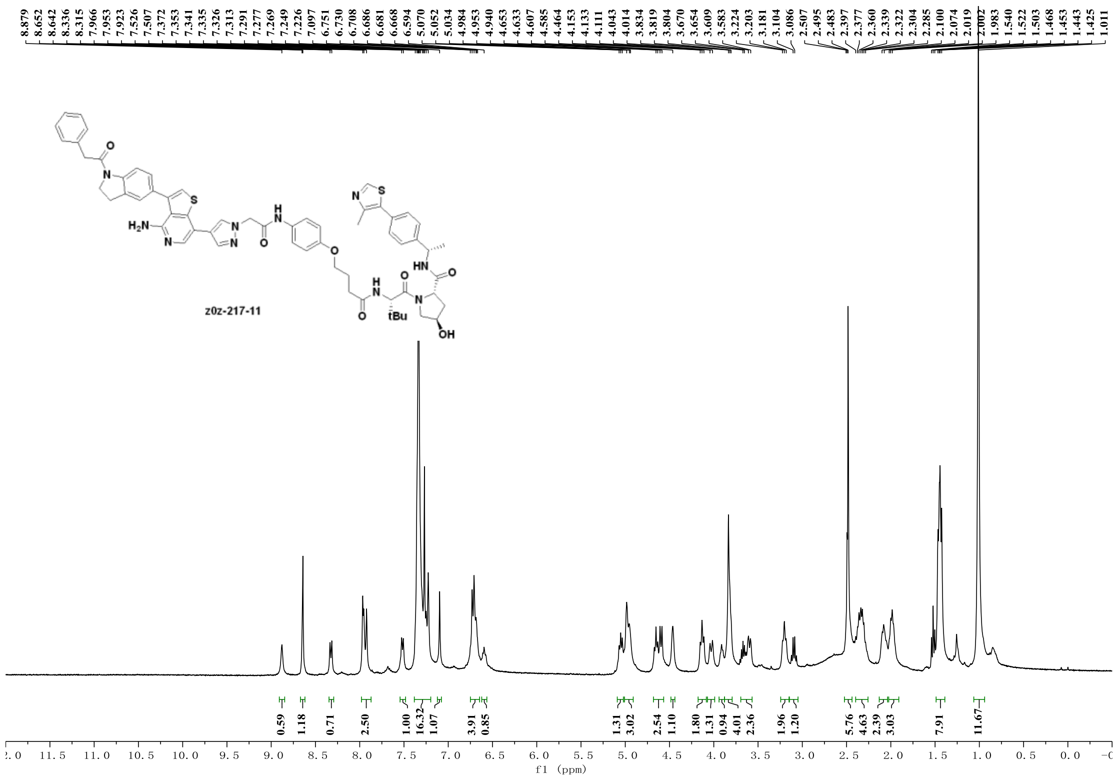
8.879 8.652 8.642 8.336 8.315 7.966 7.953 7.923 7.526 7.507 7.372 7.353 7.341 7.335 7.326 7.313 7.291 7.277 7.269 7.249 7.226 7.097 6.751 6.730 6.708 6.686 6.681 6.668 6.594 5.070 5.052 5.034 4.984 4.953 4.940 4.653 4.633 4.607 4.585 4.464 4.153 4.133 4.111 4.043 4.014 3.834 3.819 3.804 3.670 3.654 3.609 3.583 3.224 3.203 3.181 3.104 3.086 2.507 2.495 2.483 2.397 2.377 2.360 2.339 2.322 2.304 2.285 2.100 2.074 2.019 2.002 1.983 1.540 1.522 1.503 1.468 1.453 1.443 1.425 1.011
z0z-217-11
f1 (ppm)

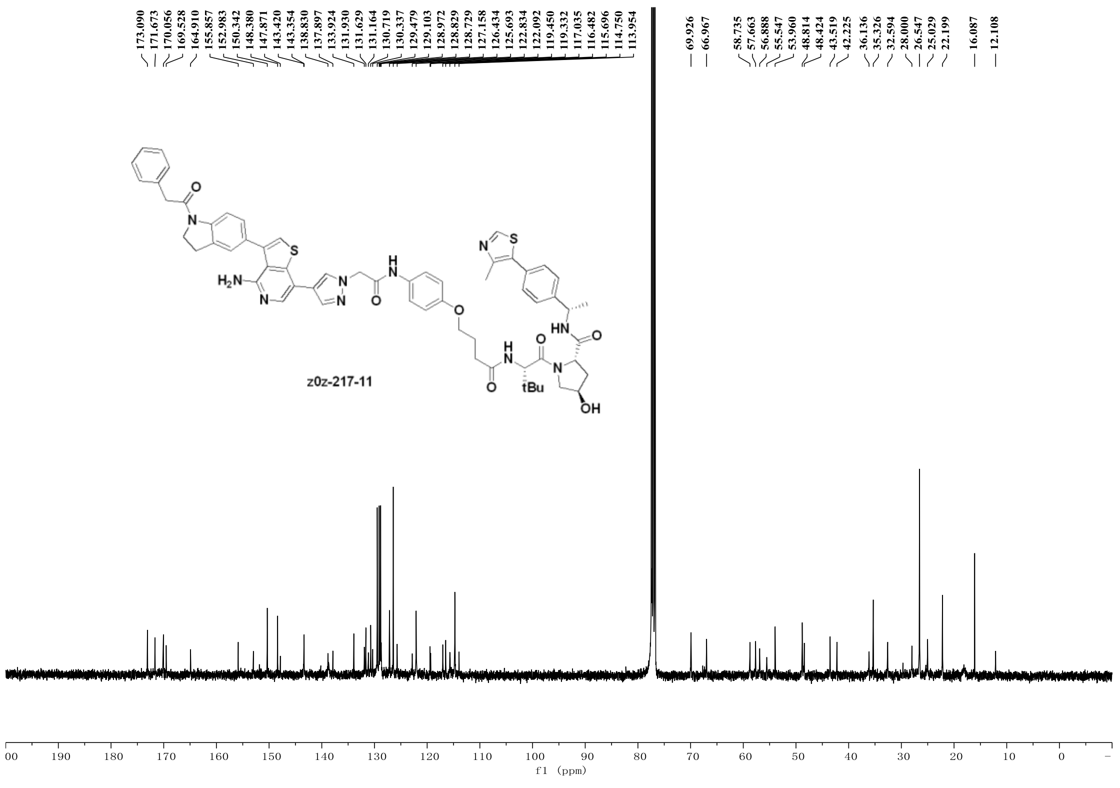
173.090 171.673 170.056 169.528 164.910 155.857 152.983 150.342 148.380 147.871 143.420 143.354 138.830 137.897 133.924 131.930 131.629 131.164 130.719 130.337 129.479 129.103 128.972 128.829 128.729 127.158 126.434 125.693 122.834 122.092 119.450 119.332 117.035 116.482 115.696 114.750 113.954
69.926 66.967
58.735 57.663 56.888 55.547 53.960 48.814 48.424 43.519 42.225 36.136 35.326 32.594 28.000 26.547 25.029 22.199 16.087 12.108
z0z-217-11
f1 (ppm)

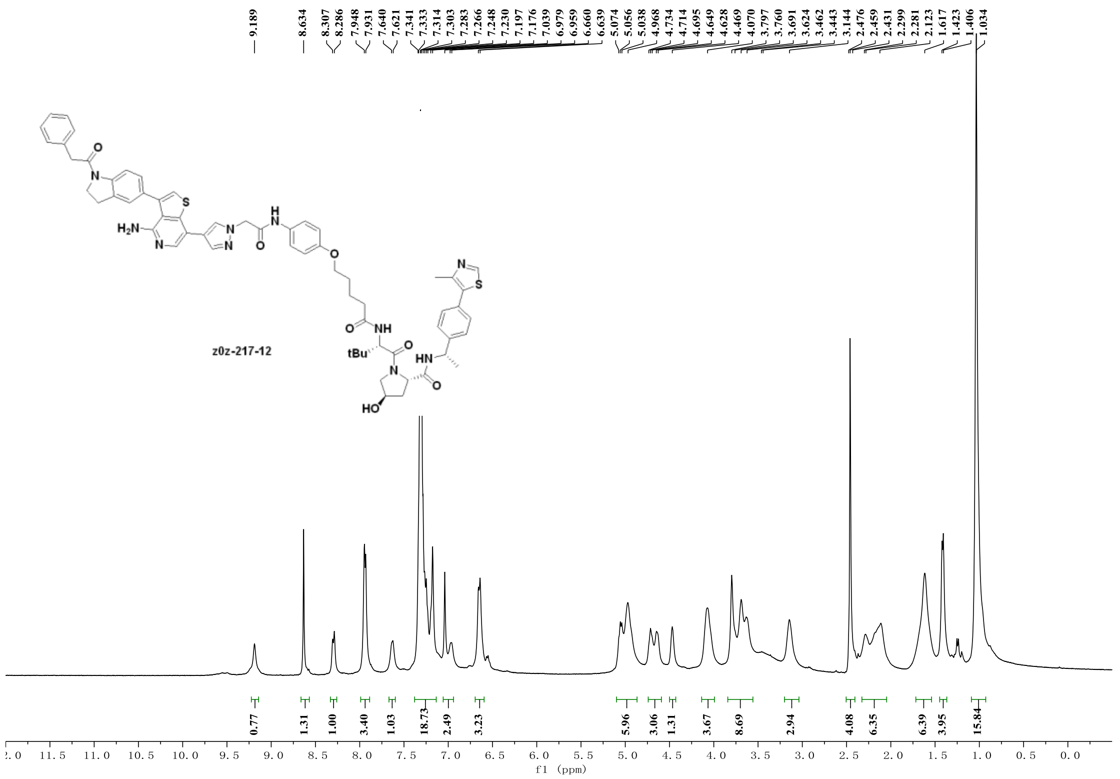
9.189
8.634
8.307
8.286
7.948
7.931
7.640
7.621
7.341
7.333
7.314
7.303
7.283
7.266
7.248
7.230
7.197
7.176
7.039
6.979
6.959
6.660
6.639
5.074
5.056
5.038
4.968
4.734
4.714
4.695
4.649
4.628
4.469
4.070
3.797
3.760
3.691
3.624
3.462
3.443
3.144
2.476
2.459
2.431
2.299
2.281
2.123
1.617
1.423
1.406
1.034
z0z-217-12
0.77
1.31
1.00
3.40
1.03
18.73
2.49
3.23
5.96
3.06
1.31
3.67
8.69
2.94
4.08
6.35
6.39
3.95
15.84
f1 (ppm)

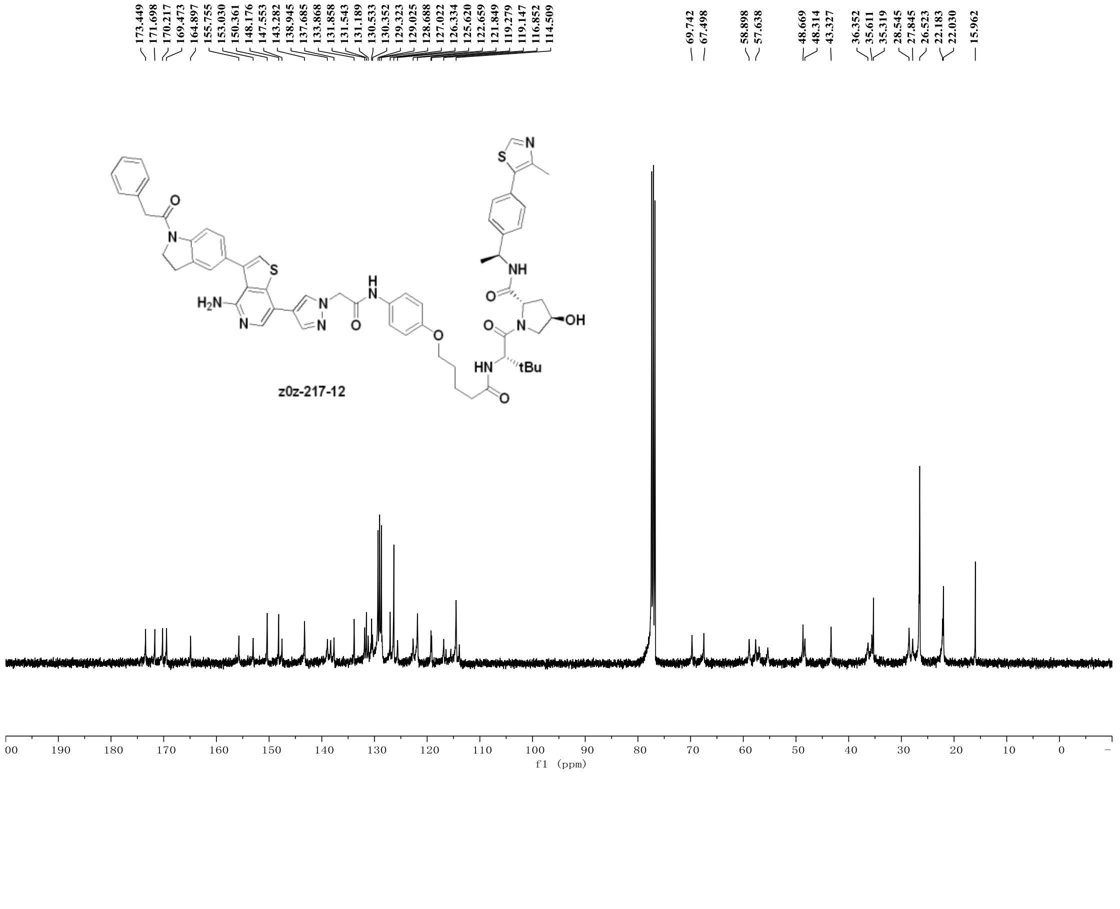
173.449
171.698
170.217
169.473
164.897
155.755
153.030
150.361
148.176
147.553
143.282
138.945
137.685
133.868
131.858
131.543
131.189
130.533
130.352
129.323
129.025
128.688
127.022
126.334
125.620
122.659
121.849
119.279
119.147
116.852
114.509
69.742
67.498
58.898
57.638
48.669
48.314
43.327
36.352
35.611
35.319
28.545
27.845
26.523
22.183
22.030
15.962
z0z-217-12
f1 (ppm)

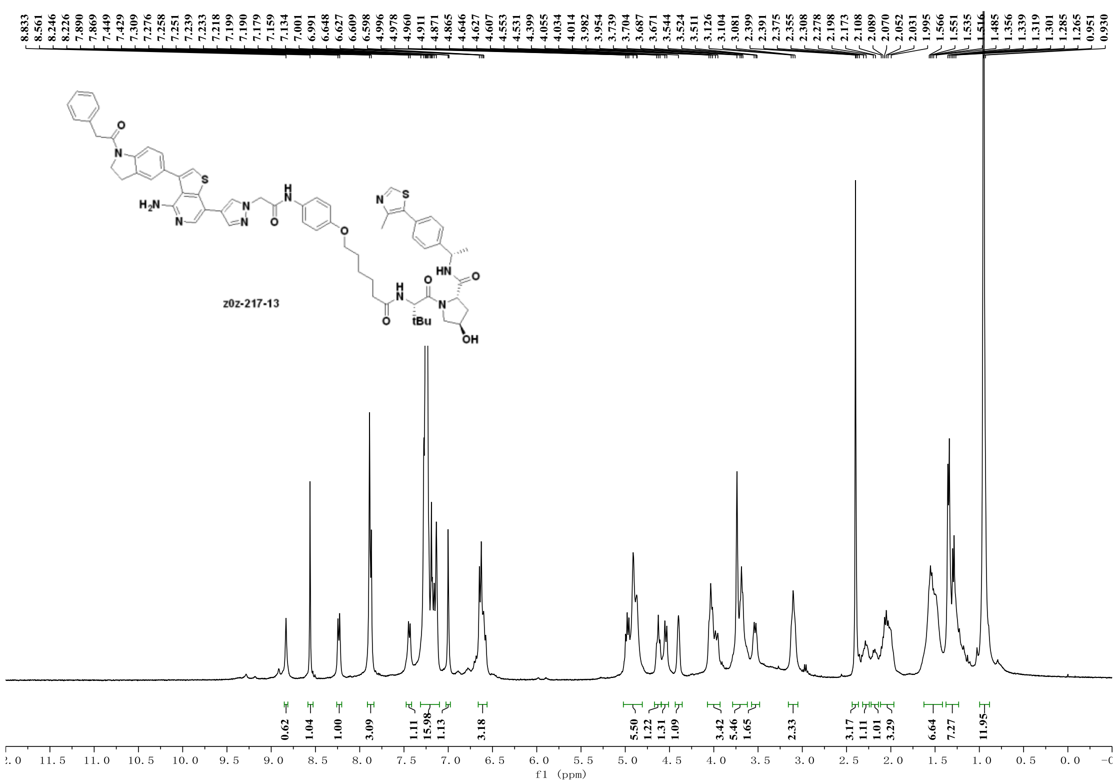
z0z-217-13
f1 (ppm)

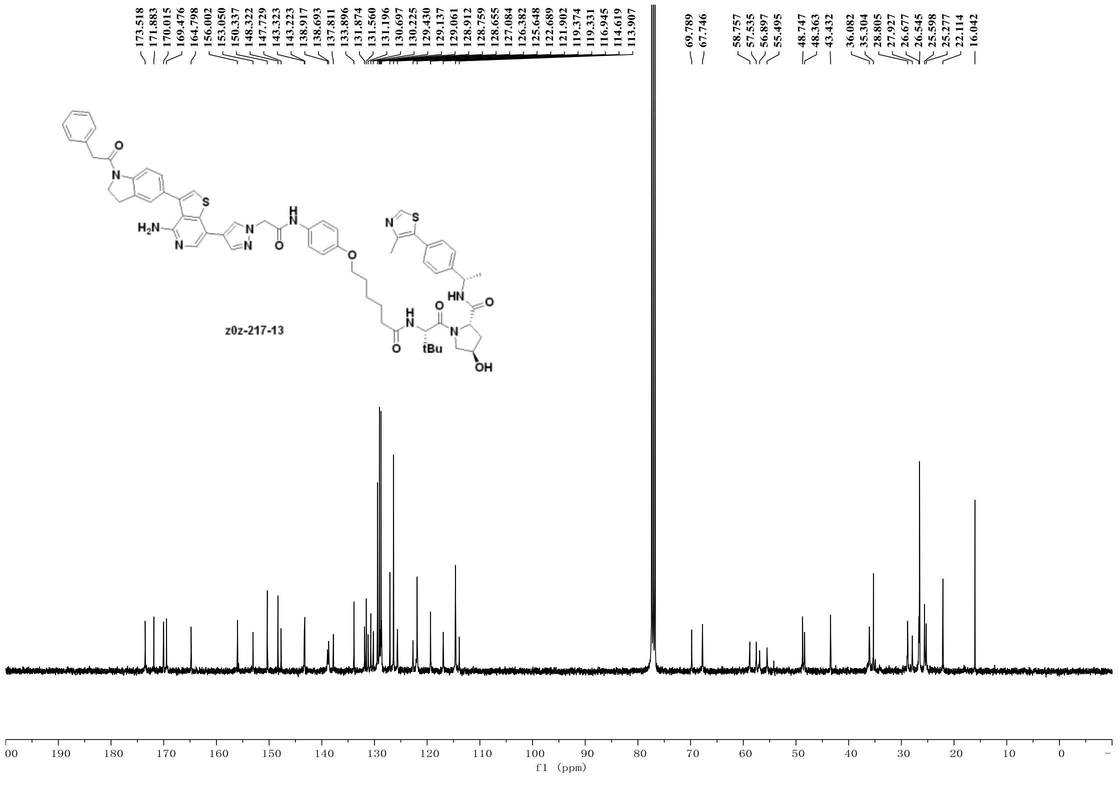
z0z-217-13
f1 (ppm)

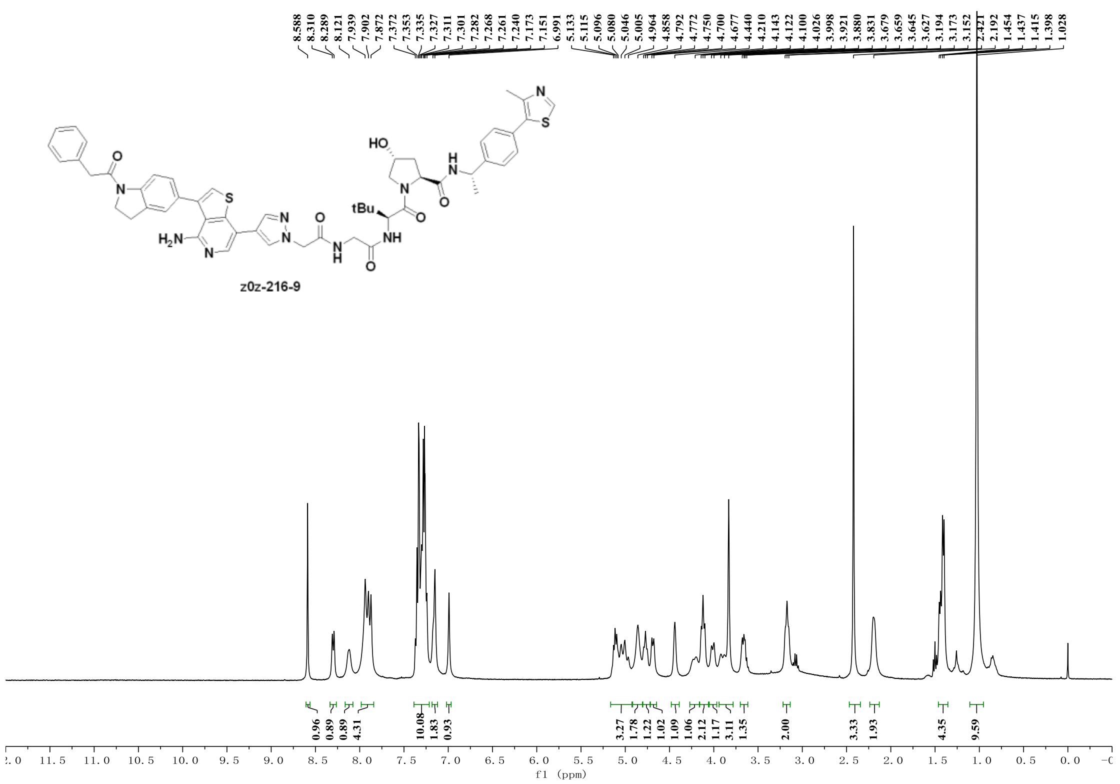
8.588
8.310
8.289
8.121
7.939
7.902
7.872
7.372
7.353
7.335
7.327
7.311
7.301
7.282
7.268
7.261
7.240
7.173
7.151
6.991
5.133
5.115
5.096
5.080
5.046
5.005
4.964
4.858
4.792
4.772
4.750
4.700
4.677
4.440
4.210
4.143
4.122
4.100
4.026
3.998
3.921
3.880
3.831
3.679
3.659
3.645
3.627
3.194
3.173
3.152
2.421
2.192
1.454
1.437
1.415
1.398
1.028
HO
tBu
NH
N
S
O
H₂N
z0z-216-9
0.96
0.89
0.89
4.31
10.08
1.83
0.93
3.27
1.78
1.22
1.02
1.09
1.06
2.12
1.17
3.11
1.35
2.00
3.33
1.93
4.35
9.59
11.5 11.0 10.5 10.0 9.5 9.0 8.5 8.0 7.5 7.0 6.5 6.0 5.5 5.0 4.5 4.0 3.5 3.0 2.5 2.0 1.5 1.0 0.5 0.0
f1 (ppm)

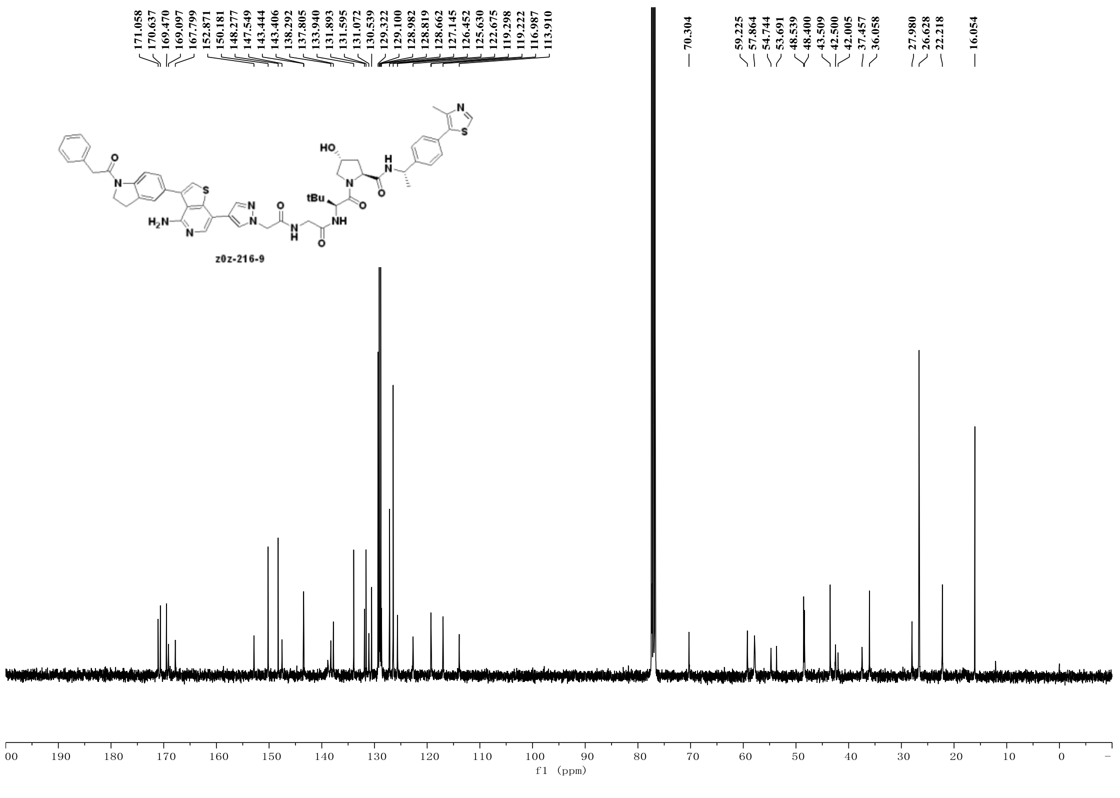
171.058
170.637
169.470
169.097
167.799
152.871
150.181
148.277
147.549
143.444
143.406
138.292
137.805
133.940
131.893
131.595
131.072
130.539
129.322
129.100
128.982
128.819
128.662
127.145
126.452
125.630
122.675
119.298
119.222
116.987
113.910
70.304
59.225
57.864
54.744
53.691
48.539
48.400
43.509
42.500
42.005
37.457
36.058
27.980
26.628
22.218
16.054
z0z-216-9
190 180 170 160 150 140 130 120 110 100 90 80 70 60 50 40 30 20 10 0
f1 (ppm)

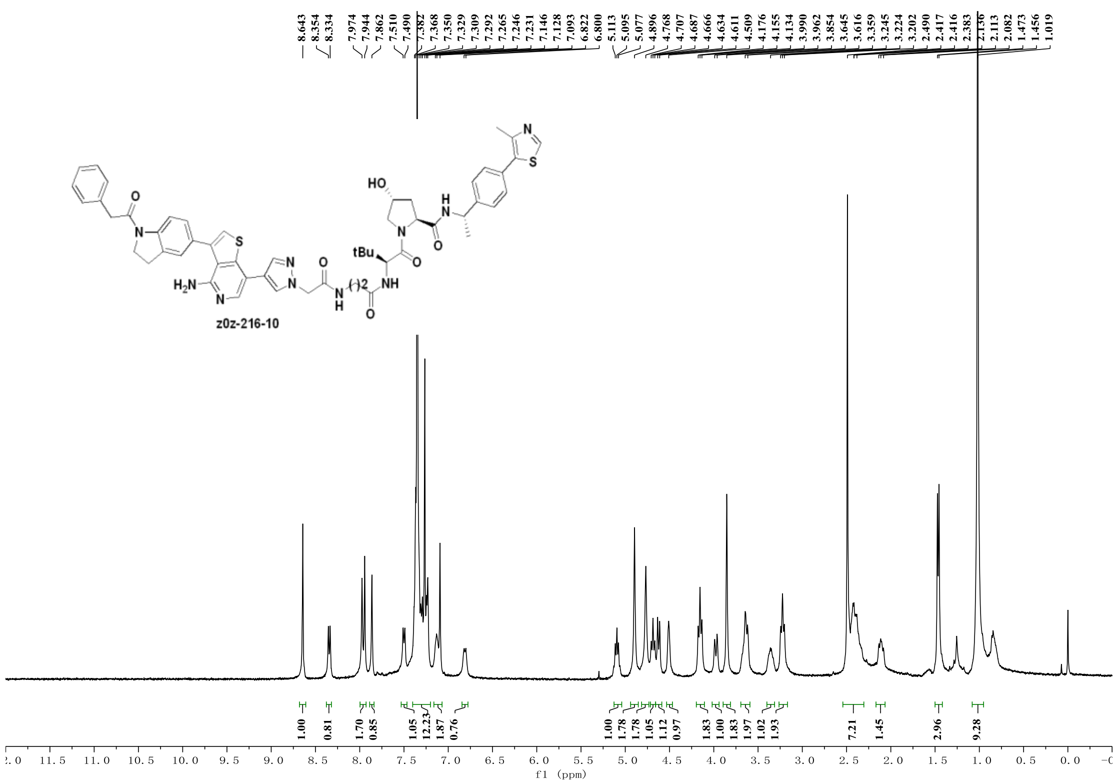
8.643
8.354
8.334
7.974
7.944
7.862
7.510
7.490
7.382
7.368
7.350
7.329
7.309
7.292
7.265
7.246
7.231
7.146
7.128
7.093
6.822
6.800
5.113
5.095
5.077
4.896
4.768
4.707
4.687
4.666
4.634
4.611
4.509
4.176
4.155
4.134
3.990
3.962
3.854
3.645
3.616
3.359
3.245
3.224
3.202
2.490
2.417
2.416
2.383
2.136
2.113
2.082
1.473
1.456
1.019
HO
NH
N
S
tBu
NH
O
N
N
O
H
H2N
N
z0z-216-10
1.00
0.81
1.70
0.85
1.05
12.23
1.87
0.76
1.00
1.78
1.78
1.05
1.12
0.97
1.83
1.00
1.83
1.97
1.02
1.93
7.21
1.45
2.96
9.28
f1 (ppm)

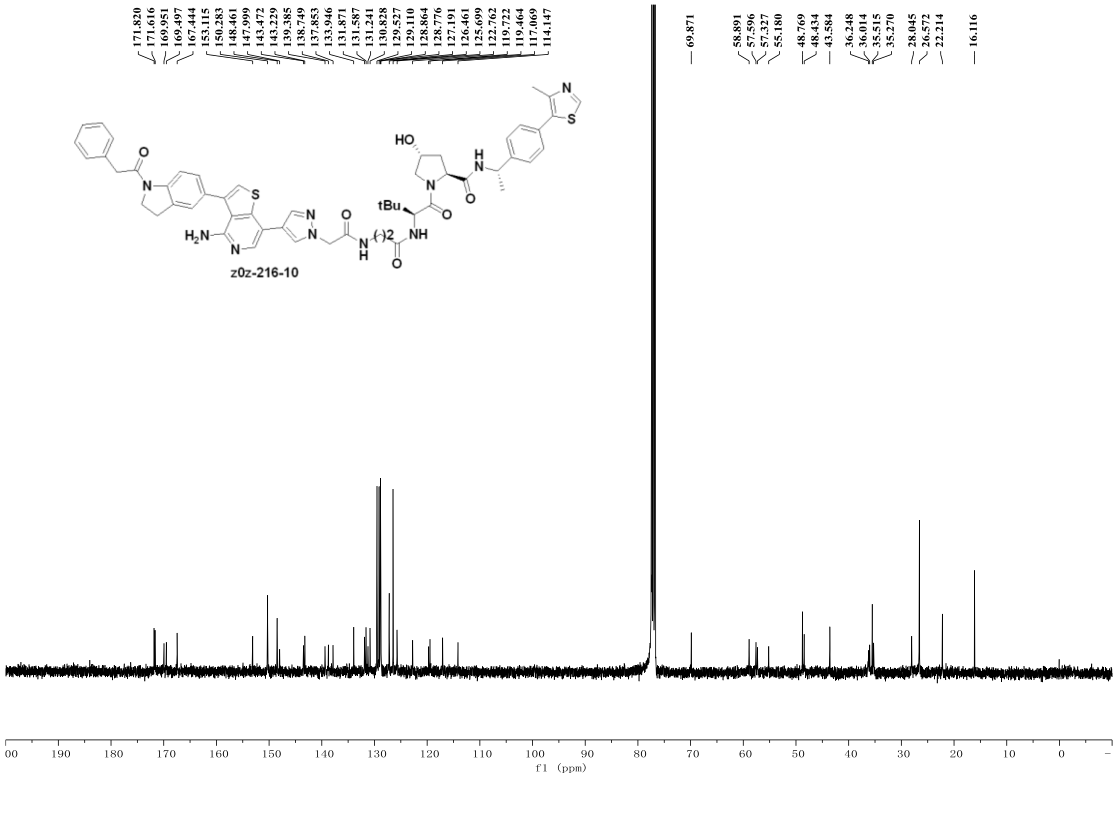
171.820
171.616
169.951
169.497
167.444
153.115
150.283
148.461
147.999
143.472
143.229
139.385
138.749
137.853
133.946
131.871
131.587
131.241
130.828
129.527
129.110
128.864
128.776
127.191
126.461
125.699
122.762
119.722
119.464
117.069
114.147
69.871
58.891
57.596
57.327
55.180
48.769
48.434
43.584
36.248
36.014
35.515
35.270
28.045
26.572
22.214
16.116
z0z-216-10
f1 (ppm)

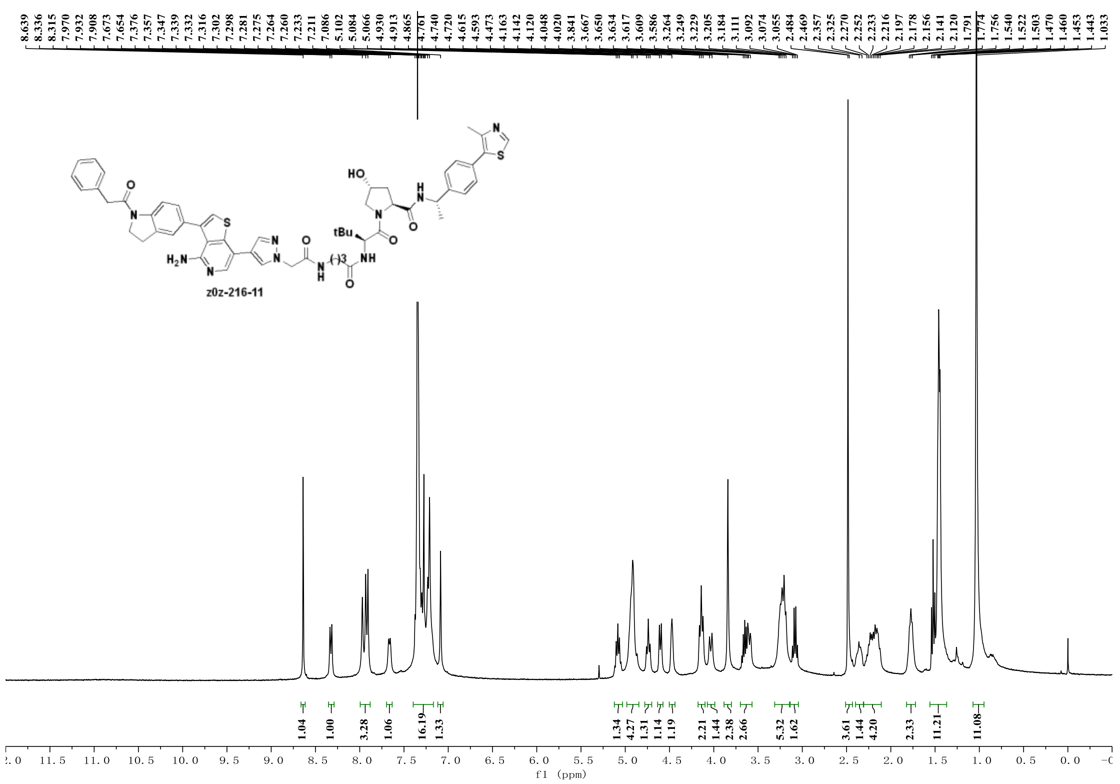

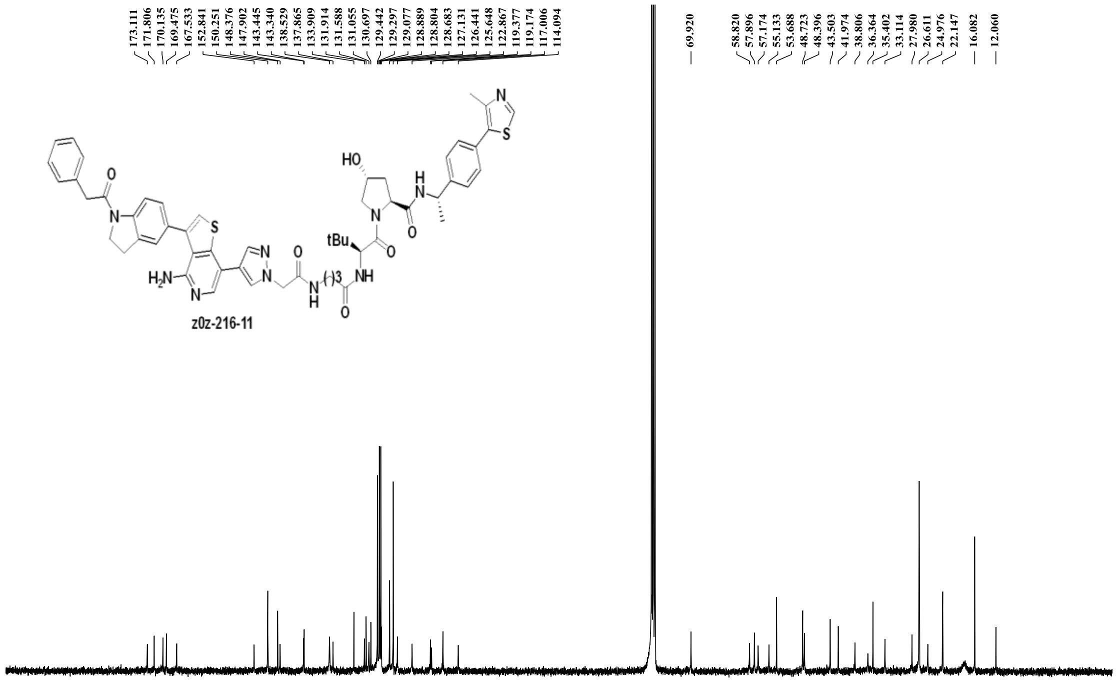

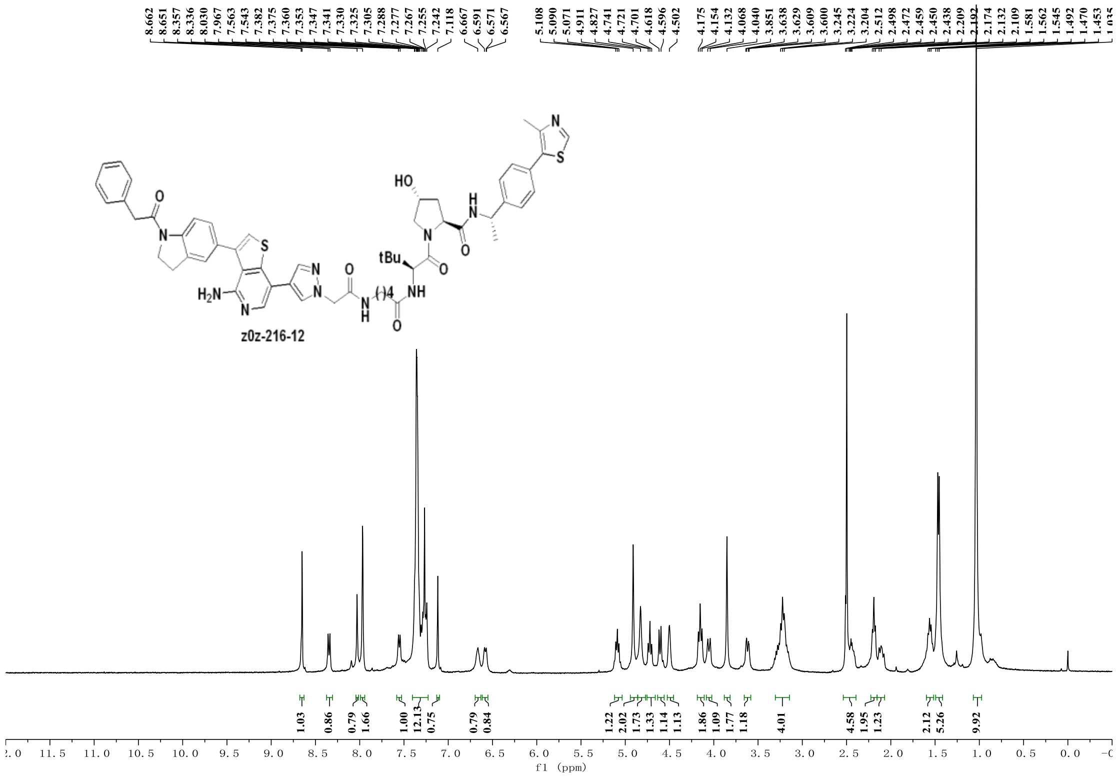

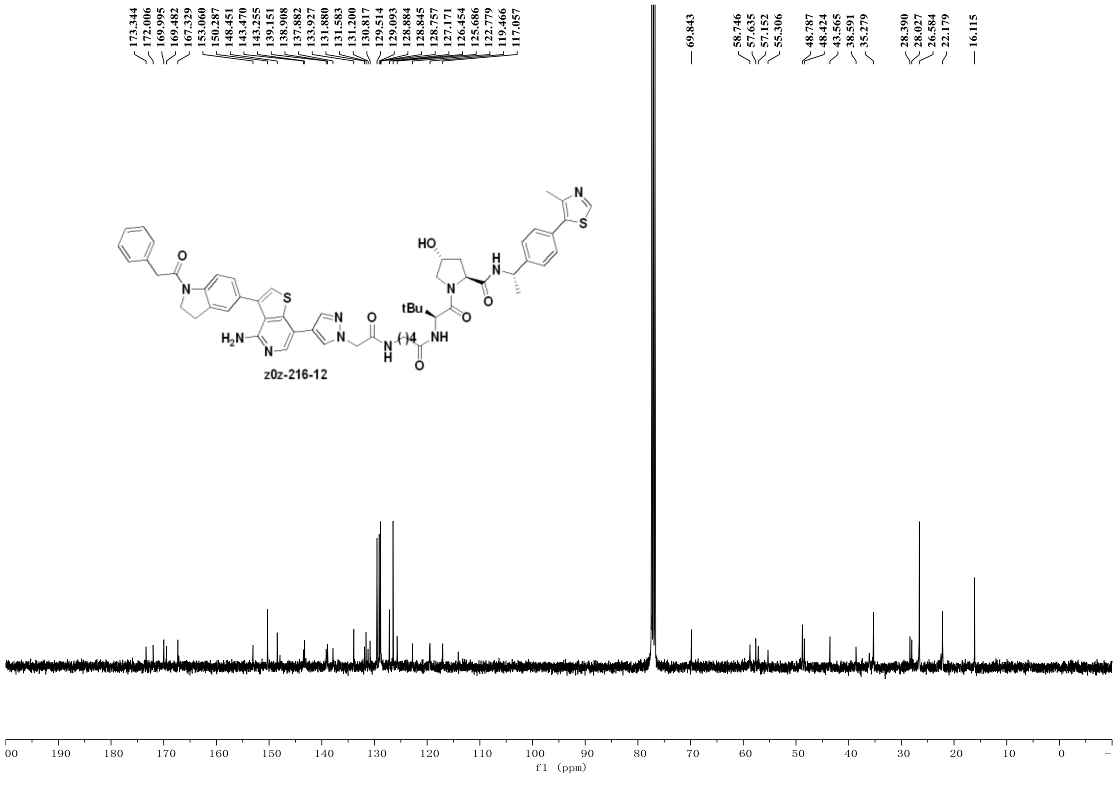

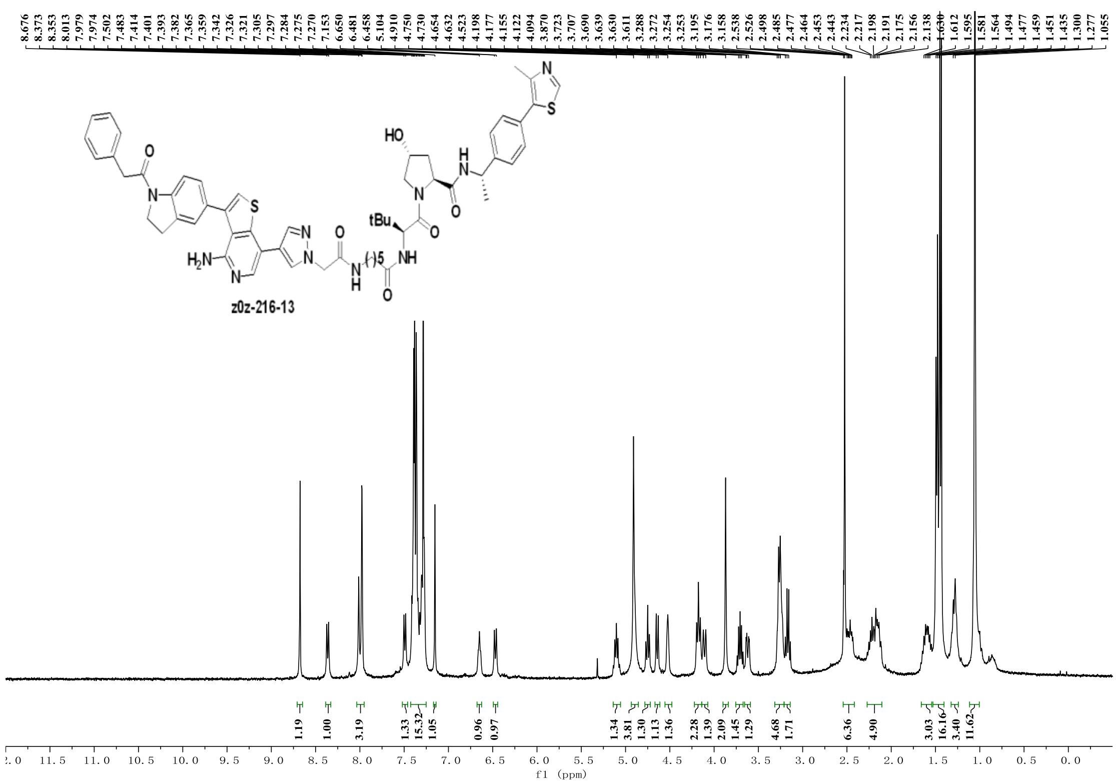
8.676 8.373 8.353 8.013 7.979 7.974 7.502 7.483 7.414 7.401 7.393 7.382 7.365 7.359 7.342 7.326 7.321 7.305 7.297 7.284 7.275 7.270 7.153 6.650 6.481 6.458 5.104 4.910 4.750 4.730 4.654 4.632 4.523 4.198 4.177 4.155 4.122 4.094 3.870 3.723 3.707 3.690 3.639 3.630 3.611 3.288 3.272 3.254 3.253 3.195 3.176 3.158 2.538 2.526 2.498 2.485 2.477 2.464 2.453 2.443 2.234 2.217 2.198 2.191 2.175 2.156 2.138 1.630 1.612 1.595 1.581 1.564 1.494 1.477 1.459 1.451 1.435 1.300 1.277 1.055
z0z-216-13
1.19 1.00 3.19 1.33 15.32 1.05 0.96 0.97 1.34 3.81 1.30 1.13 1.36 2.28 1.39 2.09 1.45 1.29 4.68 1.71 6.36 4.90 3.03 16.16 3.40 11.62
f1 (ppm)

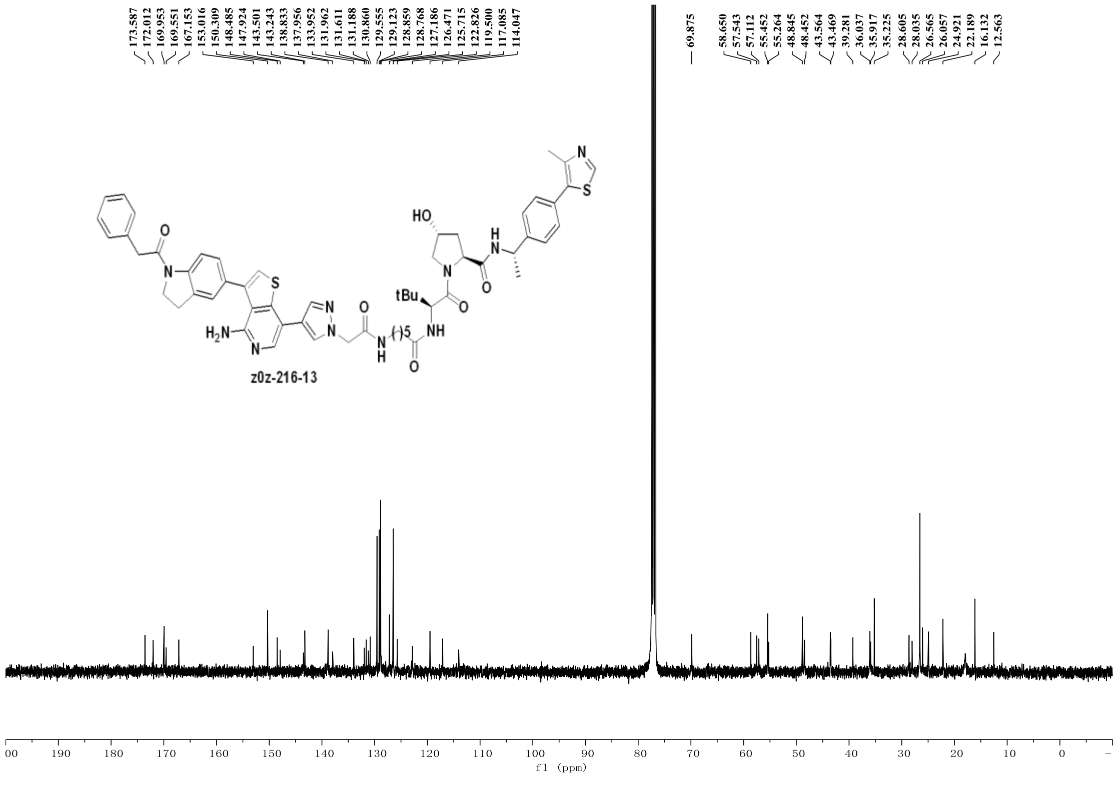
173.587 172.012 169.953 169.551 167.153 153.016 150.309 148.485 147.924 143.501 143.243 138.833 137.956 133.952 131.962 131.611 131.188 130.860 129.555 129.123 128.859 128.768 127.186 126.471 125.715 122.826 119.500 117.085 114.047
69.875
58.650 57.543 57.112 55.452 55.264 48.845 48.452 43.564 43.469 39.281 36.037 35.917 35.225 28.605 28.035 26.565 26.057 24.921 22.189 16.132 12.563
z0z-216-13
f1 (ppm)

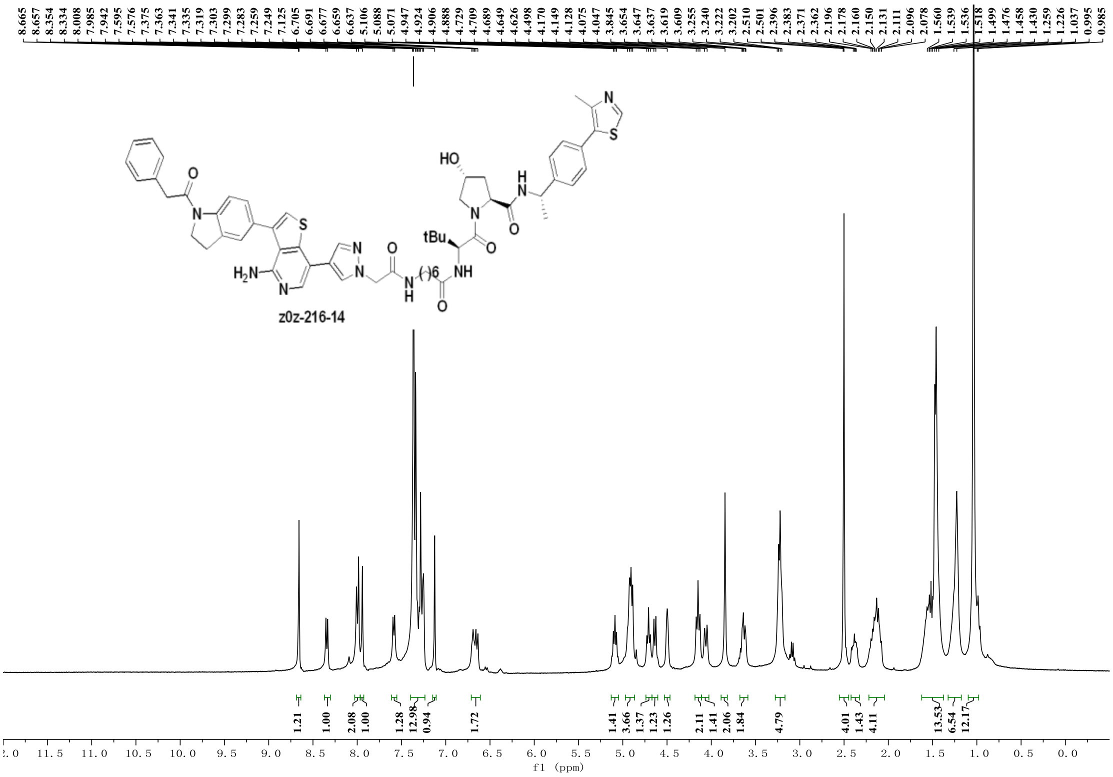
8.665 8.657 8.354 8.334 8.008 7.985 7.942 7.595 7.576 7.375 7.363 7.341 7.335 7.319 7.303 7.299 7.283 7.259 7.249 7.125 6.705 6.691 6.677 6.659 6.637 5.106 5.088 5.071 4.947 4.924 4.906 4.888 4.729 4.709 4.689 4.649 4.626 4.498 4.170 4.149 4.128 4.075 4.047 3.845 3.654 3.647 3.637 3.619 3.609 3.255 3.240 3.222 3.202 2.510 2.501 2.396 2.383 2.371 2.362 2.196 2.178 2.160 2.150 2.131 2.111 2.096 2.078 1.560 1.539 1.536 1.518 1.499 1.476 1.458 1.430 1.259 1.226 1.037 0.995 0.985
z0z-216-14
1.21 1.00 2.08 1.00 1.28 12.98 0.94 1.72 1.41 3.66 1.37 1.23 1.26 2.11 1.41 2.06 1.84 4.79 4.01 1.43 4.11 13.53 6.54 12.17
f1 (ppm)

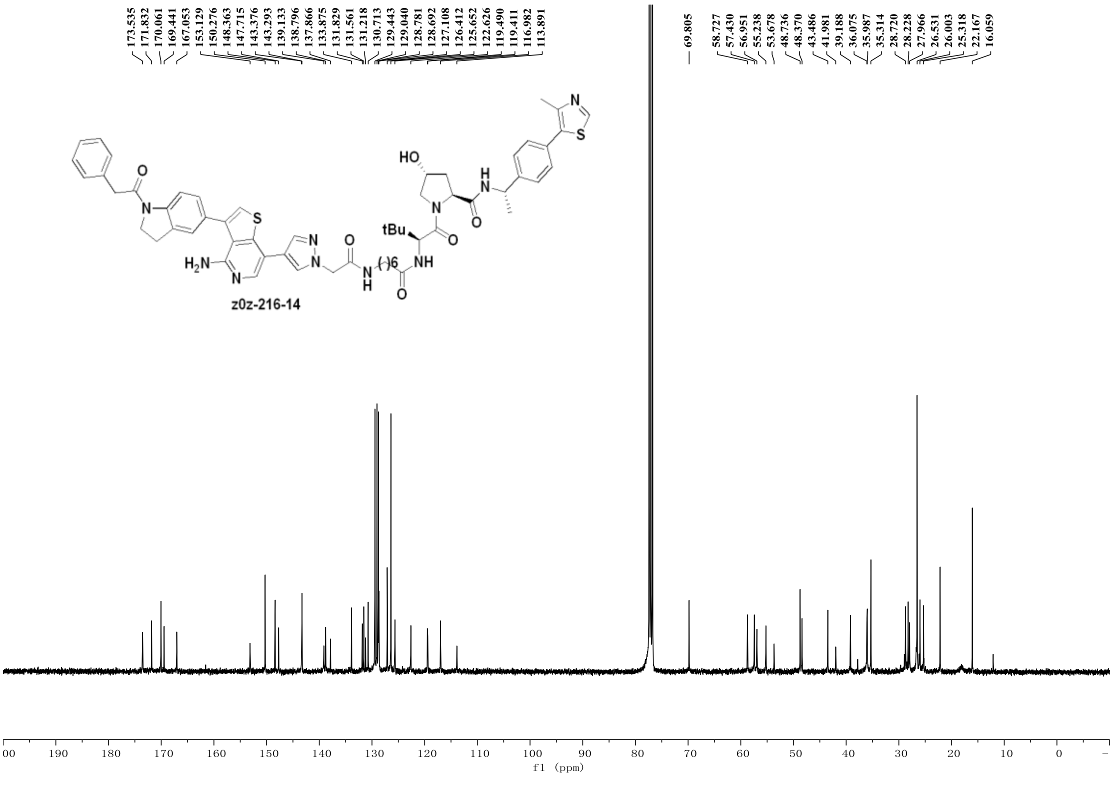
173.535 171.832 170.061 169.441 167.053 153.129 150.276 148.363 147.715 143.376 143.293 139.133 138.796 137.866 133.875 131.829 131.561 131.218 130.713 129.443 129.040 128.781 128.692 127.108 126.412 125.652 122.626 119.490 119.411 116.982 113.891
69.805
58.727 57.430 56.951 55.238 53.678 48.736 48.370 43.486 41.981 39.188 36.075 35.987 35.314 28.720 28.228 27.966 26.531 26.003 25.318 22.167 16.059
z0z-216-14
f1 (ppm)

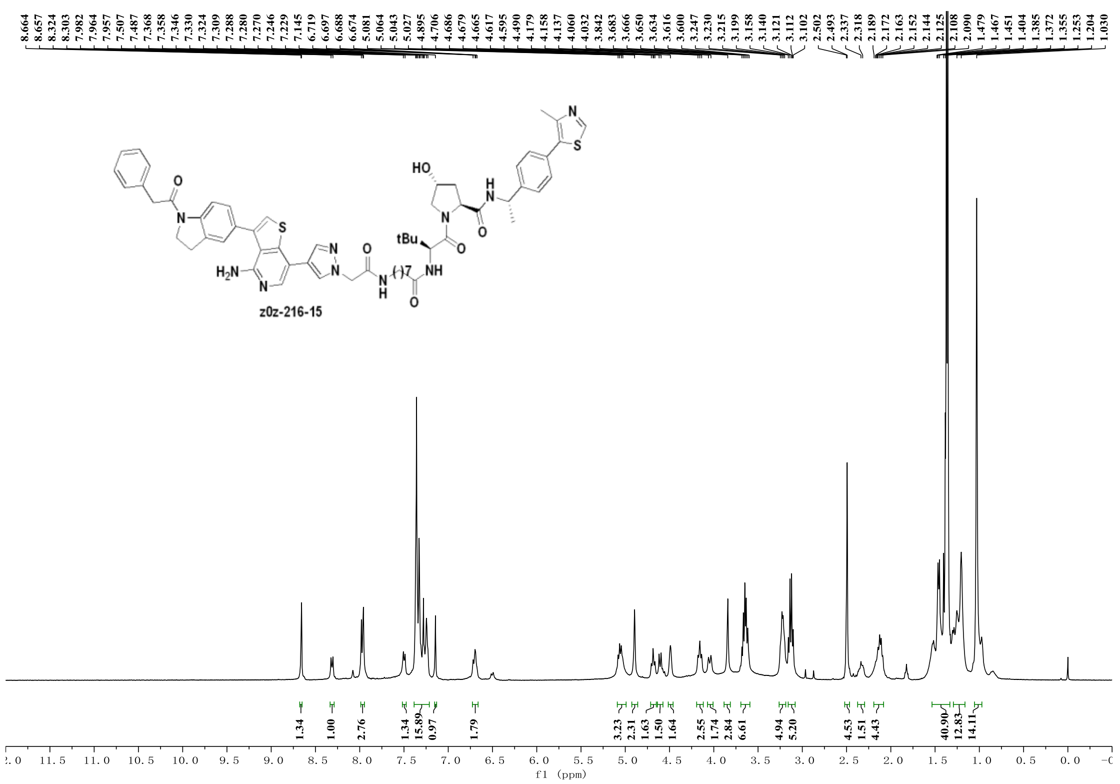
8.664 8.657 8.324 8.303 7.982 7.964 7.957 7.507 7.487 7.368 7.358 7.346 7.330 7.324 7.309 7.288 7.280 7.270 7.246 7.229 7.145 6.719 6.697 6.688 6.674 5.081 5.064 5.043 5.027 4.895 4.706 4.686 4.679 4.665 4.617 4.595 4.490 4.179 4.158 4.137 4.060 4.032 3.842 3.683 3.666 3.650 3.634 3.616 3.600 3.247 3.230 3.215 3.199 3.158 3.140 3.121 3.112 3.102 2.502 2.493 2.337 2.318 2.189 2.172 2.163 2.152 2.144 2.125 2.108 2.090 1.479 1.467 1.451 1.404 1.385 1.372 1.355 1.253 1.204 1.030
z0z-216-15
1.34 1.00 2.76 1.34 15.89 0.97 1.79 3.23 2.31 1.63 1.50 1.64 2.55 1.74 2.84 6.61 4.94 5.20 4.53 1.51 4.43 40.90 12.83 14.11
f1 (ppm)

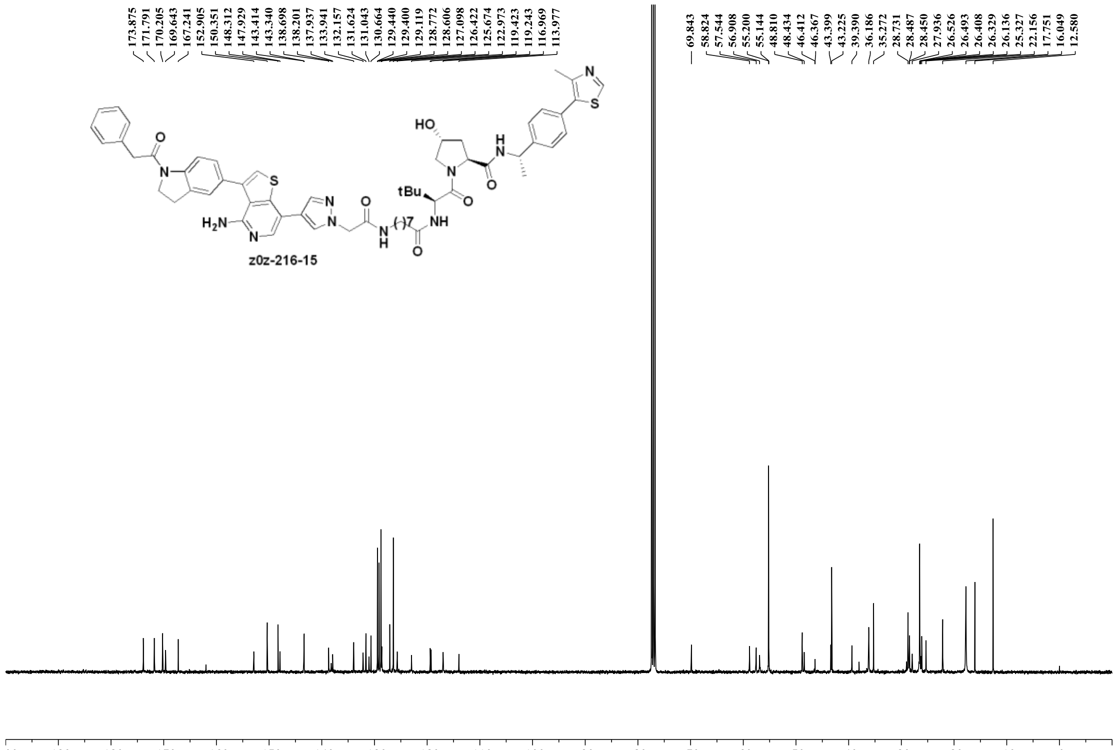
173.875 171.791 170.205 169.643 167.241 152.905 150.351 148.312 147.929 143.414 143.340 138.698 138.201 137.937 133.941 132.157 131.624 131.043 130.664 129.440 129.400 129.119 128.772 128.606 127.098 126.422 125.674 122.973 119.423 119.243 116.969 113.977
69.843 58.824 57.544 56.908 55.200 55.144 48.810 48.434 46.412 46.367 43.399 43.225 39.390 36.186 35.272 28.731 28.487 28.450 27.936 26.526 26.493 26.408 26.329 26.136 25.327 22.156 17.751 16.049 12.580
z0z-216-15
f1 (ppm)

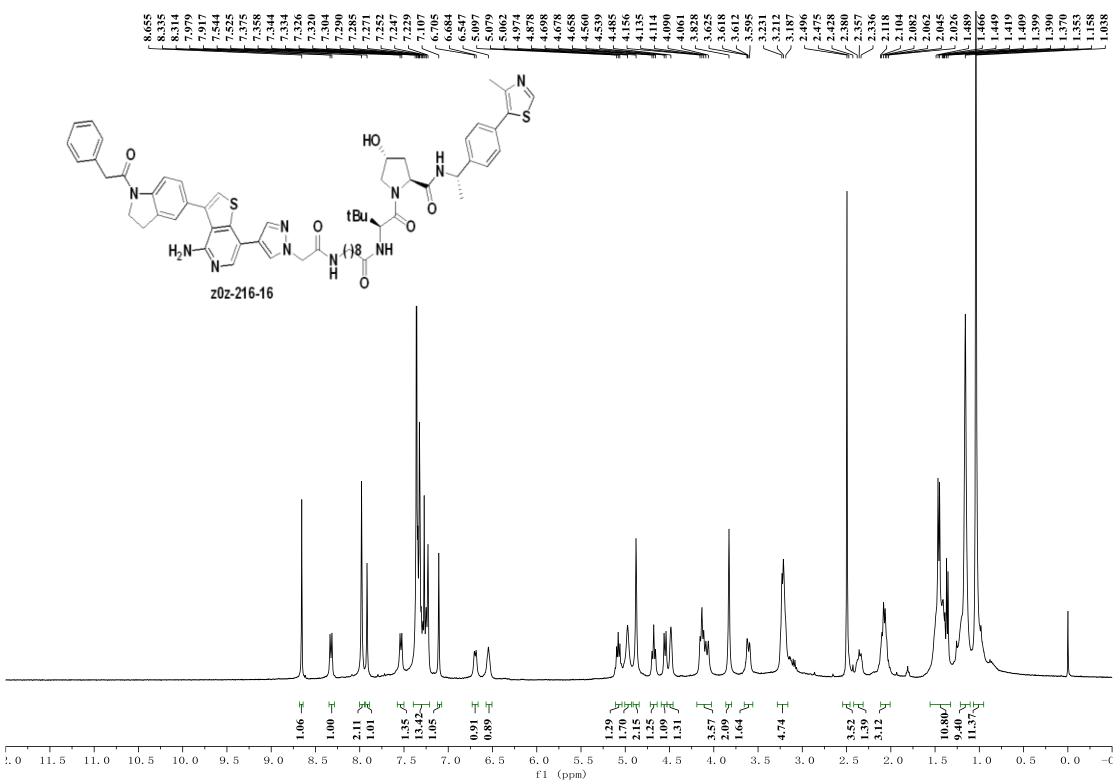
8.655 8.335 8.314 7.979 7.917 7.544 7.525 7.375 7.358 7.344 7.334 7.326 7.320 7.304 7.290 7.285 7.271 7.252 7.247 7.229 7.107 6.705 6.684 6.547 5.097 5.079 5.062 4.974 4.878 4.698 4.678 4.658 4.560 4.539 4.485 4.156 4.135 4.114 4.090 4.061 3.828 3.625 3.618 3.612 3.595 3.231 3.212 3.187 2.496 2.475 2.428 2.380 2.357 2.336 2.118 2.104 2.082 2.062 2.045 2.026 1.489 1.466 1.449 1.419 1.409 1.399 1.390 1.370 1.353 1.158 1.038
z0z-216-16
1.06 1.00 2.11 1.01 1.35 13.42 1.05 0.91 0.89 1.29 1.70 2.15 1.25 1.09 1.31 3.57 2.09 1.64 4.74 3.52 1.39 3.12 10.80 9.40 11.37
f1 (ppm)

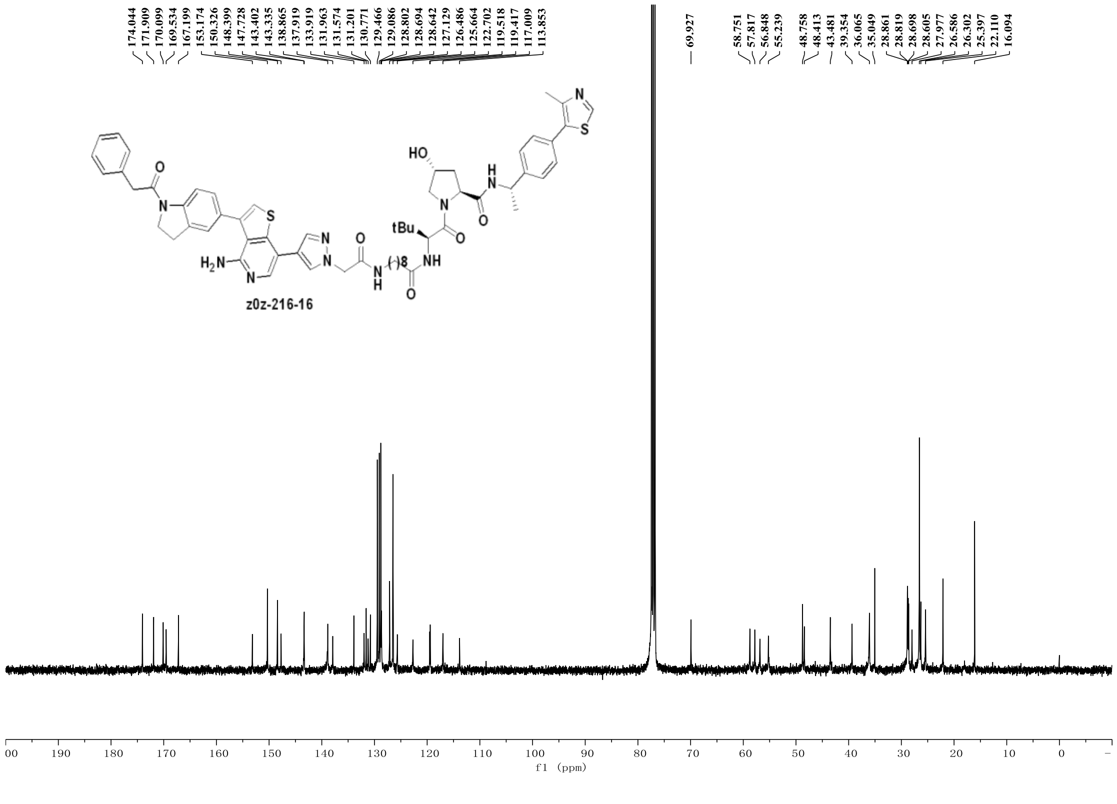
174.044 171.909 170.099 169.534 167.199 153.174 150.326 148.399 147.728 143.402 143.335 138.865 137.919 133.919 131.963 131.574 131.201 130.771 129.466 129.086 128.802 128.694 128.642 127.129 126.486 125.664 122.702 119.518 119.417 117.009 113.853
69.927
58.751 57.817 56.848 55.239 48.758 48.413 43.481 39.354 36.065 35.049 28.861 28.819 28.698 28.605 27.977 26.586 26.302 25.397 22.110 16.094
z0z-216-16
f1 (ppm)

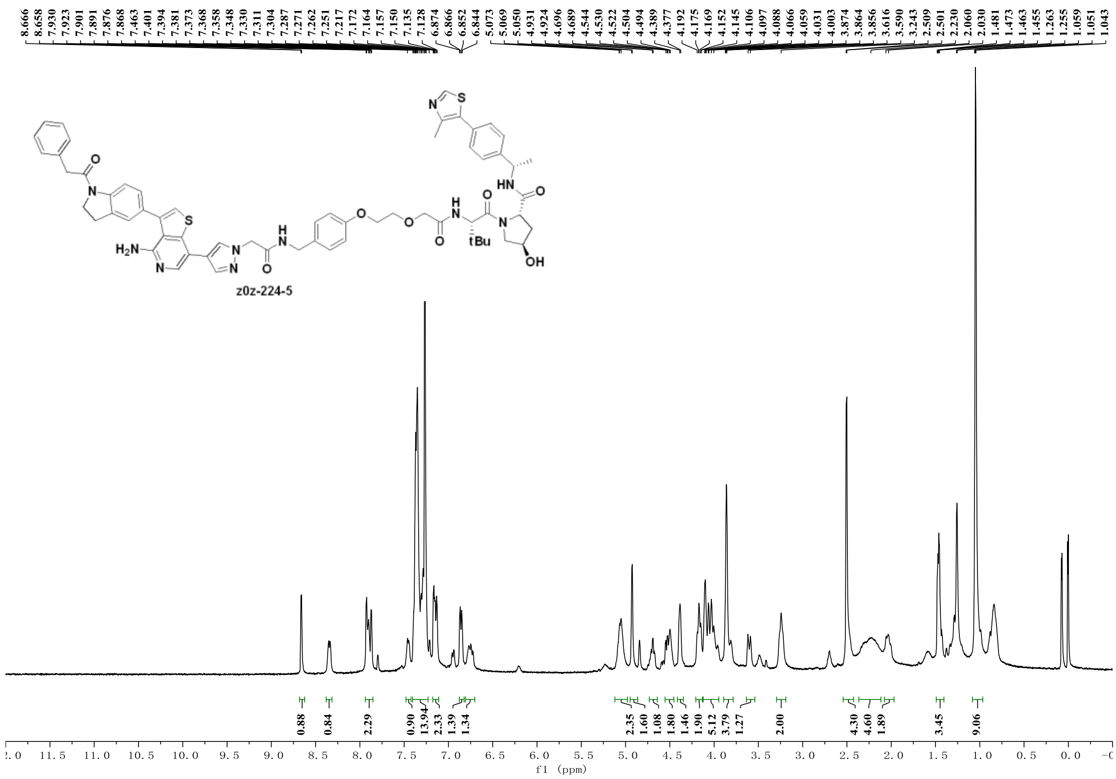
8.666 8.658 7.930 7.923 7.901 7.891 7.876 7.868 7.463 7.401 7.394 7.381 7.373 7.368 7.358 7.348 7.330 7.311 7.304 7.287 7.271 7.262 7.251 7.217 7.172 7.164 7.157 7.150 7.135 7.128 6.874 6.866 6.852 6.844 5.073 5.069 5.050 4.931 4.924 4.696 4.689 4.544 4.530 4.522 4.504 4.494 4.389 4.377 4.192 4.175 4.169 4.152 4.145 4.106 4.097 4.088 4.066 4.059 4.031 4.003 3.874 3.864 3.856 3.616 3.590 3.243 2.509 2.501 2.230 2.060 2.030 1.481 1.473 1.463 1.455 1.263 1.255 1.059 1.051 1.043
z0z-224-5
0.88 0.84 2.29 0.90 13.94 2.33 1.39 1.34 2.35 1.60 1.08 1.80 1.46 1.90 5.12 3.79 1.27 2.00 4.30 4.60 1.89 3.45 9.06
f1 (ppm)

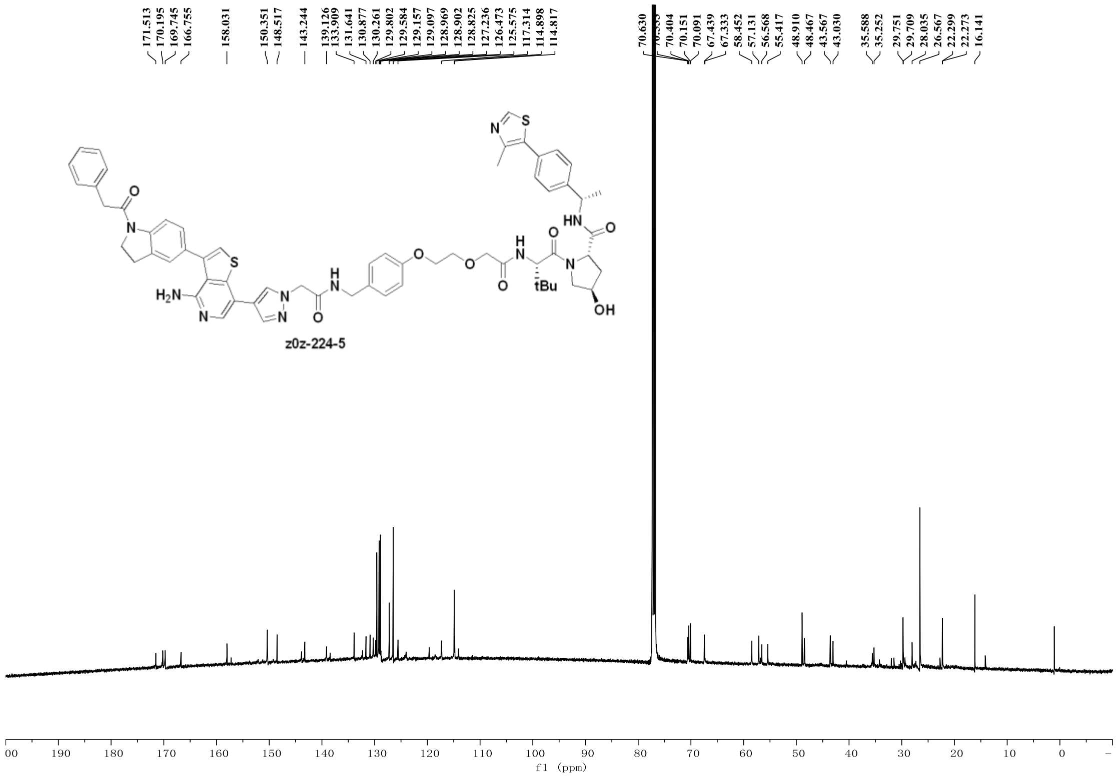
171.513 170.195 169.745 166.755 158.031 150.351 148.517 143.244 139.126 133.909 131.641 130.877 130.261 129.802 129.584 129.157 129.097 128.969 128.902 128.825 127.236 126.473 125.575 117.314 114.898 114.817
70.630 70.404 70.151 70.091 67.439 67.333 58.452 57.131 56.568 55.417 48.910 48.467 43.567 43.030 35.588 35.252 29.751 29.709 28.035 26.567 22.299 22.273 16.141
z0z-224-5
f1 (ppm)

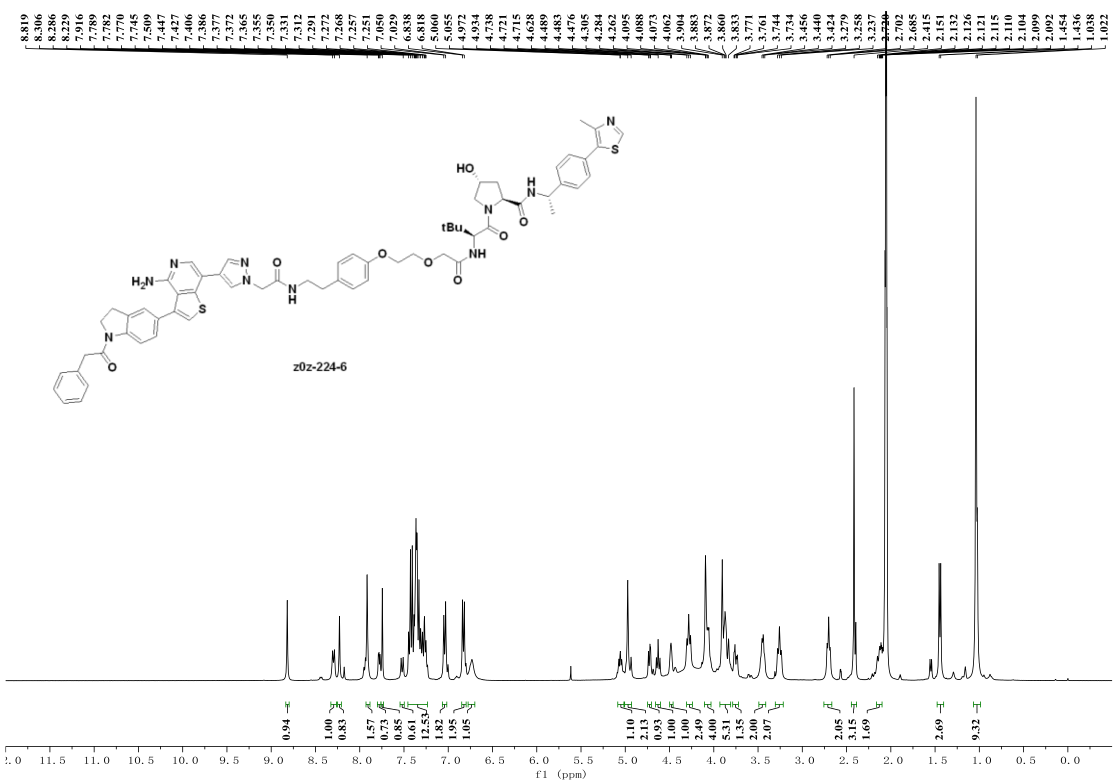
8.819
8.306
8.286
8.229
7.916
7.789
7.782
7.770
7.745
7.509
7.447
7.427
7.406
7.386
7.377
7.372
7.365
7.355
7.350
7.331
7.312
7.291
7.272
7.268
7.257
7.251
7.050
7.029
6.838
6.818
5.060
5.055
4.972
4.934
4.738
4.721
4.715
4.628
4.489
4.483
4.476
4.305
4.284
4.262
4.095
4.088
4.073
4.062
3.904
3.883
3.872
3.860
3.833
3.771
3.761
3.744
3.734
3.456
3.440
3.424
3.279
3.258
3.237
2.702
2.685
2.415
2.151
2.132
2.126
2.121
2.115
2.110
2.104
2.099
2.092
1.454
1.436
1.038
1.022
HO
N
NH
O
tBu
O
NH
O
O
O
N
S
H2N
N
N
N
O
N
H
S
N
O
z0z-224-6
0.94
1.00
0.83
1.57
0.73
0.85
0.61
12.53
1.82
1.95
1.05
1.10
2.13
0.93
1.00
1.00
2.49
4.00
5.31
1.35
2.00
2.07
2.05
3.15
1.69
2.69
9.32
11.5 11.0 10.5 10.0 9.5 9.0 8.5 8.0 7.5 7.0 6.5 6.0 5.5 5.0 4.5 4.0 3.5 3.0 2.5 2.0 1.5 1.0 0.5 0.0
f1 (ppm)

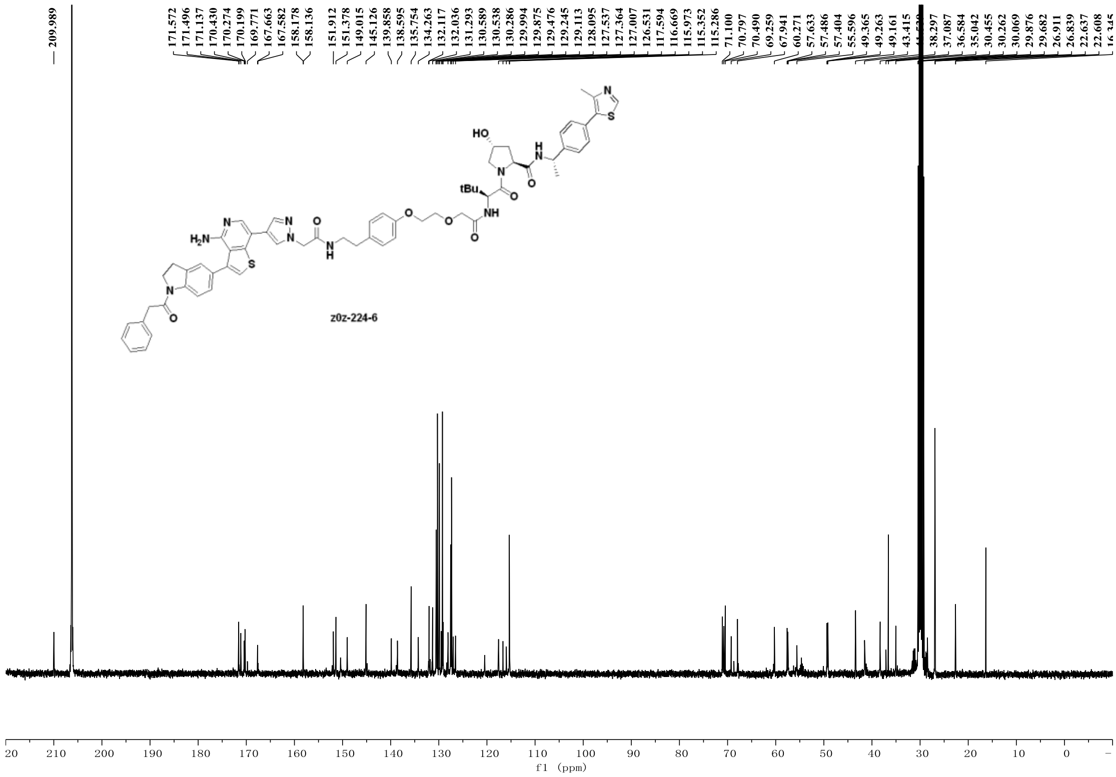
209.989
171.572
171.496
171.137
170.430
170.274
170.199
169.771
167.663
167.582
158.178
158.136
151.912
151.378
149.015
145.126
139.858
138.595
135.754
134.263
132.117
132.036
131.293
130.589
130.538
130.286
129.994
129.875
129.476
129.245
129.113
128.095
127.537
127.364
127.007
126.531
117.594
116.669
115.973
115.352
115.286
71.100
70.797
70.490
69.259
67.941
60.271
57.633
57.486
57.404
55.596
49.365
49.263
49.161
43.415
38.297
37.087
36.584
35.042
30.455
30.262
30.069
29.876
29.682
26.911
26.839
22.637
22.608
z0z-224-6
210 200 190 180 170 160 150 140 130 120 110 100 90 80 70 60 50 40 30 20 10 0
f1 (ppm)

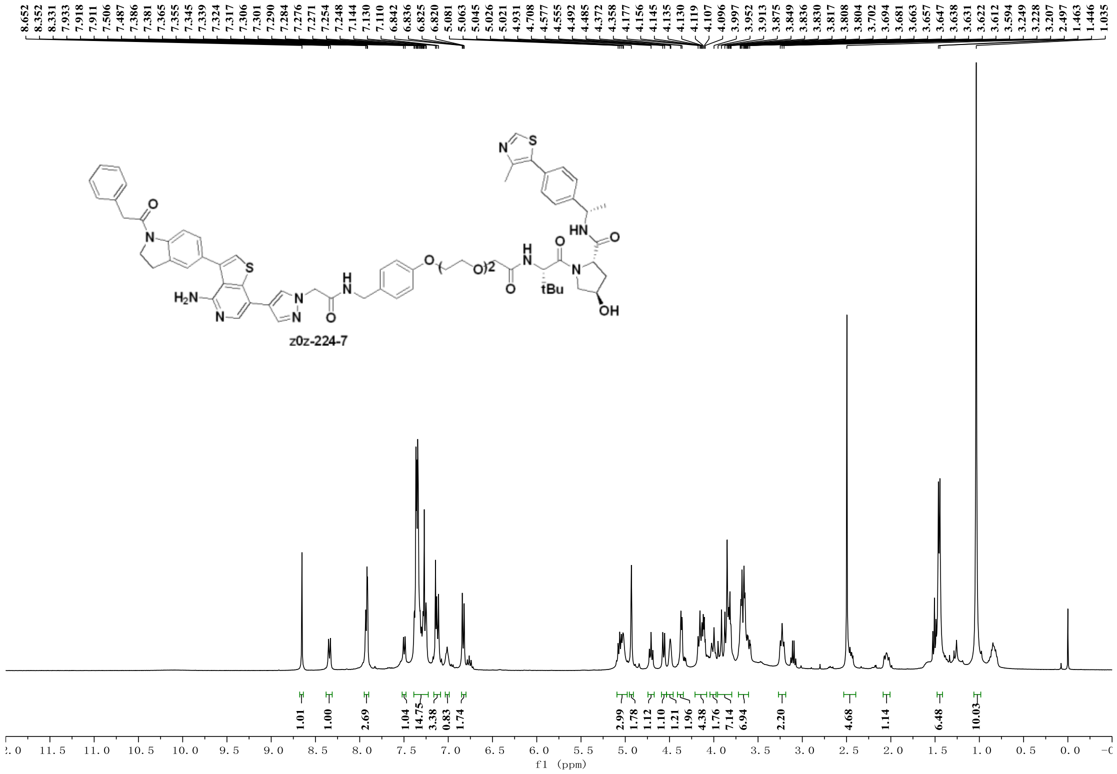
8.652 8.352 8.331 7.933 7.918 7.911 7.506 7.487 7.386 7.381 7.365 7.355 7.345 7.339 7.324 7.317 7.306 7.301 7.290 7.284 7.276 7.271 7.254 7.248 7.144 7.130 7.110 6.842 6.836 6.825 6.820 5.081 5.063 5.045 5.026 5.021 4.931 4.708 4.577 4.555 4.492 4.485 4.372 4.358 4.177 4.156 4.145 4.135 4.130 4.119 4.107 4.096 3.997 3.952 3.913 3.875 3.849 3.836 3.830 3.817 3.808 3.804 3.702 3.694 3.681 3.663 3.657 3.647 3.638 3.631 3.622 3.612 3.594 3.249 3.228 3.207 2.497 1.463 1.446 1.035
z0z-224-7
1.01 1.00 2.69 1.04 14.75 3.38 0.83 1.74 2.99 1.78 1.12 1.10 1.21 1.96 4.38 1.76 7.14 6.94 2.20 4.68 1.14 6.48 10.03
f1 (ppm)

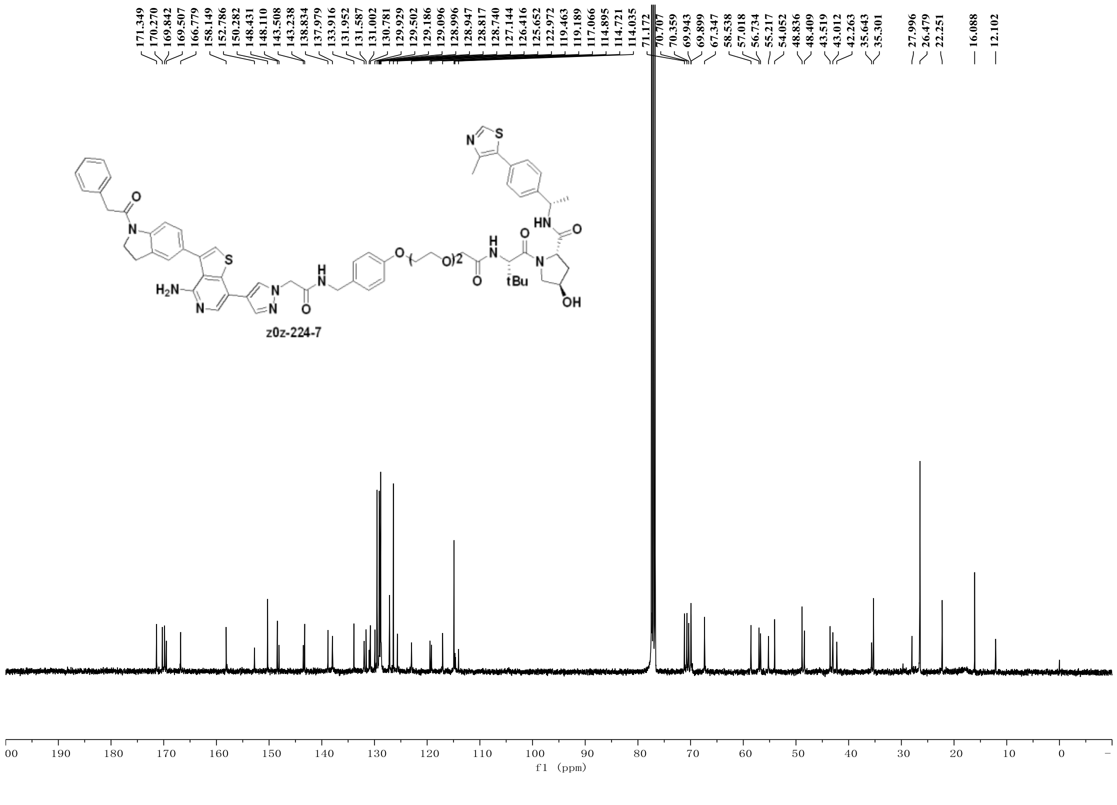
171.349 170.270 169.842 169.507 166.779 158.149 152.786 150.282 148.431 148.110 143.508 143.238 138.834 137.979 133.916 131.952 131.587 131.002 130.781 129.929 129.502 129.186 129.096 128.996 128.947 128.817 128.740 127.144 126.416 125.652 122.972 119.463 119.189 117.066 114.895 114.721 114.035 71.172 70.707 70.359 69.943 69.899 67.347 58.538 57.018 56.734 55.217 54.052 48.836 48.409 43.519 43.012 42.263 35.643 35.301 27.996 26.479 22.251 16.088 12.102
z0z-224-7
f1 (ppm)

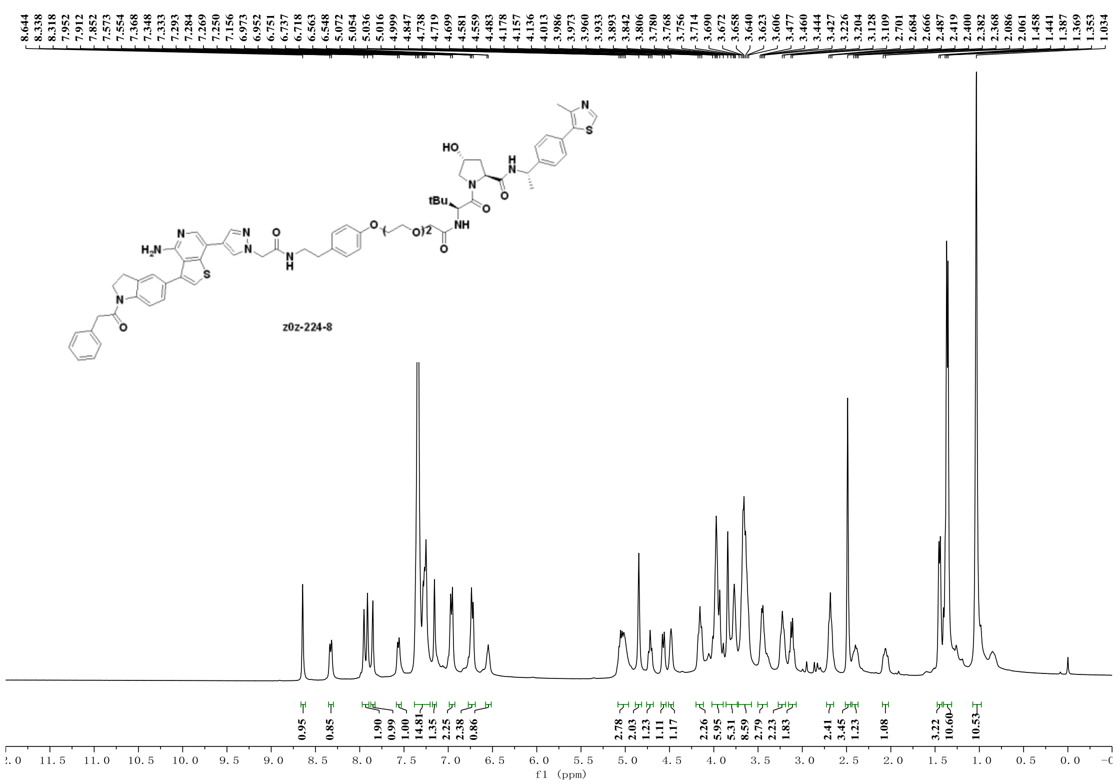
8.644
8.338
8.318
7.952
7.912
7.852
7.573
7.554
7.368
7.348
7.333
7.293
7.284
7.269
7.250
7.156
6.973
6.952
6.751
6.737
6.718
6.563
6.548
5.072
5.054
5.036
5.016
4.999
4.847
4.738
4.719
4.699
4.581
4.559
4.483
4.178
4.157
4.136
4.013
3.986
3.973
3.960
3.933
3.893
3.842
3.806
3.780
3.768
3.756
3.714
3.690
3.672
3.658
3.640
3.623
3.606
3.477
3.460
3.444
3.427
3.226
3.204
3.128
3.109
2.701
2.684
2.666
2.487
2.419
2.400
2.382
2.368
2.086
2.061
1.458
1.441
1.387
1.369
1.353
1.034
HO
tBu
NH
H2N
z0z-224-8
0.95
0.85
1.90
0.99
1.00
14.81
1.35
2.25
2.38
0.86
2.78
2.03
1.23
1.11
1.17
2.26
5.95
5.31
8.59
2.79
2.23
1.83
2.41
3.45
1.23
1.08
3.22
10.60
10.53
f1 (ppm)

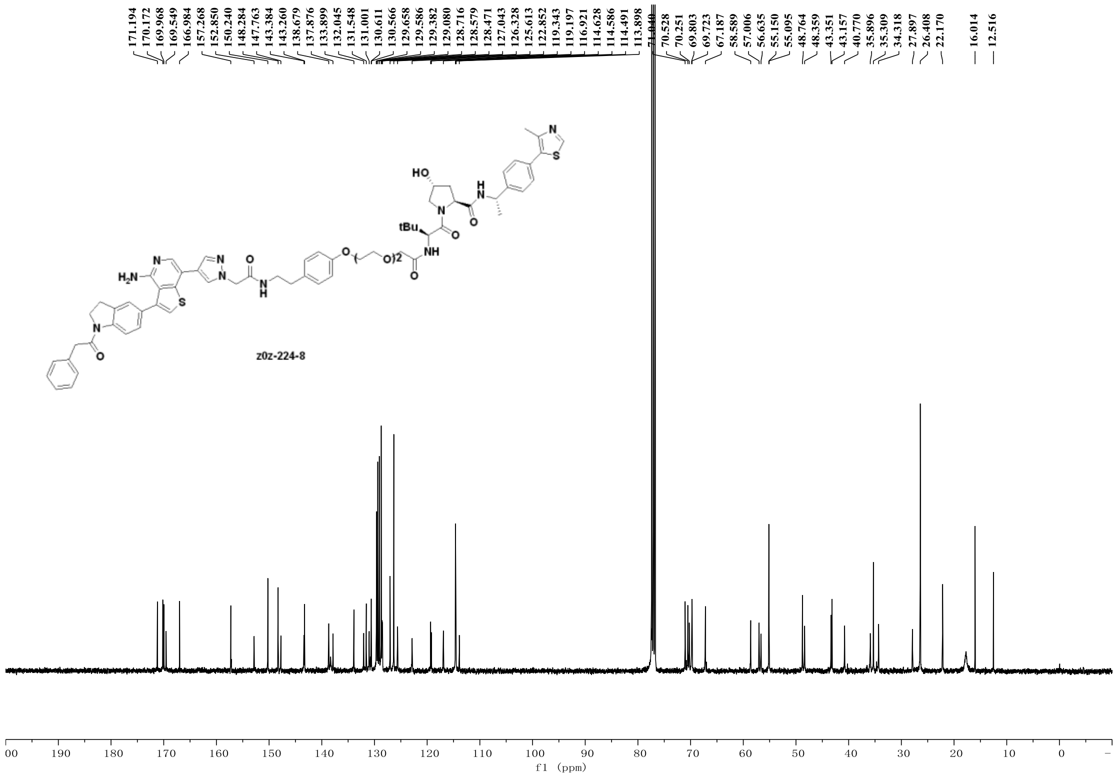
171.194
170.172
169.968
169.549
166.984
157.268
152.850
150.240
148.284
147.763
143.384
143.260
138.679
137.876
133.899
132.045
131.548
131.001
130.611
130.566
129.658
129.586
129.382
129.080
128.716
128.579
128.471
127.043
126.328
125.613
122.852
119.343
119.197
116.921
114.628
114.586
114.491
113.898
70.528
70.251
69.803
69.723
67.187
58.589
57.006
56.635
55.150
55.095
48.764
48.359
43.351
43.157
40.770
35.896
35.309
34.318
27.897
26.408
22.170
16.014
12.516
HO
tBu
NH
H2N
z0z-224-8
f1 (ppm)

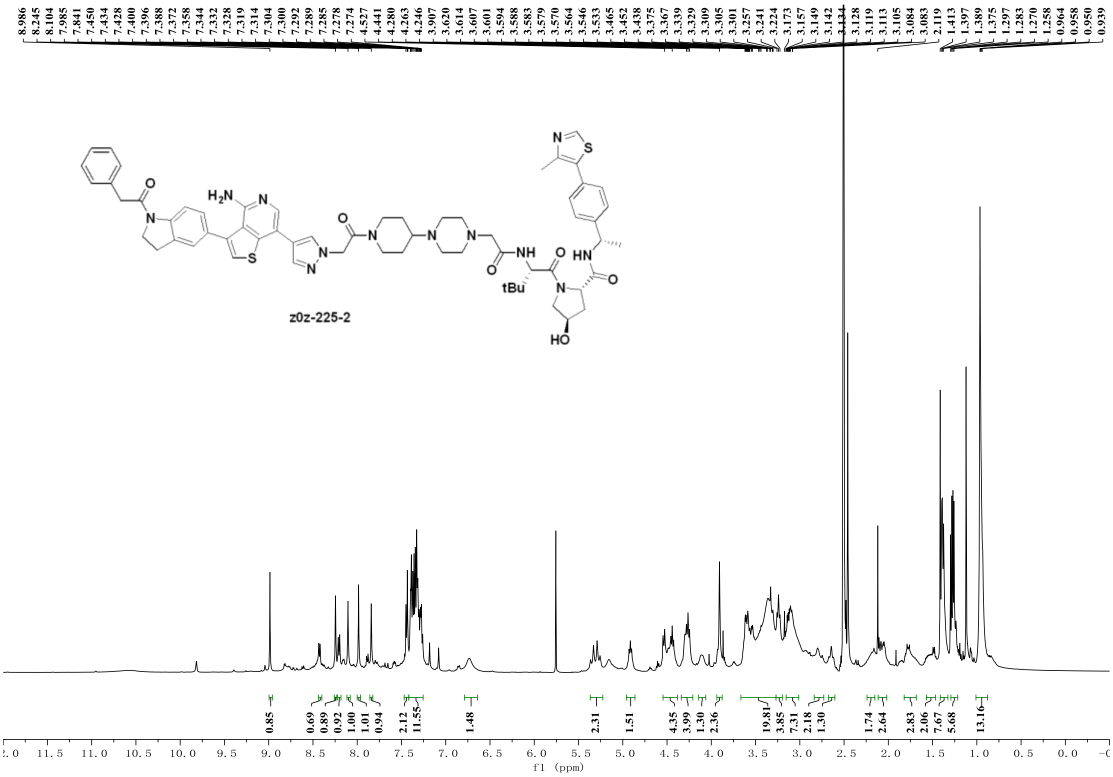
8.986 8.245 8.104 7.985 7.841 7.450 7.434 7.428 7.400 7.396 7.388 7.372 7.358 7.344 7.332 7.328 7.319 7.314 7.304 7.300 7.292 7.289 7.285 7.278 7.274 4.527 4.441 4.280 4.263 4.246 3.907 3.620 3.614 3.607 3.601 3.594 3.588 3.583 3.579 3.570 3.564 3.546 3.533 3.465 3.452 3.438 3.375 3.367 3.339 3.329 3.309 3.305 3.301 3.257 3.241 3.224 3.173 3.157 3.149 3.142 3.128 3.119 3.113 3.105 3.084 3.083 2.119 1.413 1.397 1.389 1.375 1.297 1.283 1.270 1.258 0.964 0.958 0.950 0.939
H2N
N
S
O
N
tBu
NH
HN
HO
z0z-225-2
0.85 0.69 0.89 0.92 1.00 1.01 0.94 2.12 11.55 1.48 2.31 1.51 4.35 3.99 1.30 2.36 19.81 3.85 7.31 2.18 1.30 1.74 2.64 2.83 2.06 7.67 5.68 13.16
11.5 11.0 10.5 10.0 9.5 9.0 8.5 8.0 7.5 7.0 6.5 6.0 5.5 5.0 4.5 4.0 3.5 3.0 2.5 2.0 1.5 1.0 0.5 0.0
f1 (ppm)

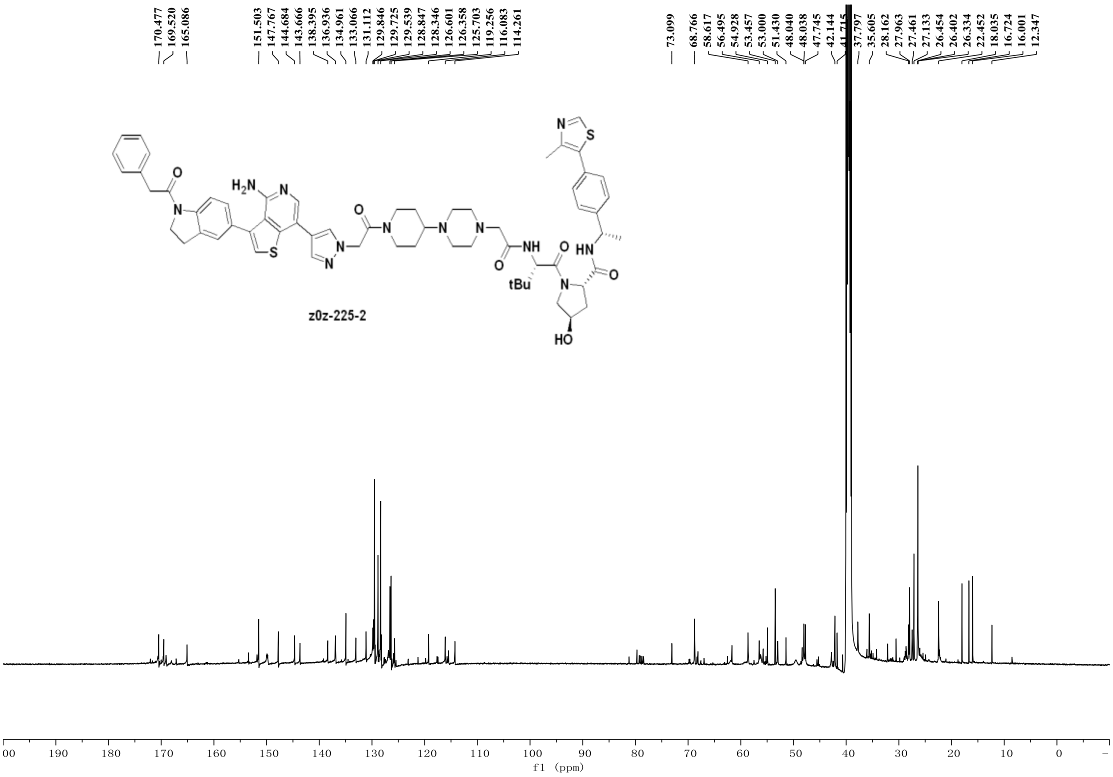
170.477 169.520 165.086 151.503 147.767 144.684 143.666 138.395 136.936 134.961 133.066 131.112 129.846 129.725 129.539 128.847 128.346 126.601 126.358 125.703 119.256 116.083 114.261
73.099 68.766 58.617 56.495 54.928 53.457 53.000 51.430 48.040 48.038 47.745 42.144 37.797 35.605 28.162 27.963 27.461 27.133 26.454 26.402 26.334 22.452 18.035 16.724 16.001 12.347
H2N
N
S
O
N
tBu
NH
HN
HO
z0z-225-2
190 180 170 160 150 140 130 120 110 100 90 80 70 60 50 40 30 20 10 0
f1 (ppm)

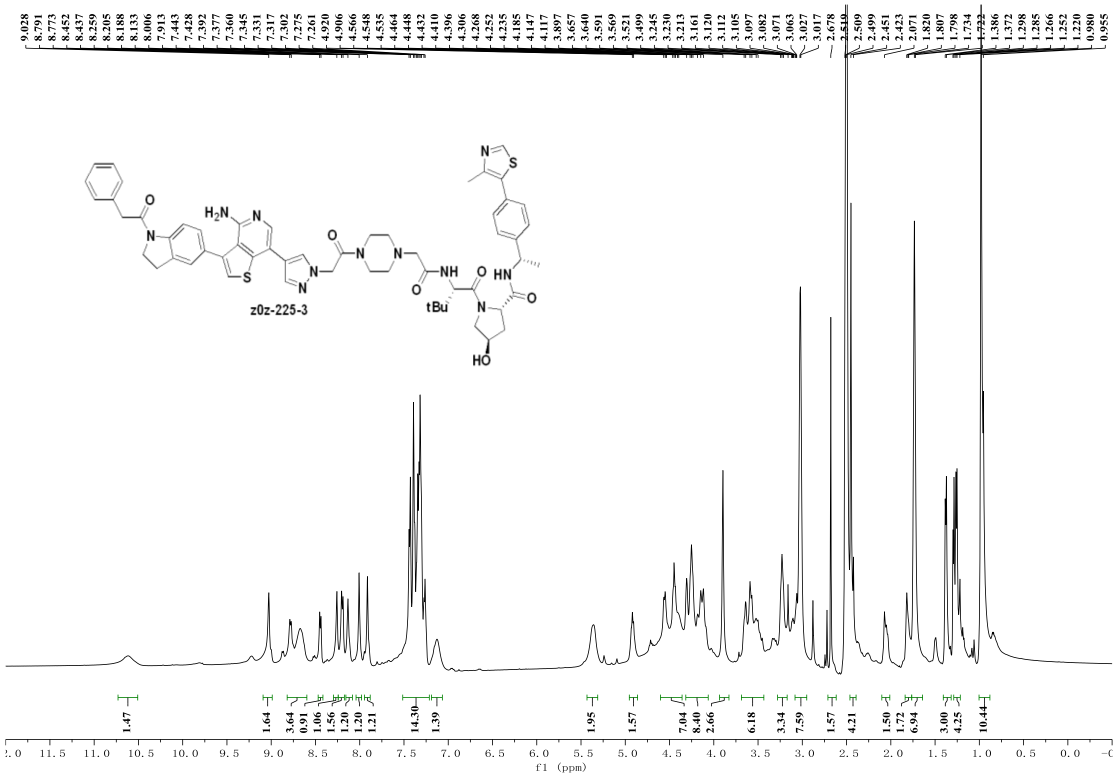
z0z-225-3
f1 (ppm)

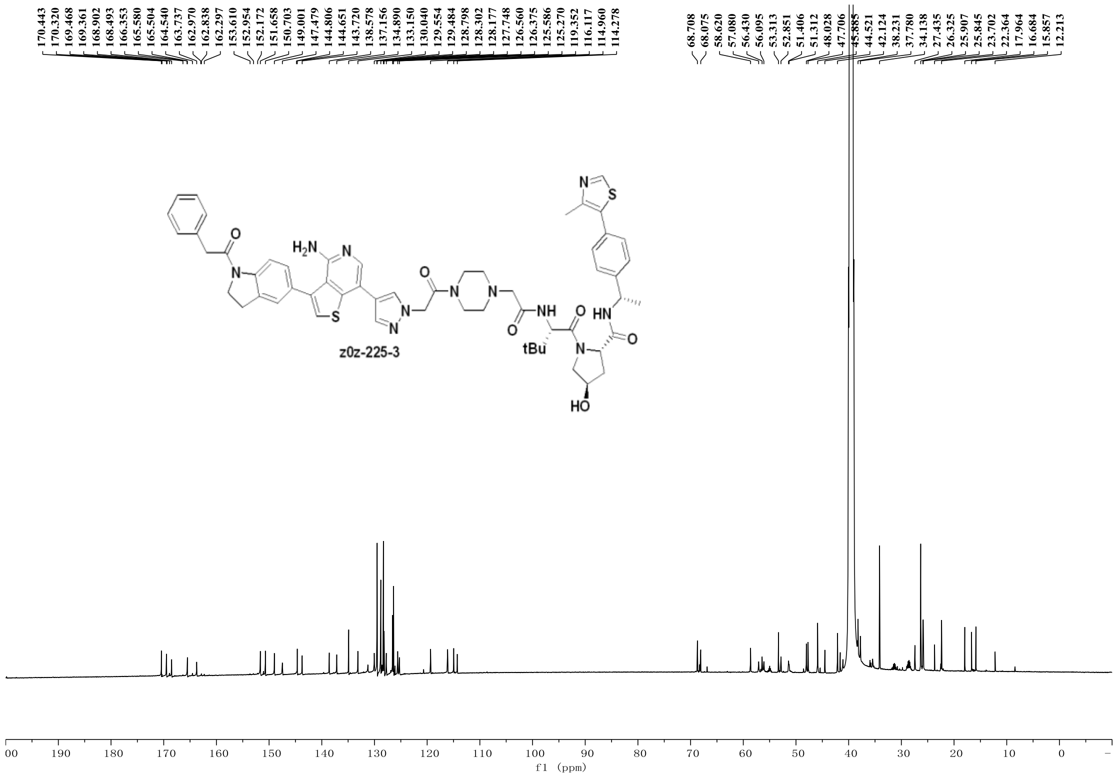
z0z-225-3
f1 (ppm)

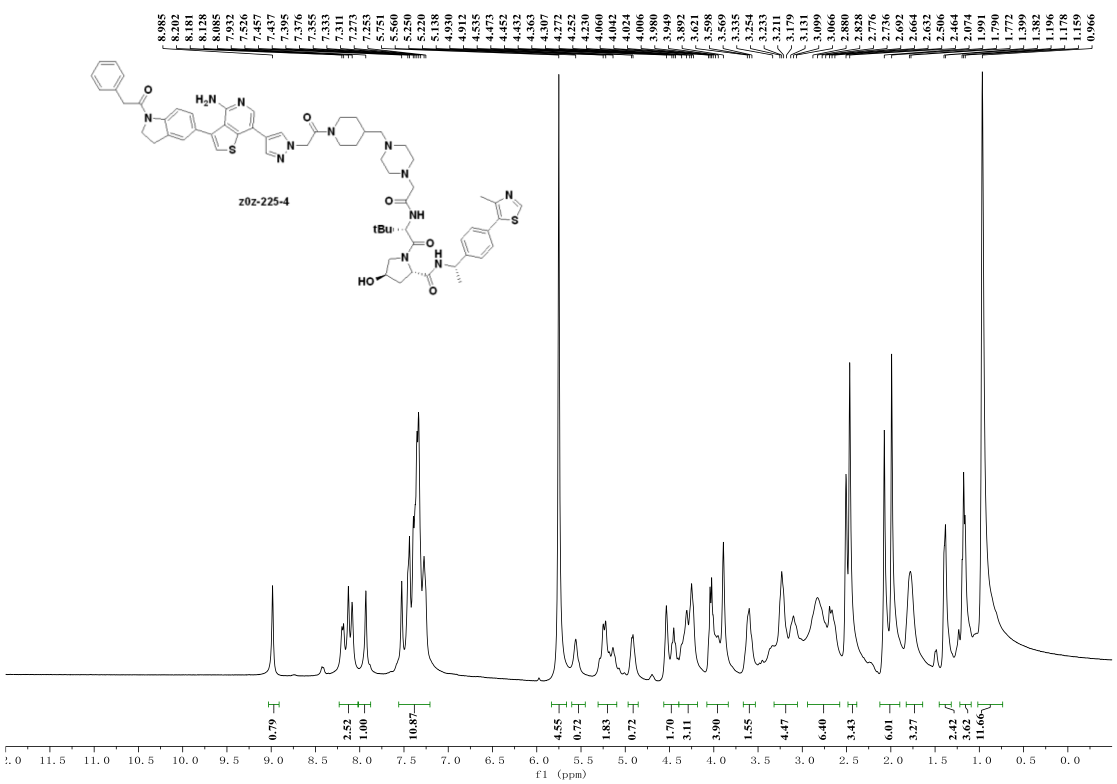
8.985 8.202 8.181 8.128 8.085 7.932 7.526 7.457 7.437 7.395 7.376 7.355 7.333 7.311 7.273 7.253 5.751 5.560 5.250 5.220 5.138 4.930 4.912 4.535 4.473 4.452 4.432 4.363 4.307 4.272 4.252 4.230 4.060 4.042 4.024 4.006 3.980 3.949 3.892 3.621 3.598 3.569 3.335 3.254 3.233 3.211 3.179 3.131 3.099 3.066 2.880 2.828 2.776 2.736 2.692 2.664 2.632 2.506 2.464 2.074 1.991 1.790 1.772 1.399 1.382 1.196 1.178 1.159 0.966
z0z-225-4
0.79 2.52 1.00 10.87 4.55 0.72 1.83 0.72 1.70 3.11 3.90 1.55 4.47 6.40 3.43 6.01 3.27 2.42 3.62 11.66
f1 (ppm)

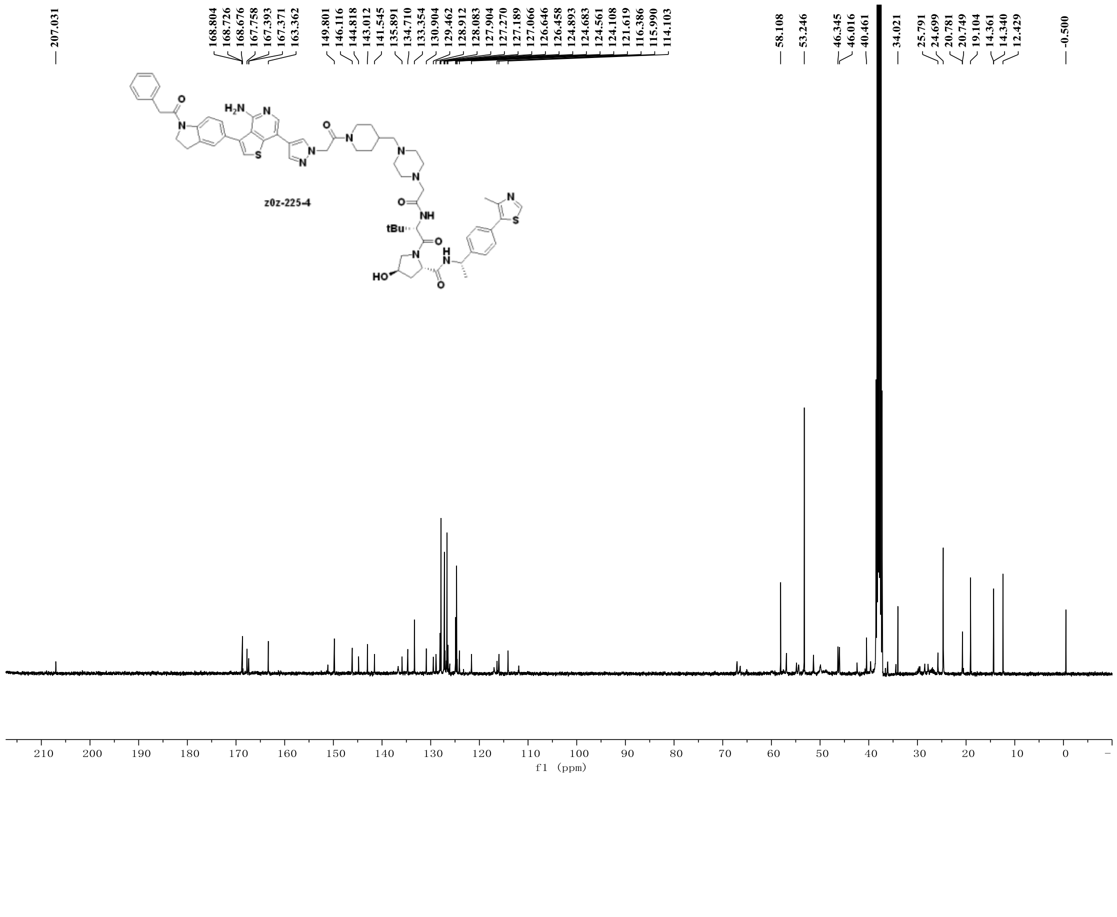
207.031
168.804 168.726 168.676 167.758 167.393 167.371 163.362
149.801 146.116 144.818 143.012 141.545 135.891 134.710 133.354 130.904 129.462 128.912 128.083 127.904 127.270 127.189 127.066 126.646 126.458 124.893 124.683 124.561 124.108 121.619 116.386 115.990 114.103
58.108 53.246 46.345 46.016 40.461 34.021 25.791 24.699 20.781 20.749 19.104 14.361 14.340 12.429 -0.500
z0z-225-4
f1 (ppm)

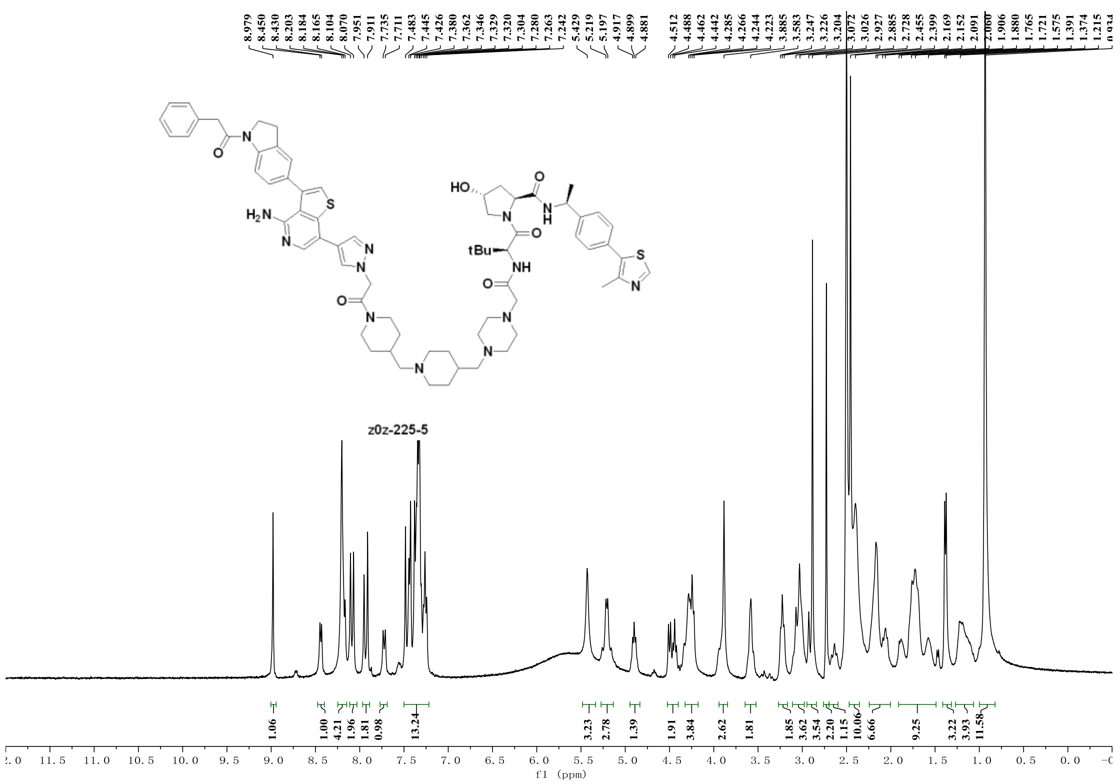
8.979
8.450
8.430
8.203
8.184
8.165
8.104
8.070
7.951
7.911
7.735
7.711
7.483
7.445
7.426
7.380
7.362
7.346
7.329
7.320
7.304
7.280
7.263
7.242
5.429
5.219
5.197
4.917
4.899
4.881
4.512
4.488
4.462
4.442
4.285
4.266
4.244
4.223
3.885
3.583
3.247
3.226
3.204
3.072
3.026
2.927
2.885
2.728
2.455
2.399
2.169
2.152
2.091
2.060
1.906
1.880
1.765
1.721
1.575
1.391
1.374
1.215
HO
H
N
O
tBu
NH
O
N
S
N
O
N
H$_2$N
N
S
N
N
O
N
N
N
N
z0z-225-5
1.06
1.00
4.21
1.96
1.81
0.98
13.24
3.23
2.78
1.39
1.91
3.84
2.62
1.81
1.85
3.62
3.54
2.20
1.15
10.06
6.66
9.25
3.22
3.93
11.58
f1 (ppm)

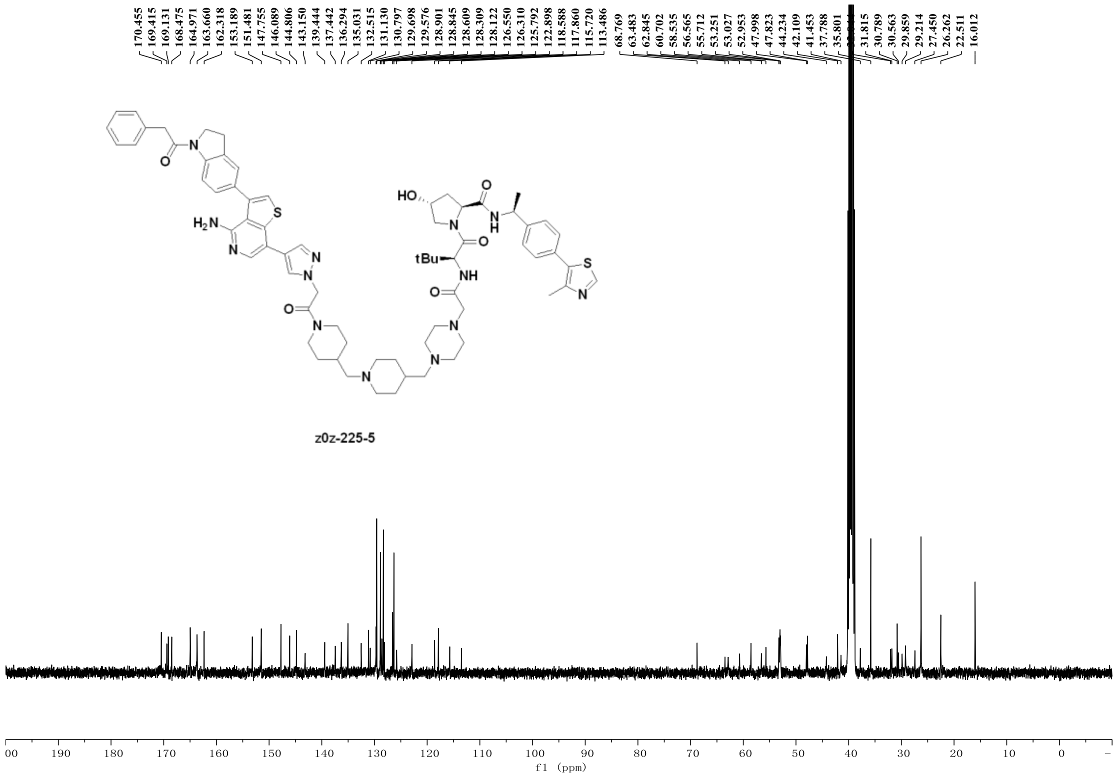
170.455
169.415
169.131
168.475
164.971
163.660
162.318
153.189
151.481
147.755
146.089
144.806
143.150
139.444
137.442
136.294
135.031
132.515
131.130
130.797
129.698
129.576
128.901
128.845
128.609
128.309
128.122
126.550
126.310
125.792
122.898
118.588
117.860
115.720
113.486
68.769
63.483
62.845
60.702
58.535
56.565
55.712
53.251
53.027
52.953
47.998
47.823
44.234
42.109
41.453
37.788
35.801
31.815
30.789
30.563
29.859
29.214
27.450
26.262
22.511
16.012
z0z-225-5
f1 (ppm)

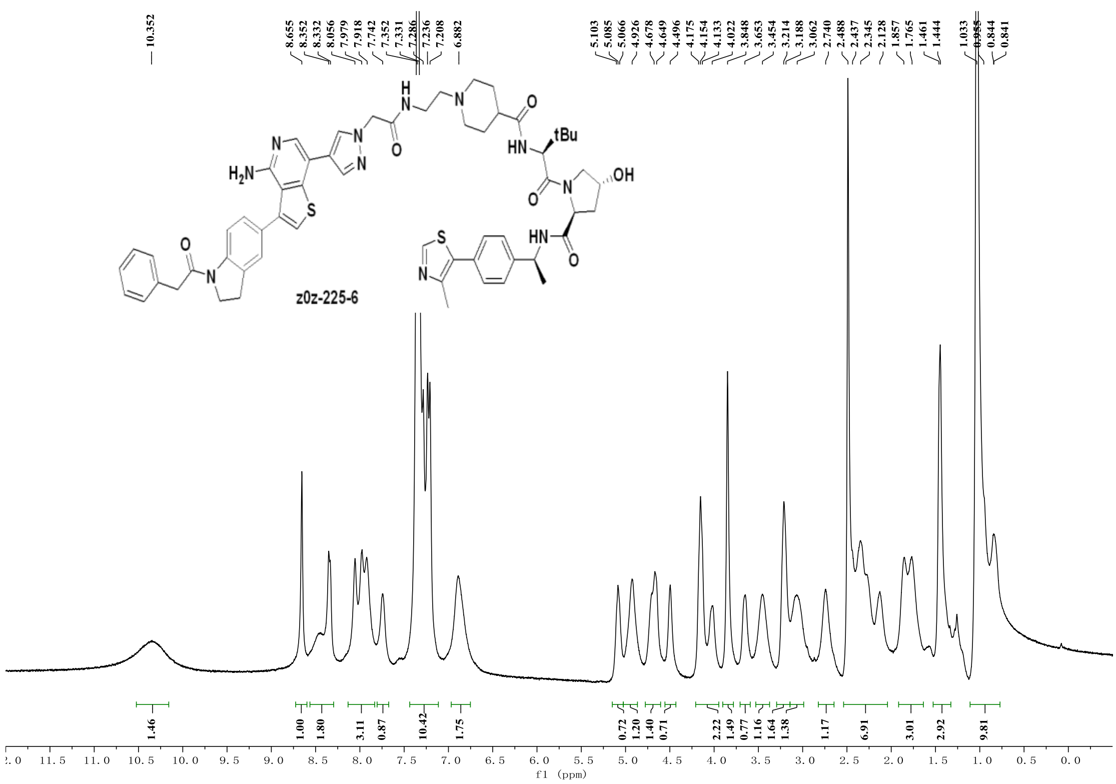
z0z-225-6
f1 (ppm)

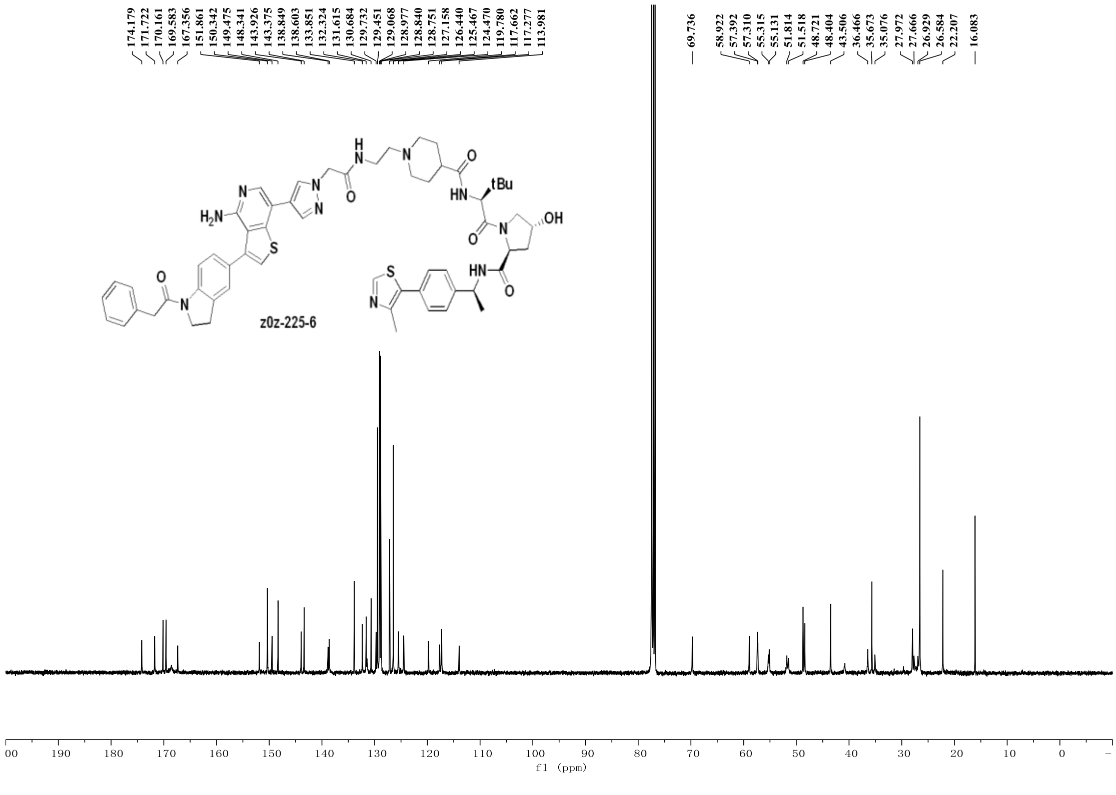
z0z-225-6
f1 (ppm)

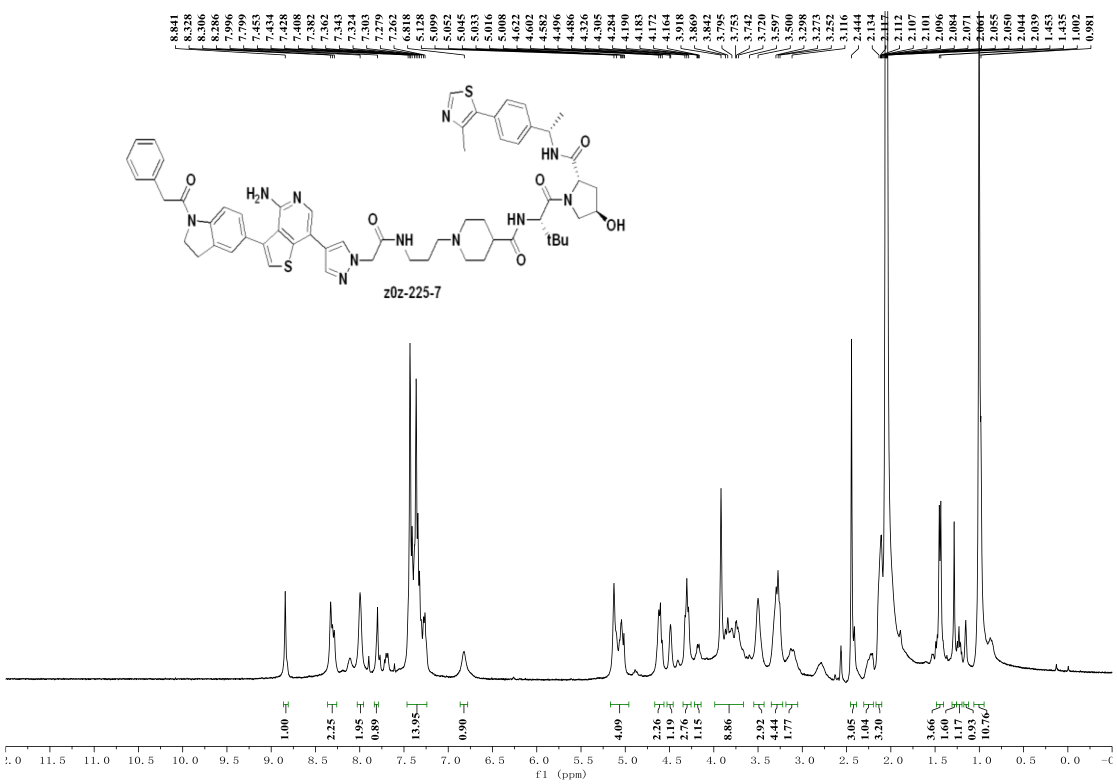

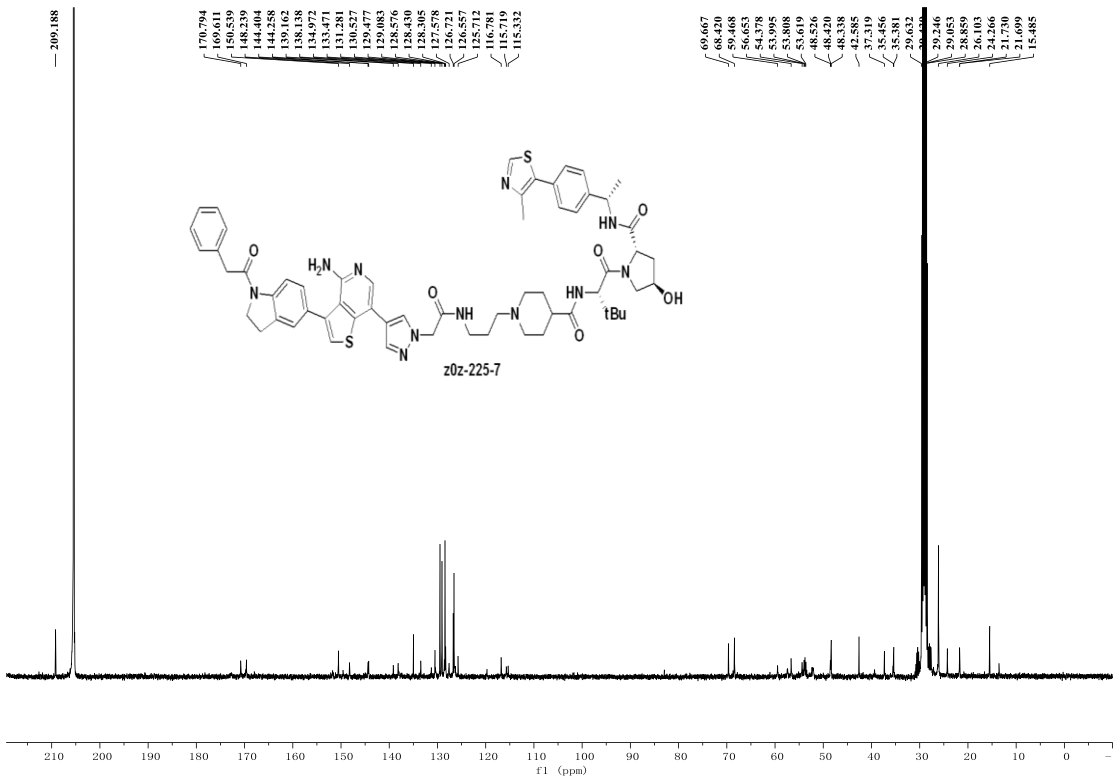

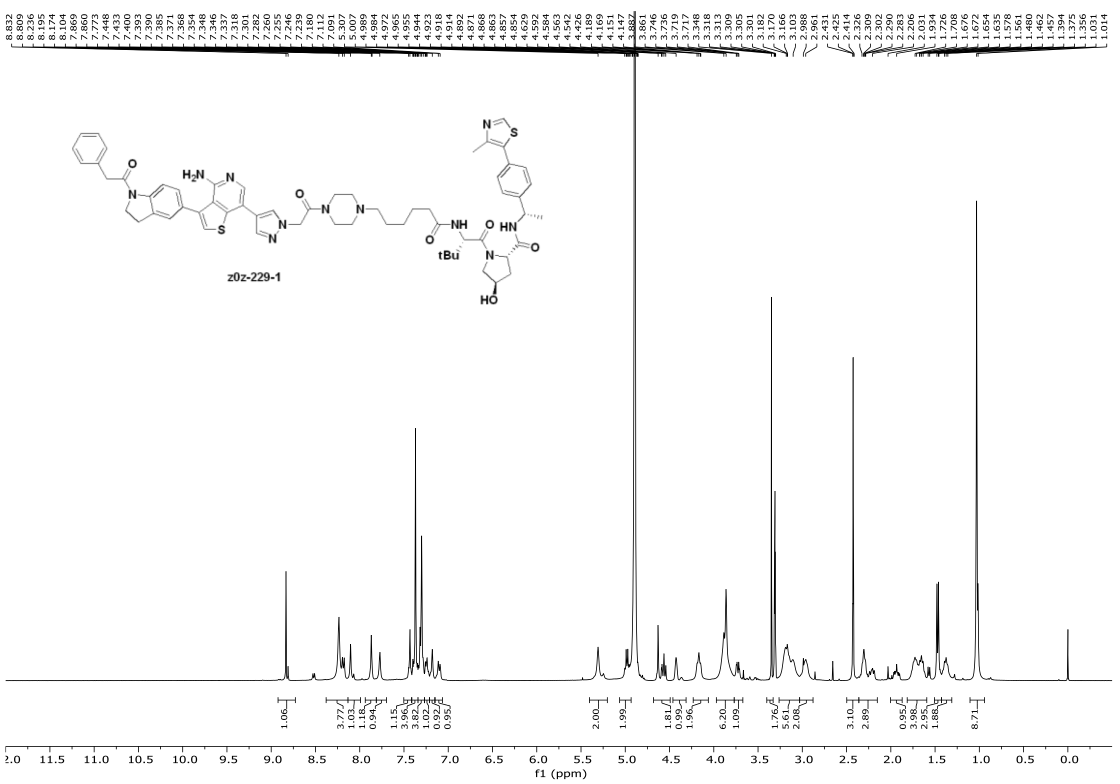

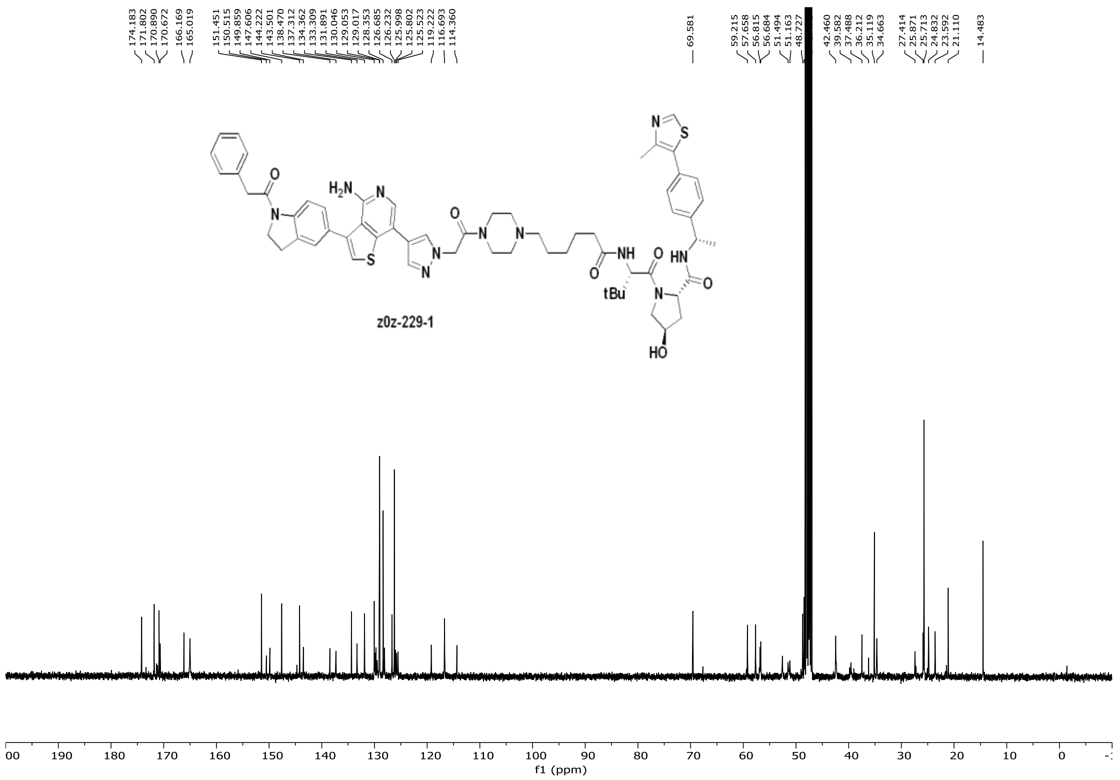

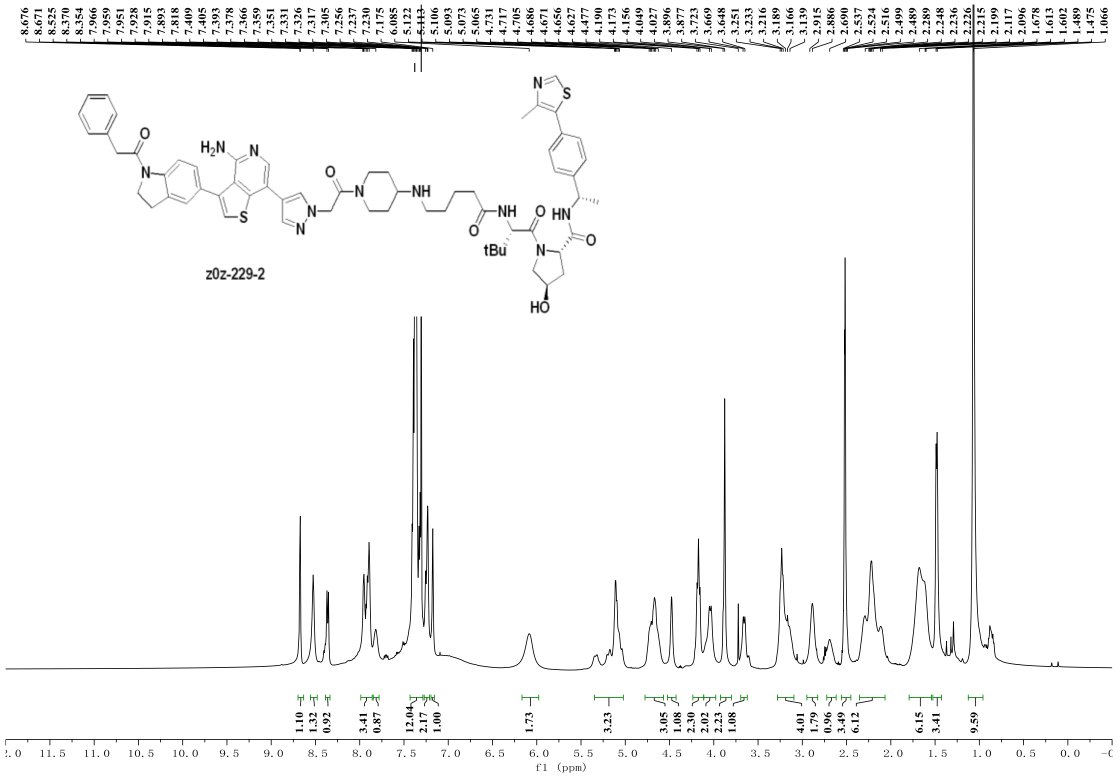

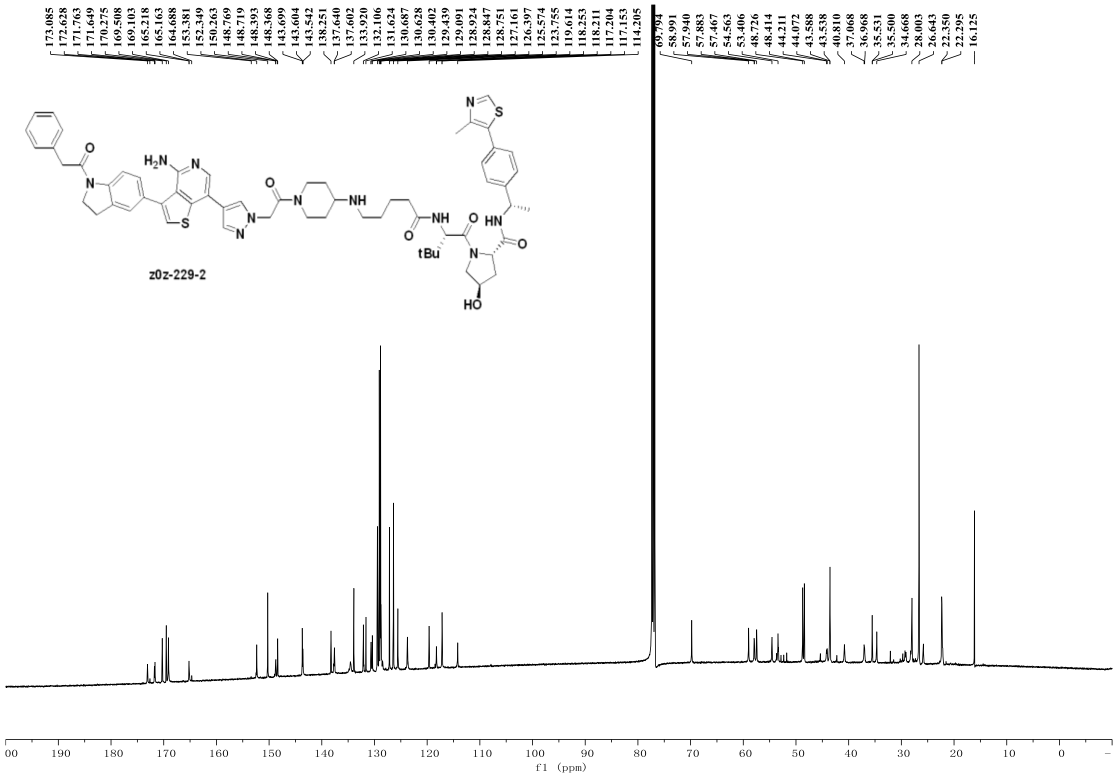

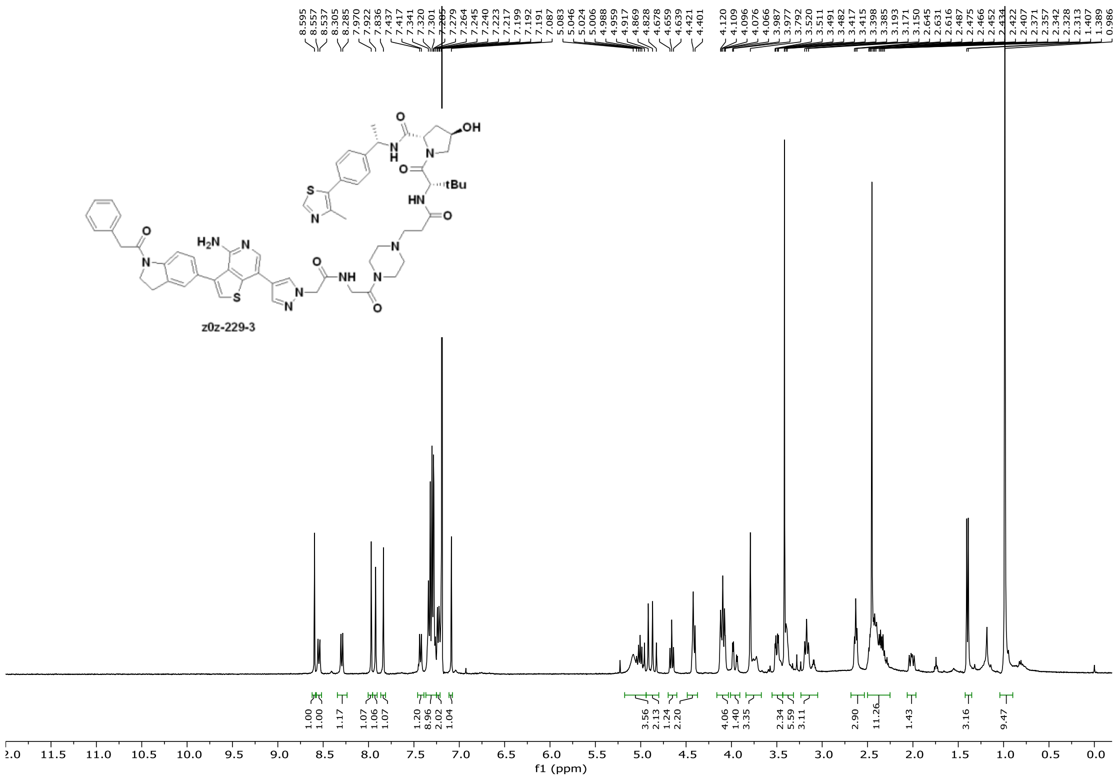
z0z-229-3
f1 (ppm)

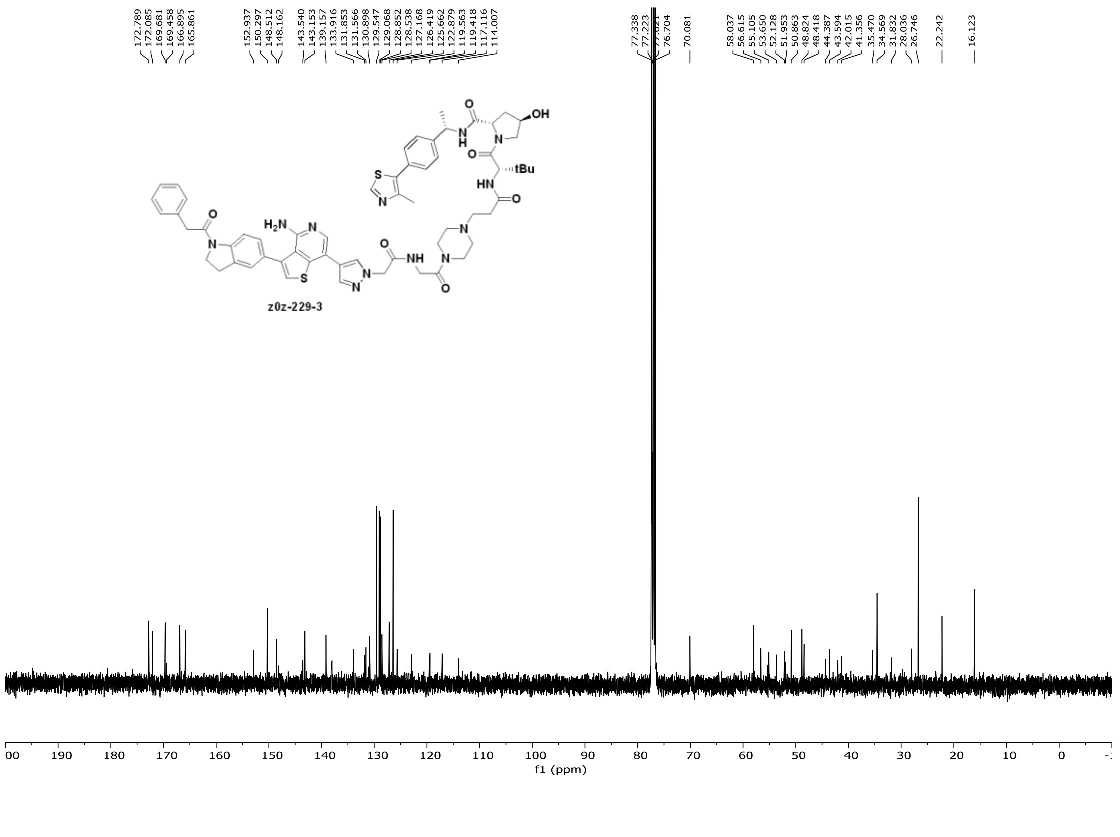
z0z-229-3
f1 (ppm)

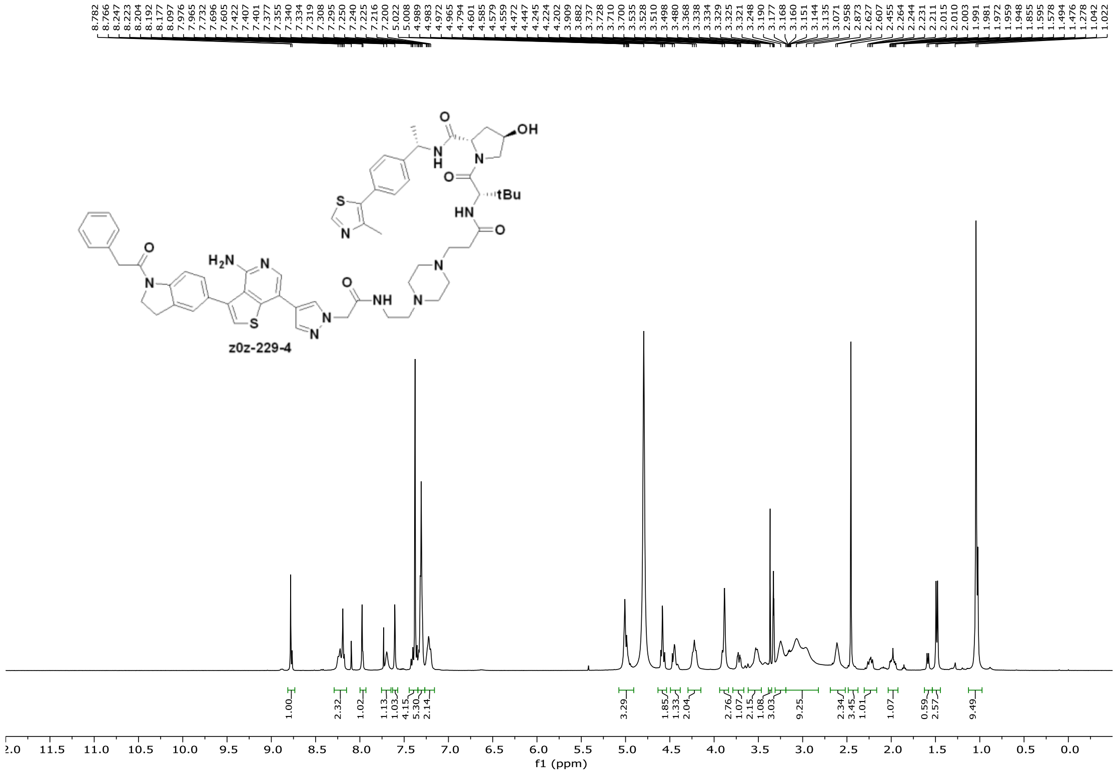
z0z-229-4
f1 (ppm)

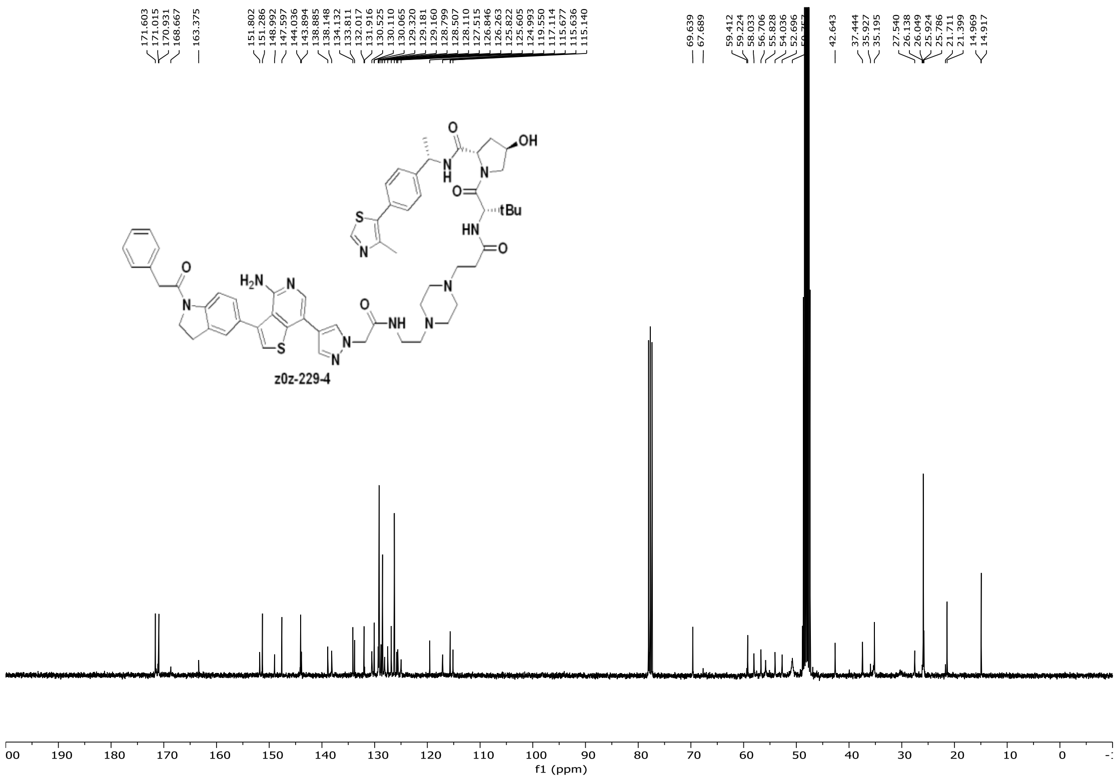
z0z-229-4
f1 (ppm)

HPLC Data

204-2

<Sample Information>

Sample Name	: 204-2-small		
Sample ID	:		
Data Filename	: 204-2-SMALL.lcd		
Method Filename	: analytic_25min_0.8ml_min_30min_total_4.5_slope.lcm		
Batch Filename	: normal batch.lcb		
Vial #	: 1-46	Sample Type	: Unknown
Injection Volume	: 5 uL		
Date Acquired	: 8/19/2024 11:38:26 AM	Acquired by	: System Administrator
Date Processed	: 8/19/2024 12:03:27 PM	Processed by	: System Administrator

<Chromatogram>

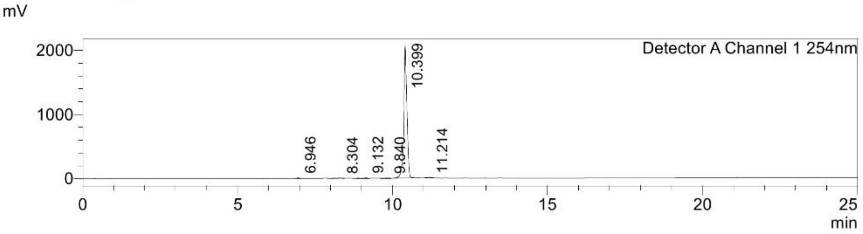

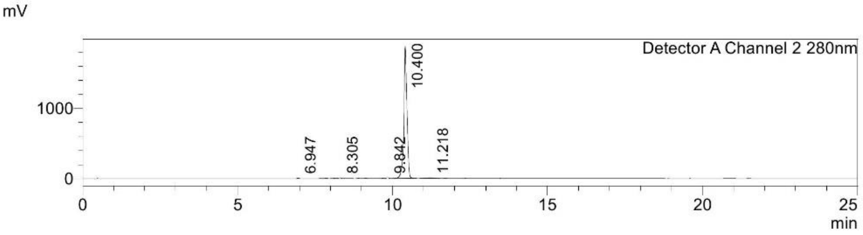

<Peak Table>

Detector A Channel 1 254nm

Peak#	Ret. Time	Height	Width at 5% Height	Area	Area%
1	6.946	10127	0.106	33135	0.230
2	8.304	5617	0.308	51897	0.360
3	9.132	8662	0.200	58501	0.405
4	9.840	5536	0.293	41737	0.289
5	10.399	2043217	0.275	14134891	97.940
6	11.214	7528	0.386	112054	0.776
Total		2080688		14432216	100.000

Detector A Channel 2 280nm

Peak#	Ret. Time	Height	Width at 5% Height	Area	Area%
1	6.947	7209	0.111	24362	0.184
2	8.305	8725	0.292	77446	0.584
3	9.842	10213	0.297	73877	0.557
4	10.400	1863591	0.275	12972833	97.885
5	11.218	8573	0.368	104574	0.789
Total		1898311		13253092	100.000

204-4

<Sample Information>

Sample Name	: 204-4		
Sample ID	:		
Data Filename	: 204-4.lcd		
Method Filename	: analytic_25min_0.8ml_min_30min_total_4.5_slope.lcm		
Batch Filename	: normal batch.lcb		
Vial #	: 1-48	Sample Type	: Unknown
Injection Volume	: 5 uL		
Date Acquired	: 8/19/2024 1:26:48 PM	Acquired by	: System Administrator
Date Processed	: 8/19/2024 1:51:49 PM	Processed by	: System Administrator

<Chromatogram>

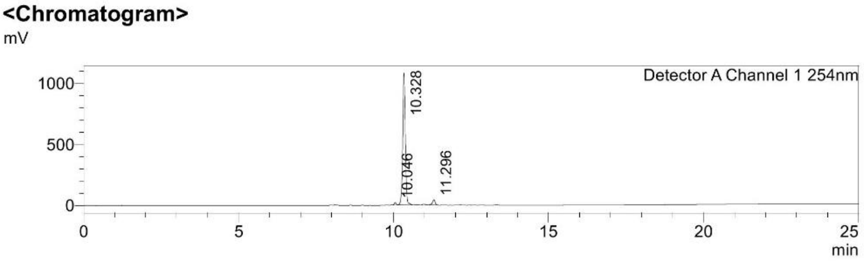

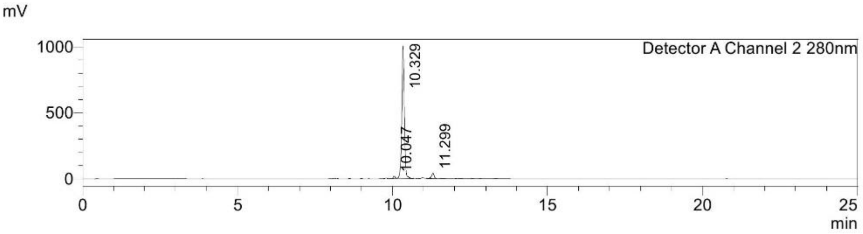

<Peak Table>

Detector A Channel 1 254nm

Peak#	Ret. Time	Height	Width at 5% Height	Area	Area%
1	10.046	18951	0.117	71058	1.086
2	10.328	1072584	0.206	6218702	95.035
3	11.296	42610	0.212	253841	3.879
Total		1134145		6543601	100.000

Detector A Channel 2 280nm

Peak#	Ret. Time	Height	Width at 5% Height	Area	Area%
1	10.047	16864	0.119	63966	1.063
2	10.329	993254	0.206	5725109	95.097
3	11.299	39010	0.216	231181	3.840
Total		1049127		6020255	100.000

204-6

<Sample Information>

Sample Name	: 204-6		
Sample ID	:		
Data Filename	: 204-6.lcd		
Method Filename	: analytic_25min_0.8ml_min_30min_total_4.5_slope.lcm		
Batch Filename	: normal batch.lcb		
Vial #	: 1-49	Sample Type	: Unknown
Injection Volume	: 5 uL		
Date Acquired	: 8/19/2024 2:17:22 PM	Acquired by	: System Administrator
Date Processed	: 8/20/2024 9:48:24 AM	Processed by	: System Administrator

<Chromatogram>

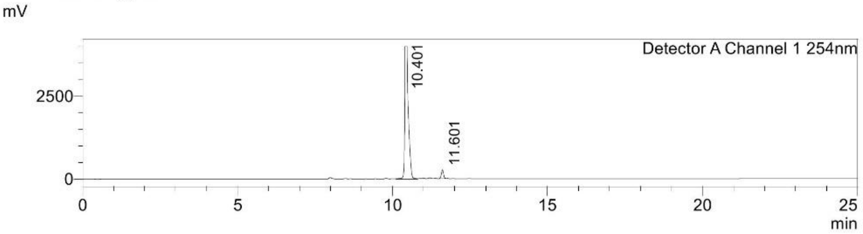

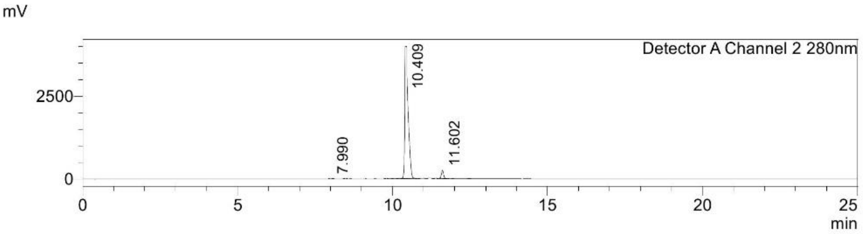

<Peak Table>

Detector A Channel 1 254nm

Peak#	Ret. Time	Height	Width at 5% Height	Area	Area%
1	10.401	3988888	0.257	32708251	96.216
2	11.601	257800	0.163	1286233	3.784
Total		4246687		33994484	100.000

Detector A Channel 2 280nm

Peak#	Ret. Time	Height	Width at 5% Height	Area	Area%
1	7.990	20860	0.230	164835	0.522
2	10.409	3985963	0.253	30222199	95.649
3	11.602	247282	0.162	1210079	3.830
Total		4254105		31597113	100.000

208-2

<Sample Information>

Sample Name	: 208-2		
Sample ID	:		
Data Filename	: 208-2.lcd		
Method Filename	: analytic_25min_0.8ml_min_30min_total_4.5_slope.lcm		
Batch Filename	: normal batch.lcb		
Vial #	: 1-50	Sample Type	: Unknown
Injection Volume	: 5 uL		
Date Acquired	: 8/19/2024 2:47:34 PM	Acquired by	: System Administrator
Date Processed	: 8/19/2024 3:12:35 PM	Processed by	: System Administrator

<Chromatogram>

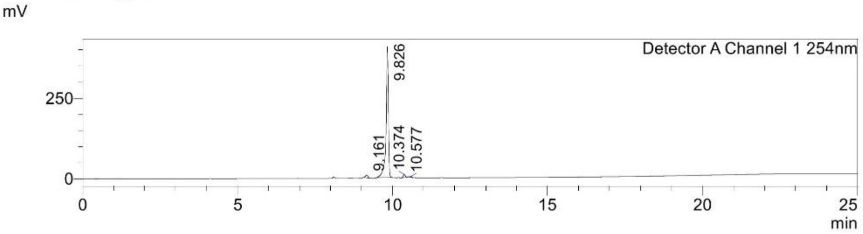

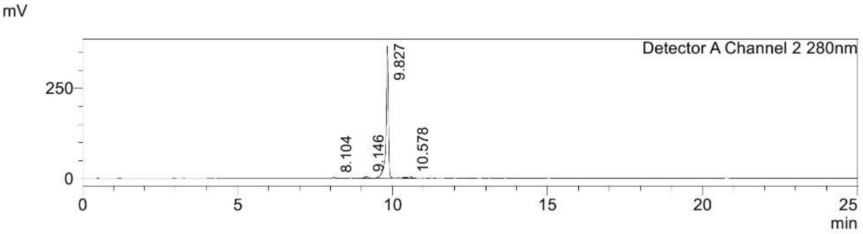

<Peak Table>

Detector A Channel 1 254nm

Peak#	Ret. Time	Height	Width at 5% Height	Area	Area%
1	9.161	7889	0.161	44174	2.009
2	9.826	399571	0.236	2103818	95.698
3	10.374	8742	0.144	40409	1.838
4	10.577	2277	0.146	9999	0.455
Total		418478		2198400	100.000

Detector A Channel 2 280nm

Peak#	Ret. Time	Height	Width at 5% Height	Area	Area%
1	8.104	3410	0.119	12803	0.633
2	9.146	5229	0.232	37125	1.835
3	9.827	361136	0.241	1927643	95.259
4	10.578	3834	0.432	46010	2.274
Total		373609		2023581	100.000

208-4

<Sample Information>

Sample Name	: 208-4		
Sample ID	:		
Data Filename	: 208-4.lcd		
Method Filename	: analytic_25min_0.8ml_min_30min_total_4.5_slope.lcm		
Batch Filename	: normal batch.lcb		
Vial #	: 1-51	Sample Type	: Unknown
Injection Volume	: 5 uL		
Date Acquired	: 8/19/2024 3:47:58 PM	Acquired by	: System Administrator
Date Processed	: 8/19/2024 4:12:59 PM	Processed by	: System Administrator

<Chromatogram>

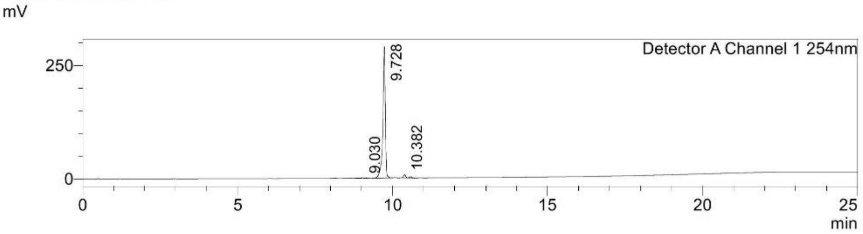

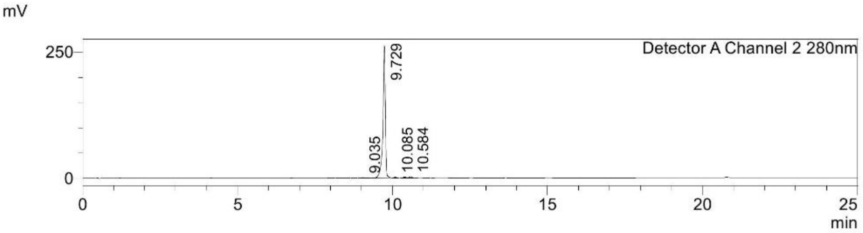

<Peak Table>

Detector A Channel 1 254nm

Peak#	Ret. Time	Height	Width at 5% Height	Area	Area%
1	9.030	966	0.418	10644	0.658
2	9.728	287802	0.228	1563751	96.635
3	10.382	6791	0.150	43813	2.707
Total		295558		1618208	100.000

Detector A Channel 2 280nm

Peak#	Ret. Time	Height	Width at 5% Height	Area	Area%
1	9.035	871	0.269	7103	0.495
2	9.729	259024	0.227	1402843	97.676
3	10.085	1131	0.152	5327	0.371
4	10.584	2196	0.356	20951	1.459
Total		263222		1436223	100.000

208-6

<Sample Information>

Sample Name	: 208-6		
Sample ID	:		
Data Filename	: 208-6.lcd		
Method Filename	: analytic_25min_0.8ml_min_30min_total_4.5_slope.lcm		
Batch Filename	: normal batch.lcb		
Vial #	: 1-52	Sample Type	: Unknown
Injection Volume	: 5 uL		
Date Acquired	: 8/19/2024 4:18:10 PM	Acquired by	: System Administrator
Date Processed	: 8/20/2024 9:47:27 AM	Processed by	: System Administrator

<Chromatogram>

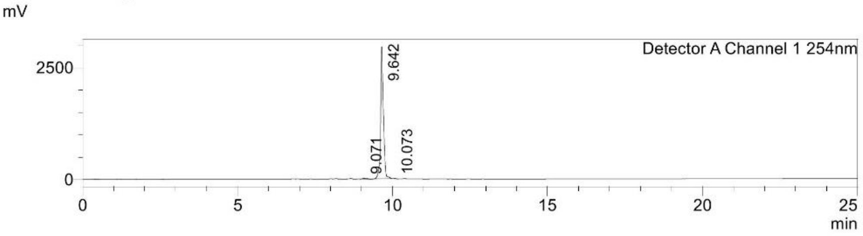

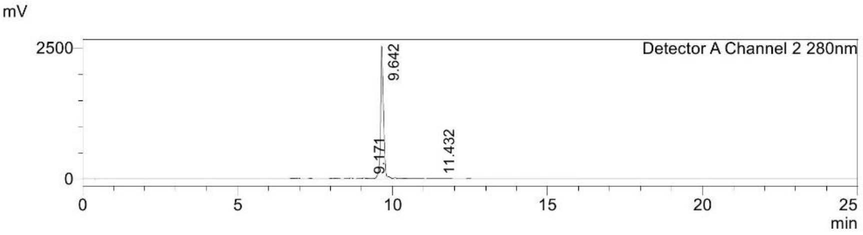

<Peak Table>

Detector A Channel 1 254nm

Peak#	Ret. Time	Height	Width at 5% Height	Area	Area%
1	9.071	18569	0.237	135480	0.731
2	9.642	2933841	0.249	18369110	99.056
3	10.073	9472	0.122	39627	0.214
Total		2961882		18544217	100.000

Detector A Channel 2 280nm

Peak#	Ret. Time	Height	Width at 5% Height	Area	Area%
1	9.171	9514	--	50787	0.293
2	9.642	2495853	0.256	17284391	99.679
3	11.432	1036	--	4891	0.028
Total		2506402		17340069	100.000

214-6

<Sample Information>

Sample Name	: 214-6-repeat		
Sample ID	:		
Data Filename	: 214-6-repeat.lcd		
Method Filename	: analytic_25min_0.8ml_min_30min_total_4.5_slope.lcm		
Batch Filename	: normal batch.lcb		
Vial #	: 1-46	Sample Type	: Unknown
Injection Volume	: 2 uL		
Date Acquired	: 8/30/2024 12:11:12 PM	Acquired by	: System Administrator
Date Processed	: 12/26/2024 2:59:00 PM	Processed by	: System Administrator

<Chromatogram>

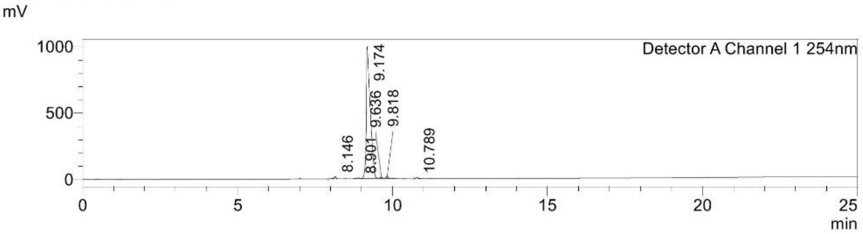

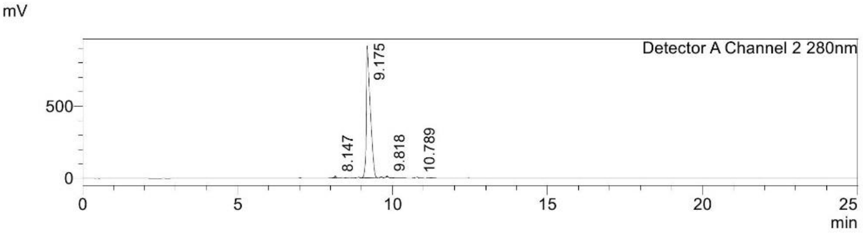

<Peak Table>

Detector A Channel 1 254nm

Peak#	Ret. Time	Height	Width at 5% Height	Area	Area%
1	8.146	16739	0.255	84463	0.926
2	8.901	6319	0.226	38153	0.418
3	9.174	988831	0.329	8844610	96.964
4	9.636	8106	0.130	32305	0.354
5	9.818	15984	0.305	79235	0.869
6	10.789	6530	0.175	42816	0.469
Total		1042510		9121583	100.000

Detector A Channel 2 280nm

Peak#	Ret. Time	Height	Width at 5% Height	Area	Area%
1	8.147	13585	0.133	52076	0.632
2	9.175	905932	0.329	8118751	98.596
3	9.818	9252	0.073	29699	0.361
4	10.789	5418	0.156	33852	0.411
Total		934187		8234378	100.000

214-7

<Sample Information>

Sample Name	: 214-7
Sample ID	:
Data Filename	: 214-7.lcd
Method Filename	: analytic_25min_0.8ml_min_30min_total_4.5_slope.lcm
Batch Filename	: normal batch.lcb
Vial #	: 1-47
Sample Type	: Unknown
Injection Volume	: 2 uL
Date Acquired	: 8/30/2024 12:41:24 PM
Acquired by	: System Administrator
Date Processed	: 8/30/2024 2:08:23 PM
Processed by	: System Administrator

<Chromatogram>

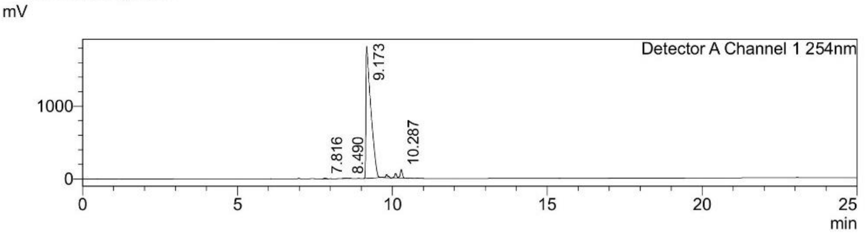

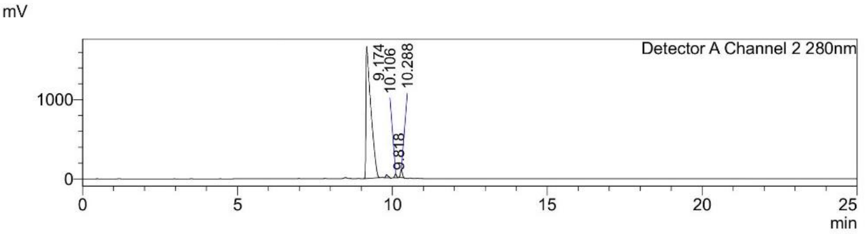

<Peak Table>

Detector A Channel 1 254nm

Peak#	Ret. Time	Height	Width at 5% Height	Area	Area%
1	7.816	6665	0.144	33994	0.162
2	8.490	7245	0.205	47928	0.228
3	9.173	1807669	0.379	19896691	94.768
4	10.287	114875	0.171	1016476	4.841
Total		1936454		20995089	100.000

Detector A Channel 2 280nm

Peak#	Ret. Time	Height	Width at 5% Height	Area	Area%
1	9.174	1663334	0.379	18350665	95.615
2	9.818	39700	0.163	205552	1.071
3	10.106	46561	0.124	192845	1.005
4	10.288	103225	0.142	443209	2.309
Total		1852820		19192270	100.000

214-8

<Sample Information>

Sample Name	: 214-8-doublecheck		
Sample ID	:		
Data Filename	: 214-8-doublecheck.lcd		
Method Filename	: analytic_15min_0.8ml_min_20min_total_9.0_slope.lcm		
Batch Filename	: normal batch.lcb		
Vial #	: 2-36	Sample Type	: Unknown
Injection Volume	: 5 uL		
Date Acquired	: 3/3/2025 3:06:34 PM	Acquired by	: System Administrator
Date Processed	: 3/4/2025 4:36:45 PM	Processed by	: System Administrator

<Chromatogram>

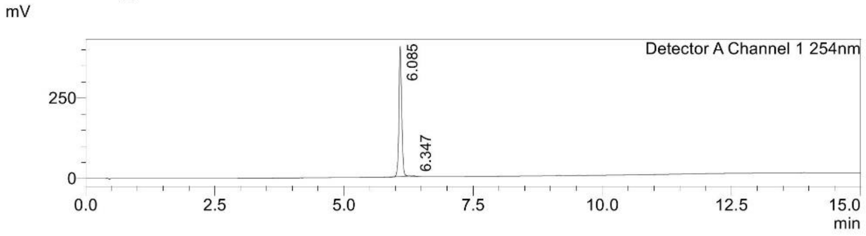

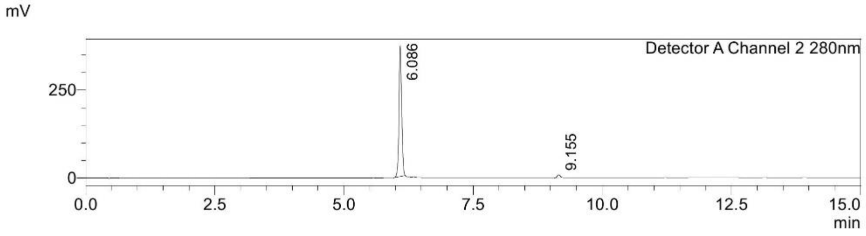

<Peak Table>

Detector A Channel 1 254nm

Peak#	Ret. Time	Height	Width at 5% Height	Area	Area%
1	6.085	394259	0.134	1487411	99.765
2	6.347	1452	0.087	3509	0.235
Total		395711		1490919	100.000

Detector A Channel 2 280nm

Peak#	Ret. Time	Height	Width at 5% Height	Area	Area%
1	6.086	364530	0.129	1338083	97.756
2	9.155	8146	0.119	30717	2.244
Total		372676		1368799	100.000

214-9

<Sample Information>

Sample Name	: 214-9-doublecheck
Sample ID	:
Data Filename	: 214-9-doublecheck.lcd
Method Filename	: analytic_15min_0.8ml_min_20min_total_9.0_slope.lcm
Batch Filename	: normal batch.lcb
Vial #	: 2-40
Sample Type	: Unknown
Injection Volume	: 10 uL
Date Acquired	: 3/4/2025 4:07:43 PM
Acquired by	: System Administrator
Date Processed	: 3/4/2025 4:38:48 PM
Processed by	: System Administrator

<Chromatogram>

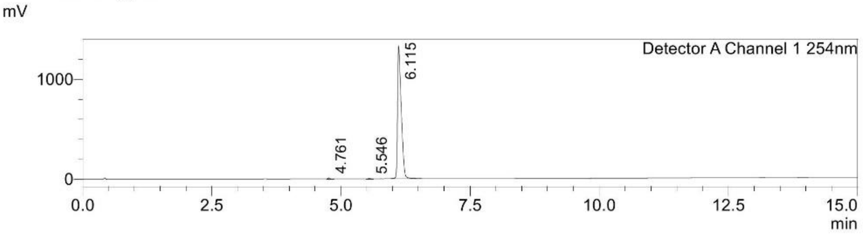

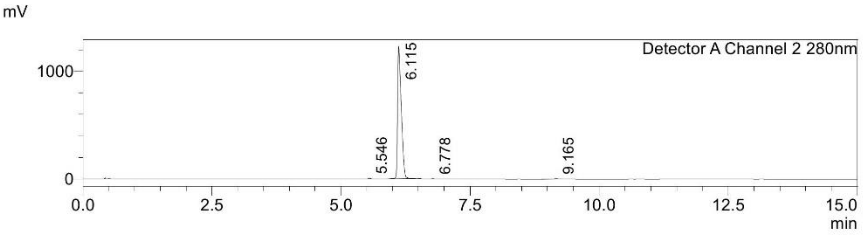

<Peak Table>

Detector A Channel 1 254nm

Peak#	Ret. Time	Height	Width at 5% Height	Area	Area%
1	4.761	8654	0.078	20586	0.314
2	5.546	3703	0.104	10000	0.153
3	6.115	1313476	0.164	6515429	99.533
Total		1325833		6546015	100.000

Detector A Channel 2 280nm

Peak#	Ret. Time	Height	Width at 5% Height	Area	Area%
1	5.546	7269	0.086	17677	0.291
2	6.115	1217551	0.164	6035844	99.242
3	6.778	2509	0.101	7892	0.130
4	9.165	3426	0.131	20527	0.338
Total		1230756		6081940	100.000

214-10

<Sample Information>

Sample Name	: 214-10-doublecheck		
Sample ID	:		
Data Filename	: 214-10-doublecheck.lcd		
Method Filename	: analytic_15min_0.8ml_min_20min_total_9.0_slope.lcm		
Batch Filename	: normal batch.lcb		
Vial #	: 2-41	Sample Type	: Unknown
Injection Volume	: 10 uL		
Date Acquired	: 3/4/2025 4:27:56 PM	Acquired by	: System Administrator
Date Processed	: 3/4/2025 4:42:57 PM	Processed by	: System Administrator

<Chromatogram>

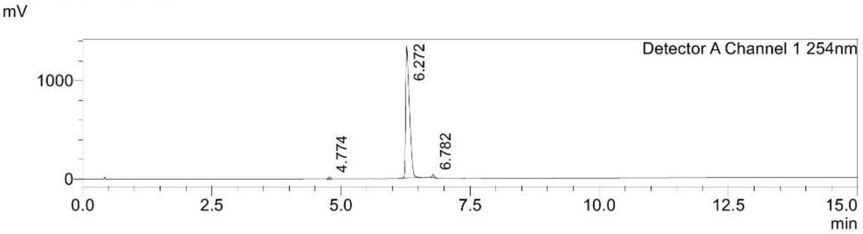

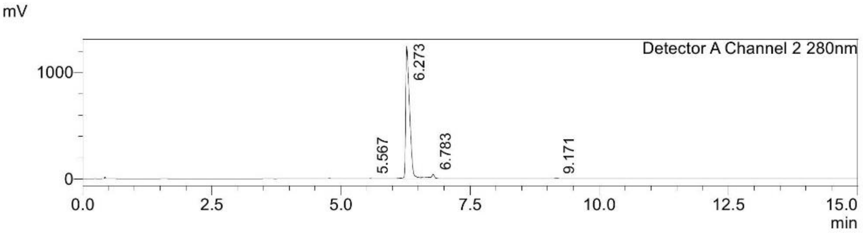

<Peak Table>

Detector A Channel 1 254nm

Peak#	Ret. Time	Height	Width at 5% Height	Area	Area%
1	4.774	19786	0.074	45683	0.664
2	6.272	1324045	0.167	6753403	98.126
3	6.782	32012	0.082	83275	1.210
Total		1375843		6882361	100.000

Detector A Channel 2 280nm

Peak#	Ret. Time	Height	Width at 5% Height	Area	Area%
1	5.567	1162	--	2719	0.041
2	6.273	1236364	0.170	6439576	96.163
3	6.783	39784	--	242784	3.626
4	9.171	2827	0.137	11411	0.170
Total		1280137		6696490	100.000

216-9

<Sample Information>

Sample Name	: 216-9-repeat
Sample ID	:
Data Filename	: 216-9-repeat.lcd
Method Filename	: analytic_25min_0.8ml_min_30min_total_4.5_slope.lcm
Batch Filename	: normal batch.lcb
Vial #	: 1-37
Injection Volume	: 5 uL
Date Acquired	: 8/20/2024 10:35:08 AM
Date Processed	: 8/20/2024 11:13:23 AM

Sample Type	: Unknown
Acquired by	: System Administrator
Processed by	: System Administrator

<Chromatogram>

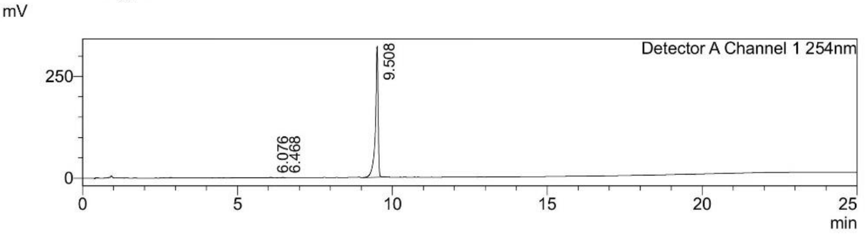

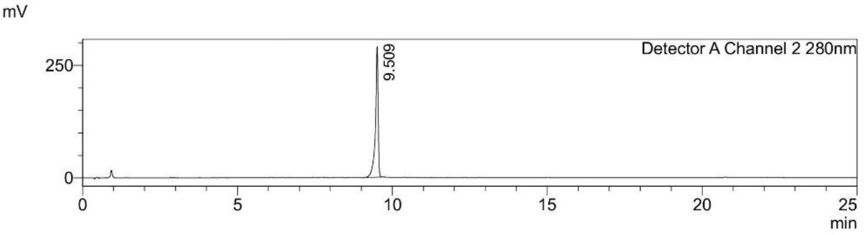

<Peak Table>

Detector A Channel 1 254nm

Peak#	Ret. Time	Height	Width at 5% Height	Area	Area%
1	6.076	940	0.180	4800	0.247
2	6.468	532	0.125	2126	0.109
3	9.508	318725	0.266	1935024	99.643
Total		320197		1941950	100.000

Detector A Channel 2 280nm

Peak#	Ret. Time	Height	Width at 5% Height	Area	Area%
1	9.509	286325	0.264	1727397	100.000
Total		286325		1727397	100.000

216-10

<Sample Information>

Sample Name : 216-10
Sample ID :
Data Filename : 216-10.lcd
Method Filename : analytic_25min_0.8ml_min_30min_total_4.5_slope.lcm
Batch Filename : normal batch.lcb
Vial # : 1-38 Sample Type : Unknown
Injection Volume : 5 uL
Date Acquired : 8/20/2024 11:05:22 AM Acquired by : System Administrator
Date Processed : 8/20/2024 12:28:22 PM Processed by : System Administrator

<Chromatogram>

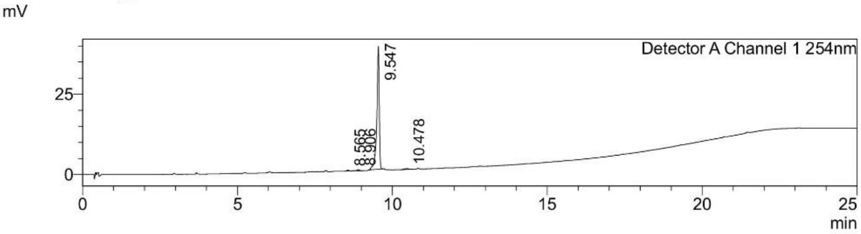

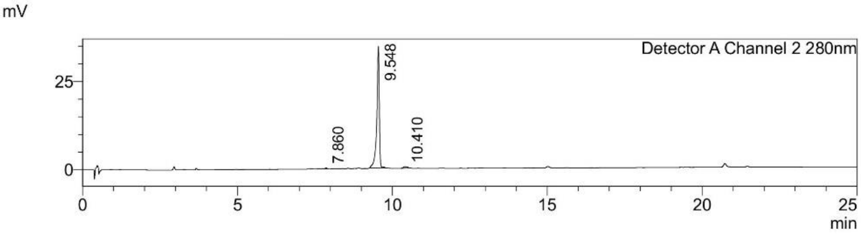

<Peak Table>

Detector A Channel 1 254nm

Peak#	Ret. Time	Height	Width at 5% Height	Area	Area%
1	8.565	269	0.134	1003	0.469
2	8.906	324	0.239	1933	0.905
3	9.547	37920	0.225	208159	97.439
4	10.478	294	0.309	2536	1.187
Total		38807		213631	100.000

Detector A Channel 2 280nm

Peak#	Ret. Time	Height	Width at 5% Height	Area	Area%
1	7.860	266	0.106	894	0.457
2	9.548	34191	0.227	191010	97.686
3	10.410	371	0.250	3632	1.857
Total		34828		195536	100.000

216-11

<Sample Information>

Sample Name	: 216-11		
Sample ID	:		
Data Filename	: 216-11.lcd		
Method Filename	: analytic_25min_0.8ml_min_30min_total_4.5_slope.lcm		
Batch Filename	: normal batch.lcb		
Vial #	: 1-39	Sample Type	: Unknown
Injection Volume	: 5 uL		
Date Acquired	: 8/20/2024 11:55:56 AM	Acquired by	: System Administrator
Date Processed	: 8/20/2024 12:28:48 PM	Processed by	: System Administrator

<Chromatogram>

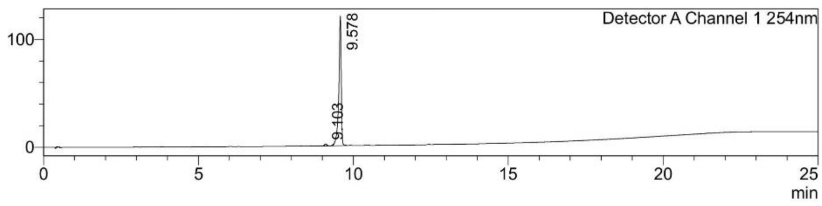

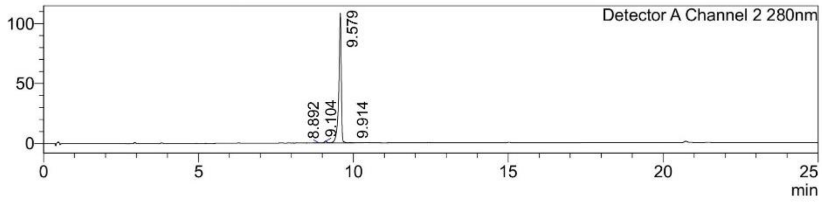

<Peak Table>

Detector A Channel 1 254nm

Peak#	Ret. Time	Height	Width at 5% Height	Area	Area%
1	9.103	1614	0.170	8622	1.293
2	9.578	119238	0.221	658410	98.707
Total		120852		667032	100.000

Detector A Channel 2 280nm

Peak#	Ret. Time	Height	Width at 5% Height	Area	Area%
1	8.892	100	--	1039	0.169
2	9.104	1590	--	10323	1.679
3	9.579	107727	0.222	600063	97.620
4	9.914	413	--	3268	0.532
Total		109831		614694	100.000

216-12

<Sample Information>

Sample Name	: 216-12
Sample ID	:
Data Filename	: 216-12.lcd
Method Filename	: analytic_25min_0.8ml_min_30min_total_4.5_slope.lcm
Batch Filename	: normal batch.lcb
Vial #	: 1-40
Sample Type	: Unknown
Injection Volume	: 5 uL
Date Acquired	: 8/20/2024 12:26:08 PM
Acquired by	: System Administrator
Date Processed	: 12/26/2024 2:50:27 PM
Processed by	: System Administrator

<Chromatogram>

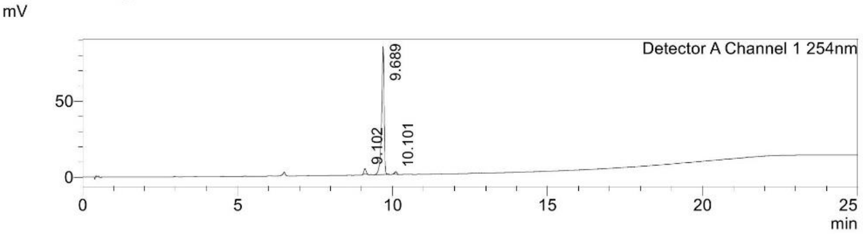

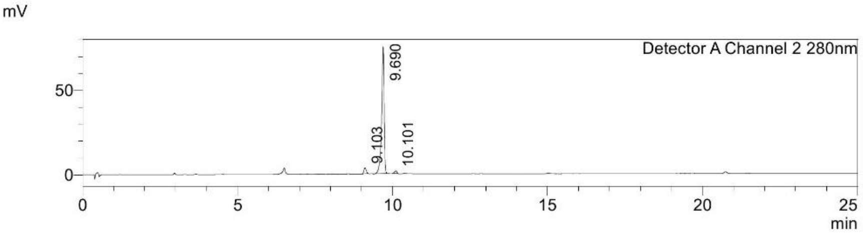

<Peak Table>

Detector A Channel 1 254nm

Peak#	Ret. Time	Height	Width at 5% Height	Area	Area%
1	9.102	3445	0.132	15981	3.235
2	9.689	83666	0.216	469927	95.128
3	10.101	1642	0.148	8084	1.637
Total		88752		493993	100.000

Detector A Channel 2 280nm

Peak#	Ret. Time	Height	Width at 5% Height	Area	Area%
1	9.103	3367	0.156	17278	3.908
2	9.690	74789	0.215	418247	94.600
3	10.101	1380	0.139	6597	1.492
Total		79536		442122	100.000

216-13

<Sample Information>

Sample Name : 216-13
Sample ID :
Data Filename : 216-13.lcd
Method Filename : analytic_25min_0.8ml_min_30min_total_4.5_slope.lcm
Batch Filename : normal batch.lcb
Vial # : 1-41 Sample Type : Unknown
Injection Volume : 5 uL
Date Acquired : 8/20/2024 1:26:38 PM Acquired by : System Administrator
Date Processed : 8/20/2024 1:51:39 PM Processed by : System Administrator

<Chromatogram>

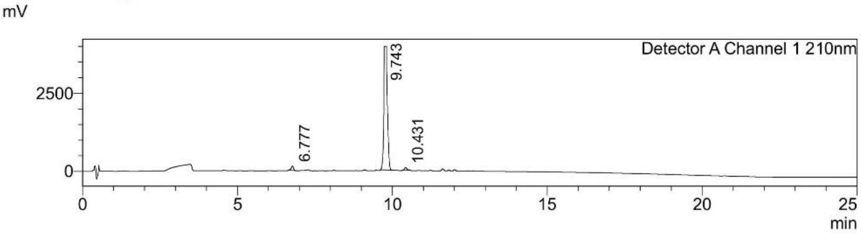

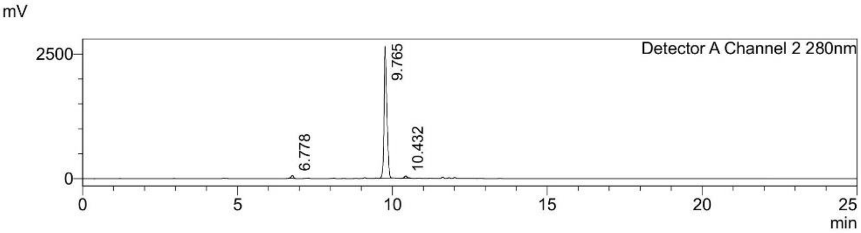

<Peak Table>

Detector A Channel 1 210nm

Peak#	Ret. Time	Height	Width at 5% Height	Area	Area%
1	6.777	145225	0.182	734922	2.250
2	9.743	3953179	0.238	31370143	96.062
3	10.431	93042	0.234	551055	1.687
Total		4191446		32656120	100.000

Detector A Channel 2 280nm

Peak#	Ret. Time	Height	Width at 5% Height	Area	Area%
1	6.778	61467	0.185	321801	1.875
2	9.765	2634651	0.226	16560729	96.488
3	10.432	43911	0.262	280934	1.637
Total		2740029		17163464	100.000

216-14

<Sample Information>

Sample Name	: 216-14-REPEAT
Sample ID	:
Data Filename	: 216-14-REPEAT.lcd
Method Filename	: analytic_25min_0.8ml_min_30min_total_4.5_slope.lcm
Batch Filename	: normal batch.lcb
Vial #	: 1-42
Sample Type	: Unknown
Injection Volume	: 5 uL
Date Acquired	: 8/20/2024 5:39:10 PM
Acquired by	: System Administrator
Date Processed	: 8/20/2024 6:24:25 PM
Processed by	: System Administrator

<Chromatogram>

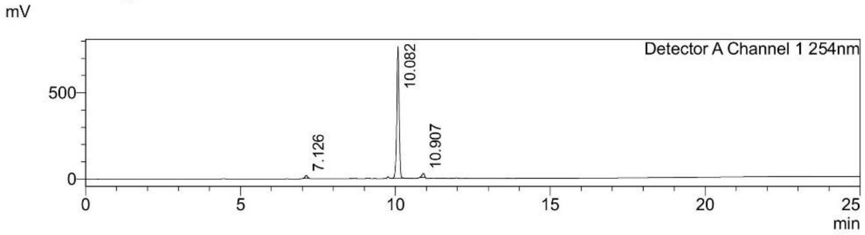

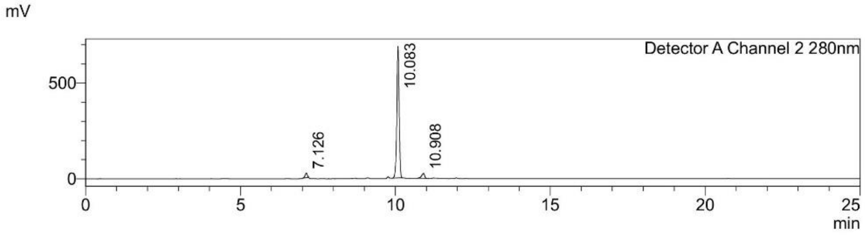

<Peak Table>

Detector A Channel 1 254nm

Peak#	Ret. Time	Height	Width at 5% Height	Area	Area%
1	7.126	15711	0.146	73758	1.784
2	10.082	758175	0.184	3939247	95.293
3	10.907	22599	0.154	120839	2.923
Total		796485		4133843	100.000

Detector A Channel 2 280nm

Peak#	Ret. Time	Height	Width at 5% Height	Area	Area%
1	7.126	23934	0.167	121638	3.172
2	10.083	681247	0.183	3537520	92.235
3	10.908	26023	0.236	176171	4.593
Total		731204		3835329	100.000

216-15

<Sample Information>

Sample Name : z0z-216-15-tube5
Sample ID :
Data Filename : z0z-216-15-tube5.lcd
Method Filename : analytic_15min_0.8ml_min_20min_total_9.0_slope.lcm
Batch Filename : normal batch.lcb
Vial # : 1-39 Sample Type : Unknown
Injection Volume : 5 uL
Date Acquired : 3/11/2025 1:33:41 PM Acquired by : System Administrator
Date Processed : 3/11/2025 2:07:40 PM Processed by : System Administrator

<Chromatogram>

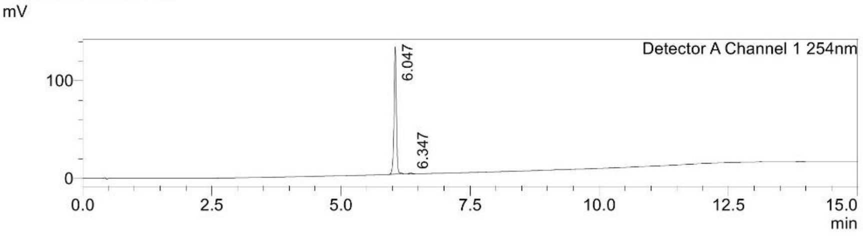

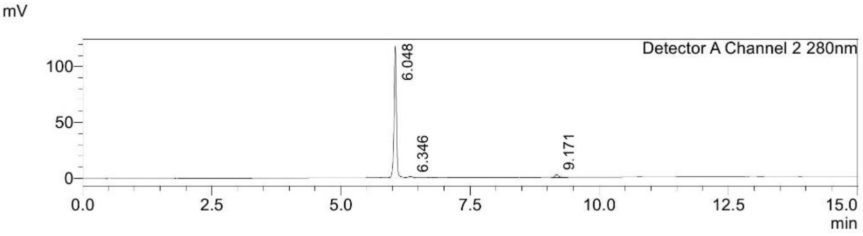

<Peak Table>

Detector A Channel 1 254nm

Peak#	Ret. Time	Height	Width at 5% Height	Area	Area%
1	6.047	129249	0.114	409440	99.275
2	6.347	769	0.119	2990	0.725
Total		130018		412431	100.000

Detector A Channel 2 280nm

Peak#	Ret. Time	Height	Width at 5% Height	Area	Area%
1	6.048	116653	0.115	376026	95.688
2	6.346	1097	--	6438	1.638
3	9.171	2402	0.174	10508	2.674
Total		120152		392971	100.000

216-16

<Sample Information>

Sample Name	: 216-16		
Sample ID	:		
Data Filename	: 216-16.lcd		
Method Filename	: analytic_25min_0.8ml_min_30min_total_4.5_slope.lcm		
Batch Filename	: normal batch.lcb		
Vial #	: 1-44	Sample Type	: Unknown
Injection Volume	: 5 uL		
Date Acquired	: 8/20/2024 6:39:36 PM	Acquired by	: System Administrator
Date Processed	: 8/30/2024 11:46:14 AM	Processed by	: System Administrator

<Chromatogram>

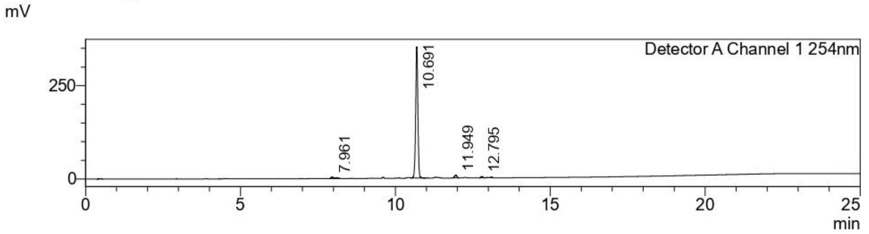

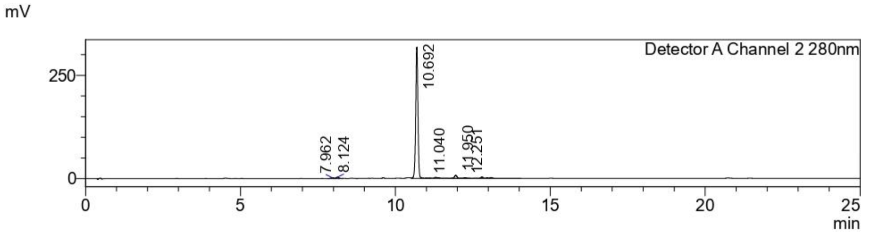

<Peak Table>

Detector A Channel 1 254nm

Peak#	Ret. Time	Height	Width at 5% Height	Area	Area%
1	7.961	3964	0.316	34159	1.865
2	10.691	349329	0.167	1732322	94.578
3	11.949	7770	0.165	40076	2.188
4	12.795	3637	0.129	25085	1.370
Total		364701		1831642	100.000

Detector A Channel 2 280nm

Peak#	Ret. Time	Height	Width at 5% Height	Area	Area%
1	7.962	2039	--	12213	0.697
2	8.124	3231	--	20614	1.177
3	10.692	314200	0.169	1666854	95.150
4	11.040	288	--	1294	0.074
5	11.950	7179	0.188	40294	2.300
6	12.251	1498	0.261	10551	0.602
Total		328436		1751819	100.000

217-10

<Sample Information>

Sample Name	: 217-10--		
Sample ID	:		
Data Filename	: 217-10--.lcd		
Method Filename	: analytic_15min_0.8ml_min_20min_total_9.0_slope.lcm		
Batch Filename	: normal batch.lcb		
Vial #	: 2-42	Sample Type	: Unknown
Injection Volume	: 10 uL		
Date Acquired	: 3/4/2025 5:08:19 PM	Acquired by	: System Administrator
Date Processed	: 3/4/2025 5:23:20 PM	Processed by	: System Administrator

<Chromatogram>

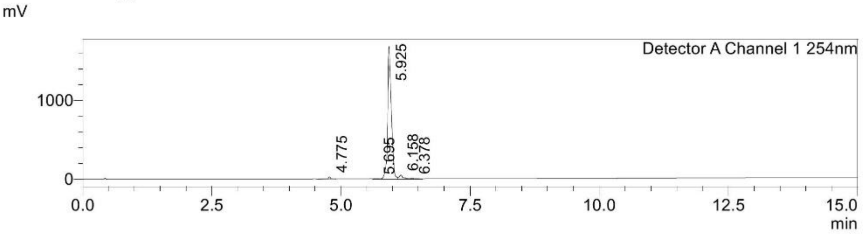

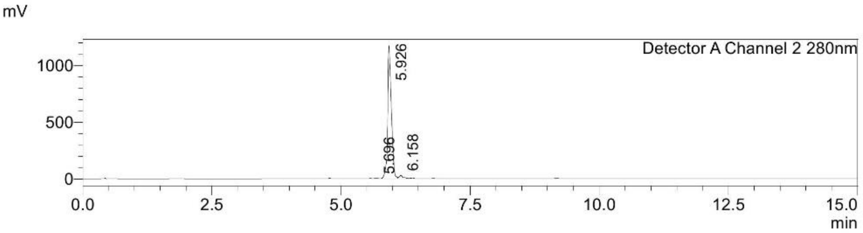

<Peak Table>

Detector A Channel 1 254nm

Peak#	Ret. Time	Height	Width at 5% Height	Area	Area%
1	4.775	24434	0.084	63500	0.734
2	5.695	3853	--	21172	0.245
3	5.925	1658054	0.191	8303810	95.929
4	6.158	47195	--	234102	2.704
5	6.378	8043	--	33582	0.388
Total		1741578		8656165	100.000

Detector A Channel 2 280nm

Peak#	Ret. Time	Height	Width at 5% Height	Area	Area%
1	5.696	2792	--	16919	0.283
2	5.926	1144985	0.192	5799924	96.981
3	6.158	29882	--	163629	2.736
Total		1177660		5980472	100.000

217-11

<Sample Information>

Sample Name	: 217-11		
Sample ID	:		
Data Filename	: 217-11.lcd		
Method Filename	: analytic_25min_0.8ml_min_30min_total_4.5_slope.lcm		
Batch Filename	: normal batch.lcb		
Vial #	: 1-51	Sample Type	: Unknown
Injection Volume	: 1 uL		
Date Acquired	: 8/30/2024 3:12:25 PM	Acquired by	: System Administrator
Date Processed	: 8/30/2024 3:45:01 PM	Processed by	: System Administrator

<Chromatogram>

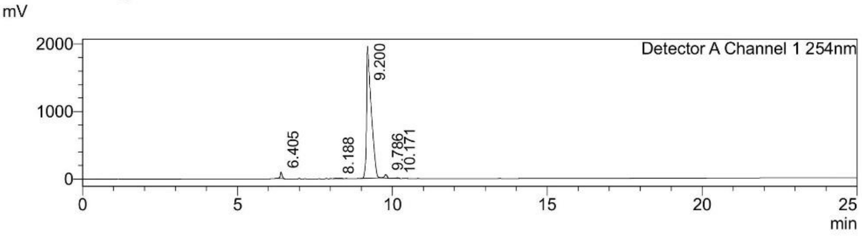

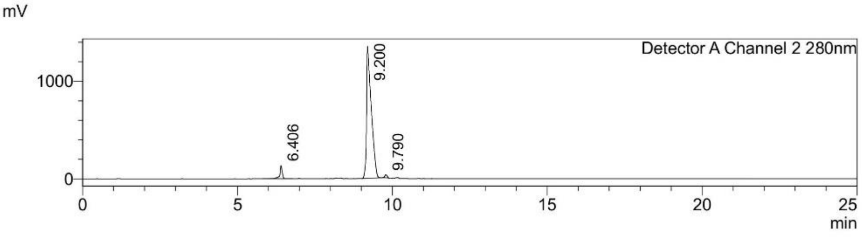

<Peak Table>

Detector A Channel 1 254nm

Peak#	Ret. Time	Height	Width at 5% Height	Area	Area%
1	6.405	95610	0.194	441576	2.067
2	8.188	8901	0.309	101596	0.476
3	9.200	1944172	0.386	20423656	95.606
4	9.786	51405	0.199	310970	1.456
5	10.171	10765	0.256	84484	0.395
Total		2110853		21362282	100.000

Detector A Channel 2 280nm

Peak#	Ret. Time	Height	Width at 5% Height	Area	Area%
1	6.406	134883	0.292	771825	5.072
2	9.200	1343969	0.387	14273931	93.795
3	9.790	30053	0.170	172522	1.134
Total		1508906		15218278	100.000

217-12

<Sample Information>

Sample Name : 217-12
Sample ID :
Data Filename : 217-12.lcd
Method Filename : analytic_25min_0.8ml_min_30min_total_4.5_slope.lcm
Batch Filename : normal batch.lcb
Vial # : 1-52 Sample Type : Unknown
Injection Volume : 1 uL
Date Acquired : 8/30/2024 3:42:36 PM Acquired by : System Administrator
Date Processed : 8/30/2024 4:07:37 PM Processed by : System Administrator

<Chromatogram>

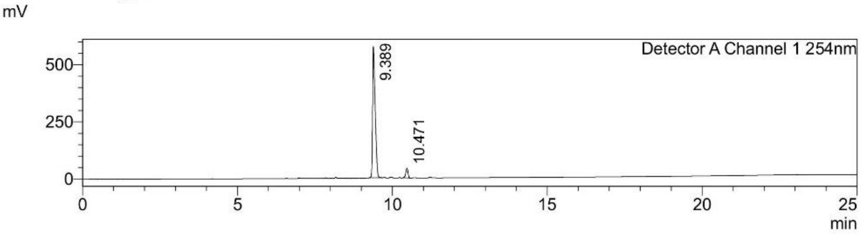

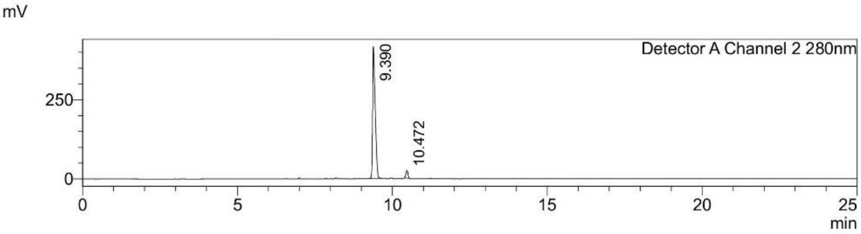

<Peak Table>

Detector A Channel 1 254nm

Peak#	Ret. Time	Height	Width at 5% Height	Area	Area%
1	9.389	572212	0.193	3285629	94.174
2	10.471	42048	0.162	203245	5.826
Total		614260		3488874	100.000

Detector A Channel 2 280nm

Peak#	Ret. Time	Height	Width at 5% Height	Area	Area%
1	9.390	414005	0.192	2368849	95.515
2	10.472	24430	0.144	111222	4.485
Total		438435		2480071	100.000

217-13

<Sample Information>

Sample Name	: 217-13-doublecheck		
Sample ID	:		
Data Filename	: 217-13-doublecheck.lcd		
Method Filename	: analytic_15min_0.8ml_min_20min_total_9.0_slope.lcm		
Batch Filename	: normal batch.lcb		
Vial #	: 2-43	Sample Type	: Unknown
Injection Volume	: 10 uL		
Date Acquired	: 3/4/2025 5:28:31 PM	Acquired by	: System Administrator
Date Processed	: 3/4/2025 5:43:32 PM	Processed by	: System Administrator

<Chromatogram>

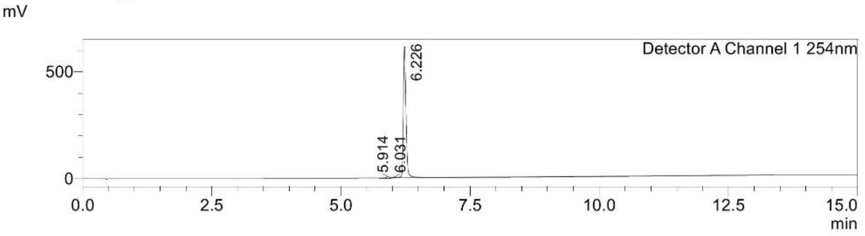

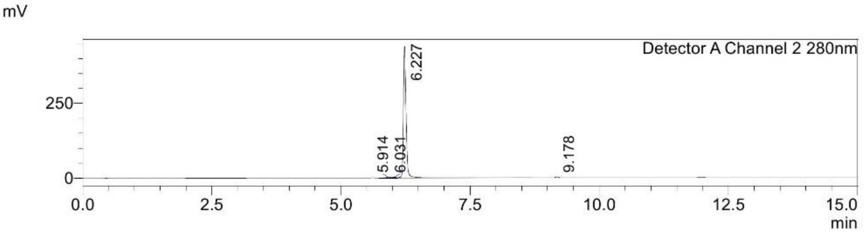

<Peak Table>

Detector A Channel 1 254nm

Peak#	Ret. Time	Height	Width at 5% Height	Area	Area%
1	5.914	4512	--	21565	1.011
2	6.031	3097	--	13620	0.638
3	6.226	597581	0.120	2098473	98.351
Total		605191		2133658	100.000

Detector A Channel 2 280nm

Peak#	Ret. Time	Height	Width at 5% Height	Area	Area%
1	5.914	3166	--	16136	0.989
2	6.031	2580	--	12893	0.790
3	6.227	429688	0.120	1588934	97.413
4	9.178	2900	0.194	13168	0.807
Total		438333		1631130	100.000

224-5

<Sample Information>

Sample Name	: 224-5-tube6		
Sample ID	:		
Data Filename	: 224-5-tube6.lcd		
Method Filename	: analytic_15min_0.8ml_min_20min_total_9.0_slope.lcm		
Batch Filename	: normal batch.lcb		
Vial #	: 2-42	Sample Type	: Unknown
Injection Volume	: 5 uL		
Date Acquired	: 12/26/2024 9:42:06 PM	Acquired by	: System Administrator
Date Processed	: 12/26/2024 9:57:07 PM	Processed by	: System Administrator

<Chromatogram>

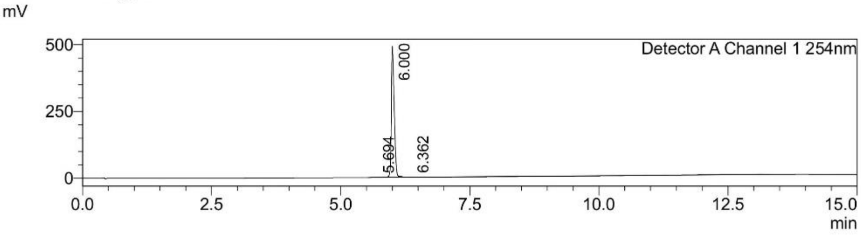

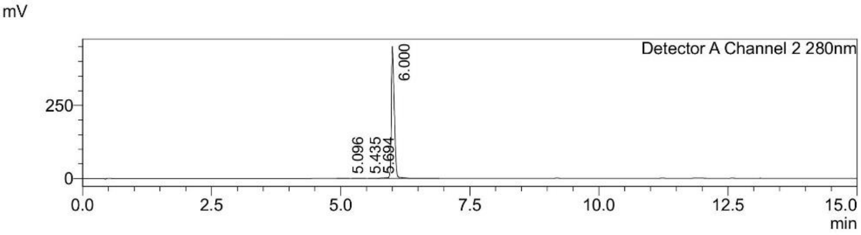

<Peak Table>

Detector A Channel 1 254nm

Peak#	Ret. Time	Height	Width at 5% Height	Area	Area%
1	5.694	505	--	1819	0.103
2	6.000	482926	0.132	1766235	99.835
3	6.362	297	0.106	1101	0.062
Total		483729		1769156	100.000

Detector A Channel 2 280nm

Peak#	Ret. Time	Height	Width at 5% Height	Area	Area%
1	5.096	170	--	1161	0.065
2	5.435	245	--	1443	0.080
3	5.694	564	--	3176	0.177
4	6.000	444226	0.132	1789964	99.678
Total		445205		1795744	100.000

224-6

<Sample Information>

Sample Name	: 224-5-tube7
Sample ID	:
Data Filename	: 224-5-tube7.lcd
Method Filename	: analytic_15min_0.8ml_min_20min_total_9.0_slope.lcm
Batch Filename	: normal batch.lcb
Vial #	: 2-43
Injection Volume	: 5 uL
Date Acquired	: 12/26/2024 10:02:17 PM
Date Processed	: 12/26/2024 10:17:18 PM
Sample Type	: Unknown
Acquired by	: System Administrator
Processed by	: System Administrator

<Chromatogram>

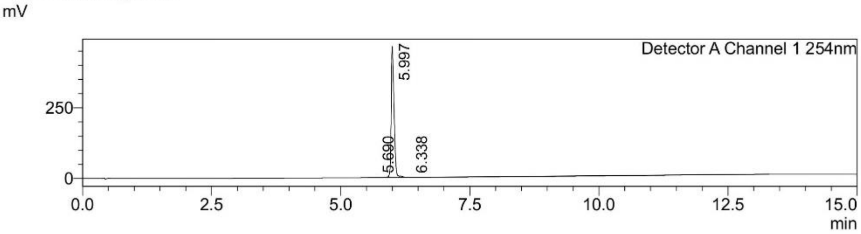

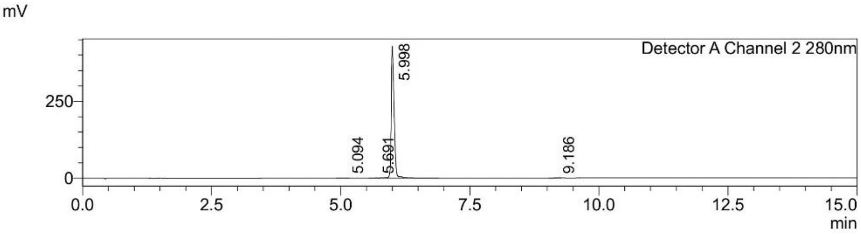

<Peak Table>

Detector A Channel 1 254nm

Peak#	Ret. Time	Height	Width at 5% Height	Area	Area%
1	5.690	442	--	1397	0.085
2	5.997	460483	0.129	1631088	99.830
3	6.338	359	--	1386	0.085
Total		461284		1633871	100.000

Detector A Channel 2 280nm

Peak#	Ret. Time	Height	Width at 5% Height	Area	Area%
1	5.094	166	--	1140	0.068
2	5.691	510	--	3033	0.181
3	5.998	425724	0.130	1672702	99.588
4	9.186	674	--	2754	0.164
Total		427074		1679629	100.000

224-7

<Sample Information>

Sample Name	: 224-7-RECHECK		
Sample ID	:		
Data Filename	: 224-7-RECHECK.lcd		
Method Filename	: analytic_15min_0.8ml_min_20min_total_9.0_slope.lcm		
Batch Filename	: normal batch.lcb		
Vial #	: 1-51	Sample Type	: Unknown
Injection Volume	: 10 uL		
Date Acquired	: 3/5/2025 10:28:40 PM	Acquired by	: System Administrator
Date Processed	: 3/5/2025 10:43:41 PM	Processed by	: System Administrator

<Chromatogram>

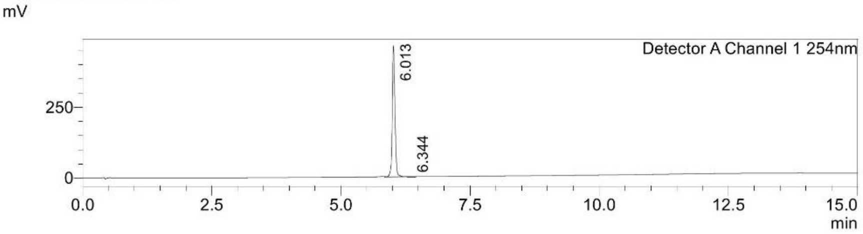

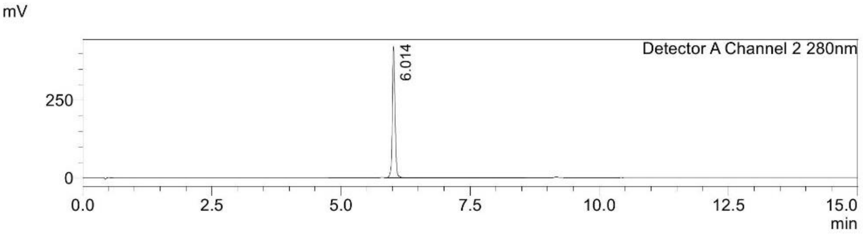

<Peak Table>

Detector A Channel 1 254nm

Peak#	Ret. Time	Height	Width at 5% Height	Area	Area%
1	6.013	454856	0.130	1645972	99.808
2	6.344	456	--	3160	0.192
Total		455312		1649132	100.000

Detector A Channel 2 280nm

Peak#	Ret. Time	Height	Width at 5% Height	Area	Area%
1	6.014	419876	0.130	1531219	100.000
Total		419876		1531219	100.000

224-8

<Sample Information>

Sample Name	: 244-8-1		
Sample ID	:		
Data Filename	: 244-8-1.lcd		
Method Filename	: analytic_15min_0.8ml_min_20min_total_9.0_slope.lcm		
Batch Filename	: normal batch.lcb		
Vial #	: 3-46	Sample Type	: Unknown
Injection Volume	: 5 uL		
Date Acquired	: 12/17/2024 6:19:35 PM	Acquired by	: System Administrator
Date Processed	: 12/17/2024 6:34:36 PM	Processed by	: System Administrator

<Chromatogram>

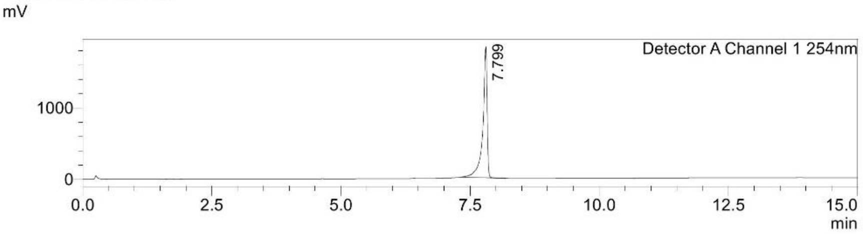

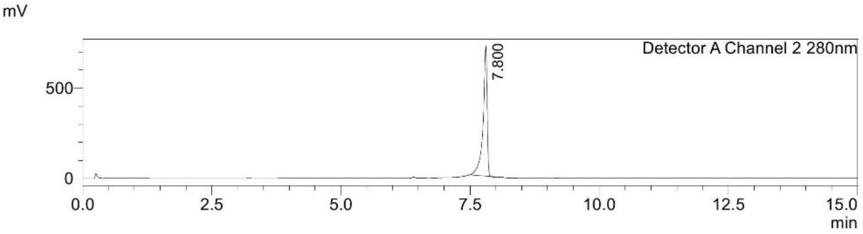

<Peak Table>

Detector A Channel 1 254nm

Peak#	Ret. Time	Height	Width at 5% Height	Area	Area%
1	7.799	1819072	0.273	10564104	100.000
Total		1819072		10564104	100.000

Detector A Channel 2 280nm

Peak#	Ret. Time	Height	Width at 5% Height	Area	Area%
1	7.800	708214	0.245	3902825	100.000
Total		708214		3902825	100.000

225-2

<Sample Information>

Sample Name	: 225-2-1		
Sample ID	:		
Data Filename	: 225-2-1.lcd		
Method Filename	: analytic_15min_0.8ml_min_20min_total_9.0_slope.lcm		
Batch Filename	: normal batch.lcb		
Vial #	: 2-46	Sample Type	: Unknown
Injection Volume	: 10 uL		
Date Acquired	: 1/9/2025 9:57:28 AM	Acquired by	: System Administrator
Date Processed	: 1/9/2025 10:12:29 AM	Processed by	: System Administrator

<Chromatogram>

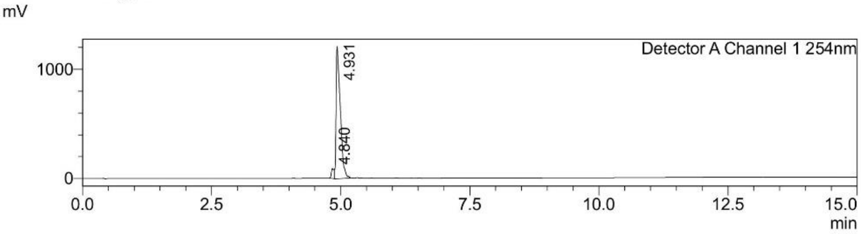

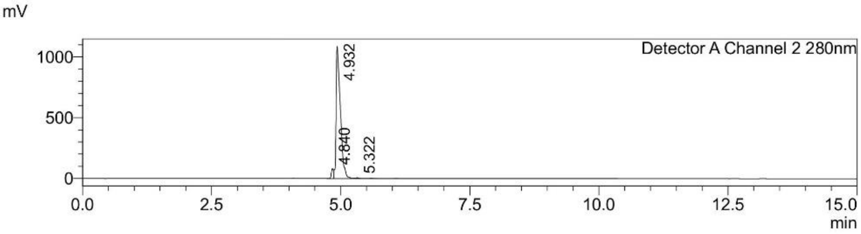

<Peak Table>

Detector A Channel 1 254nm

Peak#	Ret. Time	Height	Width at 5% Height	Area	Area%
1	4.840	88956	--	306644	4.272
2	4.931	1204736	--	6872041	95.728
Total		1293692		7178684	100.000

Detector A Channel 2 280nm

Peak#	Ret. Time	Height	Width at 5% Height	Area	Area%
1	4.840	81319	--	253396	3.729
2	4.932	1084335	--	6530361	96.099
3	5.322	3896	0.097	11665	0.172
Total		1169550		6795422	100.000

225-3

<Sample Information>

Sample Name	: 225-3-tube3
Sample ID	:
Data Filename	: 225-3-tube3.lcd
Method Filename	: analytic_15min_0.8ml_min_20min_total_9.0_slope.lcm
Batch Filename	: normal batch.lcb
Vial #	: 1-53
Sample Type	: Unknown
Injection Volume	: 10 uL
Date Acquired	: 3/7/2025 9:52:07 PM
Acquired by	: System Administrator
Date Processed	: 3/7/2025 10:07:07 PM
Processed by	: System Administrator

<Chromatogram>

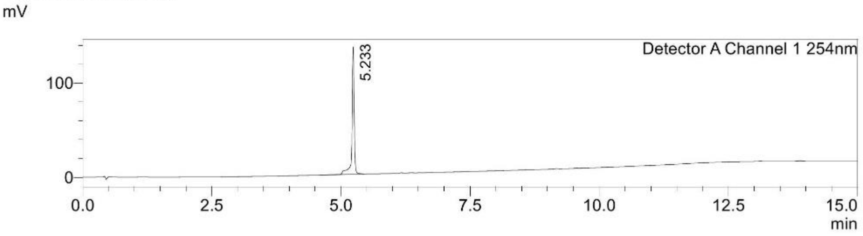

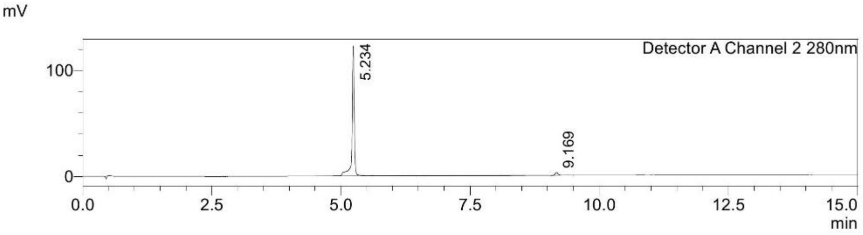

<Peak Table>

Detector A Channel 1 254nm

Peak#	Ret. Time	Height	Width at 5% Height	Area	Area%
1	5.233	130712	0.130	408749	100.000
Total		130712		408749	100.000

Detector A Channel 2 280nm

Peak#	Ret. Time	Height	Width at 5% Height	Area	Area%
1	5.234	117436	0.134	385080	97.050
2	9.169	2683	0.168	11704	2.950
Total		120119		396784	100.000

225-4

<Sample Information>

Sample Name	: 225-4-10		
Sample ID	:		
Data Filename	: 225-4-10.lcd		
Method Filename	: analytic_15min_0.8ml_min_20min_total_9.0_slope.lcm		
Batch Filename	: normal batch.lcb		
Vial #	: 2-49	Sample Type	: Unknown
Injection Volume	: 5 uL		
Date Acquired	: 12/31/2024 10:53:33 AM	Acquired by	: System Administrator
Date Processed	: 12/31/2024 11:08:33 AM	Processed by	: System Administrator

<Chromatogram>

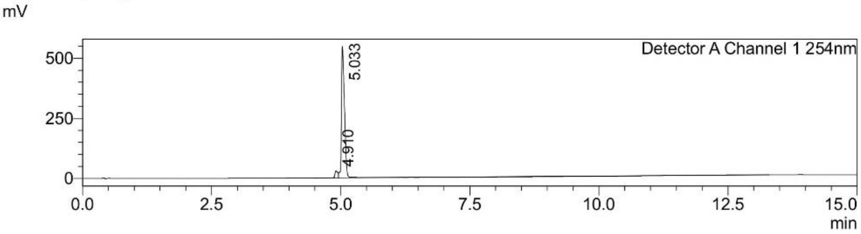

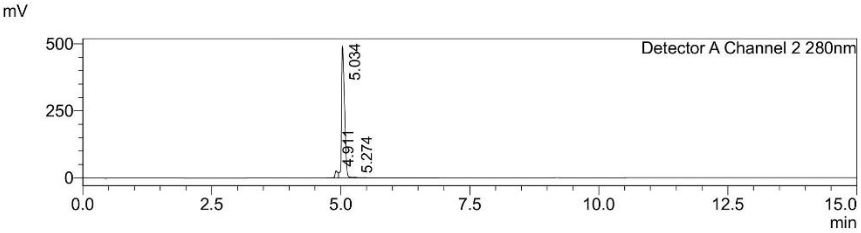

<Peak Table>

Detector A Channel 1 254nm

Peak#	Ret. Time	Height	Width at 5% Height	Area	Area%
1	4.910	28656	--	113290	5.562
2	5.033	537919	0.132	1923674	94.438
Total		566575		2036964	100.000

Detector A Channel 2 280nm

Peak#	Ret. Time	Height	Width at 5% Height	Area	Area%
1	4.911	26161	--	103574	4.906
2	5.034	482372	0.133	2006204	95.021
3	5.274	774	0.059	1550	0.073
Total		509307		2111329	100.000

225-5

<Sample Information>

Sample Name	: 225-T50		
Sample ID	:		
Data Filename	: 225-T50--.lcd		
Method Filename	: analytic_15min_0.8ml_min_20min_total_9.0_slope.lcm		
Batch Filename	: normal batch.lcb		
Vial #	: 1-31	Sample Type	: Unknown
Injection Volume	: 2 uL		
Date Acquired	: 1/20/2025 11:34:14 PM	Acquired by	: System Administrator
Date Processed	: 1/20/2025 11:49:15 PM	Processed by	: System Administrator

<Chromatogram>

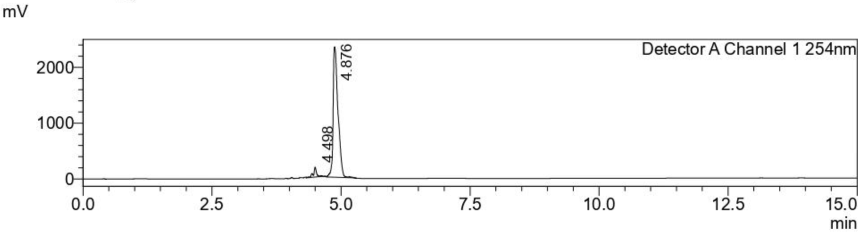

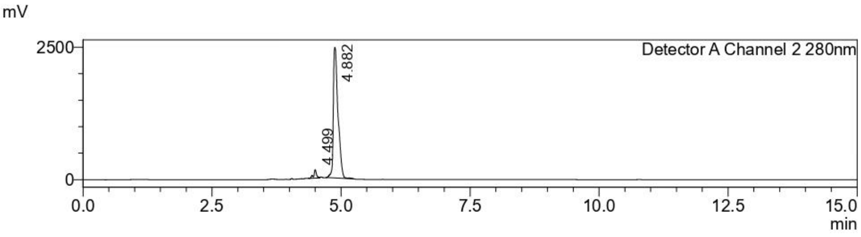

<Peak Table>

Detector A Channel 1 254nm

Peak#	Ret. Time	Height	Width at 5% Height	Area	Area%
1	4.498	176328	0.175	753468	4.624
2	4.876	2323715	0.234	15542152	95.376
Total		2500042		16295620	100.000

Detector A Channel 2 280nm

Peak#	Ret. Time	Height	Width at 5% Height	Area	Area%
1	4.499	157800	0.166	578976	3.575
2	4.882	2462212	0.217	15615281	96.425
Total		2620012		16194258	100.000

225-6

<Sample Information>

Sample Name	: 225-6-tube2		
Sample ID	:		
Data Filename	: 225-6-tube2.lcd		
Method Filename	: analytic_15min_0.8ml_min_20min_total_9.0_slope.lcm		
Batch Filename	: normal batch.lcb		
Vial #	: 1-47	Sample Type	: Unknown
Injection Volume	: 10 uL		
Date Acquired	: 3/7/2025 6:19:52 PM	Acquired by	: System Administrator
Date Processed	: 3/7/2025 6:34:53 PM	Processed by	: System Administrator

<Chromatogram>

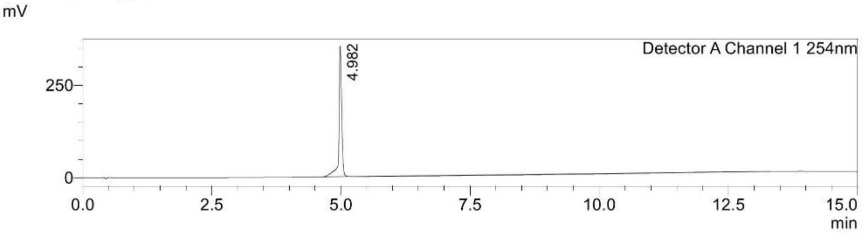

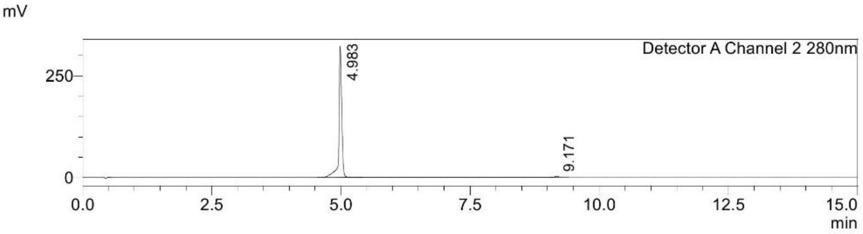

<Peak Table>

Detector A Channel 1 254nm

Peak#	Ret. Time	Height	Width at 5% Height	Area	Area%
1	4.982	347307	0.173	1016983	100.000
Total		347307		1016983	100.000

Detector A Channel 2 280nm

Peak#	Ret. Time	Height	Width at 5% Height	Area	Area%
1	4.983	317501	0.172	1214140	99.003
2	9.171	2719	0.193	12226	0.997
Total		320220		1226367	100.000

225-7

<Sample Information>

Sample Name	: 225-7-tube1
Sample ID	:
Data Filename	: 225-7-tube1.lcd
Method Filename	: analytic_15min_0.8ml_min_20min_total_9.0_slope.lcm
Batch Filename	: normal batch.lcb
Vial #	: 1-50
Sample Type	: Unknown
Injection Volume	: 10 uL
Date Acquired	: 3/7/2025 8:51:29 PM
Acquired by	: System Administrator
Date Processed	: 3/7/2025 9:06:30 PM
Processed by	: System Administrator

<Chromatogram>

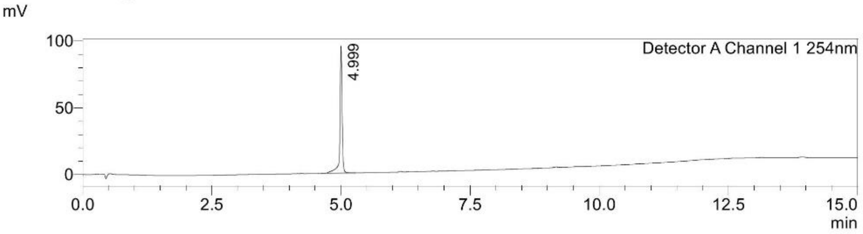

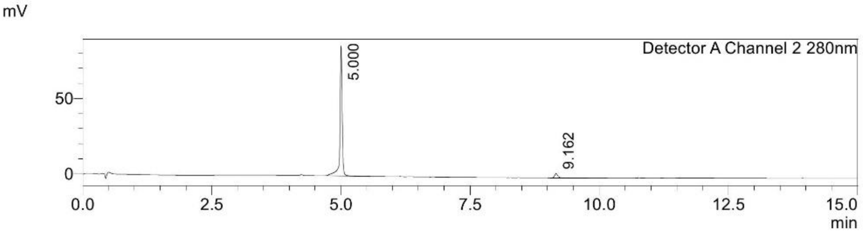

<Peak Table>

Detector A Channel 1 254nm

Peak#	Ret. Time	Height	Width at 5% Height	Area	Area%
1	4.999	92566	0.125	282013	100.000
Total		92566		282013	100.000

Detector A Channel 2 280nm

Peak#	Ret. Time	Height	Width at 5% Height	Area	Area%
1	5.000	83720	0.125	256260	95.001
2	9.162	2818	0.197	13483	4.999
Total		86538		269744	100.000

229-1

<Sample Information>

Sample Name	: 229-1-recheck		
Sample ID	:		
Data Filename	: 229-1-recheck.lcd		
Method Filename	: analytic_15min_0.8ml_min_20min_total_9.0_slope.lcm		
Batch Filename	: normal batch.lcb		
Vial #	: 1-53	Sample Type	: Unknown
Injection Volume	: 10 uL		
Date Acquired	: 3/5/2025 11:29:15 PM	Acquired by	: System Administrator
Date Processed	: 3/5/2025 11:44:15 PM	Processed by	: System Administrator

<Chromatogram>

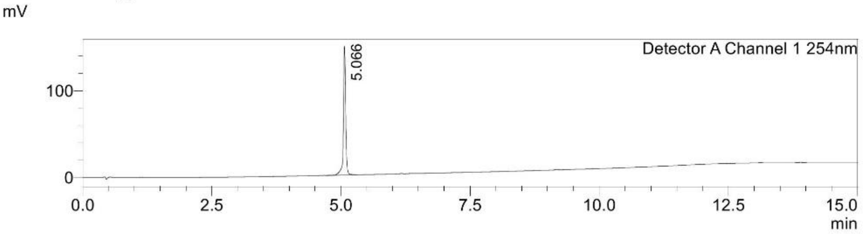

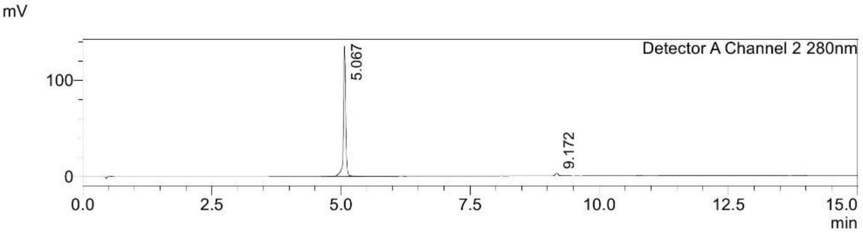

<Peak Table>

Detector A Channel 1 254nm

Peak#	Ret. Time	Height	Width at 5% Height	Area	Area%
1	5.066	145522	0.123	441123	100.000
Total		145522		441123	100.000

Detector A Channel 2 280nm

Peak#	Ret. Time	Height	Width at 5% Height	Area	Area%
1	5.067	130546	0.125	402709	96.897
2	9.172	2846	0.191	12898	3.103
Total		133392		415607	100.000

229-2

<Sample Information>

Sample Name : 229-2-tube4
Sample ID :
Data Filename : 229-2-tube6.lcd
Method Filename : analytic_15min_0.8ml_min_20min_total_9.0_slope.lcm
Batch Filename : normal batch.lcb
Vial # : 1-47 Sample Type : Unknown
Injection Volume : 10 uL
Date Acquired : 3/10/2025 3:41:09 PM Acquired by : System Administrator
Date Processed : 3/10/2025 3:56:10 PM Processed by : System Administrator

<Chromatogram>

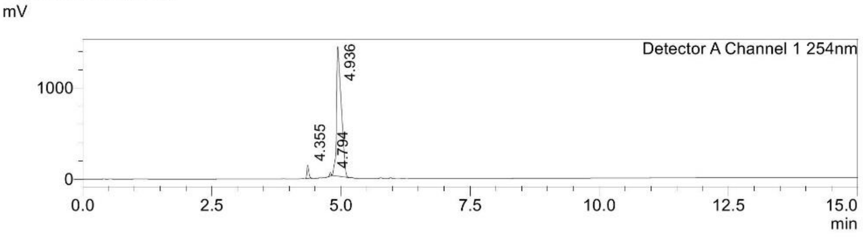

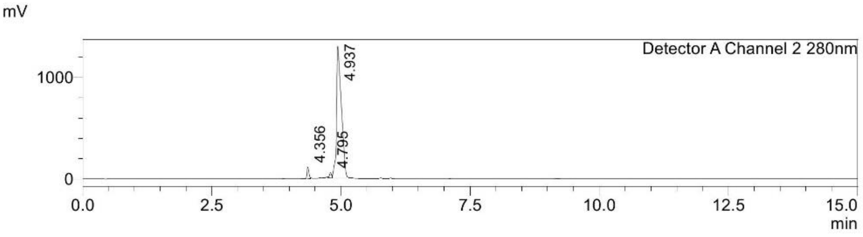

<Peak Table>

Detector A Channel 1 254nm

Peak#	Ret. Time	Height	Width at 5% Height	Area	Area%
1	4.355	139571	0.083	344602	3.622
2	4.794	37281	0.060	74251	0.780
3	4.936	1405375	0.235	9095064	95.597
Total		1582226		9513917	100.000

Detector A Channel 2 280nm

Peak#	Ret. Time	Height	Width at 5% Height	Area	Area%
1	4.356	111386	--	283439	3.208
2	4.795	48652	--	127221	1.440
3	4.937	1284094	0.242	8425191	95.352
Total		1444131		8835851	100.000

229-3

<Sample Information>

Sample Name	: 229-3-tube1		
Sample ID	:		
Data Filename	: 229-3-tube1.lcd		
Method Filename	: analytic_15min_0.8ml_min_20min_total_9.0_slope.lcm		
Batch Filename	: normal batch.lcb		
Vial #	: 1-28	Sample Type	: Unknown
Injection Volume	: 10 uL		
Date Acquired	: 3/7/2025 10:32:33 PM	Acquired by	: System Administrator
Date Processed	: 3/7/2025 10:47:34 PM	Processed by	: System Administrator

<Chromatogram>

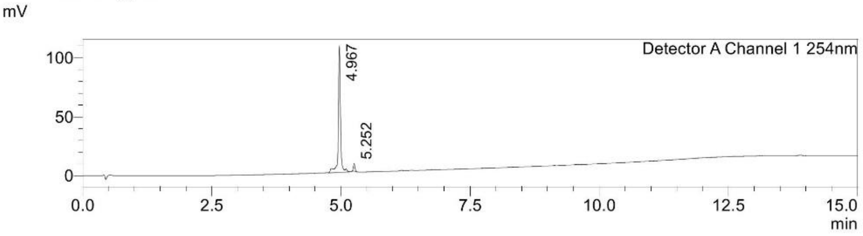

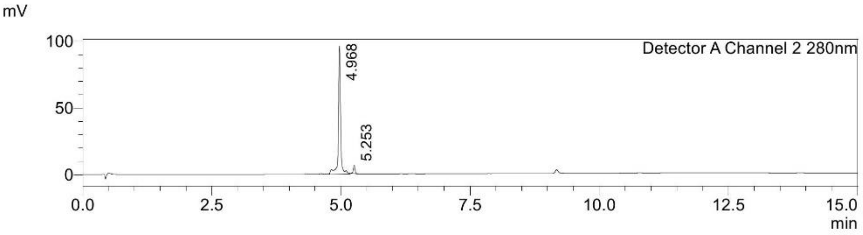

<Peak Table>

Detector A Channel 1 254nm

Peak#	Ret. Time	Height	Width at 5% Height	Area	Area%
1	4.967	102415	0.121	315552	96.124
2	5.252	5937	0.064	12722	3.876
Total		108352		328274	100.000

Detector A Channel 2 280nm

Peak#	Ret. Time	Height	Width at 5% Height	Area	Area%
1	4.968	92093	0.122	288940	95.395
2	5.253	5927	0.078	13948	4.605
Total		98020		302888	100.000

229-4

<Sample Information>

Sample Name	: 229-4-tube1		
Sample ID	:		
Data Filename	: 229-4-tube1.lcd		
Method Filename	: analytic_15min_0.8ml_min_20min_total_9.0_slope.lcm		
Batch Filename	: normal batch.lcb		
Vial #	: 1-51	Sample Type	: Unknown
Injection Volume	: 10 uL		
Date Acquired	: 3/10/2025 5:11:59 PM	Acquired by	: System Administrator
Date Processed	: 3/10/2025 5:26:59 PM	Processed by	: System Administrator

<Chromatogram>

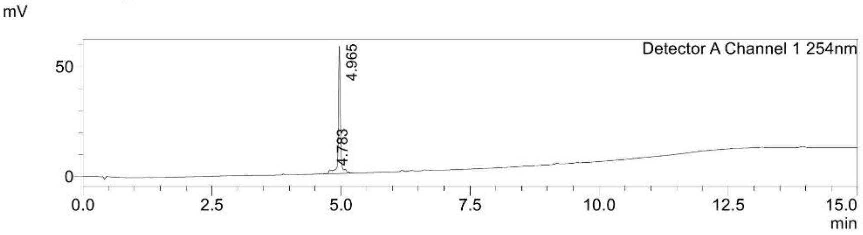

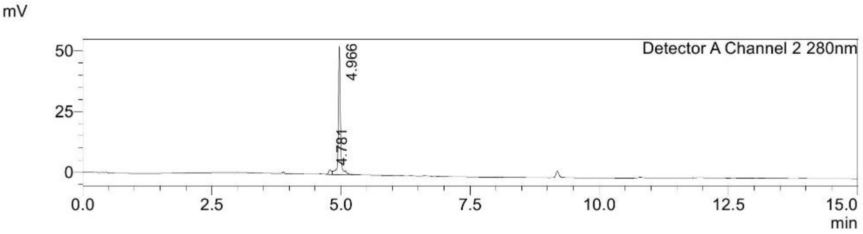

<Peak Table>

Detector A Channel 1 254nm

Peak#	Ret. Time	Height	Width at 5% Height	Area	Area%
1	4.783	1766	--	7463	4.329
2	4.965	56988	0.103	164912	95.671
Total		58755		172375	100.000

Detector A Channel 2 280nm

Peak#	Ret. Time	Height	Width at 5% Height	Area	Area%
1	4.781	1900	--	7796	4.972
2	4.966	51328	0.103	149001	95.028
Total		53227		156797	100.000

HRMS spectrum of 204-2, 216-16, 225-5

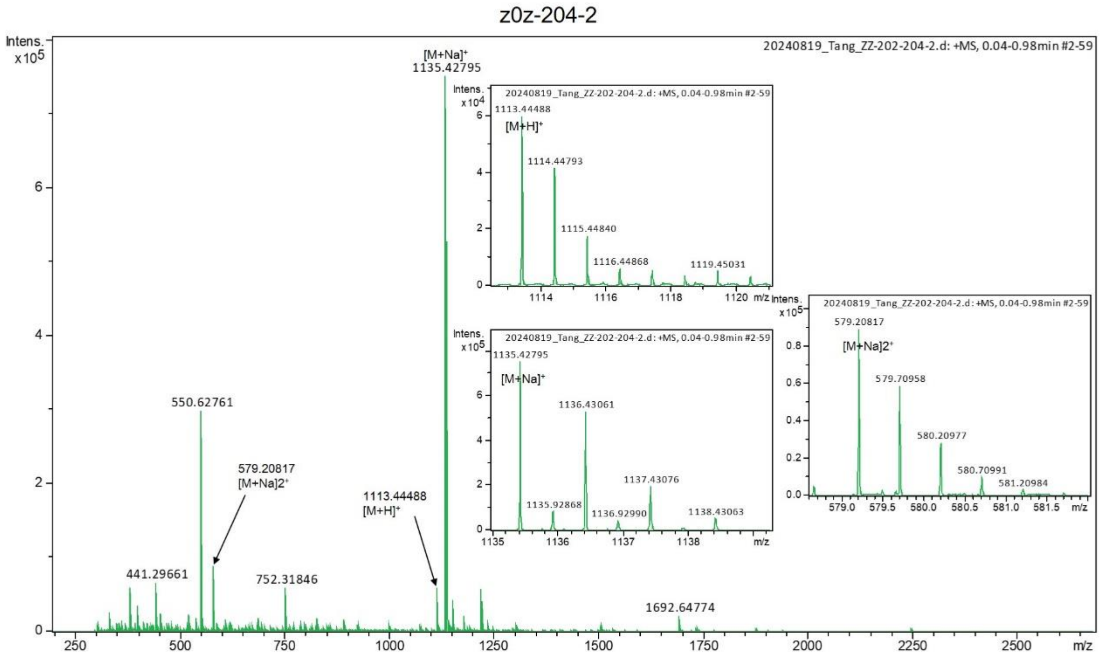

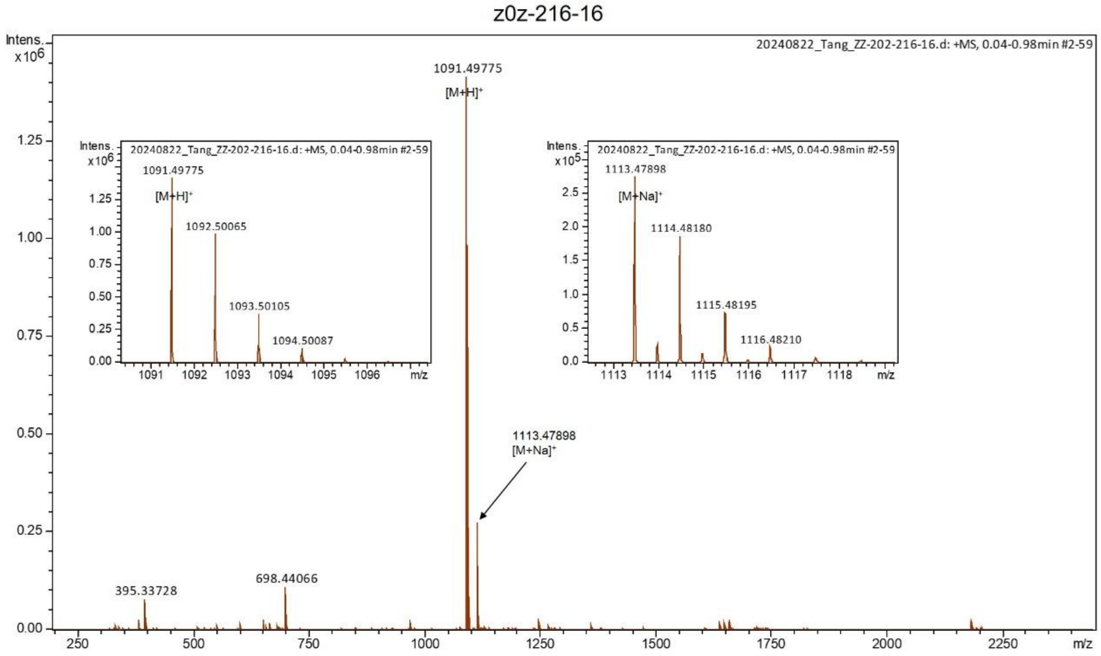

z0z-225-5

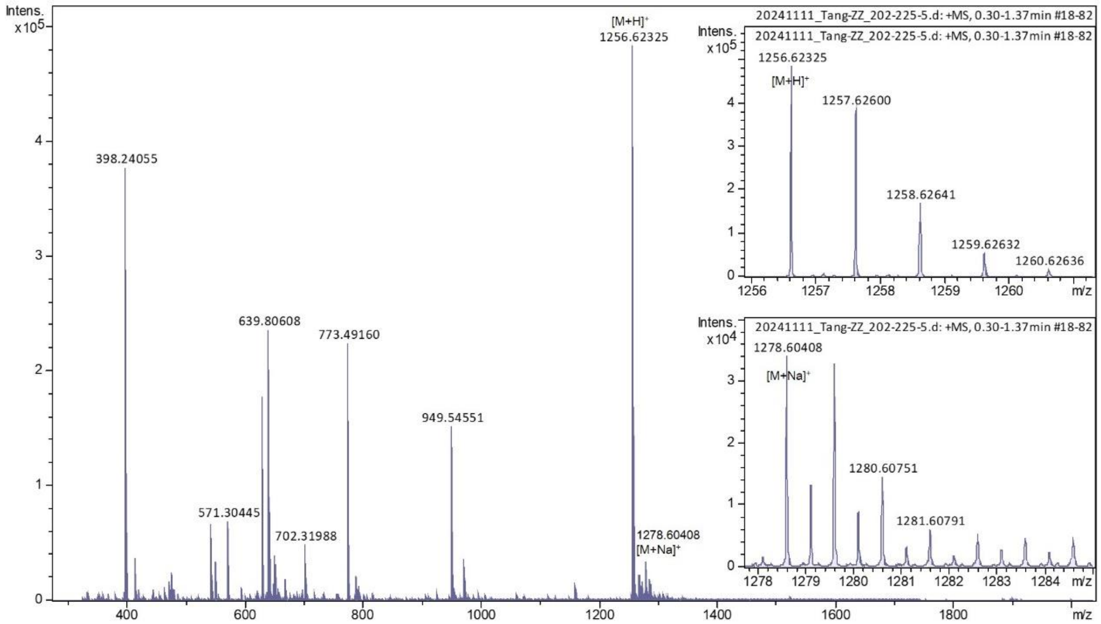
Intens.
x10^5
[M+H]+
1256.62325
398.24055
639.80608
773.49160
949.54551
571.30445
702.31988
1278.60408
[M+Na]+
m/z
20241111_Tang-ZZ_202-225-5.d: +MS, 0.30-1.37min #18-82
1256.62325
[M+H]+
1257.62600
1258.62641
1259.62632
1260.62636
1278.60408
[M+Na]+
1280.60751
1281.60791
